# A model of the regulatory network involved in the control of the cell cycle and cell differentiation in the *Caenorhabditis elegans* vulva

**DOI:** 10.1186/s12859-015-0498-z

**Published:** 2015-03-13

**Authors:** Nathan Weinstein, Elizabeth Ortiz-Gutiérrez, Stalin Muñoz, David A Rosenblueth, Elena R Álvarez-Buylla, Luis Mendoza

**Affiliations:** 1 Programa de Doctorado en Ciencias Biomédicas, Universidad Nacional Autónoma de, México, DF México; 2 Instituto de Investigaciones Biomédicas, Universidad Nacional Autónoma de México, México, DF México; 3 Instituto de Ecología, Universidad Nacional Autónoma de México, México, DF México; 4 Instituto de Investigaciones en Matemáticas Aplicadas y en Sistemas, Universidad, Nacional Autónoma de México, México, DF México; 5 Centro de Ciencias de la Complejidad, Universidad Nacional Autónoma de México, México, DF México

**Keywords:** *C. elegans*, Vulva, Fate determination, Cell cycle

## Abstract

**Background:**

There are recent experimental reports on the cross-regulation between molecules involved in the control of the cell cycle and the differentiation of the vulval precursor cells (VPCs) of *Caenorhabditis elegans*. Such discoveries provide novel clues on how the molecular mechanisms involved in the cell cycle and cell differentiation processes are coordinated during vulval development. Dynamic computational models are helpful to understand the integrated regulatory mechanisms affecting these cellular processes.

**Results:**

Here we propose a simplified model of the regulatory network that includes sufficient molecules involved in the control of both the cell cycle and cell differentiation in the *C. elegans* vulva to recover their dynamic behavior. We first infer both the topology and the update rules of the cell cycle module from an expected time series. Next, we use a symbolic algorithmic approach to find which interactions must be included in the regulatory network. Finally, we use a continuous-time version of the update rules for the cell cycle module to validate the cyclic behavior of the network, as well as to rule out the presence of potential artifacts due to the synchronous updating of the discrete model. We analyze the dynamical behavior of the model for the wild type and several mutants, finding that most of the results are consistent with published experimental results.

**Conclusions:**

Our model shows that the regulation of Notch signaling by the cell cycle preserves the potential of the VPCs and the three vulval fates to differentiate and de-differentiate, allowing them to remain completely responsive to the concentration of LIN-3 and lateral signal in the extracellular microenvironment.

**Electronic supplementary material:**

The online version of this article (doi:10.1186/s12859-015-0498-z) contains supplementary material, which is available to authorized users.

## Background

The nematode *Caenorhabditis elegans* has been extensively used as a model organism in research areas such as genetics, genomics, cellular signaling cascades, neuroscience, aging, developmental biology, and cell differentiation [1-4]. *C. elegans* is specially suitable for the study of cell differentiation because its cell lineage map is both fully characterized and almost invariant [5,6]. In particular, the vulva of *C. elegans* has been used as an experimental model for the study of organ formation, cellular fusion, and intracellular signaling [7-11].

**Figure 1:**
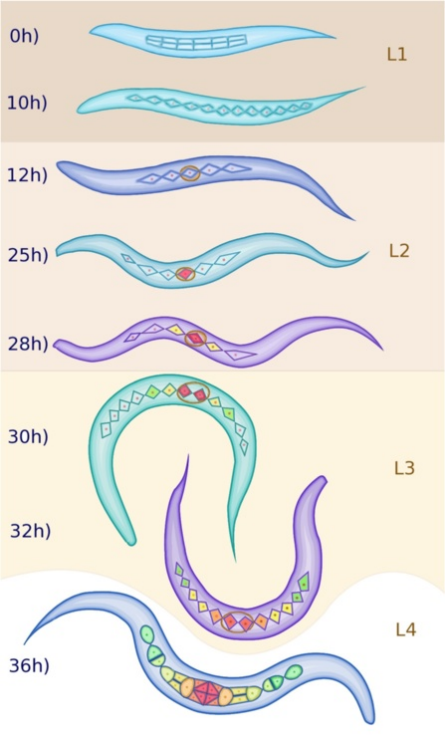
**Formation and specialization of the vulval cells during the first hours of development of** ***C. elegans*** **.** Larval phase L1: 0 h) After eclosion, the worm has two rows of P cells in the middle ventral region. 10 h) The rows merge. Larval phase L2: 12 h) The cells P1-P12 undergo a longitudinal division, the anchor cell forms (brown oval), and P3.p-P8.p become vulval precursor cells (VPCs). 25 h) P6.p responds to LIN-3/EGF secreted by the AC and acquires the primary fate (red), this cell secrets the DSL ligands that constitute the lateral signal. 28 h) P5.p and P7.p respond to the lateral signal of P6.p, thus acquiring the secondary fate (yellow). The rest of the VPCs acquire the tertiary fate forming the pattern 3^*rd*^3^*rd*^2^*nd*^1^*st*^2^*nd*^3^*rd*^. Larval phase L3: 30 h) Cells P3.p to P8.p divide longitudinally, and the daughters of the secondary fate cells are polarized. 32 h) The descendants of the tertiary fate cells fuse with hyp7 and the rest divide longitudinally once more, and the most proximal granddaughters of P6.p are induced again by the anchor cell (AC). Larval phase L4: 36 h) Formation of the adult vulval cells: some descendants of the VPCs divide a third time with the pattern LLTN TTTT NTLL. L stands for a lateral division, forming anterior and posterior daughters. T is a transverse division, forming left and right daughters. N stands for no division. Cells are classified, in proximal to distal order as vulF (red), vulE (orange red), vulD (orange), vulC (yellow), vulB2 and vulB1 (green yellow), and vulA (green).

The vulva has two main biological functions, namely, copulation and egg laying. This organ is formed by seven epithelial rings connecting the uterus with the ventral hypodermis, forming a path from the interior of the uterus to the external environment. This path is closed to keep pathogens out of the worm, except when the vulval muscles open it to perform its functions. Each ring of the vulva is formed by cells of a different kind, namely (in ventral-to-dorsal order): vulA, vulB1, vulB2, vulC, vulD, vulE, and vulF, containing a total of 22 nuclei (Figure 1). In the adult, most of these rings are formed by a single tetranucleated syncytium, the exceptions being the binucleated syncytium ring vulD, as well as the vulB1 and vulB2 rings that contain two half-ring binucleated syncytia each [6].

The cellular and molecular mechanisms controlling the development of the vulva have been experimentally studied for more than three decades by means of cell ablations [12-14], mutations causing some specific vulval phenotypes [15-18], or mutations that rescue vulval phenotypes [19-24]. Furthermore, the roles of the Ras/MAPK, Fgf, Notch, and Wnt signaling cascades during the formation of the vulva have been extensively studied [11,25-27].

The first stage of vulval development is the formation of the vulval competence group. The nematode is born with two rows of P cells containing six cells each (Figure 1, 0h); these cells migrate towards the ventral mid-line forming one row (Figure 1, 10 h). The P cells then undergo a longitudinal division; the anterior daughter cells acquire neuronal fates, while the posterior daughter cells acquire hypodermal fates. Six of the posterior daughters, namely P3.p, P4.p, P5.p, P6.p, P7.p, and P8.p (Figure 1, 12 h), are induced by Wnt and Ras signaling to become the vulval competence group [28-30].

The second stage of the process is defined by the differentiation and proliferation, instead of the formation of vulval cells. It is known that the fate of VPCs (Figure 1, 25 h and 28 h) is determined by the induction from the anchor cell (AC, a gonadal cell located dorsally with respect to the cell P6.p), the lateral signaling among the VPCs, and the concentration of Wnt ligands secreted by the AC as well as cells near the tail. The VPCs may acquire one of three fates, P6.p acquires the primary fate that is characterized by the expression of *egl-17, lin-39, apx-1*, and *dsl-1* as well as the transverse division of its granddaughters. P5.p and P7.p acquire the secondary fate that is characterized by the expression of *lin-11* and *lip-1* and the diverse planes of division of its granddaughters. Specifically, the most proximal granddaughters do not divide, the next most proximal granddaughters divide transversely, and the two most distal granddaughters divide longitudinally. P3.p, P4.p, and P8.p acquire the tertiary fate, tertiary fate VPCs divide longitudinally once, and their daughters fuse with hyp7. Then, the VPCs divide longitudinally once and the cells that acquired the tertiary fate fuse with the ventral hypoderm. Also, the daughters of the secondary fate cells are polarized by Wnt signaling (Figure 1, 30 h). At this point the six remaining VPC daughters undergo a second longitudinal division (Figure 1, 32 h). Finally, most of the granddaughters of the VPCs divide a third time, except for the most proximal descendants of the secondary fate cells.

The third stage of the process is morphogenesis and determination of the final fates of vulval cells. The vulval cells migrate towards the AC, and then they fuse forming the seven rings that give the adult vulva its final shape. During this stage the AC breaks the membrane that separates the gonad from the epidermis, connecting both tissues and opening the vulval channel. The developing vulva directs the growth and attachment of the vulval muscles [6-8,11], and the adult fates of the vulval cells are determined. Remarkably, there is scarce information regarding the molecular network that controls this third stage of the cell differentiation in the vulva [11,31].

The cell cycle and fate determination in *C. elegans* are synchronized due to the interconnection of the molecular mechanisms controlling both processes, which is described in detail as part of the molecular basis of the regulatory network. Additionally, the heterochronic genes *lin-4*, *lin-14*, and *lin-28* are important for the control of developmental timing. In the vulva, LIN-14 activity is required during L1, LIN-28 activity is necessary during L2 and early L3 to prevent premature vulval cell divisions, and *lin-4* activity is required during L3 for the cellular divisions that occur during this stage, and the proper determination of the secondary fate [11,32,33].

There are several models describing the process of cell specialization in the vulva of *C. elegans* [34]. The first models were diagrammatic and static [14,35], describing how the inductive and lateral signals interact to determine the fates of the VPCs. Later models emphasized the importance of the concentration of the inductive and lateral signals, producing bi-dimensional fate maps [36-38], and epigenetic landscapes [39]. Some models were developed with a focus on the importance of the order in the sequence of signals [40-42], others incorporated an evolutionary perspective [37,38], and still others were built to test new methodologies or tools for the simulation of molecular network models [43-47]. Recently, we proposed a dynamic regulatory network model to include the molecules that are involved in the control of cell fusion and cell polarization during the first stages of vulva development. Such a model included the Wnt, Ras, and Notch signaling pathways, as well as the interactions among them, and the relevant Hox genes [30].

The cell cycle has been extensively studied in several species, and as a result there is a large number of mathematical and computational models for eukaryotes in general [48-51], mammals [52,53], and also for several specific model systems, including fission yeast [54-58], budding yeast [59-61], amphibian embryos [62,63], *A. thaliana* ([64], Ortiz-Gutiérrez *et al.* in preparation), and notably the embryonic cell cycle of *C. elegans* [65]. Many discrete and continuous dynamical models have been used to find the molecular interactions that are necessary and sufficient to recover the observed cyclic behavior of several cell cycle regulators. Some models have focused on the cell cycle checkpoints [54,55,62], or have analyzed the role of cell mass during cell cycle progression [55,58,59]. While most of the previous models were built with the use of ordinary differential equations [48,49,53,56,58], there are also examples of hybrid [52] and discrete models [60,61,65,66].

Despite the abundance of models developed for VPC fate determination and cell cycle dynamics in other organisms and the *C. elegans* embryo, the effect of coordination of the cell cycle and cell differentiation during vulval development has not been fully explored. Hereby we present the first model to include the molecular mechanism involved in the control of the postembryonic cell cycle of *C. elegans*. Dynamical models are important to understand how the molecular components involved in these cellular processes are integrated to coordinate the differentiation and proliferation during VPC fate specification. Such an integrative model is the focus of this paper. Our main findings are that the regulation of Notch signaling by the cell cycle preserves the potential of the VPCs and the three vulval fates to differentiate and de-differentiate, and that sequential control does not eliminate the sensitivity of the VPCs to inductive or lateral signaling during VPC fate determination.

## Results and discussion

### The regulatory network

**Figure 2:**
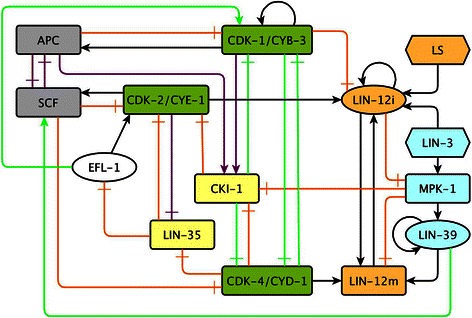
**The network of molecules involved in the control of VPC fate determination and the cell cycle in** ***C. elegans*** **.** Pointed arrows are positive regulatory interactions, and blunt arrows are negative regulatory interactions. Purple arrows are interactions reported in other organisms, and green arrows are predictions of our model. Orange nodes are part of the Notch pathway, and blue nodes are part of the Ras/MAPK pathway or its targets (LIN-39). Green nodes are CDK/Cyclin complexes, yellow nodes are CDK inhibitors, EFL-1, a transcription factor in white, and protein degradation complexes in gray. The external signals are represented as elongated hexagons, the known transcription factors as ellipses, and other proteins as rounded rectangles.

**Figure 3:**
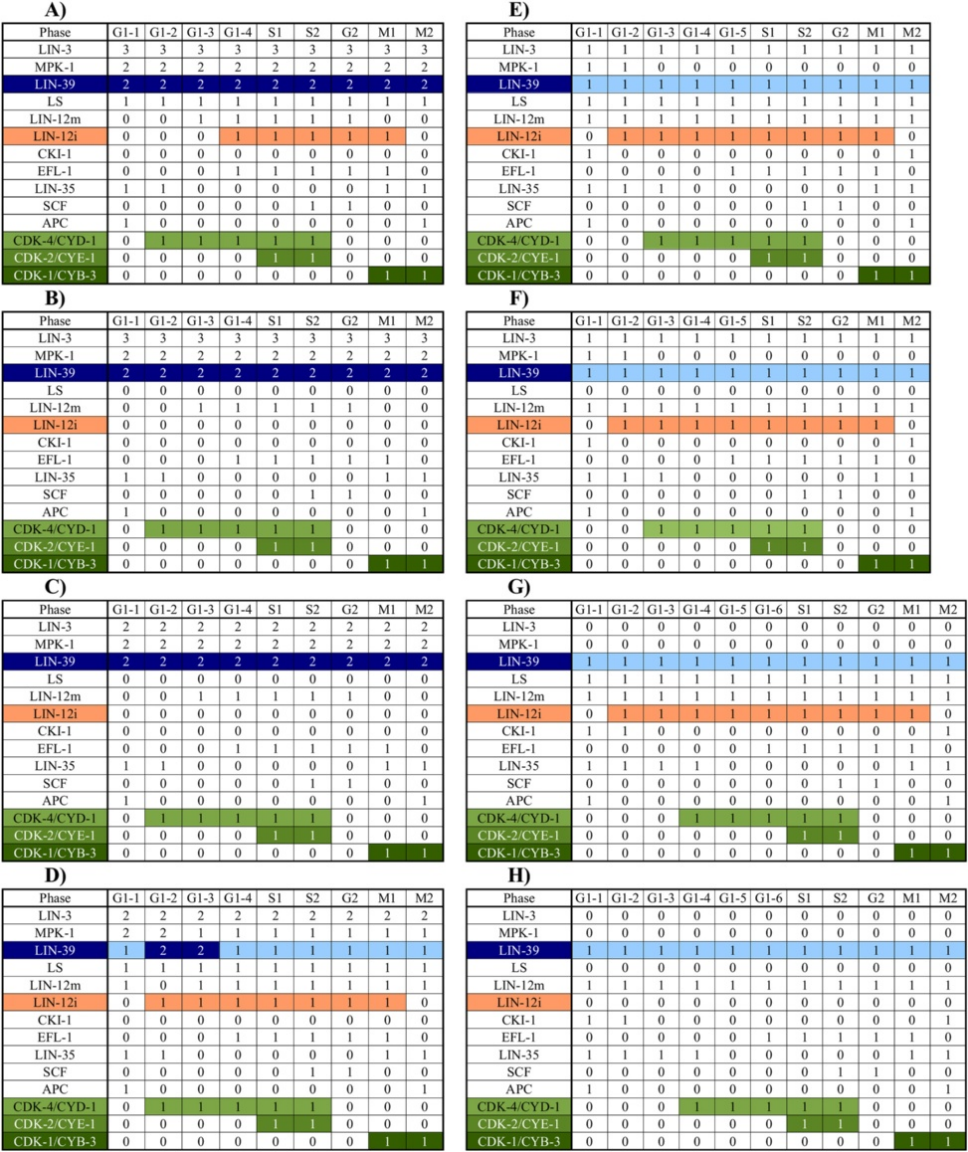
**The attractors of our model.** There is a total of eight cyclic attractors, with time running from left to right. Activity of LIN-12i, in orange, is a marker of the secondary fate (Attractors **D**, **E**, **F** and **G**). A high level of activity (2 in our model) of LIN-39, in dark blue, is a marker for the primary fate (Attractors **A**, **B**, and **C**). The tertiary fate is represented exclusively by attractor **H**, which is characterized by a low level of activity of LIN-39, and no LIN-12i, LIN-3, or lateral signal (LS) activity (This pattern of expression is observed in the VPCs during L2). CDK-4/CYD-1, shown in pale green, is activated before the S phase. CDK-2/CYE-1, shown in green, is a marker of the S phase. CDK-1/CYB-3, in dark green, is a marker for the M phase.

**Figure 4:**
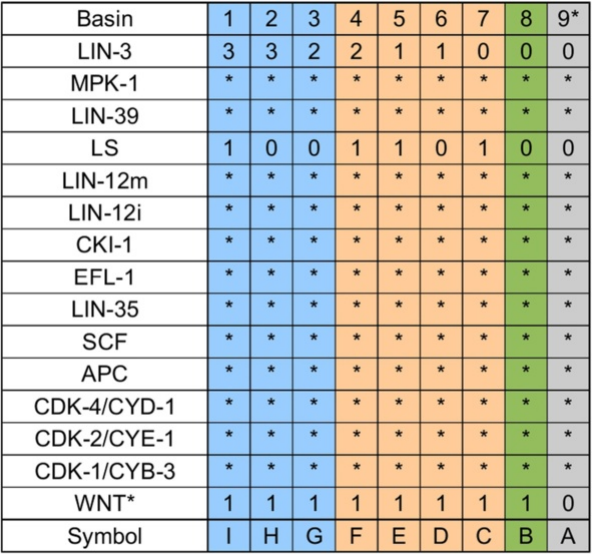
**Summary of the basins of attraction.** The stars in each basin represent all possible activity levels for the molecule. The primary fate basins are colored in blue, the secondary fate basins in orange, the tertiary fate basin in green and the fusion fate basin in gray. Loss of WNT activity was simulated by changing the basal state of LIN-39 from 1 to 0.

The regulatory network consists of 14 nodes and 37 regulatory interactions (Figure 2). The network incorporates regulatory interactions experimentally substantiated in *C. elegans*, five interactions documented in other organisms, and six interactions that constitute novel predictions from the present study. All such predictions, with the exception of the inhibition of CDK-1/CYB-3 by CKI-1, are necessary to recover the observed attractors. The exhaustive analysis of the dynamical behavior of the network as a discrete dynamical system revealed the existence of eight periodic attractors that cycle through the same stages of the cell cycle (Figure 3), partitioning evenly the state space (Figure 4).

The eight attractors can be interpreted as the patterns of molecular activation of the three vulval fates that cycle through the cell cycle (Figures 3 and 4). Attractors A, B, and C represent the primary fate, which is characterized by a high level (2 in our model) of LIN-39 and MPK-1 activity. Attractors D, E, F and G correspond to the secondary vulval fate which is characterized by LIN-12i activity. Notably, in all these attractors, LIN-12i is inactive during the first and last states of the cycle, due to the inhibitory effect of CDK-1/CYB-3. Finally, the tertiary fate is represented exclusively by attractor H, which is characterized by a low level of activity of LIN-39, and no LIN-12i, LIN-3, or lateral signal (LS) activity. This pattern of expression is observed in the VPCs during L2.

### Dynamics of the cell cycle module

Figure 3 shows the dynamical behavior of the regulatory network. The model recovers eight cyclic attractors, corresponding to the observed patterns of expression in actual cells. For example, Figure 3H shows the cyclic attractor that corresponds to tertiary fate cells and VPCs. At the beginning (G1-1), the inhibitors of the cell cycle, CKI-1, LIN-35, and the APC complex, are active. Then, APC is turned off, which leads to CKI-1 inactivation (G1-3), and thus allowing the CDK-4/CYD-1 complex to become active (G1-4), which is a marker for G1 progression. Next, the LIN-35 activity is inhibited (G1-5), leading to EFL-1 activation (G1-6) first, and then the CDK-2/CYE-1 complex, a marker of the S phase (S1). Later, the SCF turns on (S2), leading to the G2 phase where only EFL-1 and SCF are active (G2). Further on, LIN-35, and the CDK-1/CYB-3 complex, which is a marker for the M phase, are activated (M1). Leading to the EFL-1 inhibition and APC activation (M2). Finally, the activity of CDK-1/CYB-3 is inhibited, leading back to the beginning of G1 (G1-1).

Figures 3E, 3F and 3G show attractors that describe the behavior of most secondary fate cells, the cell cycle in secondary fate cells is one step shorter because the G1 phase lasts for only 5 time steps. Figure 3D, which describes the cyclic behavior of a secondary fate cell in a microenvironment with a high level of LIN-3 (2 in our model), as well as Figures 3A-C that describe the cyclic behavior of primary fate cells, share the same cyclic behavior as secondary and tertiary fate cells, except for the fact that their G1 phase lasts for only 4 steps.

**Figure 5:**
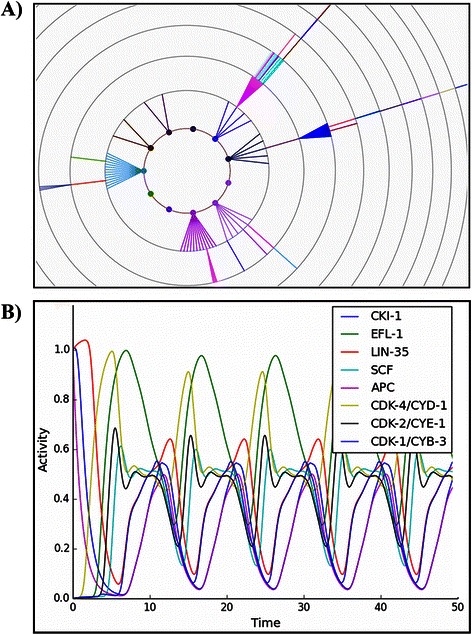
**Dynamics of the cell cycle.** **A)** State space of our Boolean module. Color-coded transition diagram of the cell cycle: the red component represents the activity of CKI-1, EFL-1 and LIN-35, the green component represents the activity of SCF and APC, and the blue component represents the activity of CDK-4/CYD-1, CDK-2/CYE-1 and CDK-1/CYB-3. **B)** Dynamics of our continuous model of the cell cycle. The initial state for the numerical integration starts at the first stage of G1.

By modeling the cell cycle module on its own, it is possible to observe that the cyclic behavior described above covers its entire state space (Figure 5A). Observe that the cyclic behavior of this module is maintained when it is modeled as a continuous dynamic system (Figure 5B), thus reducing the possibility of the cyclic behavior being an artifact of the modeling framework.

### The differentiation process

**Figure 6:**
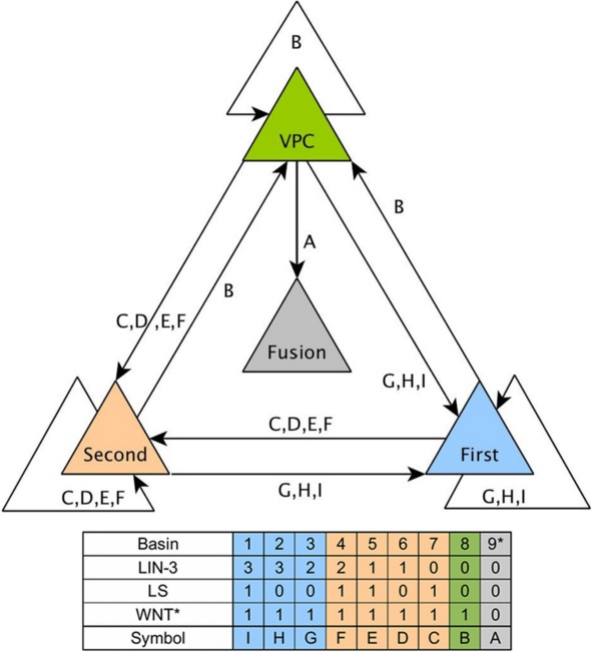
**The process of the cell differentiation.** Colored triangles represent the different cellular fates. The different signals that are able to move the model from one fate to another are specified on the table at the bottom of the figure, and the primary fate basins are colored in blue, the secondary fate basins in orange, the tertiary fate basin in green and the fusion fate basin in gray.

**Figure 7:**
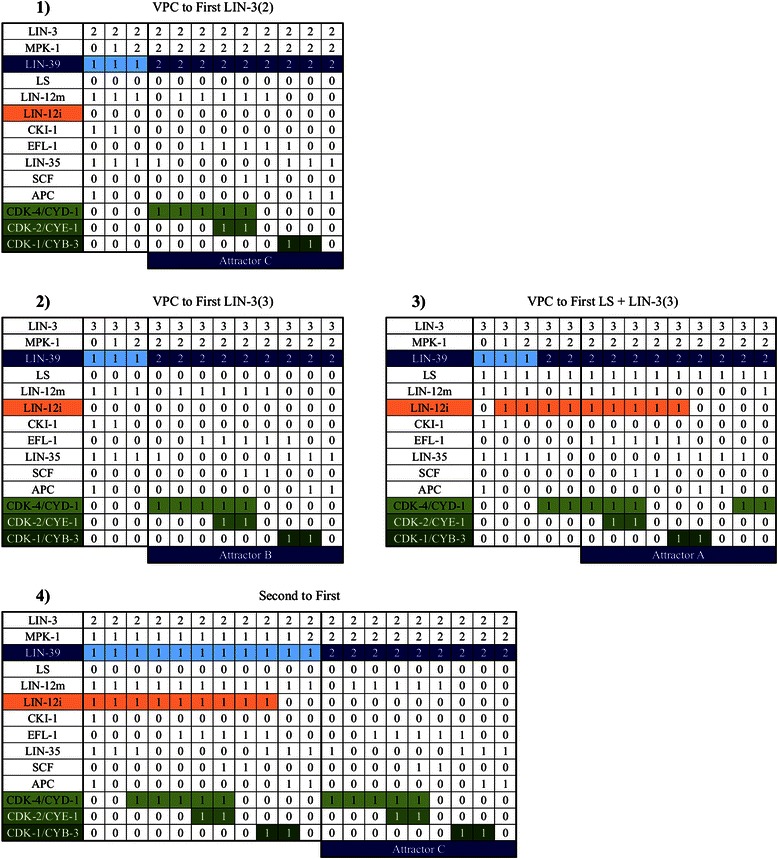
**Primary fate determination.** **1)** A VPC differentiating into a primary fate cell in an extracellular microenvironment with a moderately high concentration of LIN-3 (2 in our model), **2)** A VPC differentiating into a primary fate cell in an extracellular microenvironment with a high concentration of LIN-3 (3 in our model), **3)** A VPC differentiating into a primary fate cell in an extracellular microenvironment with lateral signal and a high concentration of LIN-3 (3 in our model), **4)** A secondary fate cell transdifferentiating into a primary fate cell in an extracellular microenvironment with a high concentration of LIN-3 (3 in our model).

**Figure 8:**
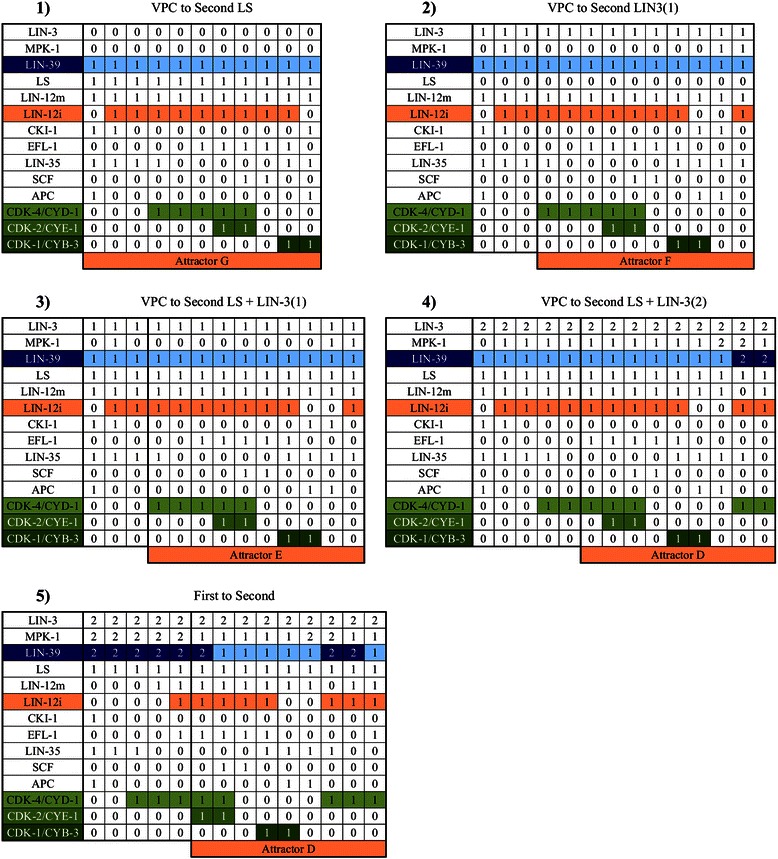
**Secondary fate determination.** **1)** A VPC differentiating into a secondary fate cell in an extracellular microenvironment with lateral signal, **2)** A VPC differentiating into a secondary fate cell in an extracellular microenvironment with a low concentration of LIN-3 (1 in our model), **3)** A VPC differentiating into a secondary fate cell in an extracellular microenvironment with lateral signal and a low concentration of LIN-3, **4)** A VPC differentiating into a secondary fate cell in an extracellular microenvironment with lateral signal and a moderately high (2 in our model) concentration of LIN-3, **5)** A primary fate cell transdifferentiating into a secondary fate cell in an extracellular microenvironment with a moderately high concentration of LIN-3 (2 in our model) and lateral signal.

**Figure 9:**
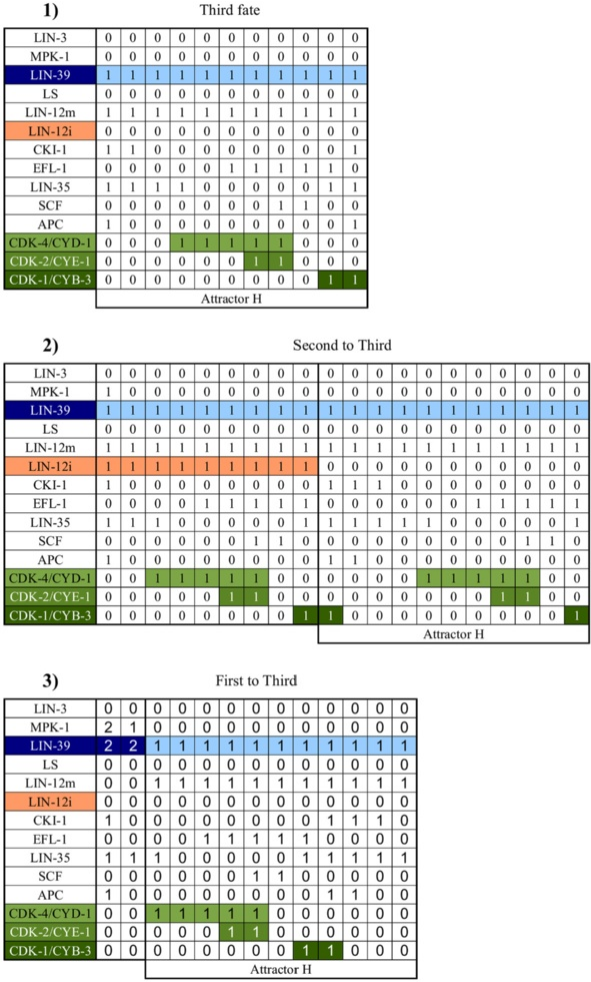
**Tertiary fate determination.** **1)** The tertiary fate is stable in an extracellular microenvironment without LS or LIN-3, **2)** A secondary fate cell dedifferentiating into a third fate cell in an extracellular microenvironment without LS or LIN-3, **3)** A primary fate cell dedifferentiating into a third fate cell in an extracellular microenvironment without LS or LIN-3.

To study the process of fate determination in our model, we followed the dynamics of the system starting from the initial patterns of expression that represent the different cell fates under different extracellular microenvironments (Figures 4 and 6). First, we followed the determination of the primary fate, which occurs when the concentration of LIN-3 is very high (3 in our model), with (Figure 7-3) or without (Figure 7-2) an active LS, or when the concentration of LIN-3 is moderately high (2 in our model) and the concentration of LS is lower than the threshold (0 in our model) (Figure 7-1). We also followed the network with an initial state representing a secondary fate cell under a microenvironment which induces a VPC to acquire the primary fate. In this case the network reaches a cyclic pattern of molecular activation that corresponds to the primary fate (Figure 7-4). Then, we followed the determination of the secondary fate, which may occur when a VPC or a primary fate cell is in the following microenvironments: LS and no LIN-3 activity (0 in our model)(Figure 8-1), low LIN-3 (1 in our model) with no LS (Figure 8-2), LS and low LIN-3 (Figure 8-3), or LS and medium high LIN-3 (Figure 8-4 and 8-5). Last, we followed the determination of the tertiary fate which may occur when a VPC/Tertiary-fate cell (Figure 9-1), a secondary fate cell (Figure 9-2) or a primary fate cell(Figure 9-3), is in a microenvironment with no LS and no LIN-3 (0 in our model).

Our model shows that the cell fates remain stable if the extracellular microenvironment remains stable, but the cells keep the potential to acquire any other fate if the microenvironment changes. Specifically, a primary or secondary fate cell has the potential to de-differentiate in a microenvironment without both LIN-3 and LS, a primary fate cell may trans-differentiate into a secondary fate cell in a microenvironment with low LIN-3 or LS and a secondary fate cell may also trans-differentiate into a primary fate cell in a microenvironment with moderately high LIN-3 and no LS or very high LIN-3 (Figures 4 and 6). Both de-differentiation and trans-differentiation have been observed experimentally [67].

Finally, it is important to note that the influence of the differentiation process over the cell cycle module has an effect on the length of the periodic behavior (Figure 3). Specifically, G1 lasts 4 time steps for the primary fate, 4 to 6 time steps for the secondary fate, and 6 time steps for the tertiary fate. This behavior arises because the Ras signaling shortens the duration of the cell cycle by inhibiting CKI-1. Specifically, when Ras signaling is moderate (Figure 3E-F), the cell cycle lasts ten time steps. But when the level of Ras signaling is high (Figure 3A-D), CKI-1 is not activated and thus the cell cycle lasts 9 time steps. The duration of the cell cycle may determine the number of times the VPCs divide, because the period of time when the VPCs may divide is limited.

### Simulation of mutants

One way to validate the type of regulatory network model presented here, is to test if altered expression states of the model components lead to altered attractors that mimic the observed patterns of expression and/or phenotypes described for loss and gain of function mutants. We simulated the effect of all 32 possible single loss- and gain-of-function mutations by setting the expression level of the corresponding node to zero, one, two, or three. We obtained the attractors of all these mutant models and compared them against the available experimental data (Additional file 1: Table S1, Additional file 2). Notably, 16 of the 18 phenotypes reported in the literature (88.9%) are recovered by these simulations. The simulated phenotypes caused by 24 mutations can be considered novel predictions of our model; of those 24 simulated mutations the effect of 14 has not been reported at all in the literature, and the other 10 cause additional effects in specific extracellular microenvironments that have not been observed experimentally. Specifically, our model predicts that *a*) a constitutively active lateral signal will prevent the determination of the tertiary fate, *b*) *mpk-1(1)* will cause the loss of the primary fate, *c*) *mpk-1(2)* will cause the loss of the secondary fate, *d*) five mutations will cause the VPCs to exit the cell cycle, *e*) two mutations will cause endoreplication, *f*) 15 mutations will allow the determination of the secondary fate in an extracellular microenvironment with a medium level of LIN-3 and no LS, *g*) nine mutations will allow the determination of the primary fate in an extracellular microenvironment with a medium level of LIN-3 and LS, *h*) 15 mutations will allow the determination of the secondary fate in an extracellular microenvironment with no LIN-3 and no LS, *i*) two mutations allow the determination of the tertiary fate in an extracellular microenvironment with LS and no LIN-3, *j*) according to our model, *lin-39(2)* represents a high concentration of constitutively phosphorylated LIN-39 and the result of its simulation is a Muv phenotype where all VPCs acquire the primary fate. Experimentally, the expression of heat shock-inducible *lin-39* after the ablation of the anchor cell during L2 was not enough to allow the VPCs to divide, and a high level of LIN-39 protein does not cause a Muv phenotype. But that might be because LIN-39 needs to be phosphorylated by MPK-1 in order to be activated [68], in order to prove or refute our prediction, the same experiment would need to be repeated, but with a constitutively phosphorylated LIN-39, *k*) according to our model, *lin-12i(0)* represents the loss of the transcription factor function of *lin-12* locally in the VPCs that causes the loss of secondary fate VPCs, but not necessarily the Muv phenotype reported in the literature where *lin-12(lf)* causes two anchor cells to form, causing a Muv and Egl phenotype where most VPCs acquire the primary fate [35], in order to verify this prediction the extra AC needs to be ablated in a *lin-12(lf)* and *lip-1::GFP* background to verify that the secondary fate is lost but the VPCs may still acquire the primary and tertiary fates, *l*) According to our model, *lin-12m(0)* represents the loss of LIN-12 protein in the membrane that produces a wild type phenotype, because we assume that Notch may still be activated internally by weak Ras signaling [27]. We remark that two of our simulated mutants, namely *efl-1(0), and SCF(0)*, do not have a clear correspondence with the experimental results.

The loss of *efl-1* function has been reported to cause a phenotype that resembles the phenotype caused by *lin-35(lf)*, suggesting that it is a G1/S inhibitor. Conversely, its co-factor *dpl-1* has been reported to both activate and inhibit the G1/S transition [69]. However, according to our model, EFL-1/DPL-1 functions as a transcriptional activator, as reported for the yeast and mammalian cell cycles [70], while *efl-1(0)* causes the cell cycle to stop between G1 and S.

Finally, according to our model, *SCF(0)* causes the cell cycle to stop at the S phase. However, the function of SCF is necessary for the cell cycle exit. *cul-1* is one of the main components of SCF, and *cul-1(lf)* causes very strong hyperplasia in the vulva, and more than 80 vulval cells are formed in those mutants [71]. SCF complexes have many diverse functions and only a few are well characterized. Specifically, SCF is necessary for CDC-25.1 degradation, which may be necessary for cell cycle quiescence [72]. Furthermore, negative regulation of CDK/Cyclin complexes is an important component even in minimal cell cycle oscillators [63]. Due to the simplified nature of our model, the function of SCF as a G1/S CDK/Cyclin regulator is crucial for cell cycle progression, but in the real system other complexes such as APC may act redundantly with SCF.

### Removal of regulatory interactions

We systematically eliminated all of the 38 regulatory interactions, one at the time, and evaluated the effect on the attractors attained by the model (Additional file 3: Table S2, Additional file 4). Notice that the removal of 12 interactions had no effect on the dynamics of the network, thus showing the structural robustness of the model.

There is a discrepancy between our model and the reported experimental results regarding the removal of the activation of LIN-12i by CDK-2/CYE-1. In the model, the elimination of this interaction has no effect on the dynamics of the network. Experimentally, however, the elimination of the aforementioned interaction causes a diminished concentration of LIN-12i in secondary fate cells [42]. Given the Boolean nature of the variable representing LIN-12i, the model cannot represent a partial reduction on its activation. Future versions on our model will, by necessity, incorporate more levels of activation to describe LIN-12i.

We predict the effect of removing 28 interactions that are not reported in the literature. Specifically, our model predicts that *a*) removing one of eight interactions would not affect the behavior of the system, *b*) removing the ability of MPK-1 to phosphorylate LIN-39 will cause the loss of the primary fate, *c*) removing one of four interactions would inhibit VPC divisions, *d*) removing one of two interactions, will lead to an endoreplication cell cycle, *e*) removing one of four interactions would cause a longer cell cycle, *f*) removing one of eight interactions causes a shorter cell cycle, *g*) removing one of seven interactions will allow the determination of the secondary fate in an extracellular microenvironment with no LIN-3 and no LS, *h*) removing one of ten interactions will allow the determination of the secondary fate in an extracellular microenvironment with a medium level of LIN-3 and no LS, and *j*) removing one of four interactions will allow the determination of the primary fate in an extracellular microenvironment with a medium level of LIN-3 and LS.

We then searched for the interactions that are necessary for the existence of the cyclic attractor. The update rules of our model of the cell cycle module (Equations 7-14) are not ambiguous. Without allowing ambiguity in the update rules, we found with *Griffin* ^a^ [73] 120 functional network topologies of the cell cycle module that allow the existence of the cyclic attractor. All such topologies include the following 14 interactions: *a*) the inhibition of EFL-1 by LIN-35, *b*) the activation of APC by CDK-1/CYB-3, *c*) the inhibition of LIN-35 by CDK-4/CYD-1, *d*) the activation of SCF by CDK-2/CYE-1, *e*) the inhibition of CDK-2/CYE-1 by SCF, *f*) the inhibition of CDK-4/CYD-1 by SCF, *g*) the inhibition of CDK-2/CYE-1 by LIN-35, *h*) the activation of CDK-1/CYB-3 by EFL-1, *i*) the inhibition of CDK-4/CYD-1 by CKI-1, *j*) the activation of CKI-1 by APC, *k*) the activation of CKI-1 by CDK-1/CYB-3, *l*) the inhibition of CDK-1/CYB-3 by CDK-4/CYD-1, *m*) the inhibition of CDK-4/CYD-1 by CDK-1/CYB-3, and *n*) the activation of CDK-2/CYE-1 by EFL-1.

We performed an additional test to check which interaction signs may be ambiguous by including all the interactions of our original model. Allowing ambiguity resulted in 2740 functional network topologies. However, in order for the cyclic attractor to exist, the following eight interaction signs must *not* be ambiguous: *a*) the inhibition of EFL-1 by LIN-35, *b*) the inhibition of CKI-1 by CDK-4/CYD-1, *c*) the inhibition of APC by SCF, *d*) the inhibition of SCF by APC, *e*) the activation of SCF by CDK-2/CYE-1, *f*) the activation of APC by CDK-1/CYB-3, *g*) the inhibition of LIN-35 by CDK-4/CYD-1, and *h*) the inhibition of LIN-35 by CDK-2/CYE-1.

### Circuits

Circuits or feedback loops are circular chains of interactions. The present model contains 60 positive feedback loops, and 51 negative feedback loops (Tables 1 and 2). The work of Thomas and collaborators has demonstrated the central role of feedback loops in the determination of the dynamic behavior of a regulatory network [74]. Specifically, functional positive feedback loops are necessary for the existence of multistationarity, while negative feedback loops are necessary to obtain oscillations [75].

**Table 1:** **Positive feedback loops in the network of Figure** 2

1	CDK-1/CYB-3 →
2	LIN-12i →
3	LIN-39 →
4	CDK-4/CYD-1 ⊣ CKI-1 ⊣
5	CDK-2/CYE-1 ⊣ LIN-35 ⊣
6	LIN-12i → LIN-12m →
7	CDK-4/CYD-1 ⊣ CDK-1/CYB-3 ⊣
8	SCF ⊣ APC ⊣
9	CDK-2/CYE-1 ⊣ LIN-35 ⊣ EFL-1 →
10	LIN-12i ⊣ MPK-1 ⊣ LIN-12m →
11	CDK-4/CYD-1 ⊣ CDK-1/CYB-3 → CKI-1 ⊣
12	CDK-4/CYD-1 ⊣ CDK-1/CYB-3 → APC → CKI-1 ⊣
13	SCF ⊣ APC → CKI-1 ⊣ CDK-2/CYE-1 →
14	CKI-1 ⊣ CDK-2/CYE-1 → LIN-12i ⊣ MPK-1 ⊣
15	CKI-1 ⊣ CDK-1/CYB-3 ⊣ LIN-12i ⊣ MPK-1 ⊣
16	SCF ⊣ CDK-4/CYD-1 ⊣ CKI-1 ⊣ CDK-1/CYB-3 → APC ⊣
17	SCF ⊣ CDK-2/CYE-1 → LIN-12i ⊣ MPK-1 → LIN-39 →
18	SCF ⊣ CDK-4/CYD-1 ⊣ LIN-35 ⊣ EFL-1 → CDK-1/CYB-3 → APC ⊣
19	SCF ⊣ CDK-4/CYD-1 → LIN-12m → LIN-12i ⊣ MPK-1 → LIN-39 →
20	SCF ⊣ CDK-4/CYD-1 ⊣ CDK-1/CYB-3 ⊣ LIN-12i ⊣ MPK-1 →
	LIN-39 →
21	SCF ⊣ APC → CKI-1 ⊣ CDK-4/CYD-1 ⊣ LIN-35 ⊣ CDK-2/CYE-1 →
22	SCF ⊣ APC ⊣ CDK-1/CYB-3 ⊣ LIN-12i ⊣ MPK-1 → LIN-39 →
23	SCF ⊣ CDK-2/CYE-1 ⊣ LIN-35 ⊣ EFL-1 → CDK-1/CYB-3 → APC ⊣
24	CKI-1 ⊣ CDK-1/CYB-3 ⊣ CDK-4/CYD-1 → LIN-12m → LIN-12i ⊣
	MPK-1 ⊣
25	CKI-1 ⊣ CDK-4/CYD-1 ⊣ LIN-35 → CDK-2/CYE-1 → LIN-12i ⊣
	MPK-1 ⊣
26	SCF ⊣ CDK-4/CYD-1 ⊣ CDK-1/CYB-3 ⊣ LIN-12i ⊣ MPK-1 ⊣ CKI-1 ⊣
	CDK-2/CYE-1 →
27	SCF ⊣ CDK-4/CYD-1 ⊣ CKI-1 ⊣ CDK-2/CYE-1 → LIN-12i ⊣
	MPK-1 → LIN-39 →
28	SCF ⊣ CDK-4/CYD-1 ⊣ LIN-35 ⊣ CDK-2/CYE-1 → LIN-12i ⊣
	MPK-1 → LIN-39 →
29	SCF ⊣ CDK-4/CYD-1 ⊣ LIN-35 ⊣ EFL-1 → CDK-1/CYB-3 → CKI-1 ⊣
	CDK-2/CYE-1 →
30	SCF ⊣ APC → CKI-1 ⊣ CDK-4/CYD-1 ⊣ LIN-35 ⊣ EFL-1 →
	CDK-2/CYE-1 →
31	SCF ⊣ APC → CKI-1 ⊣ CDK-1/CYB-3 ⊣ LIN-12i ⊣ MPK-1 →
	LIN-39 →
32	SCF ⊣ CDK-4/CYD-1 → LIN-12m → LIN-12i ⊣ MPK-1 ⊣ CKI-1 ⊣
	CDK-2/CYE-1 →
33	SCF ⊣ APC ⊣ CDK-1/CYB-3 ⊣ LIN-12i ⊣ MPK-1 ⊣ CKI-1 ⊣
	CDK-2/CYE-1 →
34	CKI-1 ⊣ CDK-2/CYE-1 ⊣ LIN-35 ⊣ EFL-1 → CDK-1/CYB-3 ⊣
	LIN-12i ⊣ MPK-1 ⊣
35	CKI-1 ⊣ CDK-4/CYD-1 ⊣ LIN-35 ⊣ EFL-1 → CDK-1/CYB-3 ⊣
	LIN-12i ⊣ MPK-1 ⊣
36	SCF ⊣ CDK-4/CYD-1 ⊣ CKI-1 ⊣ CDK-2/CYE-1 ⊣ LIN-35 ⊣ EFL-1 →
	CDK-1/CYB-3 → APC ⊣
37	SCF ⊣ CDK-4/CYD-1 ⊣ LIN-35 ⊣ EFL-1 → CDK-2/CYE-1 →
	LIN-12i ⊣ MPK-1 → LIN-39 →
38	SCF ⊣ CDK-4/CYD-1 ⊣ LIN-35 ⊣ EFL-1 → CDK-1/CYB-3 → APC →
	CKI-1 ⊣ CDK-2/CYE-1 →
39	SCF ⊣ CDK-4/CYD-1 ⊣ CDK-1/CYB-3 → CKI-1 ⊣ CDK-2/CYE-1 →
	LIN-12i ⊣ MPK-1 → LIN-39 →
40	SCF ⊣ APC ⊣ CDK-1/CYB-3 ⊣ CDK-4/CYD-1 → LIN-12m →
	LIN-12i ⊣ MPK-1 → LIN-39 →
41	SCF ⊣ APC ⊣ CDK-1/CYB-3 → CKI-1 ⊣ CDK-2/CYE-1 → LIN-12i ⊣
	MPK-1 → LIN-39 →
42	CKI-1 ⊣ CDK-1/CYB-3 ⊣ CDK-4/CYD-1 ⊣ LIN-35 ⊣ EFL-1 →
	CDK-2/CYE-1 → LIN-12i ⊣ MPK-1 ⊣
43	SCF ⊣ CDK-2/CYE-1 → LIN-12i ⊣ MPK-1 ⊣ CKI-1 ⊣ CDK-4/CYD-1 ⊣
	CDK-1/CYB-3 → APC ⊣
44	SCF ⊣ CDK-4/CYD-1 ⊣ CDK-1/CYB-3 → APC → CKI-1 ⊣
	CDK-2/CYE-1 → LIN-12i ⊣ MPK-1 → LIN-39 →
45	CKI-1 ⊣ CDK-2/CYE-1 ⊣ LIN-35 ⊣ EFL-1 → CDK-1/CYB-3 ⊣
	CDK-4/CYD-1 → LIN-12m → LIN-12i ⊣ MPK-1 ⊣
46	SCF ⊣ APC → CKI-1 ⊣ CDK-1/CYB-3 ⊣ CDK-4/CYD-1 → LIN-12m →
	LIN-12i ⊣ MPK-1 → LIN-39 →
47	SCF ⊣ APC ⊣ CDK-1/CYB-3 ⊣ CDK-4/CYD-1 → LIN-12m → LIN-12i
	⊣ MPK-1 ⊣ CKI-1 ⊣ CDK-2/CYE-1 →
48	SCF ⊣ CDK-4/CYD-1 ⊣ LIN-35 → CDK-2/CYE-1 → LIN-12i ⊣ MPK-1
	⊣ CKI-1 ⊣ CDK-1/CYB-3 → APC ⊣
49	SCF ⊣ APC ⊣ CDK-1/CYB-3 ⊣ CDK-4/CYD-1 ⊣ CKI-1 ⊣ CDK-2/CYE-1
	→ LIN-12i ⊣ MPK-1 → LIN-39 →
50	SCF ⊣ APC ⊣ CDK-1/CYB-3 ⊣ CDK-4/CYD-1 ⊣ LIN-35 ⊣ CDK-2/CYE-1
	→ LIN-12i ⊣ MPK-1 → LIN-39 →
51	SCF ⊣ APC ⊣ CDK-1/CYB-3 → CKI-1 ⊣ CDK-4/CYD-1 → LIN-12m →
	LIN-12i ⊣ MPK-1 → LIN-39 →
52	SCF ⊣ APC ⊣ CDK-1/CYB-3 ⊣ LIN-12i ⊣ MPK-1 ⊣ CKI-1 ⊣
	CDK-4/CYD-1 ⊣ LIN-35 ⊣ EFL-1 → CDK-2/CYE-1 →
53	SCF ⊣ APC → CKI-1 ⊣ CDK-4/CYD-1 ⊣ LIN-35 ⊣ EFL-1 →
	CDK-1/CYB-3 ⊣ LIN-12i ⊣ MPK-1 → LIN-39 →
54	SCF ⊣ APC → CKI-1 ⊣ CDK-2/CYE-1 ⊣ LIN-35 ⊣ EFL-1 →
	CDK-1/CYB-3 ⊣ LIN-12i ⊣ MPK-1 → LIN-39 →
55	SCF ⊣ APC → CKI-1 ⊣ CDK-1/CYB-3 ⊣ CDK-4/CYD-1 ⊣ LIN-35 ⊣
	CDK-2/CYE-1 → LIN-12i ⊣ MPK-1 → LIN-39 →
56	SCF ⊣ APC ⊣ CDK-1/CYB-3 ⊣ CDK-4/CYD-1 ⊣ LIN-35 ⊣ EFL-1 →
	CDK-2/CYE-1 → LIN-12i ⊣ MPK-1 → LIN-39 →
57	SCF ⊣ APC ⊣ CDK-1/CYB-3 → CKI-1 ⊣ CDK-4/CYD-1 ⊣ LIN-35 ⊣
	CDK-2/CYE-1 → LIN-12i ⊣ MPK-1 → LIN-39 →
58	SCF ⊣ APC → CKI-1 ⊣ CDK-1/CYB-3 ⊣ CDK-4/CYD-1 ⊣ LIN-35 ⊣
	EFL-1 → CDK-2/CYE-1 → LIN-12i ⊣ MPK-1 → LIN-39 →
59	SCF ⊣ APC ⊣ CDK-1/CYB-3 → CKI-1 ⊣ CDK-4/CYD-1 ⊣ LIN-35 ⊣
	EFL-1 → CDK-2/CYE-1 → LIN-12i ⊣ MPK-1 → LIN-39 →
60	SCF ⊣ APC → CKI-1 ⊣ CDK-2/CYE-1 ⊣ LIN-35 ⊣ EFL-1 →
	CDK-1/CYB-3 ⊣ CDK-4/CYD-1 → LIN-12m → LIN-12i ⊣ MPK-1 →
	LIN-39 →

**Table 2:** **Negative feedback loops in the network of Figure** 2

1	CDK-1/CYB-3 → APC ⊣
2	CKI-1 ⊣ CDK-1/CYB-3 →
3	SCF ⊣ CDK-2/CYE-1 →
4	CDK-4/CYD-1 ⊣ CKI-1 ⊣ CDK-1/CYB-3 ⊣
5	CKI-1 ⊣ CDK-1/CYB-3 → APC →
6	SCF ⊣ CDK-4/CYD-1 ⊣ CKI-1 ⊣ CDK-2/CYE-1 →
7	LIN-12i ⊣ MPK-1 → LIN-39 → LIN-12m →
8	SCF ⊣ CDK-4/CYD-1 ⊣ LIN-35 ⊣ CDK-2/CYE-1 →
9	CDK-4/CYD-1 ⊣ LIN-35 ⊣ EFL-1 → CDK-1/CYB-3 ⊣
10	SCF ⊣ CDK-4/CYD-1 ⊣ CDK-1/CYB-3 → APC ⊣
11	CKI-1 ⊣ CDK-2/CYE-1 ⊣ LIN-35 ⊣ EFL-1 → CDK-1/CYB-3 →
12	SCF ⊣ CDK-4/CYD-1 ⊣ LIN-35 ⊣ EFL-1 → CDK-2/CYE-1 →
13	CDK-4/CYD-1 ⊣ LIN-35 ⊣ EFL-1 → CDK-1/CYB-3 → CKI-1 ⊣
14	SCF ⊣ CDK-4/CYD-1 ⊣ CDK-1/CYB-3 → CKI-1 ⊣ CDK-2/CYE-1 →
15	SCF ⊣ APC ⊣ CDK-1/CYB-3 → CKI-1 ⊣ CDK-2/CYE-1 →
16	CKI-1 ⊣ CDK-4/CYD-1 → LIN-12m → LIN-12i ⊣ MPK-1 ⊣
17	CKI-1 ⊣ CDK-4/CYD-1 ⊣ CDK-1/CYB-3 ⊣ LIN-12i ⊣ MPK-1 ⊣
18	CDK-4/CYD-1 ⊣ CKI-1 ⊣ CDK-2/CYE-1 ⊣ LIN-35 ⊣ EFL-1 →
	CDK-1/CYB-3 ⊣
19	CKI-1 ⊣ CDK-2/CYE-1 ⊣ LIN-35 ⊣ EFL-1 → CDK-1/CYB-3 → APC →
20	CDK-4/CYD-1 ⊣ LIN-35 ⊣ EFL-1 → CDK-1/CYB-3 → APC → CKI-1 ⊣
21	SCF ⊣ CDK-4/CYD-1 ⊣ CDK-1/CYB-3 → APC → CKI-1 ⊣
	CDK-2/CYE-1 →
22	SCF ⊣ APC ⊣ CDK-1/CYB-3 ⊣ CDK-4/CYD-1 ⊣ CKI-1 ⊣
	CDK-2/CYE-1 →
23	SCF ⊣ APC ⊣ CDK-1/CYB-3 ⊣ CDK-4/CYD-1 ⊣ LIN-35 ⊣
	CDK-2/CYE-1 →
24	CKI-1 ⊣ CDK-1/CYB-3 ⊣ CDK-4/CYD-1 ⊣ LIN-35 → CDK-2/CYE-1 →
	LIN-12i ⊣ MPK-1 ⊣
25	CKI-1 ⊣ CDK-4/CYD-1 ⊣ LIN-35 ⊣ EFL-1 → CDK-2/CYE-1 →
	LIN-12i ⊣ MPK-1 ⊣
26	SCF ⊣ CDK-2/CYE-1 → LIN-12i ⊣ MPK-1 ⊣ CKI-1 ⊣ CDK-1/CYB-3 →
	APC ⊣
27	SCF ⊣ CDK-4/CYD-1 ⊣ CKI-1 ⊣ CDK-1/CYB-3 ⊣ LIN-12i ⊣ MPK-1 →
	LIN-39 →
28	SCF ⊣ APC → CKI-1 ⊣ CDK-2/CYE-1 → LIN-12i ⊣ MPK-1 →
	LIN-39 →
29	SCF ⊣ APC → CKI-1 ⊣ CDK-1/CYB-3 ⊣ CDK-4/CYD-1 ⊣ LIN-35 ⊣
	CDK-2/CYE-1 →
30	SCF ⊣ APC ⊣ CDK-1/CYB-3 ⊣ CDK-4/CYD-1 ⊣ LIN-35 ⊣ EFL-1 →
	CDK-2/CYE-1 →
31	SCF ⊣ APC ⊣ CDK-1/CYB-3 → CKI-1 ⊣ CDK-4/CYD-1 ⊣ LIN-35 ⊣
	CDK-2/CYE-1 →
32	SCF ⊣ CDK-4/CYD-1 ⊣ LIN-35 ⊣ EFL-1 → CDK-1/CYB-3 ⊣ LIN-12i ⊣
	MPK-1 → LIN-39 →
33	SCF ⊣ APC → CKI-1 ⊣ CDK-4/CYD-1 → LIN-12m → LIN-12i ⊣
	MPK-1 → LIN-39 →
34	SCF ⊣ APC → CKI-1 ⊣ CDK-4/CYD-1 ⊣ CDK-1/CYB-3 ⊣ LIN-12i ⊣
	MPK-1 → LIN-39 →
35	SCF ⊣ APC → CKI-1 ⊣ CDK-1/CYB-3 ⊣ CDK-4/CYD-1 ⊣ LIN-35 ⊣
	EFL-1 → CDK-2/CYE-1 →
36	SCF ⊣ APC ⊣ CDK-1/CYB-3 → CKI-1 ⊣ CDK-4/CYD-1 ⊣ LIN-35 ⊣
	EFL-1 → CDK-2/CYE-1 →
37	SCF ⊣ CDK-2/CYE-1 ⊣ LIN-35 ⊣ EFL-1 → CDK-1/CYB-3 ⊣ LIN-12i ⊣
	MPK-1 → LIN-39 →
38	SCF ⊣ CDK-4/CYD-1 → LIN-12m → LIN-12i ⊣ MPK-1 ⊣ CKI-1 ⊣
	CDK-1/CYB-3 → APC ⊣
39	SCF ⊣ APC → CKI-1 ⊣ CDK-4/CYD-1 ⊣ LIN-35 ⊣ CDK-2/CYE-1 →
	LIN-12i ⊣ MPK-1 → LIN-39 →
40	SCF ⊣ APC ⊣ CDK-1/CYB-3 ⊣ LIN-12i ⊣ MPK-1 ⊣ CKI-1 ⊣
	CDK-4/CYD-1 ⊣ LIN-35 → CDK-2/CYE-1 →
41	SCF ⊣ CDK-4/CYD-1 ⊣ LIN-35 ⊣ EFL-1 → CDK-1/CYB-3 ⊣ LIN-12i ⊣
	MPK-1 ⊣ CKI-1 ⊣ CDK-2/CYE-1 →
42	SCF ⊣ CDK-2/CYE-1 → LIN-12i ⊣ MPK-1 ⊣ CKI-1 ⊣ CDK-4/CYD-1 ⊣
	LIN-35 ⊣ EFL-1 → CDK-1/CYB-3 → APC ⊣
43	SCF ⊣ CDK-4/CYD-1 ⊣ CKI-1 ⊣ CDK-2/CYE-1 ⊣ LIN-35 ⊣ EFL-1 →
	CDK-1/CYB-3 ⊣ LIN-12i ⊣ MPK-1 → LIN-39 →
44	SCF ⊣ CDK-4/CYD-1 ⊣ LIN-35 ⊣ EFL-1 → CDK-2/CYE-1 → LIN-12i ⊣
	MPK-1 ⊣ CKI-1 ⊣ CDK-1/CYB-3 → APC ⊣
45	SCF ⊣ CDK-4/CYD-1 ⊣ LIN-35 ⊣ EFL-1 → CDK-1/CYB-3 → CKI-1 ⊣
	CDK-2/CYE-1 → LIN-12i ⊣ MPK-1 → LIN-39 →
46	SCF ⊣ APC → CKI-1 ⊣ CDK-4/CYD-1 ⊣ LIN-35 ⊣ EFL-1 →
	CDK-2/CYE-1 → LIN-12i ⊣ MPK-1 → LIN-39 →
47	SCF ⊣ CDK-2/CYE-1 ⊣ LIN-35 ⊣ EFL-1 → CDK-1/CYB-3 ⊣
	CDK-4/CYD-1 → LIN-12m → LIN-12i ⊣ MPK-1 → LIN-39 →
48	SCF ⊣ CDK-4/CYD-1 → LIN-12m → LIN-12i ⊣ MPK-1 ⊣ CKI-1 ⊣
	CDK-2/CYE-1 ⊣ LIN-35 ⊣ EFL-1 → CDK-1/CYB-3 → APC ⊣
49	SCF ⊣ CDK-4/CYD-1 ⊣ LIN-35 ⊣ EFL-1 → CDK-1/CYB-3 → APC →
	CKI-1 ⊣ CDK-2/CYE-1 → LIN-12i ⊣ MPK-1 → LIN-39 →
50	SCF ⊣ CDK-2/CYE-1 ⊣ LIN-35 ⊣ EFL-1 → CDK-1/CYB-3 → CKI-1 ⊣
	CDK-4/CYD-1 → LIN-12m → LIN-12i ⊣ MPK-1 → LIN-39 →
51	SCF ⊣ CDK-2/CYE-1 ⊣ LIN-35 ⊣ EFL-1 → CDK-1/CYB-3 → APC →
	CKI-1 ⊣ CDK-4/CYD-1 → LIN-12m → LIN-12i ⊣ MPK-1 → LIN-39 →

According to our model, there is large redundancy among circuits, and thus no single circuit can be considered essential for the determination vulval fates. By contrast, the specific combination of input signals determines the number of attractors attained by the system. Redundancy among feedback circuits is emerging as a generic trait of regulatory networks. Furthermore, feedback circuit redundancy could play an important role in network robustness to mutations and noise [76].

## Conclusions

Our model recovers the stable patterns of activation of the considered molecular components under wild type and mutant conditions, replicating those patterns encountered on actual cells during vulval development in *C. elegans*. To the best of our knowledge, the present model is the first that explores the dynamic effect of the mechanism for the cross-regulation between the cell cycle and the cell fate determination of vulval cells, mechanism that was proposed by [42] and [77]. Our model provides a suitable approach to understand the coordinated regulation of cell-cycle progression and differentiation during this process.

The cross-regulation between cell differentiation and cell-cycle progression in vulval cells of *C. elegans* is mediated by the activation of SCF by LIN-39, the inhibition of CKI-1 by MPK-1, the inhibition of LIN-12i by CDK-1/CYB-3, the activation of LIN-12i by CDK-2/CYE-1, and the activation of LIN-12m by CDK-4/CYD-1. The type of regulatory interactions that grant such dynamic coordination might be conserved in other biological systems [78,79], and may also constitute a useful framework to address such coordination in other systems.

Our modeling effort resulted in the following predictions for the system under study: *a*) the activation of SCF by LIN-39, removing this interaction causes cell cycle quiescence in our model. *b*) the activation of CDK-1/CYB-3 by EFL-1, removing this interaction causes an endoreplication-like cell cycle. *c*) the inhibition of CDK-4/CYD-1 by CKI-1, removing this interaction causes a short cell cycle. *d*) the inhibition of CDK-1/CYB-3 by CKI-1, removing this interaction does not change the dynamic behavior of our model, *e*) the inhibition of CDK-1/CYB-3 by CDK-4/CYD-1, removing this interaction causes a modified cell cycle. *f*) the inhibition of CDK-4/CYD-1 by CDK-1/CYB-3, removing this interaction causes a short cell cycle.

Given the importance of the Notch signaling pathway for different developmental processes, it is fundamental to understand how it interacts with other signaling pathways. Here, we highlight the temporal regulation of LIN-12 by different CDK/cyclin complexes, which leads to precise spatio-temporal regulation of Notch signaling during vulva development and opens a wide range of possibilities in the comprehension of how cell fates are established under a specific combination of intracellular and extracellular signals. Our model shows that regulation of Notch signaling by the cell cycle preserves the potential of the VPCs and the three fates to differentiate and de-differentiate, allowing them to remain completely responsive to the concentration of LIN-3 and LS in the extracellular micro-environment.

Another important contribution of our model is that the need for a sequential control of fate determination disappears completely. Without the cell cycle effect, in a microenvironment with a moderately high level of LIN-3 and LS, a VPC would acquire either the primary or the secondary fate, depending on which inductive signal affected the cell first. In our model, however, the VPC will always acquire the secondary fate. Dynamical analysis such as ours are needed to achieve an adequate understanding of molecular regulation during the development of multicellular organisms.

Certain mutations can dramatically affect the behavior of a regulatory network, even if they do not cause the loss of all the functions of a protein. Simulating the removal of certain interactions, we were able to propose some changes to specific regions of certain proteins that could lead to abnormal vulval development in *C. elegans*.

Despite the broad agreement between our model and the experimental data, there is ample room for improvement. Specifically, we will try to incorporate in future versions the molecular mechanism involved when the cell cycle is activated or inactivated by a combination of CKI-1 activation by LIN-29 during L4 and SCF mediated degradation of CDC-25.1, as well as the molecules involved in the transitions between mitosis, meiosis, endoreplication and the embryonic cell cycle.

## Methods

### Molecular basis of the regulatory network

We built a simplified model of the molecular network involved in the fate determination of the VPCs and the control of the cell cycle by connecting two functional modules, that is, sets of biological molecules that are involved in accomplishing a specific biological function in the cell. Specifically, we used one module for the network of molecules involved in cell cycle control and a second module for the network of molecules involved in the control of vulval fate determination. We built the two functional modules by including only the molecules with very penetrant mutant phenotypes reported in the literature. For the fate determination module, we included only the ligands and the effectors of the Ras and Notch signaling cascades, and MPK-1, necessary to represent the mutual inhibition between them. For the cell cycle module, we included only the three main CDK/Cyclin complexes and their main regulators. Most interactions in the model are supported by experimental evidence in *C. elegans*, as summarized below. These interactions can be classified as activations or inhibitions [80], defined as follows: Given two genes *i* and *j*, *i* *activates* *j* if there exists a configuration (*i.e.* a pattern of molecular activation) *x*=(*x*_1_,…,*x*_*n*_) and values *a* and *b*, with *a*>*b*, such that:

$$\begin{aligned} &f_{j}(x_{1},\ldots,x_{i-1},a,x_{i+1},\ldots,x_{n})\\&-f_{j}(x_{1},\ldots,x_{i-1},b,x_{i+1},\ldots,x_{n}) > 0 \end{aligned} $$

Conversely, *i* *inhibits* *j* if there exists a configuration *x*=(*x*_1_,…,*x*_*n*_) and values *a* and *b*, with *a*>*b*, such that:

$$\begin{aligned} &f_{j}(x_{1},\ldots,x_{i-1},a,x_{i+1},\ldots,x_{n})\\&-f_{j}(x_{1},\ldots,{x_{i-1}},b,x_{i+1},\ldots,x_{n}) < 0 \end{aligned} $$

According to this definition, it is possible for gene *i* to both activate and inhibit gene *j*. In this case, we say that the rule or interaction is *ambiguous*. The only ambiguous update rule in our model is in LIN-12i (Equation 6). The model also contains interactions reported in other organisms, namely, the mutual inhibition between APC and SCF in mammals, the activation of CKI-1 by APC in humans, the activation of CKI-1 by CDK-1/CYB-3 in yeast, and the inhibition of LIN-35 by CDK-2/CYE-1 in mammals. Finally, the model also includes interactions that are predictions of our model, namely, the mutual inhibition between CDK-1/CYB-3 and CDK-4/CYD-1, the activation of CDK-1/CYB-3 by EFL-1, the inhibition of CDK-4/CYD-1 and CDK-1/CYB-3 by CKI-1, and the activation of SCF by LIN-39.

#### Molecules involved in VPC fate determination:

The Ras/MAPK signaling cascade is represented by three nodes, LIN-3, MPK-1, and LIN-39. LIN-3 is an EGF ortholog, and functions as an external signal that is used as a parameter that does not change during each simulation. LIN-3 activates the Ras/MAPK signaling [16,29,81,82], whose main effector is MPK-1 [83,84], an ERK ortholog. MPK-1 phosphorylates many important transcription factors, such as LIN-39, LIN-1, LIN-31 and some components of the mediator complex like LIN-25 and SUR-2, that bind to the promoters of *lin-39*, activating its transcription. The LIN-39 product, is a HOM-C protein homologous to Deformed and Sex combs reduced. Importantly, phosphorylated LIN-39 activates its own expression [68,83-85].

To represent the Notch signaling cascade we included in the simplified model the lateral signal as well as the ortholog of Notch *lin-12*. The lateral signal (LS) functions as a parameter of the model, and it comprises DSL-1 and LAG-2 or APX-1. DSL-1 originates from P6.p and forms a gradient, while LAG-2 and APX-1 are membrane proteins that are also expressed by P6.p, whose effect is on the neighbor cells that are in direct physical contact, that is, P5.p and P7.p [86]. LIN-12 is represented by two nodes in our model. On the one hand, LIN-12i represents the fragment of LIN-12 that travels to the nucleus and forms part of the LIN-12intra/LAG-1/SEL-8 complex. This complex activates the transcription of the lateral signal targets, such as *lin-11*, *lip-1* and *lin-12* [87], and stabilizes the membrane localization of LIN-12 through *mir-61* and VAV-1 [88]. On the other hand, LIN-12m represents the protein localized in the membrane, functioning as a receptor for the lateral signal [89,90]. Finally, it is known that LIN-39 activates the transcription of *lin-12* [91].

We included the interactions that allow for a mutual inhibition of the Ras and Notch pathways. MPK-1 activates the mediator complex, increasing the rate at which LIN-12 is removed from the membrane and marked for degradation [92,93]. Now, LIP-1 inactivates MPK-1 [94], and ARK-1 inhibits LET-23 in a SEM-5-dependent mechanism [95]. Both LIP-1 and ARK-1 are lateral signal targets, and are activated by LIN-12i. Thus, there is a net negative effect from LIN-12i to MPK-1.

#### Molecules involved in the control of the cell cycle and their interactions:

In general, the molecular mechanism for the control of the cell cycle is based on the activity of a cyclin-dependent kinase (CDK), which is required to advance from one stage of the cell cycle to the next.

Each CDK binds to certain cyclins when they are available; specifically those that have a high enough binding affinity with the CDK. CDK-inhibitory proteins (CKIs) associate with Cyclin/CDK complexes to keep them inactive, and phosphorylation by Wee1/Myt1 kinases also inhibits their activity. Cyclin/CDK activation requires phosphorylation, ubiquitination and proteolysis of the CKI, phosphorylation of the CDK by a CDK-activating kinase (CAK), as well as the removal of the inhibitory phosphates by a Cdc25 phosphatase. Cyclin destruction leads to inactivation. Ubiquitination and proteolysis of cell cycle regulators in late G1 and S requires cullin-based E3 ligases such as Skp1-Cul1-F box (SCF), while in M phase and early G1 the activity of the anaphase-promoting complex (APC) –which is an E3 RING ubiquitin ligase– is needed [69].

In *C. elegans*, the regulatory function of three CDK/Cyclin complexes during the cell cycle is known: CDK-4/CYD-1 is the first complex involved in the control of the G1 to S transition, and the expression of *cdk-4* and *cyd-1* is sufficient to activate the expression the S phase marker rnr::GFP [96]. CYD-1 is likely to be degraded by SCF [97], and CDK-4/CYD-1 may be a target of CKI-1 inhibition because CKI-1 binds CYD-1 [98]. CDK-2/CYE-1 is the second complex involved in the control of the G1 to S transition, CDK-2 binds to CYE-1 and CKI-1 may inhibit CYE-1 function [69,98]. When CKI-1 is ubiquitinated, it dissociates from CDK-2/CYE-1, then CDK-2/CYE-1 activity allows the progression from G1 to S [69]. The promoter of *cye-1* contains potential EFL-1/DPL-1 binding sites [99]. CUL-1 (part of SCF) may inhibit CYE-1, and CDK-1/CYB-3 is involved in the G2 to M transition but not G1 to S [69]. CDK-1 binds to CYB-1 and CYB-3 *in vitro*, and APC-11 inhibits CYB-1 [100]. CDK-1/CYB-3 activates CDC-25.1 and it inhibits WEE-1.3 [63,69,101], thus forming two positive self regulation cycles that we include in our model without explicitly incorporating the nodes for simplicity.

Two CKIs exist in *C. elegans*, namely, *cki-1* and *cki-2*. Both are known to regulate the cell cycle, but we only included *cki-1* in the model for simplicity, and because many of the most penetrant phenotypes are the result of mutations affecting both genes [102]. The gene *cki-1* is orthologous to the mammalian cyclin-dependent kinase inhibitor p27/KIP1 and *cki-1* is required to stop the cell cycle and to stay at the G0/G1 phase. The protein CKI-1 is one of the main regulators of the postembryonic cell cycle in *C. elegans* [103]. In humans, the ortholog of FZR-1, which is a regulatory subunit of APC indirectly promotes the accumulation of the ortholog of CKI-1 [65,69]. Additionally, in yeast the ortholog of CDK-1/CYB-3 activates the ortholog of CDC-14 [65,66], then CDC-14 upregulates the accumulation of CKI-1 [65,104]. Finally, CKI-1 is negatively regulated by CDK-4/CYD-1; the evidence supporting this interaction is that CKI-1 binds to CDK-4/CYD-1 and loss of *cki-1/2* function rescues multiple aspects of the *cyd-1* loss of function (lf) and *cdk-4(lf)* mutant phenotypes [100].

The protein EFL-1 is a homolog of mammalian E2F, which inhibits the G1-to-S transition. In mammals, Rb binds to E2F, inhibiting its function as an activating transcription factor. It is worth noting that EFL-1 may need DPL-1 as a co-factor [69]. The protein LIN-35 is orthologous to Rb, CDK-4/CYD-1 negatively regulates LIN-35 activity [100,105], and in mammals, the orthologs of CDK-2 and CYE-1 are needed to fully inhibit the Rb function [105,106].

In *C. elegans*, the components of the SCF complex include the Skp1-like proteins SKR-1 and SKR-2 [97], LIN-23 (F-box), and CUL-1 [71,107,108]. The components of APC include MAT-1, MAT-2, MAT-3, CDC-26, APC-2, APC-10, APC-11, APC-17, EMB-1(APC16), EMB-27, EMB-30, and FZR-1 [97,109]. Furthermore, mammalian Cdk1 activates APC/C [110], while APC and SCF inhibit each other [110-112].

CDK-7 is a CAK ortholog, which likely associates with the cyclin CYH-1 [100], but the function of these molecules in the regulation of the cell cycle in *C. elegans* is not known because most *cdk-7* mutations are lethal. *C. elegans*, has two WEE-1 homologues, namely, *wee-1.1* and *wee-1.3*, which are active in the germ line [101,113]. There are also four CDC-25 homologues: *cdc-25.1*, *cdc-25.2*, *cdc-25.3*, and *cdc-25.4*. Of these four, only the function of CDC-25.1 as a cell cycle regulator is known [101].

#### Interactions between the molecules involved in the control of the cell cycle and the molecules involved in the control of VPC fate determination:

LIN-3/EGF activates Ras signaling. Now, LIN-1 and LIN-31—effectors of Ras—as well as the Mediator complex are necessary for cell cycle quiescence. Specifically, when Ras is active, LIN-1 and LIN-31 do not activate the transcription of CKI-1, and we included this regulation as an inhibition of CKI-1 by MPK-1 [77]. Moreover, LIN-1, LIN-31 and the Mediator complex activate *lin-39* transcription [68,85] and LIN-39 is required for the divisions of the VPCs [114]. Additionally, the three CDK/Cyclin complexes that regulate the cell cycle also regulate Notch signaling. In particular, the CDK-4/CYD-1 complex stabilizes the location of LIN-12 (NOTCH) on the cell membrane; the CDK-2/CYE-1 complex inhibits the proteolysis of the fragment of LIN-12 that functions as a transcription factor in the nucleus; and the CDK-1/CYB-3 complex activates the expulsion from the nucleus and the degradation of the LIN-12 fragment [42].

### The regulatory network as a dynamical system

We first reconstructed the cell cycle functional module from an expected time series with the use of *BoolNet* [115]. *BoolNet* produced a probabilistic Boolean network with several possible update rules for each node. We chose for each node the rule that best reflected the biological knowledge, and made a few modifications. Specifically, we included the mutual inhibition between APC and SCF, which does not change the dynamic of the wild type model, but changes the simulated effect of several mutations.

Next, we developed a deterministic discrete dynamical model by building the VPC fate determination module, and then connecting it with the cell cycle module. In our model there is one node with four possible levels of activation—LIN-3—. This characteristic is necessary because *a*) the VPCs P3.p, P8.p and P9.p, which acquire the tertiary fate, have no Ras activity (*i.e.* a level of 0), *b*) the VPCs P5.p and P7.p usually have a moderate level of Ras signaling which is sufficient to determine the secondary fate (*i.e.* a level of 1), *c*) P6.p is characterized by a high level of Ras signaling (*i.e.* a level of 2), which is sufficient to determine the primary fate, but only in the absence of negative regulation, and *d*) in some experiments with worms that have two or more anchor cells, the level of Ras signaling is high enough to overcome the effects of the negative regulators (*i.e.* a level of 3).

Two nodes of the network needed to be modeled as components with three levels of activation—MPK-1 and LIN-39—, which are at the end of the Ras signaling cascade, or downstream from it. They have no inhibitors to overcome, and hence only the levels 0, 1, and 2 are considered.

The rest of the nodes in the network were considered as Boolean, since the experimental evidence report either a full gain or total loss of function. Therefore, the rules determining the state of activation of each node as a function of their regulatory inputs are as follows:

(1) $${} \text{LIN-3}(t + 1) = \text{LIN-3}(t)  $$

(2) $${} {\fontsize{9.2pt}{9.6pt}\selectfont{\begin{aligned} \text{MPK-1}(t + 1) = \left\{ \begin{array}{lll} 2 & \text{if } (\text{LIN-3}(t) = 3 \text{ and MPK-1}(t) > 0) \text{ or} \\ & \text{(LIN-3}(t) = 2 \text{ and LIN-12i}(t) = 0 \text{ and}\\ & \text{MPK-1}(t) > 0)\\ 0 & \text{if }\text{MPK-1}(t) < 2 \text{ and} \\ & ((\text{LIN-3}(t) = 1 \text{ and }\text{LIN-12i}(t) = 1) \text{or}\\ & (\text{LIN-3}(t) = 0))\\ 1 & \text{otherwise} \end{array} \right. \end{aligned}}}  $$

(3) $${} {\fontsize{9.2pt}{9.6pt}\selectfont{\begin{aligned} \text{LIN-39}(t + 1) = \left\{ \begin{array}{lll} 2 & \text{if MPK-1}(t) = 2 \text{ and LIN-39}(t) > 0 \\ 0 & \text{When simulating \textit{lin-39} loss of function } \\ 1 & \text{otherwise} \end{array} \right. \end{aligned}}}  $$

(4) $${} \text{LS} (t + 1) = \text{LS} (t)  $$

(5) $${} {\fontsize{8.6pt}{9.6pt}\selectfont{\begin{aligned} \text{LIN-12m}(t + 1) = \left\{ \begin{array}{lll} 1 & \text{if } (\text{LIN-39}(t) > 0 \text{ or LIN-12i}(t) = 1) \text{ and } \\ & (\text{MPK-1}(t) \leq 1 \text{ or CDK-4/CYD-1}(t) = 1) \\ 0 & \text{otherwise } \end{array} \right. \end{aligned}}}  $$

(6) $${} {\fontsize{9.2pt}{9.6pt}\selectfont{\begin{aligned} \text{LIN-12i}(t + 1) = \left\{ \begin{array}{lll} 1 & \text{if (LS}(t) = 1 \text{ and LIN-12m}(t) = 1) \text{ or }\\ & \text{((LIN-12i}(t) = 1 \text{or LIN-3}(t) = 1) \text{ and }\\ & (\text{CDK-2/CYE-1}(t) = 1 \text{ or} \\ & \text{CDK-1/CYB-3}(t) = 0)) \\ 0 & \text{otherwise } \end{array} \right. \end{aligned}}}  $$

(7) $${} {\fontsize{9.2pt}{9.6pt}\selectfont{\begin{aligned} \text{CKI-1}(t + 1) = \left\{ \begin{array}{lll} 1 & \text{if (MPK-1}(t) = 0) \text{ and} \\ & \text{(CDK-4/CYD-1}(t) = 0 \text{ and APC}(t) = 1 \\ & \text{or CDK-1/CYB-3}(t) = 1))\\ 0 & \text{otherwise } \end{array} \right. \end{aligned}}}  $$

(8) $$ \text{EFL-1}(t + 1) = \left\{ \begin{array}{lll} 1 & \text{if LIN-35}(t) = 0\\ 0 & \text{otherwise} \end{array} \right.  $$

(9) $${} \text{LIN-35}(t + 1) = \left\{ \begin{array}{lll} 1 & \text{if CDK-4/CYD-1}(t) = 0 \text{ and } \\ & \text{CDK-2/CYE-1}(t) = 0\\ 0 & \text{otherwise } \end{array} \right.  $$

(10) $${} \text{SCF}(t + 1) = \left\{ \begin{array}{lll} 1 & \text{if LIN-39}(t) > 0 \text{ and APC}(t) = 0 \\ & \text{and CDK-2/CYE-1}(t) = 1\\ 0 & \text{otherwise } \end{array} \right.  $$

(11) $${} \text{APC}(t + 1) = \left\{ \begin{array}{lll} 1 & \text{if SCF}(t) = 0 \text{ and CDK-1/CYB-3}(t) = 1 \\ 0 & \text{otherwise } \end{array} \right.  $$

(12) $${} {\fontsize{9.2pt}{9.6pt}\selectfont{\begin{aligned} \text{CDK-4/CYD-1}(t + 1) = \left\{ \begin{array}{lll} 1 & \text{if CKI-1}(t) = 0 \text{ and SCF}(t) = 0 \\ & \text{and CDK-1/CYB-3}(t) = 0 \\ 0 & \text{otherwise } \end{array} \right. \end{aligned}}}  $$

(13) $${} {\fontsize{8.8pt}{9.6pt}\selectfont{\begin{aligned} \text{CDK-2/CYE-1}(t + 1) = \left\{ \begin{array}{lll} 1 & \text{if EFL-1}(t) = 1 \text{ and LIN-35}(t) = 0 \\ & \text{and CKI-1}(t) = 0 \text{ and SCF}(t) = 0 \\ 0 & \text{otherwise } \end{array} \right. \end{aligned}}}  $$

(14) $${} {\fontsize{9.2pt}{9.6pt}\selectfont{\begin{aligned} \text{CDK-1/CYB-3}(t + 1) = \left\{ \begin{array}{lll} 1 & \text{if CKI-1}(t) = 0 \text{ and APC}(t) = 0 \\ & \text{and EFL-1}(t) = 1 \\ & \text{and (CDK-4/CYD-1}(t) = 0 \\ & \text{or CDK-1/CYB-3}(t) = 1)\\ 0 & \text{otherwise } \end{array} \right. \end{aligned}}}  $$

Subsequently, we used the software package *Griffin* [73], to find the interactions that are necessary to recover a cyclic attractor that visits the configurations characteristic of each cell-cycle phase in the temporal sequence that is observed in actual cells. Additionally, we specified that all the interaction signs must be unambiguous.

Finally, we used the SQUAD methodology [116] to build a deterministic continuous version of the cell cycle module, based on the Boolean update rules that we obtained based on an expected time series with the use of *BoolNet*. We used the continuous version of the module to verify that the cyclic behavior of the network is not an artifact of the synchronous updating of the discrete model [63]. The differential equations describing the continuous model are as follows:

(15) $${} squad(X_{i}, \omega_{i}) = \frac{- {e}^{0.5 h} + {e}^{-h(\omega_{i} - 0.5)}}{(1 - {e}^{0.5 h})(1 + {e}^{h (\omega_{i} - 0.5)})} - \gamma_{i} X_{i}  $$

(16) $${} h = 10  $$

(17) $${} \gamma_{i} = 0.95  $$

(18) $${} {\fontsize{8.7pt}{9.6pt}\selectfont{\begin{aligned} \omega_{CKI-1} = \min((1 - \text{CDK-4/CYD-1)}, \max(\text{APC, CDK-1/CYB-3})) \end{aligned}}}  $$

(19) $${} \frac{d(\text{CKI-1})}{dt} = squad(\text{CKI-1, } \omega_{CKI-1})  $$

(20) $${} \omega_{EFL-1} = 1 - \text{LIN-35}  $$

(21) $${} \frac{d(\text{EFL-1})}{dt} = squad(\text{EFL-1, } \omega_{EFL-1})  $$

(22) $${} \omega_{LIN-35} = \min(1 - \text{CDK-4/CYD-1}, 1 - \text{CDK-2/CYE-1}))  $$

(23) $${} \frac{d(\text{LIN-35})}{dt} = squad(\text{LIN-35, } \omega_{LIN-35})  $$

(24) $${} \omega_{SCF} = \min(1 - \text{APC, CDK-2/CYE-1}))  $$

(25) $${} \frac{d(\text{SCF})}{dt} = squad(\text{SCF, } \omega_{SCF})  $$

(26) $${} \omega_{APC} = \min(1 - \text{SCF, CDK-1/CYB-3}))  $$

(27) $${} \frac{d(\text{APC})}{dt} = squad(\text{APC, } \omega_{APC})  $$

(28) $${} {\fontsize{8.8pt}{9.6pt}\selectfont{\begin{aligned} \omega_{CDK-4/CYD-1} = \min(1 - \text{CKI-1}, 1 - \text{SCF}, 1 - \text{CDK-1/CYB-3}) \end{aligned}}}  $$

(29) $${} {\fontsize{8.8pt}{9.6pt}\selectfont{\begin{aligned} \frac{d(\text{CDK-4/CYD-1})}{dt} = squad(\text{CDK-4/CYD-1, } \omega_{CDK-4/CYD-1}) \end{aligned}}}  $$

(30) $${} {\fontsize{8.8pt}{9.6pt}\selectfont{\begin{aligned} \omega_{CDK-2/CYE-1} = \min(\text{EFL-1}, 1 - \text{LIN-35}, 1 - \text{CKI-1}, 1 - \text{SCF}) \end{aligned}}}  $$

(31) $${} {\fontsize{8.8pt}{9.6pt}\selectfont{\begin{aligned} \frac{d(\text{CDK-2/CYE-1})}{dt} = squad(\text{CDK-2/CYE-1, } \omega_{CDK-2/CYE-1}) \end{aligned}}}  $$

(32) $${} {\fontsize{8.8pt}{9.6pt}\selectfont{\begin{aligned} &\omega_{CDK-1/CYB-3} = \min(1 - \text{CKI-1}, 1 - \text{APC}, \text{EFL-1},\\ &\max(1 - \text{CDK-4/CYD-1}, \text{CDK-1/CYB-3})) \end{aligned}}}  $$

(33) $$ {} {\fontsize{8.8pt}{9.6pt}\selectfont{\begin{aligned} \frac{d(\text{CDK-1/CYB-3})}{dt} = squad(\text{CDK-1/CYB-3, } \omega_{CDK-1/CYB-3}) \end{aligned}}}  $$

## Endnote

^a^ *Griffin* is a symbolic computational tool under development that uses a SAT solver to find Boolean networks satisfying certain constraints, for example, the existence of known or hypothetical interactions.

## Additional files

Additional file 1
**Table S1.** Simulation of mutants and their phenotypic effect [8,35,71,86,100,103,114,117-121].

Additional file 2
**Mutations.** This file contains the attractors produced by the simulation of each mutation.

Additional file 3
**Table S2.** The simulated phenotypic effect caused by removing each interaction [25,27,38,42,68,85,86,88,91-94,117,119,122,123].

Additional file 4
**Interactions.** This file contains the attractors produced by the simulation of each interaction removal.
