# Supplementary material for: A model of the regulatory network involved in the control of the cell cycle and cell differentiation in the Caenorhabditis elegans vulva
Source: BMC Bioinformatics. 2015 Mar 13;16:81. doi: 10.1186/s12859-015-0498-z (PMC4367908; doi:10.1186/s12859-015-0498-z)
Supplement: Additional file 1 — Table S1. Simulation of mutants and their phenotypic effect [8,35,71,86,100,103,114,117-121]. [file 12859_2015_498_MOESM1_ESM.pdf]

Additional file 1: Table S1 Simulation of mutants and their phenotypic effect

Phenotypes	Mutations
Wild type	*lin-12m(1) and lin-12m(0) [35,69], LS(0) [70]*
No primary fate	*lin-3(0) [8], lin3(1) [8], mpk-1(0) [71], mpk-1(1), lin-39(0) [72], lin-39(1) [72]*
No secondary fate	*lin-12i(0), lin-3(3) [8,35] mpk-1(2), lin-39(2) [72]*
No tertiary fate	*lin-12i(1) [69], lin-3(1) [73], lin-3(2) [73], lin-3(3) [8,35] mpk-1(1) [71], mpk-1(2) [71] LS(1)*
No divisions	*lin-39(0) [74], cki-1(1) [75], efl-1(0), lin-35(1), SCF(1), SCF(0) [76], APC(1), CDK-4/CYD-1(0) [77], CDK-2/CYE-1(0) [78], CDK-2/CYE-1(1), CDK-1/CYB-3(1)*
Endo-replication	*CDK-4/CYD-1(1) [77,78], CDK-1/CYB-3(0)*
No gap phases	*CDK- 2/CYE-1(1) [77]*
Modified cell cycle	*cki-1(0) [75], efl-1(1), lin-35(0), APC(0), CDK-4/CYD-1(1), CDK- 2/CYE-1(1)*
Secondary with LIN-3(2) and LS(0)	*lin-39(0), lin-12i(1), cki-1(1), efl-1(0), lin-35(0), lin-35(1), SCF(1), SCF(0), APC(1), CDK-4/CYD-1(1), CDK-4/CYD-1(0), CDK-2/CYE-1(1), CDK-2/CYE-1(0), CDK-1/CYB-3(0), CDK-1/CYB-3(1)*
Primary with LIN-3(2) and LS(1)	*mpk-1(2), lin-39(2), lin-12m(0), lin-12i(0), cki-1(1), SCF(1), CDK-4/CYD-1(0), CDK- 2/CYE-1(1), CDK-1/CYB-3(1)*
Secondary with LIN-3(0) and LS(0)	*lin-39(0), lin-12i(0), cki-1(1), efl-1(0), lin-35(0), lin-35(1), SCF(1), SCF(0), APC(1), CDK-4/CYD-1(1), CDK-4/CYD-1(0), CDK- 2/CYE-1(1), CDK-2/CYE-1(0), CDK-1/CYB-3(0), CDK-1/CYB-3(1)*
Tertiary with LIN-3(0) and LS(1)	*lin-39(0), lin-12i(0)*

Mutants in black have a phenotype that reproduce what is reported in the literature; mutants in red have a simulated effect that differs from what is reported in the literature; and mutants in blue are predictions of our model.
