# Supplementary material for: A model of the regulatory network involved in the control of the cell cycle and cell differentiation in the Caenorhabditis elegans vulva
Source: BMC Bioinformatics. 2015 Mar 13;16:81. doi: 10.1186/s12859-015-0498-z (PMC4367908; doi:10.1186/s12859-015-0498-z)
Supplement: Additional file 2 — Mutations. This file contains the attractors produced by the simulation of each mutation. [file 12859_2015_498_MOESM2_ESM.pdf]

Mutations

LIN-3 0

**LIN-3**	MPK-1	LIN-39	LS	LIN-12m	LIN-12i	CKI-1	EFL-1	LIN-35	SCF	APC	CDK-4/CYD-1	CDK-2/CYE-1	CDK-1/CYB-3
0	0	1	0	1	0	0	0	0	0	0	1	0	0
0	0	1	0	1	0	0	1	0	0	0	1	0	0
0	0	1	0	1	0	0	1	0	0	0	1	1	0
0	0	1	0	1	0	0	1	0	1	0	1	1	0
0	0	1	0	1	0	0	1	0	1	0	0	0	0
0	0	1	0	1	0	0	1	1	0	0	0	0	1
0	0	1	0	1	0	1	0	1	0	1	0	0	1
0	0	1	0	1	0	1	0	1	0	1	0	0	0
0	0	1	0	1	0	1	0	1	0	0	0	0	0
0	0	1	0	1	0	0	0	1	0	0	0	0	0
0	0	1	0	1	0	0	0	1	0	0	1	0	0

LIN-3	MPK-1	LIN-39	LS	LIN-12m	LIN-12i	CKI-1	EFL-1	LIN-35	SCF	APC	CDK-4/CYD-1	CDK-2/CYE-1	CDK-1/CYB-3
0	0	1	1	1	0	1	0	1	0	1	0	0	0
0	0	1	1	1	1	1	0	1	0	0	0	0	0
0	0	1	1	1	1	0	0	1	0	0	0	0	0
0	0	1	1	1	1	0	0	1	0	0	1	0	0
0	0	1	1	1	1	0	0	0	0	0	1	0	0
0	0	1	1	1	1	0	1	0	0	0	1	0	0
0	0	1	1	1	1	0	1	0	0	0	1	1	0
0	0	1	1	1	1	0	1	0	1	0	1	1	0
0	0	1	1	1	1	0	1	0	1	0	0	0	0
0	0	1	1	1	1	0	1	1	0	0	0	0	1
0	0	1	1	1	0	1	0	1	0	1	0	0	1

LIN-3 1

LIN-3	MPK-1	LIN-39	LS	LIN-12m	LIN-12i	CKI-1	EFL-1	LIN-35	SCF	APC	CDK-4/CYD-1	CDK-2/CYE-1	CDK-1/CYB-3
1	0	1	0	1	0	1	0	1	0	1	0	0	1
**1**	1	1	0	1	0	1	0	1	0	1	0	0	0
1	1	1	0	1	1	0	0	1	0	0	0	0	0
1	0	1	0	1	1	0	0	1	0	0	1	0	0
1	0	1	0	1	1	0	0	0	0	0	1	0	0
1	0	1	0	1	1	0	1	0	0	0	1	0	0
1	0	1	0	1	1	0	1	0	0	0	1	1	0
1	0	1	0	1	1	0	1	0	1	0	1	1	0
1	0	1	0	1	1	0	1	0	1	0	0	0	0
1	0	1	0	1	1	0	1	1	0	0	0	0	1

LIN-3	MPK-1	LIN-39	LS	LIN-12m	LIN-12i	CKI-1	EFL-1	LIN-35	SCF	APC	CDK-4/CYD-1	CDK-2/CYE-1	CDK-1/CYB-3
1	0	1	1	1	0	1	0	1	0	1	0	0	1
1	1	1	1	1	0	1	0	1	0	1	0	0	0
1	1	1	1	1	1	0	0	1	0	0	0	0	0
1	0	1	1	1	1	0	0	1	0	0	1	0	0
1	0	1	1	1	1	0	0	0	0	0	1	0	0
1	0	1	1	1	1	0	1	0	0	0	1	0	0
1	0	1	1	1	1	0	1	0	0	0	1	1	0
1	0	1	1	1	1	0	1	0	1	0	1	1	0
1	0	1	1	1	1	0	1	0	1	0	0	0	0
1	0	1	1	1	1	0	1	1	0	0	0	0	1

LIN-3 2

LIN-3	MPK-1	LIN-39	LS	LIN-12m	LIN-12i	CKI-1	EFL-1	LIN-35	SCF	APC	CDK-4/CYD-1	CDK-2/CYE-1	CDK-1/CYB-3
2	1	1	1	1	0	0	0	1	0	1	0	0	1
2	2	1	1	1	0	0	0	1	0	1	0	0	0
2	2	2	1	0	1	0	0	1	0	0	1	0	0
2	1	2	1	1	1	0	0	0	0	0	1	0	0
2	1	1	1	1	1	0	1	0	0	0	1	0	0
2	1	1	1	1	1	0	1	0	0	0	1	1	0
2	1	1	1	1	1	0	1	0	1	0	1	1	0
2	1	1	1	1	1	0	1	0	1	0	0	0	0
2	1	1	1	1	1	0	1	1	0	0	0	0	1

LIN-3	MPK-1	LIN-39	LS	LIN-12m	LIN-12i	CKI-1	EFL-1	LIN-35	SCF	APC	CDK-4/CYD-1	CDK-2/CYE-1	CDK-1/CYB-3
2	2	2	0	0	0	0	0	1	0	0	1	0	0
2	2	2	0	1	0	0	0	0	0	0	1	0	0
2	2	2	0	1	0	0	1	0	0	0	1	0	0
2	2	2	0	1	0	0	1	0	0	0	1	1	0
2	2	2	0	1	0	0	1	0	1	0	1	1	0
2	2	2	0	1	0	0	1	0	1	0	0	0	0
2	2	2	0	0	0	0	1	1	0	0	0	0	1
2	2	2	0	0	0	0	0	1	0	1	0	0	1
2	2	2	0	0	0	0	0	1	0	1	0	0	0

LIN-3 3

LIN-3	MPK-1	LIN-39	LS	LIN-12m	LIN-12i	CKI-1	EFL-1	LIN-35	SCF	APC	CDK-4/CYD-1	CDK-2/CYE-1	CDK-1/CYB-3
3	2	2	0	0	0	0	0	1	0	0	1	0	0
3	2	2	0	1	0	0	0	0	0	0	1	0	0
3	2	2	0	1	0	0	1	0	0	0	1	0	0
3	2	2	0	1	0	0	1	0	0	0	1	1	0
3	2	2	0	1	0	0	1	0	1	0	1	1	0
3	2	2	0	1	0	0	1	0	1	0	0	0	0
3	2	2	0	0	0	0	1	1	0	0	0	0	1

3	2	2	0	0	0	0	0	1	0	1	0	0	1
3	2	2	0	0	0	0	0	1	0	1	0	0	0

LIN-3	MPK-1	LIN-39	LS	LIN-12m	LIN-12i	CKI-1	EFL-1	LIN-35	SCF	APC	CDK-4/CYD-1	CDK-2/CYE-1	CDK-1/CYB-3
3	2	2	1	0	0	0	0	1	0	0	1	0	0
3	2	2	1	1	0	0	0	0	0	0	1	0	0
3	2	2	1	1	1	0	1	0	0	0	1	0	0
3	2	2	1	1	1	0	1	0	0	0	1	1	0
3	2	2	1	1	1	0	1	0	1	0	1	1	0
3	2	2	1	1	1	0	1	0	1	0	0	0	0
3	2	2	1	0	1	0	1	1	0	0	0	0	1
3	2	2	1	0	0	0	0	1	0	1	0	0	1
3	2	2	1	0	0	0	0	1	0	1	0	0	0

MPK-1 0

LIN-3	MPK-1	LIN-39	LS	LIN-12m	LIN-12i	CKI-1	EFL-1	LIN-35	SCF	APC	CDK-4/CYD-1	CDK-2/CYE-1	CDK-1/CYB-3
0	0	1	0	1	0	0	0	0	0	0	1	0	0
0	0	1	0	1	0	0	1	0	0	0	1	0	0
0	0	1	0	1	0	0	1	0	0	0	1	1	0
0	0	1	0	1	0	0	1	0	1	0	1	1	0
0	0	1	0	1	0	0	1	0	1	0	0	0	0
0	0	1	0	1	0	0	1	1	0	0	0	0	1
0	0	1	0	1	0	1	0	1	0	1	0	0	1
0	0	1	0	1	0	1	0	1	0	1	0	0	0
0	0	1	0	1	0	1	0	1	0	0	0	0	0
0	0	1	0	1	0	0	0	1	0	0	0	0	0
0	0	1	0	1	0	0	0	1	0	0	1	0	0

LIN-3	MPK-1	LIN-39	LS	LIN-12m	LIN-12i	CKI-1	EFL-1	LIN-35	SCF	APC	CDK-4/CYD-1	CDK-2/CYE-1	CDK-1/CYB-3
0	0	1	1	1	0	1	0	1	0	1	0	0	0
0	0	1	1	1	1	1	0	1	0	0	0	0	0
0	0	1	1	1	1	0	0	1	0	0	0	0	0
0	0	1	1	1	1	0	0	1	0	0	1	0	0
0	0	1	1	1	1	0	0	0	0	0	1	0	0
0	0	1	1	1	1	0	1	0	0	0	1	0	0
0	0	1	1	1	1	0	1	0	0	0	1	1	0
0	0	1	1	1	1	0	1	0	1	0	1	1	0
0	0	1	1	1	1	0	1	0	1	0	0	0	0
0	0	1	1	1	1	0	1	1	0	0	0	0	1
0	0	1	1	1	0	1	0	1	0	1	0	0	1

LIN-3	MPK-1	LIN-39	LS	LIN-12m	LIN-12i	CKI-1	EFL-1	LIN-35	SCF	APC	CDK-4/CYD-1	CDK-2/CYE-1	CDK-1/CYB-3
1	0	1	0	1	0	1	0	1	0	1	0	0	0
1	0	1	0	1	1	1	0	1	0	0	0	0	0
1	0	1	0	1	1	0	0	1	0	0	0	0	0
1	0	1	0	1	1	0	0	1	0	0	1	0	0
1	0	1	0	1	1	0	0	0	0	0	1	0	0
1	0	1	0	1	1	0	1	0	0	0	1	0	0
1	0	1	0	1	1	0	1	0	0	0	1	1	0
1	0	1	0	1	1	0	1	0	1	0	1	1	0
1	0	1	0	1	1	0	1	0	1	0	0	0	0
1	0	1	0	1	1	0	1	1	0	0	0	0	1
1	0	1	0	1	0	1	0	1	0	1	0	0	1

LIN-3	MPK-1	LIN-39	LS	LIN-12m	LIN-12i	CKI-1	EFL-1	LIN-35	SCF	APC	CDK-4/CYD-1	CDK-2/CYE-1	CDK-1/CYB-3
1	0	1	1	1	0	1	0	1	0	1	0	0	0
1	0	1	1	1	1	1	0	1	0	0	0	0	0
1	0	1	1	1	1	0	0	1	0	0	0	0	0
1	0	1	1	1	1	0	0	1	0	0	1	0	0
1	0	1	1	1	1	0	0	0	0	0	1	0	0
1	0	1	1	1	1	0	1	0	0	0	1	0	0
1	0	1	1	1	1	0	1	0	0	0	1	1	0
1	0	1	1	1	1	0	1	0	1	0	1	1	0
1	0	1	1	1	1	0	1	0	1	0	0	0	0
1	0	1	1	1	1	0	1	1	0	0	0	0	1
1	0	1	1	1	0	1	0	1	0	1	0	0	1

LIN-3	MPK-1	LIN-39	LS	LIN-12m	LIN-12i	CKI-1	EFL-1	LIN-35	SCF	APC	CDK-4/CYD-1	CDK-2/CYE-1	CDK-1/CYB-3
2	0	1	0	1	0	0	0	0	0	0	1	0	0
2	0	1	0	1	0	0	1	0	0	0	1	0	0
2	0	1	0	1	0	0	1	0	0	0	1	1	0
2	0	1	0	1	0	0	1	0	1	0	1	1	0
2	0	1	0	1	0	0	1	0	1	0	0	0	0
2	0	1	0	1	0	0	1	1	0	0	0	0	1
2	0	1	0	1	0	1	0	1	0	1	0	0	1
2	0	1	0	1	0	1	0	1	0	1	0	0	0
2	0	1	0	1	0	1	0	1	0	0	0	0	0
2	0	1	0	1	0	0	0	1	0	0	0	0	0
2	0	1	0	1	0	0	0	1	0	0	1	0	0

LIN-3	MPK-1	LIN-39	LS	LIN-12m	LIN-12i	CKI-1	EFL-1	LIN-35	SCF	APC	CDK-4/CYD-1	CDK-2/CYE-1	CDK-1/CYB-3
2	0	1	1	1	0	1	0	1	0	1	0	0	0
2	0	1	1	1	1	1	0	1	0	0	0	0	0
2	0	1	1	1	1	0	0	1	0	0	0	0	0
2	0	1	1	1	1	0	0	1	0	0	1	0	0
2	0	1	1	1	1	0	0	0	0	0	1	0	0

2	0	1	1	1	1	0	1	0	0	0	1	0	0
2	0	1	1	1	1	0	1	0	0	0	1	1	0
2	0	1	1	1	1	0	1	0	1	0	1	1	0
2	0	1	1	1	1	0	1	0	1	0	0	0	0
2	0	1	1	1	1	0	1	1	0	0	0	0	1
2	0	1	1	1	0	1	0	1	0	1	0	0	1

LIN-3	MPK-1	LIN-39	LS	LIN-12m	LIN-12i	CKI-1	EFL-1	LIN-35	SCF	APC	CDK-4/CYD-1	CDK-2/CYE-1	CDK-1/CYB-3
3	0	1	0	1	0	0	0	0	0	0	1	0	0
3	0	1	0	1	0	0	1	0	0	0	1	0	0
3	0	1	0	1	0	0	1	0	0	0	1	1	0
3	0	1	0	1	0	0	1	0	1	0	1	1	0
3	0	1	0	1	0	0	1	0	1	0	0	0	0
3	0	1	0	1	0	0	1	1	0	0	0	0	1
3	0	1	0	1	0	1	0	1	0	1	0	0	1
3	0	1	0	1	0	1	0	1	0	1	0	0	0
3	0	1	0	1	0	1	0	1	0	0	0	0	0
3	0	1	0	1	0	0	0	1	0	0	0	0	0
3	0	1	0	1	0	0	0	1	0	0	1	0	0

LIN-3	MPK-1	LIN-39	LS	LIN-12m	LIN-12i	CKI-1	EFL-1	LIN-35	SCF	APC	CDK-4/CYD-1	CDK-2/CYE-1	CDK-1/CYB-3
3	0	1	1	1	0	1	0	1	0	1	0	0	0
3	0	1	1	1	1	1	0	1	0	0	0	0	0
3	0	1	1	1	1	0	0	1	0	0	0	0	0
3	0	1	1	1	1	0	0	1	0	0	1	0	0
3	0	1	1	1	1	0	0	0	0	0	1	0	0
3	0	1	1	1	1	0	1	0	0	0	1	0	0
3	0	1	1	1	1	0	1	0	0	0	1	1	0
3	0	1	1	1	1	0	1	0	1	0	1	1	0
3	0	1	1	1	1	0	1	0	1	0	0	0	0
3	0	1	1	1	1	0	1	1	0	0	0	0	1
3	0	1	1	1	0	1	0	1	0	1	0	0	1

MPK-1 1

LIN-3	MPK-1	LIN-39	LS	LIN-12m	LIN-12i	CKI-1	EFL-1	LIN-35	SCF	APC	CDK-4/CYD-1	CDK-2/CYE-1	CDK-1/CYB-3
0	1	1	0	1	0	0	0	0	0	0	1	0	0
0	1	1	0	1	0	0	1	0	0	0	1	0	0
0	1	1	0	1	0	0	1	0	0	0	1	1	0
0	1	1	0	1	0	0	1	0	1	0	1	1	0
0	1	1	0	1	0	0	1	0	1	0	0	0	0
0	1	1	0	1	0	0	1	1	0	0	0	0	1
0	1	1	0	1	0	0	0	1	0	1	0	0	1
0	1	1	0	1	0	0	0	1	0	1	0	0	0
0	1	1	0	1	0	0	0	1	0	0	1	0	0

LIN-3	MPK-1	LIN-39	LS	LIN-12m	LIN-12i	CKI-1	EFL-1	LIN-35	SCF	APC	CDK-4/CYD-1	CDK-2/CYE-1	CDK-1/CYB-3
0	1	1	1	1	0	0	0	1	0	1	0	0	0
0	1	1	1	1	1	0	0	1	0	0	1	0	0
0	1	1	1	1	1	0	0	0	0	0	1	0	0
0	1	1	1	1	1	0	1	0	0	0	1	0	0
0	1	1	1	1	1	0	1	0	0	0	1	1	0
0	1	1	1	1	1	0	1	0	1	0	1	1	0
0	1	1	1	1	1	0	1	0	1	0	0	0	0
0	1	1	1	1	1	0	1	1	0	0	0	0	1
0	1	1	1	1	0	0	0	1	0	1	0	0	1

LIN-3	MPK-1	LIN-39	LS	LIN-12m	LIN-12i	CKI-1	EFL-1	LIN-35	SCF	APC	CDK-4/CYD-1	CDK-2/CYE-1	CDK-1/CYB-3
1	1	1	0	1	0	0	0	1	0	1	0	0	0
1	1	1	0	1	1	0	0	1	0	0	1	0	0
1	1	1	0	1	1	0	0	0	0	0	1	0	0
1	1	1	0	1	1	0	1	0	0	0	1	0	0
1	1	1	0	1	1	0	1	0	0	0	1	1	0
1	1	1	0	1	1	0	1	0	1	0	1	1	0
1	1	1	0	1	1	0	1	0	1	0	0	0	0
1	1	1	0	1	1	0	1	1	0	0	0	0	1
1	1	1	0	1	0	0	0	1	0	1	0	0	1

LIN-3	MPK-1	LIN-39	LS	LIN-12m	LIN-12i	CKI-1	EFL-1	LIN-35	SCF	APC	CDK-4/CYD-1	CDK-2/CYE-1	CDK-1/CYB-3
1	1	1	1	1	0	0	0	1	0	1	0	0	0
1	1	1	1	1	1	0	0	1	0	0	1	0	0
1	1	1	1	1	1	0	0	0	0	0	1	0	0
1	1	1	1	1	1	0	1	0	0	0	1	0	0
1	1	1	1	1	1	0	1	0	0	0	1	1	0
1	1	1	1	1	1	0	1	0	1	0	1	1	0
1	1	1	1	1	1	0	1	0	1	0	0	0	0
1	1	1	1	1	1	0	1	1	0	0	0	0	1
1	1	1	1	1	0	0	0	1	0	1	0	0	1

LIN-3	MPK-1	LIN-39	LS	LIN-12m	LIN-12i	CKI-1	EFL-1	LIN-35	SCF	APC	CDK-4/CYD-1	CDK-2/CYE-1	CDK-1/CYB-3
2	1	1	0	1	0	0	0	0	0	0	1	0	0
2	1	1	0	1	0	0	1	0	0	0	1	0	0
2	1	1	0	1	0	0	1	0	0	0	1	1	0
2	1	1	0	1	0	0	1	0	1	0	1	1	0
2	1	1	0	1	0	0	1	0	1	0	0	0	0
2	1	1	0	1	0	0	1	1	0	0	0	0	1
2	1	1	0	1	0	0	0	1	0	1	0	0	1

2	1	1	0	1	0	0	0	1	0	1	0	0	0
2	1	1	0	1	0	0	0	1	0	0	1	0	0

LIN-3	MPK-1	LIN-39	LS	LIN-12m	LIN-12i	CKI-1	EFL-1	LIN-35	SCF	APC	CDK-4/CYD-1	CDK-2/CYE-1	CDK-1/CYB-3
2	1	1	1	1	0	0	0	1	0	1	0	0	0
2	1	1	1	1	1	0	0	1	0	0	1	0	0
2	1	1	1	1	1	0	0	0	0	0	1	0	0
2	1	1	1	1	1	0	1	0	0	0	1	0	0
2	1	1	1	1	1	0	1	0	0	0	1	1	0
2	1	1	1	1	1	0	1	0	1	0	1	1	0
2	1	1	1	1	1	0	1	0	1	0	0	0	0
2	1	1	1	1	1	0	1	1	0	0	0	0	1
2	1	1	1	1	0	0	0	1	0	1	0	0	1

LIN-3	MPK-1	LIN-39	LS	LIN-12m	LIN-12i	CKI-1	EFL-1	LIN-35	SCF	APC	CDK-4/CYD-1	CDK-2/CYE-1	CDK-1/CYB-3
3	1	1	0	1	0	0	0	0	0	0	1	0	0
3	1	1	0	1	0	0	1	0	0	0	1	0	0
3	1	1	0	1	0	0	1	0	0	0	1	1	0
3	1	1	0	1	0	0	1	0	1	0	1	1	0
3	1	1	0	1	0	0	1	0	1	0	0	0	0
3	1	1	0	1	0	0	1	1	0	0	0	0	1
3	1	1	0	1	0	0	0	1	0	1	0	0	1
3	1	1	0	1	0	0	0	1	0	1	0	0	0
3	1	1	0	1	0	0	0	1	0	0	1	0	0

LIN-3	MPK-1	LIN-39	LS	LIN-12m	LIN-12i	CKI-1	EFL-1	LIN-35	SCF	APC	CDK-4/CYD-1	CDK-2/CYE-1	CDK-1/CYB-3
3	1	1	1	1	0	0	0	1	0	1	0	0	0
3	1	1	1	1	1	0	0	1	0	0	1	0	0
3	1	1	1	1	1	0	0	0	0	0	1	0	0
3	1	1	1	1	1	0	1	0	0	0	1	0	0
3	1	1	1	1	1	0	1	0	0	0	1	1	0
3	1	1	1	1	1	0	1	0	1	0	1	1	0
3	1	1	1	1	1	0	1	0	1	0	0	0	0
3	1	1	1	1	1	0	1	1	0	0	0	0	1
3	1	1	1	1	0	0	0	1	0	1	0	0	1

MPK-1 2

LIN-3	MPK-1	LIN-39	LS	LIN-12m	LIN-12i	CKI-1	EFL-1	LIN-35	SCF	APC	CDK-4/CYD-1	CDK-2/CYE-1	CDK-1/CYB-3
0	2	2	0	0	0	0	0	1	0	0	1	0	0
0	2	2	0	1	0	0	0	0	0	0	1	0	0
0	2	2	0	1	0	0	1	0	0	0	1	0	0
0	2	2	0	1	0	0	1	0	0	0	1	1	0
0	2	2	0	1	0	0	1	0	1	0	1	1	0
0	2	2	0	1	0	0	1	0	1	0	0	0	0
0	2	2	0	0	0	0	1	1	0	0	0	0	1
0	2	2	0	0	0	0	0	1	0	1	0	0	1
0	2	2	0	0	0	0	0	1	0	1	0	0	0

LIN-3	MPK-1	LIN-39	LS	LIN-12m	LIN-12i	CKI-1	EFL-1	LIN-35	SCF	APC	CDK-4/CYD-1	CDK-2/CYE-1	CDK-1/CYB-3
0	2	2	1	0	0	0	0	1	0	0	1	0	0
0	2	2	1	1	0	0	0	0	0	0	1	0	0
0	2	2	1	1	1	0	1	0	0	0	1	0	0
0	2	2	1	1	1	0	1	0	0	0	1	1	0
0	2	2	1	1	1	0	1	0	1	0	1	1	0
0	2	2	1	1	1	0	1	0	1	0	0	0	0
0	2	2	1	0	1	0	1	1	0	0	0	0	1
0	2	2	1	0	0	0	0	1	0	1	0	0	1
0	2	2	1	0	0	0	0	1	0	1	0	0	0

LIN-3	MPK-1	LIN-39	LS	LIN-12m	LIN-12i	CKI-1	EFL-1	LIN-35	SCF	APC	CDK-4/CYD-1	CDK-2/CYE-1	CDK-1/CYB-3
1	2	2	0	0	0	0	0	1	0	1	0	0	0
1	2	2	0	0	1	0	0	1	0	0	1	0	0
1	2	2	0	1	1	0	0	0	0	0	1	0	0
1	2	2	0	1	1	0	1	0	0	0	1	0	0
1	2	2	0	1	1	0	1	0	0	0	1	1	0
1	2	2	0	1	1	0	1	0	1	0	1	1	0
1	2	2	0	1	1	0	1	0	1	0	0	0	0
1	2	2	0	0	1	0	1	1	0	0	0	0	1
1	2	2	0	0	0	0	0	1	0	1	0	0	1

LIN-3	MPK-1	LIN-39	LS	LIN-12m	LIN-12i	CKI-1	EFL-1	LIN-35	SCF	APC	CDK-4/CYD-1	CDK-2/CYE-1	CDK-1/CYB-3
1	2	2	1	0	0	0	0	1	0	1	0	0	0
1	2	2	1	0	1	0	0	1	0	0	1	0	0
1	2	2	1	1	1	0	0	0	0	0	1	0	0
1	2	2	1	1	1	0	1	0	0	0	1	0	0
1	2	2	1	1	1	0	1	0	0	0	1	1	0
1	2	2	1	1	1	0	1	0	1	0	1	1	0
1	2	2	1	1	1	0	1	0	1	0	0	0	0
1	2	2	1	0	1	0	1	1	0	0	0	0	1
1	2	2	1	0	0	0	0	1	0	1	0	0	1

LIN-3	MPK-1	LIN-39	LS	LIN-12m	LIN-12i	CKI-1	EFL-1	LIN-35	SCF	APC	CDK-4/CYD-1	CDK-2/CYE-1	CDK-1/CYB-3
2	2	2	0	0	0	0	0	1	0	0	1	0	0
2	2	2	0	1	0	0	0	0	0	0	1	0	0
2	2	2	0	1	0	0	1	0	0	0	1	0	0
2	2	2	0	1	0	0	1	0	0	0	1	1	0

2	2	2	0	1	0	0	1	0	1	0	1	1	0
2	2	2	0	1	0	0	1	0	1	0	0	0	0
2	2	2	0	0	0	0	1	1	0	0	0	0	1
2	2	2	0	0	0	0	0	1	0	1	0	0	1
2	2	2	0	0	0	0	0	1	0	1	0	0	0

LIN-3	MPK-1	LIN-39	LS	LIN-12m	LIN-12i	CKI-1	EFL-1	LIN-35	SCF	APC	CDK-4/CYD-1	CDK-2/CYE-1	CDK-1/CYB-3
2	2	2	1	0	0	0	0	1	0	0	1	0	0
2	2	2	1	1	0	0	0	0	0	0	1	0	0
2	2	2	1	1	1	0	1	0	0	0	1	0	0
2	2	2	1	1	1	0	1	0	0	0	1	1	0
2	2	2	1	1	1	0	1	0	1	0	1	1	0
2	2	2	1	1	1	0	1	0	1	0	0	0	0
2	2	2	1	0	1	0	1	1	0	0	0	0	1
2	2	2	1	0	0	0	0	1	0	1	0	0	1
2	2	2	1	0	0	0	0	1	0	1	0	0	0

LIN-3	MPK-1	LIN-39	LS	LIN-12m	LIN-12i	CKI-1	EFL-1	LIN-35	SCF	APC	CDK-4/CYD-1	CDK-2/CYE-1	CDK-1/CYB-3
3	2	2	0	0	0	0	0	1	0	0	1	0	0
3	2	2	0	1	0	0	0	0	0	0	1	0	0
3	2	2	0	1	0	0	1	0	0	0	1	0	0
3	2	2	0	1	0	0	1	0	0	0	1	1	0
3	2	2	0	1	0	0	1	0	1	0	1	1	0
3	2	2	0	1	0	0	1	0	1	0	0	0	0
3	2	2	0	0	0	0	1	1	0	0	0	0	1
3	2	2	0	0	0	0	0	1	0	1	0	0	1
3	2	2	0	0	0	0	0	1	0	1	0	0	0

LIN-3	MPK-1	LIN-39	LS	LIN-12m	LIN-12i	CKI-1	EFL-1	LIN-35	SCF	APC	CDK-4/CYD-1	CDK-2/CYE-1	CDK-1/CYB-3
3	2	2	1	0	0	0	0	1	0	0	1	0	0
3	2	2	1	1	0	0	0	0	0	0	1	0	0
3	2	2	1	1	1	0	1	0	0	0	1	0	0
3	2	2	1	1	1	0	1	0	0	0	1	1	0
3	2	2	1	1	1	0	1	0	1	0	1	1	0
3	2	2	1	1	1	0	1	0	1	0	0	0	0
3	2	2	1	0	1	0	1	1	0	0	0	0	1
3	2	2	1	0	0	0	0	1	0	1	0	0	1
3	2	2	1	0	0	0	0	1	0	1	0	0	0

LIN-39 0

LIN-3	MPK-1	LIN-39	LS	LIN-12m	LIN-12i	CKI-1	EFL-1	LIN-35	SCF	APC	CDK-4/CYD-1	CDK-2/CYE-1	CDK-1/CYB-3
0	0	0	0	0	0	0	1	0	0	0	1	1	0

LIN-3	MPK-1	LIN-39	LS	LIN-12m	LIN-12i	CKI-1	EFL-1	LIN-35	SCF	APC	CDK-4/CYD-1	CDK-2/CYE-1	CDK-1/CYB-3
0	0	0	0	1	1	0	1	0	0	0	1	1	0

LIN-3	MPK-1	LIN-39	LS	LIN-12m	LIN-12i	CKI-1	EFL-1	LIN-35	SCF	APC	CDK-4/CYD-1	CDK-2/CYE-1	CDK-1/CYB-3
0	0	0	1	0	0	0	1	0	0	0	1	1	0

LIN-3	MPK-1	LIN-39	LS	LIN-12m	LIN-12i	CKI-1	EFL-1	LIN-35	SCF	APC	CDK-4/CYD-1	CDK-2/CYE-1	CDK-1/CYB-3
0	0	0	1	1	1	0	1	0	0	0	1	1	0

LIN-3	MPK-1	LIN-39	LS	LIN-12m	LIN-12i	CKI-1	EFL-1	LIN-35	SCF	APC	CDK-4/CYD-1	CDK-2/CYE-1	CDK-1/CYB-3
1	0	0	0	1	1	0	1	0	0	0	1	1	0

LIN-3	MPK-1	LIN-39	LS	LIN-12m	LIN-12i	CKI-1	EFL-1	LIN-35	SCF	APC	CDK-4/CYD-1	CDK-2/CYE-1	CDK-1/CYB-3
1	0	0	1	1	1	0	1	0	0	0	1	1	0

LIN-3	MPK-1	LIN-39	LS	LIN-12m	LIN-12i	CKI-1	EFL-1	LIN-35	SCF	APC	CDK-4/CYD-1	CDK-2/CYE-1	CDK-1/CYB-3
2	1	0	0	1	1	0	1	0	0	0	1	1	0

LIN-3	MPK-1	LIN-39	LS	LIN-12m	LIN-12i	CKI-1	EFL-1	LIN-35	SCF	APC	CDK-4/CYD-1	CDK-2/CYE-1	CDK-1/CYB-3
2	1	0	1	1	1	0	1	0	0	0	1	1	0

LIN-3	MPK-1	LIN-39	LS	LIN-12m	LIN-12i	CKI-1	EFL-1	LIN-35	SCF	APC	CDK-4/CYD-1	CDK-2/CYE-1	CDK-1/CYB-3
2	2	0	0	0	0	0	1	0	0	0	1	1	0

LIN-3	MPK-1	LIN-39	LS	LIN-12m	LIN-12i	CKI-1	EFL-1	LIN-35	SCF	APC	CDK-4/CYD-1	CDK-2/CYE-1	CDK-1/CYB-3
2	2	0	1	0	0	0	1	0	0	0	1	1	0

LIN-3	MPK-1	LIN-39	LS	LIN-12m	LIN-12i	CKI-1	EFL-1	LIN-35	SCF	APC	CDK-4/CYD-1	CDK-2/CYE-1	CDK-1/CYB-3
3	2	0	0	0	0	0	1	0	0	0	1	1	0

LIN-3	MPK-1	LIN-39	LS	LIN-12m	LIN-12i	CKI-1	EFL-1	LIN-35	SCF	APC	CDK-4/CYD-1	CDK-2/CYE-1	CDK-1/CYB-3
3	2	0	0	1	1	0	1	0	0	0	1	1	0

LIN-3	MPK-1	LIN-39	LS	LIN-12m	LIN-12i	CKI-1	EFL-1	LIN-35	SCF	APC	CDK-4/CYD-1	CDK-2/CYE-1	CDK-1/CYB-3
3	2	0	1	0	0	0	1	0	0	0	1	1	0

LIN-3	MPK-1	LIN-39	LS	LIN-12m	LIN-12i	CKI-1	EFL-1	LIN-35	SCF	APC	CDK-4/CYD-1	CDK-2/CYE-1	CDK-1/CYB-3
3	2	0	1	1	1	0	1	0	0	0	1	1	0

LIN-39 1

LIN-3	MPK-1	LIN-39	LS	LIN-12m	LIN-12i	CKI-1	EFL-1	LIN-35	SCF	APC	CDK-4/CYD-1	CDK-2/CYE-1	CDK-1/CYB-3
2	1	1	1	1	0	0	0	1	0	1	0	0	1

2	2	1	1	1	0	0	0	1	0	1	0	0	0
2	2	1	1	0	1	0	0	1	0	0	1	0	0
2	1	1	1	1	1	0	0	0	0	0	1	0	0
2	1	1	1	1	1	0	1	0	0	0	1	0	0
2	1	1	1	1	1	0	1	0	0	0	1	1	0
2	1	1	1	1	1	0	1	0	1	0	1	1	0
2	1	1	1	1	1	0	1	0	1	0	0	0	0
2	1	1	1	1	1	0	1	1	0	0	0	0	1

LIN-3	MPK-1	LIN-39	LS	LIN-12m	LIN-12i	CKI-1	EFL-1	LIN-35	SCF	APC	CDK-4/CYD-1	CDK-2/CYE-1	CDK-1/CYB-3
2	2	1	0	0	0	0	0	1	0	0	1	0	0
2	2	1	0	1	0	0	0	0	0	0	1	0	0
2	2	1	0	1	0	0	1	0	0	0	1	0	0
2	2	1	0	1	0	0	1	0	0	0	1	1	0
2	2	1	0	1	0	0	1	0	1	0	1	1	0
2	2	1	0	1	0	0	1	0	1	0	0	0	0
2	2	1	0	0	0	0	1	1	0	0	0	0	1
2	2	1	0	0	0	0	0	1	0	1	0	0	1
2	2	1	0	0	0	0	0	1	0	1	0	0	0

LIN-3	MPK-1	LIN-39	LS	LIN-12m	LIN-12i	CKI-1	EFL-1	LIN-35	SCF	APC	CDK-4/CYD-1	CDK-2/CYE-1	CDK-1/CYB-3
3	2	1	0	0	0	0	0	1	0	0	1	0	0
3	2	1	0	1	0	0	0	0	0	0	1	0	0
3	2	1	0	1	0	0	1	0	0	0	1	0	0
3	2	1	0	1	0	0	1	0	0	0	1	1	0
3	2	1	0	1	0	0	1	0	1	0	1	1	0
3	2	1	0	1	0	0	1	0	1	0	0	0	0
3	2	1	0	0	0	0	1	1	0	0	0	0	1
3	2	1	0	0	0	0	0	1	0	1	0	0	1
3	2	1	0	0	0	0	0	1	0	1	0	0	0

LIN-3	MPK-1	LIN-39	LS	LIN-12m	LIN-12i	CKI-1	EFL-1	LIN-35	SCF	APC	CDK-4/CYD-1	CDK-2/CYE-1	CDK-1/CYB-3
3	2	1	1	0	0	0	0	1	0	0	1	0	0
3	2	1	1	1	0	0	0	0	0	0	1	0	0
3	2	1	1	1	1	0	1	0	0	0	1	0	0
3	2	1	1	1	1	0	1	0	0	0	1	1	0
3	2	1	1	1	1	0	1	0	1	0	1	1	0
3	2	1	1	1	1	0	1	0	1	0	0	0	0
3	2	1	1	0	1	0	1	1	0	0	0	0	1
3	2	1	1	0	0	0	0	1	0	1	0	0	1
3	2	1	1	0	0	0	0	1	0	1	0	0	0

LIN-3	MPK-1	LIN-39	LS	LIN-12m	LIN-12i	CKI-1	EFL-1	LIN-35	SCF	APC	CDK-4/CYD-1	CDK-2/CYE-1	CDK-1/CYB-3
1	0	1	0	1	0	1	0	1	0	1	0	0	1
1	1	1	0	1	0	1	0	1	0	1	0	0	0
1	1	1	0	1	1	0	0	1	0	0	0	0	0
1	0	1	0	1	1	0	0	1	0	0	1	0	0
1	0	1	0	1	1	0	0	0	0	0	1	0	0
1	0	1	0	1	1	0	1	0	0	0	1	0	0
1	0	1	0	1	1	0	1	0	0	0	1	1	0
1	0	1	0	1	1	0	1	0	1	0	1	1	0
1	0	1	0	1	1	0	1	0	1	0	0	0	0
1	0	1	0	1	1	0	1	1	0	0	0	0	1

LIN-3	MPK-1	LIN-39	LS	LIN-12m	LIN-12i	CKI-1	EFL-1	LIN-35	SCF	APC	CDK-4/CYD-1	CDK-2/CYE-1	CDK-1/CYB-3
1	0	1	1	1	0	1	0	1	0	1	0	0	1
1	1	1	1	1	0	1	0	1	0	1	0	0	0
1	1	1	1	1	1	0	0	1	0	0	0	0	0
1	0	1	1	1	1	0	0	1	0	0	1	0	0
1	0	1	1	1	1	0	0	0	0	0	1	0	0
1	0	1	1	1	1	0	1	0	0	0	1	0	0
1	0	1	1	1	1	0	1	0	0	0	1	1	0
1	0	1	1	1	1	0	1	0	1	0	1	1	0
1	0	1	1	1	1	0	1	0	1	0	0	0	0
1	0	1	1	1	1	0	1	1	0	0	0	0	1

LIN-3	MPK-1	LIN-39	LS	LIN-12m	LIN-12i	CKI-1	EFL-1	LIN-35	SCF	APC	CDK-4/CYD-1	CDK-2/CYE-1	CDK-1/CYB-3
0	0	1	0	1	0	0	0	0	0	0	1	0	0
0	0	1	0	1	0	0	1	0	0	0	1	0	0
0	0	1	0	1	0	0	1	0	0	0	1	1	0
0	0	1	0	1	0	0	1	0	1	0	1	1	0
0	0	1	0	1	0	0	1	0	1	0	0	0	0
0	0	1	0	1	0	0	1	1	0	0	0	0	1
0	0	1	0	1	0	1	0	1	0	1	0	0	1
0	0	1	0	1	0	1	0	1	0	1	0	0	0
0	0	1	0	1	0	1	0	1	0	0	0	0	0
0	0	1	0	1	0	0	0	1	0	0	0	0	0
0	0	1	0	1	0	0	0	1	0	0	1	0	0

LIN-3	MPK-1	LIN-39	LS	LIN-12m	LIN-12i	CKI-1	EFL-1	LIN-35	SCF	APC	CDK-4/CYD-1	CDK-2/CYE-1	CDK-1/CYB-3
0	0	1	1	1	0	1	0	1	0	1	0	0	0
0	0	1	1	1	1	1	0	1	0	0	0	0	0
0	0	1	1	1	1	0	0	1	0	0	0	0	0
0	0	1	1	1	1	0	0	1	0	0	1	0	0
0	0	1	1	1	1	0	0	0	0	0	1	0	0
0	0	1	1	1	1	0	1	0	0	0	1	0	0
0	0	1	1	1	1	0	1	0	0	0	1	1	0

0	0	1	1	1	1	0	1	0	1	0	1	1	0
0	0	1	1	1	1	0	1	0	1	0	0	0	0
0	0	1	1	1	1	0	1	1	0	0	0	0	1
0	0	1	1	1	0	1	0	1	0	1	0	0	1

LIN-39 2

LIN-3	MPK-1	LIN-39	LS	LIN-12m	LIN-12i	CKI-1	EFL-1	LIN-35	SCF	APC	CDK-4/CYD-1	CDK-2/CYE-1	CDK-1/CYB-3
2	1	2	1	1	0	0	0	1	0	1	0	0	1
2	2	2	1	1	0	0	0	1	0	1	0	0	0
2	2	2	1	0	1	0	0	1	0	0	1	0	0
2	1	2	1	1	1	0	0	0	0	0	1	0	0
2	1	2	1	1	1	0	1	0	0	0	1	0	0
2	1	2	1	1	1	0	1	0	0	0	1	1	0
2	1	2	1	1	1	0	1	0	1	0	1	1	0
2	1	2	1	1	1	0	1	0	1	0	0	0	0
2	1	2	1	1	1	0	1	1	0	0	0	0	1

LIN-3	MPK-1	LIN-39	LS	LIN-12m	LIN-12i	CKI-1	EFL-1	LIN-35	SCF	APC	CDK-4/CYD-1	CDK-2/CYE-1	CDK-1/CYB-3
2	2	2	0	0	0	0	0	1	0	0	1	0	0
2	2	2	0	1	0	0	0	0	0	0	1	0	0
2	2	2	0	1	0	0	1	0	0	0	1	0	0
2	2	2	0	1	0	0	1	0	0	0	1	1	0
2	2	2	0	1	0	0	1	0	1	0	1	1	0
2	2	2	0	1	0	0	1	0	1	0	0	0	0
2	2	2	0	0	0	0	1	1	0	0	0	0	1
2	2	2	0	0	0	0	0	1	0	1	0	0	1
2	2	2	0	0	0	0	0	1	0	1	0	0	0

LIN-3	MPK-1	LIN-39	LS	LIN-12m	LIN-12i	CKI-1	EFL-1	LIN-35	SCF	APC	CDK-4/CYD-1	CDK-2/CYE-1	CDK-1/CYB-3
3	2	2	0	0	0	0	0	1	0	0	1	0	0
3	2	2	0	1	0	0	0	0	0	0	1	0	0
3	2	2	0	1	0	0	1	0	0	0	1	0	0
3	2	2	0	1	0	0	1	0	0	0	1	1	0
3	2	2	0	1	0	0	1	0	1	0	1	1	0
3	2	2	0	1	0	0	1	0	1	0	0	0	0
3	2	2	0	0	0	0	1	1	0	0	0	0	1
3	2	2	0	0	0	0	0	1	0	1	0	0	1
3	2	2	0	0	0	0	0	1	0	1	0	0	0

LIN-3	MPK-1	LIN-39	LS	LIN-12m	LIN-12i	CKI-1	EFL-1	LIN-35	SCF	APC	CDK-4/CYD-1	CDK-2/CYE-1	CDK-1/CYB-3
3	2	2	1	0	0	0	0	1	0	0	1	0	0
3	2	2	1	1	0	0	0	0	0	0	1	0	0
3	2	2	1	1	1	0	1	0	0	0	1	0	0
3	2	2	1	1	1	0	1	0	0	0	1	1	0
3	2	2	1	1	1	0	1	0	1	0	1	1	0
3	2	2	1	1	1	0	1	0	1	0	0	0	0
3	2	2	1	0	1	0	1	1	0	0	0	0	1
3	2	2	1	0	0	0	0	1	0	1	0	0	1
3	2	2	1	0	0	0	0	1	0	1	0	0	0

LIN-3	MPK-1	LIN-39	LS	LIN-12m	LIN-12i	CKI-1	EFL-1	LIN-35	SCF	APC	CDK-4/CYD-1	CDK-2/CYE-1	CDK-1/CYB-3
1	0	2	0	1	0	1	0	1	0	1	0	0	1
1	1	2	0	1	0	1	0	1	0	1	0	0	0
1	1	2	0	1	1	0	0	1	0	0	0	0	0
1	0	2	0	1	1	0	0	1	0	0	1	0	0
1	0	2	0	1	1	0	0	0	0	0	1	0	0
1	0	2	0	1	1	0	1	0	0	0	1	0	0
1	0	2	0	1	1	0	1	0	0	0	1	1	0
1	0	2	0	1	1	0	1	0	1	0	1	1	0
1	0	2	0	1	1	0	1	0	1	0	0	0	0
1	0	2	0	1	1	0	1	1	0	0	0	0	1

LIN-3	MPK-1	LIN-39	LS	LIN-12m	LIN-12i	CKI-1	EFL-1	LIN-35	SCF	APC	CDK-4/CYD-1	CDK-2/CYE-1	CDK-1/CYB-3
1	0	2	1	1	0	1	0	1	0	1	0	0	1
1	1	2	1	1	0	1	0	1	0	1	0	0	0
1	1	2	1	1	1	0	0	1	0	0	0	0	0
1	0	2	1	1	1	0	0	1	0	0	1	0	0
1	0	2	1	1	1	0	0	0	0	0	1	0	0
1	0	2	1	1	1	0	1	0	0	0	1	0	0
1	0	2	1	1	1	0	1	0	0	0	1	1	0
1	0	2	1	1	1	0	1	0	1	0	1	1	0
1	0	2	1	1	1	0	1	0	1	0	0	0	0
1	0	2	1	1	1	0	1	1	0	0	0	0	1

LIN-3	MPK-1	LIN-39	LS	LIN-12m	LIN-12i	CKI-1	EFL-1	LIN-35	SCF	APC	CDK-4/CYD-1	CDK-2/CYE-1	CDK-1/CYB-3
0	0	2	0	1	0	0	0	0	0	0	1	0	0
0	0	2	0	1	0	0	1	0	0	0	1	0	0
0	0	2	0	1	0	0	1	0	0	0	1	1	0
0	0	2	0	1	0	0	1	0	1	0	1	1	0
0	0	2	0	1	0	0	1	0	1	0	0	0	0
0	0	2	0	1	0	0	1	1	0	0	0	0	1
0	0	2	0	1	0	1	0	1	0	1	0	0	1
0	0	2	0	1	0	1	0	1	0	1	0	0	0
0	0	2	0	1	0	1	0	1	0	0	0	0	0
0	0	2	0	1	0	0	0	1	0	0	0	0	0
0	0	2	0	1	0	0	0	1	0	0	1	0	0

LIN-3	MPK-1	LIN-39	LS	LIN-12m	LIN-12i	CKI-1	EFL-1	LIN-35	SCF	APC	CDK-4/CYD-1	CDK-2/CYE-1	CDK-1/CYB-3
0	0	2	1	1	0	1	0	1	0	1	0	0	0
0	0	2	1	1	1	1	0	1	0	0	0	0	0
0	0	2	1	1	1	0	0	1	0	0	0	0	0
0	0	2	1	1	1	0	0	1	0	0	1	0	0
0	0	2	1	1	1	0	0	0	0	0	1	0	0
0	0	2	1	1	1	0	1	0	0	0	1	0	0
0	0	2	1	1	1	0	1	0	0	0	1	1	0
0	0	2	1	1	1	0	1	0	1	0	1	1	0
0	0	2	1	1	1	0	1	0	1	0	0	0	0
0	0	2	1	1	1	0	1	1	0	0	0	0	1
0	0	2	1	1	0	1	0	1	0	1	0	0	1

LS 0

LIN-3	MPK-1	LIN-39	LS	LIN-12m	LIN-12i	CKI-1	EFL-1	LIN-35	SCF	APC	CDK-4/CYD-1	CDK-2/CYE-1	CDK-1/CYB-3
2	2	2	0	0	0	0	0	1	0	0	1	0	0
2	2	2	0	1	0	0	0	0	0	0	1	0	0
2	2	2	0	1	0	0	1	0	0	0	1	0	0
2	2	2	0	1	0	0	1	0	0	0	1	1	0
2	2	2	0	1	0	0	1	0	1	0	1	1	0
2	2	2	0	1	0	0	1	0	1	0	0	0	0
2	2	2	0	0	0	0	1	1	0	0	0	0	1
2	2	2	0	0	0	0	0	1	0	1	0	0	1
2	2	2	0	0	0	0	0	1	0	1	0	0	0

LIN-3	MPK-1	LIN-39	LS	LIN-12m	LIN-12i	CKI-1	EFL-1	LIN-35	SCF	APC	CDK-4/CYD-1	CDK-2/CYE-1	CDK-1/CYB-3
3	2	2	0	0	0	0	0	1	0	0	1	0	0
3	2	2	0	1	0	0	0	0	0	0	1	0	0
3	2	2	0	1	0	0	1	0	0	0	1	0	0
3	2	2	0	1	0	0	1	0	0	0	1	1	0
3	2	2	0	1	0	0	1	0	1	0	1	1	0
3	2	2	0	1	0	0	1	0	1	0	0	0	0
3	2	2	0	0	0	0	1	1	0	0	0	0	1
3	2	2	0	0	0	0	0	1	0	1	0	0	1
3	2	2	0	0	0	0	0	1	0	1	0	0	0

LIN-3	MPK-1	LIN-39	LS	LIN-12m	LIN-12i	CKI-1	EFL-1	LIN-35	SCF	APC	CDK-4/CYD-1	CDK-2/CYE-1	CDK-1/CYB-3
1	0	1	0	1	0	1	0	1	0	1	0	0	1
1	1	1	0	1	0	1	0	1	0	1	0	0	0
1	1	1	0	1	1	0	0	1	0	0	0	0	0
1	0	1	0	1	1	0	0	1	0	0	1	0	0
1	0	1	0	1	1	0	0	0	0	0	1	0	0
1	0	1	0	1	1	0	1	0	0	0	1	0	0
1	0	1	0	1	1	0	1	0	0	0	1	1	0
1	0	1	0	1	1	0	1	0	1	0	1	1	0
1	0	1	0	1	1	0	1	0	1	0	0	0	0
1	0	1	0	1	1	0	1	1	0	0	0	0	1

LIN-3	MPK-1	LIN-39	LS	LIN-12m	LIN-12i	CKI-1	EFL-1	LIN-35	SCF	APC	CDK-4/CYD-1	CDK-2/CYE-1	CDK-1/CYB-3
0	0	1	0	1	0	0	0	0	0	0	1	0	0
0	0	1	0	1	0	0	1	0	0	0	1	0	0
0	0	1	0	1	0	0	1	0	0	0	1	1	0
0	0	1	0	1	0	0	1	0	1	0	1	1	0
0	0	1	0	1	0	0	1	0	1	0	0	0	0
0	0	1	0	1	0	0	1	1	0	0	0	0	1
0	0	1	0	1	0	1	0	1	0	1	0	0	1
0	0	1	0	1	0	1	0	1	0	1	0	0	0
0	0	1	0	1	0	1	0	1	0	0	0	0	0
0	0	1	0	1	0	0	0	1	0	0	0	0	0
0	0	1	0	1	0	0	0	1	0	0	1	0	0

LS 1

LIN-3	MPK-1	LIN-39	LS	LIN-12m	LIN-12i	CKI-1	EFL-1	LIN-35	SCF	APC	CDK-4/CYD-1	CDK-2/CYE-1	CDK-1/CYB-3
2	1	1	1	1	0	0	0	1	0	1	0	0	1
2	2	1	1	1	0	0	0	1	0	1	0	0	0
2	2	2	1	0	1	0	0	1	0	0	1	0	0
2	1	2	1	1	1	0	0	0	0	0	1	0	0
2	1	1	1	1	1	0	1	0	0	0	1	0	0
2	1	1	1	1	1	0	1	0	0	0	1	1	0
2	1	1	1	1	1	0	1	0	1	0	1	1	0
2	1	1	1	1	1	0	1	0	1	0	0	0	0
2	1	1	1	1	1	0	1	1	0	0	0	0	1

LIN-3	MPK-1	LIN-39	LS	LIN-12m	LIN-12i	CKI-1	EFL-1	LIN-35	SCF	APC	CDK-4/CYD-1	CDK-2/CYE-1	CDK-1/CYB-3
3	2	2	1	0	0	0	0	1	0	0	1	0	0
3	2	2	1	1	0	0	0	0	0	0	1	0	0
3	2	2	1	1	1	0	1	0	0	0	1	0	0
3	2	2	1	1	1	0	1	0	0	0	1	1	0
3	2	2	1	1	1	0	1	0	1	0	1	1	0
3	2	2	1	1	1	0	1	0	1	0	0	0	0
3	2	2	1	0	1	0	1	1	0	0	0	0	1
3	2	2	1	0	0	0	0	1	0	1	0	0	1
3	2	2	1	0	0	0	0	1	0	1	0	0	0

LIN-3	MPK-1	LIN-39	LS	LIN-12m	LIN-12i	CKI-1	EFL-1	LIN-35	SCF	APC	CDK-4/CYD-1	CDK-2/CYE-1	CDK-1/CYB-3
1	0	1	1	1	0	1	0	1	0	1	0	0	1
1	1	1	1	1	0	1	0	1	0	1	0	0	0
1	1	1	1	1	1	0	0	1	0	0	0	0	0
1	0	1	1	1	1	0	0	1	0	0	1	0	0
1	0	1	1	1	1	0	0	0	0	0	1	0	0
1	0	1	1	1	1	0	1	0	0	0	1	0	0
1	0	1	1	1	1	0	1	0	0	0	1	1	0
1	0	1	1	1	1	0	1	0	1	0	1	1	0
1	0	1	1	1	1	0	1	0	1	0	0	0	0
1	0	1	1	1	1	0	1	1	0	0	0	0	1

LIN-3	MPK-1	LIN-39	LS	LIN-12m	LIN-12i	CKI-1	EFL-1	LIN-35	SCF	APC	CDK-4/CYD-1	CDK-2/CYE-1	CDK-1/CYB-3
0	0	1	1	1	0	1	0	1	0	1	0	0	0
0	0	1	1	1	1	1	0	1	0	0	0	0	0
0	0	1	1	1	1	0	0	1	0	0	0	0	0
0	0	1	1	1	1	0	0	1	0	0	1	0	0
0	0	1	1	1	1	0	0	0	0	0	1	0	0
0	0	1	1	1	1	0	1	0	0	0	1	0	0
0	0	1	1	1	1	0	1	0	0	0	1	1	0
0	0	1	1	1	1	0	1	0	1	0	1	1	0
0	0	1	1	1	1	0	1	0	1	0	0	0	0
0	0	1	1	1	1	0	1	1	0	0	0	0	1
0	0	1	1	1	0	1	0	1	0	1	0	0	1

LIN-12m 0

LIN-3	MPK-1	LIN-39	LS	LIN-12m	LIN-12i	CKI-1	EFL-1	LIN-35	SCF	APC	CDK-4/CYD-1	CDK-2/CYE-1	CDK-1/CYB-3
2	2	2	0	0	0	0	0	0	0	0	1	0	0
2	2	2	0	0	0	0	1	0	0	0	1	0	0
2	2	2	0	0	0	0	1	0	0	0	1	1	0
2	2	2	0	0	0	0	1	0	1	0	1	1	0
2	2	2	0	0	0	0	1	0	1	0	0	0	0
2	2	2	0	0	0	0	1	1	0	0	0	0	1
2	2	2	0	0	0	0	0	1	0	1	0	0	1
2	2	2	0	0	0	0	0	1	0	1	0	0	0
2	2	2	0	0	0	0	0	1	0	0	1	0	0

LIN-3	MPK-1	LIN-39	LS	LIN-12m	LIN-12i	CKI-1	EFL-1	LIN-35	SCF	APC	CDK-4/CYD-1	CDK-2/CYE-1	CDK-1/CYB-3
2	2	2	1	0	0	0	0	0	0	0	1	0	0
2	2	2	1	0	0	0	1	0	0	0	1	0	0
2	2	2	1	0	0	0	1	0	0	0	1	1	0
2	2	2	1	0	0	0	1	0	1	0	1	1	0
2	2	2	1	0	0	0	1	0	1	0	0	0	0
2	2	2	1	0	0	0	1	1	0	0	0	0	1
2	2	2	1	0	0	0	0	1	0	1	0	0	1
2	2	2	1	0	0	0	0	1	0	1	0	0	0
2	2	2	1	0	0	0	0	1	0	0	1	0	0

LIN-3	MPK-1	LIN-39	LS	LIN-12m	LIN-12i	CKI-1	EFL-1	LIN-35	SCF	APC	CDK-4/CYD-1	CDK-2/CYE-1	CDK-1/CYB-3
3	2	2	0	0	0	0	0	0	0	0	1	0	0
3	2	2	0	0	0	0	1	0	0	0	1	0	0
3	2	2	0	0	0	0	1	0	0	0	1	1	0
3	2	2	0	0	0	0	1	0	1	0	1	1	0
3	2	2	0	0	0	0	1	0	1	0	0	0	0
3	2	2	0	0	0	0	1	1	0	0	0	0	1
3	2	2	0	0	0	0	0	1	0	1	0	0	1
3	2	2	0	0	0	0	0	1	0	1	0	0	0
3	2	2	0	0	0	0	0	1	0	0	1	0	0

LIN-3	MPK-1	LIN-39	LS	LIN-12m	LIN-12i	CKI-1	EFL-1	LIN-35	SCF	APC	CDK-4/CYD-1	CDK-2/CYE-1	CDK-1/CYB-3
3	2	2	1	0	0	0	0	0	0	0	1	0	0
3	2	2	1	0	0	0	1	0	0	0	1	0	0
3	2	2	1	0	0	0	1	0	0	0	1	1	0
3	2	2	1	0	0	0	1	0	1	0	1	1	0
3	2	2	1	0	0	0	1	0	1	0	0	0	0
3	2	2	1	0	0	0	1	1	0	0	0	0	1
3	2	2	1	0	0	0	0	1	0	1	0	0	1
3	2	2	1	0	0	0	0	1	0	1	0	0	0
3	2	2	1	0	0	0	0	1	0	0	1	0	0

LIN-3	MPK-1	LIN-39	LS	LIN-12m	LIN-12i	CKI-1	EFL-1	LIN-35	SCF	APC	CDK-4/CYD-1	CDK-2/CYE-1	CDK-1/CYB-3
1	0	1	0	0	0	1	0	1	0	1	0	0	1
1	1	1	0	0	0	1	0	1	0	1	0	0	0
1	1	1	0	0	1	0	0	1	0	0	0	0	0
1	0	1	0	0	1	0	0	1	0	0	1	0	0
1	0	1	0	0	1	0	0	0	0	0	1	0	0
1	0	1	0	0	1	0	1	0	0	0	1	0	0
1	0	1	0	0	1	0	1	0	0	0	1	1	0
1	0	1	0	0	1	0	1	0	1	0	1	1	0
1	0	1	0	0	1	0	1	0	1	0	0	0	0
1	0	1	0	0	1	0	1	1	0	0	0	0	1

LIN-3	MPK-1	LIN-39	LS	LIN-12m	LIN-12i	CKI-1	EFL-1	LIN-35	SCF	APC	CDK-4/CYD-1	CDK-2/CYE-1	CDK-1/CYB-3
1	0	1	1	0	0	1	0	1	0	1	0	0	1
1	1	1	1	0	0	1	0	1	0	1	0	0	0
1	1	1	1	0	1	0	0	1	0	0	0	0	0

1	0	1	1	0	1	0	0	1	0	0	1	0	0
1	0	1	1	0	1	0	0	0	0	0	1	0	0
1	0	1	1	0	1	0	1	0	0	0	1	0	0
1	0	1	1	0	1	0	1	0	0	0	1	1	0
1	0	1	1	0	1	0	1	0	1	0	1	1	0
1	0	1	1	0	1	0	1	0	1	0	0	0	0
1	0	1	1	0	1	0	1	1	0	0	0	0	1

LIN-3	MPK-1	LIN-39	LS	LIN-12m	LIN-12i	CKI-1	EFL-1	LIN-35	SCF	APC	CDK-4/CYD-1	CDK-2/CYE-1	CDK-1/CYB-3
0	0	1	0	0	0	0	0	0	0	0	1	0	0
0	0	1	0	0	0	0	1	0	0	0	1	0	0
0	0	1	0	0	0	0	1	0	0	0	1	1	0
0	0	1	0	0	0	0	1	0	1	0	1	1	0
0	0	1	0	0	0	0	1	0	1	0	0	0	0
0	0	1	0	0	0	0	1	1	0	0	0	0	1
0	0	1	0	0	0	1	0	1	0	1	0	0	1
0	0	1	0	0	0	1	0	1	0	1	0	0	0
0	0	1	0	0	0	1	0	1	0	0	0	0	0
0	0	1	0	0	0	0	0	1	0	0	0	0	0
0	0	1	0	0	0	0	0	1	0	0	1	0	0

LIN-3	MPK-1	LIN-39	LS	LIN-12m	LIN-12i	CKI-1	EFL-1	LIN-35	SCF	APC	CDK-4/CYD-1	CDK-2/CYE-1	CDK-1/CYB-3
0	0	1	1	0	0	0	0	0	0	0	1	0	0
0	0	1	1	0	0	0	1	0	0	0	1	0	0
0	0	1	1	0	0	0	1	0	0	0	1	1	0
0	0	1	1	0	0	0	1	0	1	0	1	1	0
0	0	1	1	0	0	0	1	0	1	0	0	0	0
0	0	1	1	0	0	0	1	1	0	0	0	0	1
0	0	1	1	0	0	1	0	1	0	1	0	0	1
0	0	1	1	0	0	1	0	1	0	1	0	0	0
0	0	1	1	0	0	1	0	1	0	0	0	0	0
0	0	1	1	0	0	0	0	1	0	0	0	0	0
0	0	1	1	0	0	0	0	1	0	0	1	0	0

LIN-12m 1

LIN-3	MPK-1	LIN-39	LS	LIN-12m	LIN-12i	CKI-1	EFL-1	LIN-35	SCF	APC	CDK-4/CYD-1	CDK-2/CYE-1	CDK-1/CYB-3
2	1	1	1	1	0	0	0	1	0	1	0	0	1
2	2	1	1	1	0	0	0	1	0	1	0	0	0
2	2	2	1	1	1	0	0	1	0	0	1	0	0
2	1	2	1	1	1	0	0	0	0	0	1	0	0
2	1	1	1	1	1	0	1	0	0	0	1	0	0
2	1	1	1	1	1	0	1	0	0	0	1	1	0
2	1	1	1	1	1	0	1	0	1	0	1	1	0
2	1	1	1	1	1	0	1	0	1	0	0	0	0
2	1	1	1	1	1	0	1	1	0	0	0	0	1

LIN-3	MPK-1	LIN-39	LS	LIN-12m	LIN-12i	CKI-1	EFL-1	LIN-35	SCF	APC	CDK-4/CYD-1	CDK-2/CYE-1	CDK-1/CYB-3
2	2	2	0	1	0	0	0	0	0	0	1	0	0
2	2	2	0	1	0	0	1	0	0	0	1	0	0
2	2	2	0	1	0	0	1	0	0	0	1	1	0
2	2	2	0	1	0	0	1	0	1	0	1	1	0
2	2	2	0	1	0	0	1	0	1	0	0	0	0
2	2	2	0	1	0	0	1	1	0	0	0	0	1
2	2	2	0	1	0	0	0	1	0	1	0	0	1
2	2	2	0	1	0	0	0	1	0	1	0	0	0
2	2	2	0	1	0	0	0	1	0	0	1	0	0

LIN-3	MPK-1	LIN-39	LS	LIN-12m	LIN-12i	CKI-1	EFL-1	LIN-35	SCF	APC	CDK-4/CYD-1	CDK-2/CYE-1	CDK-1/CYB-3
3	2	2	0	1	0	0	0	0	0	0	1	0	0
3	2	2	0	1	0	0	1	0	0	0	1	0	0
3	2	2	0	1	0	0	1	0	0	0	1	1	0
3	2	2	0	1	0	0	1	0	1	0	1	1	0
3	2	2	0	1	0	0	1	0	1	0	0	0	0
3	2	2	0	1	0	0	1	1	0	0	0	0	1
3	2	2	0	1	0	0	0	1	0	1	0	0	1
3	2	2	0	1	0	0	0	1	0	1	0	0	0
3	2	2	0	1	0	0	0	1	0	0	1	0	0

LIN-3	MPK-1	LIN-39	LS	LIN-12m	LIN-12i	CKI-1	EFL-1	LIN-35	SCF	APC	CDK-4/CYD-1	CDK-2/CYE-1	CDK-1/CYB-3
3	2	2	1	1	0	0	0	1	0	1	0	0	0
3	2	2	1	1	1	0	0	1	0	0	1	0	0
3	2	2	1	1	1	0	0	0	0	0	1	0	0
3	2	2	1	1	1	0	1	0	0	0	1	0	0
3	2	2	1	1	1	0	1	0	0	0	1	1	0
3	2	2	1	1	1	0	1	0	1	0	1	1	0
3	2	2	1	1	1	0	1	0	1	0	0	0	0
3	2	2	1	1	1	0	1	1	0	0	0	0	1
3	2	2	1	1	0	0	0	1	0	1	0	0	1

LIN-3	MPK-1	LIN-39	LS	LIN-12m	LIN-12i	CKI-1	EFL-1	LIN-35	SCF	APC	CDK-4/CYD-1	CDK-2/CYE-1	CDK-1/CYB-3
1	0	1	0	1	0	1	0	1	0	1	0	0	1
1	1	1	0	1	0	1	0	1	0	1	0	0	0
1	1	1	0	1	1	0	0	1	0	0	0	0	0
1	0	1	0	1	1	0	0	1	0	0	1	0	0
1	0	1	0	1	1	0	0	0	0	0	1	0	0
1	0	1	0	1	1	0	1	0	0	0	1	0	0

1	0	1	0	1	1	0	1	0	0	0	1	1	0
1	0	1	0	1	1	0	1	0	1	0	1	1	0
1	0	1	0	1	1	0	1	0	1	0	0	0	0
1	0	1	0	1	1	0	1	1	0	0	0	0	1

LIN-3	MPK-1	LIN-39	LS	LIN-12m	LIN-12i	CKI-1	EFL-1	LIN-35	SCF	APC	CDK-4/CYD-1	CDK-2/CYE-1	CDK-1/CYB-3
1	0	1	1	1	0	1	0	1	0	1	0	0	1
1	1	1	1	1	0	1	0	1	0	1	0	0	0
1	1	1	1	1	1	0	0	1	0	0	0	0	0
1	0	1	1	1	1	0	0	1	0	0	1	0	0
1	0	1	1	1	1	0	0	0	0	0	1	0	0
1	0	1	1	1	1	0	1	0	0	0	1	0	0
1	0	1	1	1	1	0	1	0	0	0	1	1	0
1	0	1	1	1	1	0	1	0	1	0	1	1	0
1	0	1	1	1	1	0	1	0	1	0	0	0	0
1	0	1	1	1	1	0	1	1	0	0	0	0	1

LIN-3	MPK-1	LIN-39	LS	LIN-12m	LIN-12i	CKI-1	EFL-1	LIN-35	SCF	APC	CDK-4/CYD-1	CDK-2/CYE-1	CDK-1/CYB-3
0	0	1	0	1	0	0	0	0	0	0	1	0	0
0	0	1	0	1	0	0	1	0	0	0	1	0	0
0	0	1	0	1	0	0	1	0	0	0	1	1	0
0	0	1	0	1	0	0	1	0	1	0	1	1	0
0	0	1	0	1	0	0	1	0	1	0	0	0	0
0	0	1	0	1	0	0	1	1	0	0	0	0	1
0	0	1	0	1	0	1	0	1	0	1	0	0	1
0	0	1	0	1	0	1	0	1	0	1	0	0	0
0	0	1	0	1	0	1	0	1	0	0	0	0	0
0	0	1	0	1	0	0	0	1	0	0	0	0	0
0	0	1	0	1	0	0	0	1	0	0	1	0	0

LIN-3	MPK-1	LIN-39	LS	LIN-12m	LIN-12i	CKI-1	EFL-1	LIN-35	SCF	APC	CDK-4/CYD-1	CDK-2/CYE-1	CDK-1/CYB-3
0	0	1	1	1	0	1	0	1	0	1	0	0	0
0	0	1	1	1	1	1	0	1	0	0	0	0	0
0	0	1	1	1	1	0	0	1	0	0	0	0	0
0	0	1	1	1	1	0	0	1	0	0	1	0	0
0	0	1	1	1	1	0	0	0	0	0	1	0	0
0	0	1	1	1	1	0	1	0	0	0	1	0	0
0	0	1	1	1	1	0	1	0	0	0	1	1	0
0	0	1	1	1	1	0	1	0	1	0	1	1	0
0	0	1	1	1	1	0	1	0	1	0	0	0	0
0	0	1	1	1	1	0	1	1	0	0	0	0	1
0	0	1	1	1	0	1	0	1	0	1	0	0	1

LIN-12i 0

LIN-3	MPK-1	LIN-39	LS	LIN-12m	LIN-12i	CKI-1	EFL-1	LIN-35	SCF	APC	CDK-4/CYD-1	CDK-2/CYE-1	CDK-1/CYB-3
1	1	1	0	1	0	0	0	0	0	0	1	0	0
1	1	1	0	1	0	0	1	0	0	0	1	0	0
1	1	1	0	1	0	0	1	0	0	0	1	1	0
1	1	1	0	1	0	0	1	0	1	0	1	1	0
1	1	1	0	1	0	0	1	0	1	0	0	0	0
1	1	1	0	1	0	0	1	1	0	0	0	0	1
1	1	1	0	1	0	0	0	1	0	1	0	0	1
1	1	1	0	1	0	0	0	1	0	1	0	0	0
1	1	1	0	1	0	0	0	1	0	0	1	0	0

LIN-3	MPK-1	LIN-39	LS	LIN-12m	LIN-12i	CKI-1	EFL-1	LIN-35	SCF	APC	CDK-4/CYD-1	CDK-2/CYE-1	CDK-1/CYB-3
1	1	1	1	1	0	0	0	0	0	0	1	0	0
1	1	1	1	1	0	0	1	0	0	0	1	0	0
1	1	1	1	1	0	0	1	0	0	0	1	1	0
1	1	1	1	1	0	0	1	0	1	0	1	1	0
1	1	1	1	1	0	0	1	0	1	0	0	0	0
1	1	1	1	1	0	0	1	1	0	0	0	0	1
1	1	1	1	1	0	0	0	1	0	1	0	0	1
1	1	1	1	1	0	0	0	1	0	1	0	0	0
1	1	1	1	1	0	0	0	1	0	0	1	0	0

LIN-3	MPK-1	LIN-39	LS	LIN-12m	LIN-12i	CKI-1	EFL-1	LIN-35	SCF	APC	CDK-4/CYD-1	CDK-2/CYE-1	CDK-1/CYB-3
2	2	2	0	0	0	0	0	1	0	0	1	0	0
2	2	2	0	1	0	0	0	0	0	0	1	0	0
2	2	2	0	1	0	0	1	0	0	0	1	0	0
2	2	2	0	1	0	0	1	0	0	0	1	1	0
2	2	2	0	1	0	0	1	0	1	0	1	1	0
2	2	2	0	1	0	0	1	0	1	0	0	0	0
2	2	2	0	0	0	0	1	1	0	0	0	0	1
2	2	2	0	0	0	0	0	1	0	1	0	0	1
2	2	2	0	0	0	0	0	1	0	1	0	0	0

LIN-3	MPK-1	LIN-39	LS	LIN-12m	LIN-12i	CKI-1	EFL-1	LIN-35	SCF	APC	CDK-4/CYD-1	CDK-2/CYE-1	CDK-1/CYB-3
2	2	2	1	0	0	0	0	1	0	0	1	0	0
2	2	2	1	1	0	0	0	0	0	0	1	0	0
2	2	2	1	1	0	0	1	0	0	0	1	0	0
2	2	2	1	1	0	0	1	0	0	0	1	1	0
2	2	2	1	1	0	0	1	0	1	0	1	1	0
2	2	2	1	1	0	0	1	0	1	0	0	0	0
2	2	2	1	0	0	0	1	1	0	0	0	0	1
2	2	2	1	0	0	0	0	1	0	1	0	0	1

2	2	2	1	0	0	0	0	1	0	1	0	0	0

LIN-3	MPK-1	LIN-39	LS	LIN-12m	LIN-12i	CKI-1	EFL-1	LIN-35	SCF	APC	CDK-4/CYD-1	CDK-2/CYE-1	CDK-1/CYB-3
3	2	2	0	0	0	0	0	1	0	0	1	0	0
3	2	2	0	1	0	0	0	0	0	0	1	0	0
3	2	2	0	1	0	0	1	0	0	0	1	0	0
3	2	2	0	1	0	0	1	0	0	0	1	1	0
3	2	2	0	1	0	0	1	0	1	0	1	1	0
3	2	2	0	1	0	0	1	0	1	0	0	0	0
3	2	2	0	0	0	0	1	1	0	0	0	0	1
3	2	2	0	0	0	0	0	1	0	1	0	0	1
3	2	2	0	0	0	0	0	1	0	1	0	0	0

LIN-3	MPK-1	LIN-39	LS	LIN-12m	LIN-12i	CKI-1	EFL-1	LIN-35	SCF	APC	CDK-4/CYD-1	CDK-2/CYE-1	CDK-1/CYB-3
3	2	2	1	0	0	0	0	1	0	0	1	0	0
3	2	2	1	1	0	0	0	0	0	0	1	0	0
3	2	2	1	1	0	0	1	0	0	0	1	0	0
3	2	2	1	1	0	0	1	0	0	0	1	1	0
3	2	2	1	1	0	0	1	0	1	0	1	1	0
3	2	2	1	1	0	0	1	0	1	0	0	0	0
3	2	2	1	0	0	0	1	1	0	0	0	0	1
3	2	2	1	0	0	0	0	1	0	1	0	0	1
3	2	2	1	0	0	0	0	1	0	1	0	0	0

LIN-3	MPK-1	LIN-39	LS	LIN-12m	LIN-12i	CKI-1	EFL-1	LIN-35	SCF	APC	CDK-4/CYD-1	CDK-2/CYE-1	CDK-1/CYB-3
0	0	1	0	1	0	0	0	0	0	0	1	0	0
0	0	1	0	1	0	0	1	0	0	0	1	0	0
0	0	1	0	1	0	0	1	0	0	0	1	1	0
0	0	1	0	1	0	0	1	0	1	0	1	1	0
0	0	1	0	1	0	0	1	0	1	0	0	0	0
0	0	1	0	1	0	0	1	1	0	0	0	0	1
0	0	1	0	1	0	1	0	1	0	1	0	0	1
0	0	1	0	1	0	1	0	1	0	1	0	0	0
0	0	1	0	1	0	1	0	1	0	0	0	0	0
0	0	1	0	1	0	0	0	1	0	0	0	0	0
0	0	1	0	1	0	0	0	1	0	0	1	0	0

LIN-3	MPK-1	LIN-39	LS	LIN-12m	LIN-12i	CKI-1	EFL-1	LIN-35	SCF	APC	CDK-4/CYD-1	CDK-2/CYE-1	CDK-1/CYB-3
0	0	1	1	1	0	0	0	0	0	0	1	0	0
0	0	1	1	1	0	0	1	0	0	0	1	0	0
0	0	1	1	1	0	0	1	0	0	0	1	1	0
0	0	1	1	1	0	0	1	0	1	0	1	1	0
0	0	1	1	1	0	0	1	0	1	0	0	0	0
0	0	1	1	1	0	0	1	1	0	0	0	0	1
0	0	1	1	1	0	1	0	1	0	1	0	0	1
0	0	1	1	1	0	1	0	1	0	1	0	0	0
0	0	1	1	1	0	1	0	1	0	0	0	0	0
0	0	1	1	1	0	0	0	1	0	0	0	0	0
0	0	1	1	1	0	0	0	1	0	0	1	0	0

LIN-12i 1

LIN-3	MPK-1	LIN-39	LS	LIN-12m	LIN-12i	CKI-1	EFL-1	LIN-35	SCF	APC	CDK-4/CYD-1	CDK-2/CYE-1	CDK-1/CYB-3
2	1	1	0	1	1	0	0	0	0	0	1	0	0
2	1	1	0	1	1	0	1	0	0	0	1	0	0
2	1	1	0	1	1	0	1	0	0	0	1	1	0
2	1	1	0	1	1	0	1	0	1	0	1	1	0
2	1	1	0	1	1	0	1	0	1	0	0	0	0
2	1	1	0	1	1	0	1	1	0	0	0	0	1
2	1	1	0	1	1	0	0	1	0	1	0	0	1
2	1	1	0	1	1	0	0	1	0	1	0	0	0
2	1	1	0	1	1	0	0	1	0	0	1	0	0

LIN-3	MPK-1	LIN-39	LS	LIN-12m	LIN-12i	CKI-1	EFL-1	LIN-35	SCF	APC	CDK-4/CYD-1	CDK-2/CYE-1	CDK-1/CYB-3
2	1	1	1	1	1	0	0	0	0	0	1	0	0
2	1	1	1	1	1	0	1	0	0	0	1	0	0
2	1	1	1	1	1	0	1	0	0	0	1	1	0
2	1	1	1	1	1	0	1	0	1	0	1	1	0
2	1	1	1	1	1	0	1	0	1	0	0	0	0
2	1	1	1	1	1	0	1	1	0	0	0	0	1
2	1	1	1	1	1	0	0	1	0	1	0	0	1
2	1	1	1	1	1	0	0	1	0	1	0	0	0
2	1	1	1	1	1	0	0	1	0	0	1	0	0

LIN-3	MPK-1	LIN-39	LS	LIN-12m	LIN-12i	CKI-1	EFL-1	LIN-35	SCF	APC	CDK-4/CYD-1	CDK-2/CYE-1	CDK-1/CYB-3
3	2	2	0	0	1	0	0	1	0	0	1	0	0
3	2	2	0	1	1	0	0	0	0	0	1	0	0
3	2	2	0	1	1	0	1	0	0	0	1	0	0
3	2	2	0	1	1	0	1	0	0	0	1	1	0
3	2	2	0	1	1	0	1	0	1	0	1	1	0
3	2	2	0	1	1	0	1	0	1	0	0	0	0
3	2	2	0	0	1	0	1	1	0	0	0	0	1
3	2	2	0	0	1	0	0	1	0	1	0	0	1
3	2	2	0	0	1	0	0	1	0	1	0	0	0

LIN-3	MPK-1	LIN-39	LS	LIN-12m	LIN-12i	CKI-1	EFL-1	LIN-35	SCF	APC	CDK-4/CYD-1	CDK-2/CYE-1	CDK-1/CYB-3
3	2	2	1	0	1	0	0	1	0	0	1	0	0

3	2	2	1	1	1	0	0	0	0	0	1	0	0
3	2	2	1	1	1	0	1	0	0	0	1	0	0
3	2	2	1	1	1	0	1	0	0	0	1	1	0
3	2	2	1	1	1	0	1	0	1	0	1	1	0
3	2	2	1	1	1	0	1	0	1	0	0	0	0
3	2	2	1	0	1	0	1	1	0	0	0	0	1
3	2	2	1	0	1	0	0	1	0	1	0	0	1
3	2	2	1	0	1	0	0	1	0	1	0	0	0

LIN-3	MPK-1	LIN-39	LS	LIN-12m	LIN-12i	CKI-1	EFL-1	LIN-35	SCF	APC	CDK-4/CYD-1	CDK-2/CYE-1	CDK-1/CYB-3
0	0	1	0	1	1	0	0	0	0	0	1	0	0
0	0	1	0	1	1	0	1	0	0	0	1	0	0
0	0	1	0	1	1	0	1	0	0	0	1	1	0
0	0	1	0	1	1	0	1	0	1	0	1	1	0
0	0	1	0	1	1	0	1	0	1	0	0	0	0
0	0	1	0	1	1	0	1	1	0	0	0	0	1
0	0	1	0	1	1	1	0	1	0	1	0	0	1
0	0	1	0	1	1	1	0	1	0	1	0	0	0
0	0	1	0	1	1	1	0	1	0	0	0	0	0
0	0	1	0	1	1	0	0	1	0	0	0	0	0
0	0	1	0	1	1	0	0	1	0	0	1	0	0

LIN-3	MPK-1	LIN-39	LS	LIN-12m	LIN-12i	CKI-1	EFL-1	LIN-35	SCF	APC	CDK-4/CYD-1	CDK-2/CYE-1	CDK-1/CYB-3
0	0	1	1	1	1	0	0	0	0	0	1	0	0
0	0	1	1	1	1	0	1	0	0	0	1	0	0
0	0	1	1	1	1	0	1	0	0	0	1	1	0
0	0	1	1	1	1	0	1	0	1	0	1	1	0
0	0	1	1	1	1	0	1	0	1	0	0	0	0
0	0	1	1	1	1	0	1	1	0	0	0	0	1
0	0	1	1	1	1	1	0	1	0	1	0	0	1
0	0	1	1	1	1	1	0	1	0	1	0	0	0
0	0	1	1	1	1	1	0	1	0	0	0	0	0
0	0	1	1	1	1	0	0	1	0	0	0	0	0
0	0	1	1	1	1	0	0	1	0	0	1	0	0

LIN-3	MPK-1	LIN-39	LS	LIN-12m	LIN-12i	CKI-1	EFL-1	LIN-35	SCF	APC	CDK-4/CYD-1	CDK-2/CYE-1	CDK-1/CYB-3
1	0	1	0	1	1	0	0	0	0	0	1	0	0
1	0	1	0	1	1	0	1	0	0	0	1	0	0
1	0	1	0	1	1	0	1	0	0	0	1	1	0
1	0	1	0	1	1	0	1	0	1	0	1	1	0
1	0	1	0	1	1	0	1	0	1	0	0	0	0
1	0	1	0	1	1	0	1	1	0	0	0	0	1
1	0	1	0	1	1	1	0	1	0	1	0	0	1
1	0	1	0	1	1	1	0	1	0	1	0	0	0
1	0	1	0	1	1	1	0	1	0	0	0	0	0
1	0	1	0	1	1	0	0	1	0	0	0	0	0
1	0	1	0	1	1	0	0	1	0	0	1	0	0

LIN-3	MPK-1	LIN-39	LS	LIN-12m	LIN-12i	CKI-1	EFL-1	LIN-35	SCF	APC	CDK-4/CYD-1	CDK-2/CYE-1	CDK-1/CYB-3
1	0	1	1	1	1	0	0	0	0	0	1	0	0
1	0	1	1	1	1	0	1	0	0	0	1	0	0
1	0	1	1	1	1	0	1	0	0	0	1	1	0
1	0	1	1	1	1	0	1	0	1	0	1	1	0
1	0	1	1	1	1	0	1	0	1	0	0	0	0
1	0	1	1	1	1	0	1	1	0	0	0	0	1
1	0	1	1	1	1	1	0	1	0	1	0	0	1
1	0	1	1	1	1	1	0	1	0	1	0	0	0
1	0	1	1	1	1	1	0	1	0	0	0	0	0
1	0	1	1	1	1	0	0	1	0	0	0	0	0
1	0	1	1	1	1	0	0	1	0	0	1	0	0

CKI-1 0

LIN-3	MPK-1	LIN-39	LS	LIN-12m	LIN-12i	CKI-1	EFL-1	LIN-35	SCF	APC	CDK-4/CYD-1	CDK-2/CYE-1	CDK-1/CYB-3
0	0	1	0	1	0	0	0	0	0	0	1	0	0
0	0	1	0	1	0	0	1	0	0	0	1	0	0
0	0	1	0	1	0	0	1	0	0	0	1	1	0
0	0	1	0	1	0	0	1	0	1	0	1	1	0
0	0	1	0	1	0	0	1	0	1	0	0	0	0
0	0	1	0	1	0	0	1	1	0	0	0	0	1
0	0	1	0	1	0	0	0	1	0	1	0	0	1
0	0	1	0	1	0	0	0	1	0	1	0	0	0
0	0	1	0	1	0	0	0	1	0	0	1	0	0

LIN-3	MPK-1	LIN-39	LS	LIN-12m	LIN-12i	CKI-1	EFL-1	LIN-35	SCF	APC	CDK-4/CYD-1	CDK-2/CYE-1	CDK-1/CYB-3
0	0	1	1	1	0	0	0	1	0	1	0	0	0
0	0	1	1	1	1	0	0	1	0	0	1	0	0
0	0	1	1	1	1	0	0	0	0	0	1	0	0
0	0	1	1	1	1	0	1	0	0	0	1	0	0
0	0	1	1	1	1	0	1	0	0	0	1	1	0
0	0	1	1	1	1	0	1	0	1	0	1	1	0
0	0	1	1	1	1	0	1	0	1	0	0	0	0
0	0	1	1	1	1	0	1	1	0	0	0	0	1
0	0	1	1	1	0	0	0	1	0	1	0	0	1

LIN-3	MPK-1	LIN-39	LS	LIN-12m	LIN-12i	CKI-1	EFL-1	LIN-35	SCF	APC	CDK-4/CYD-1	CDK-2/CYE-1	CDK-1/CYB-3
1	0	1	0	1	0	0	0	1	0	1	0	0	1

1	1	1	0	1	0	0	0	1	0	1	0	0	0
1	1	1	0	1	1	0	0	1	0	0	1	0	0
1	0	1	0	1	1	0	0	0	0	0	1	0	0
1	0	1	0	1	1	0	1	0	0	0	1	0	0
1	0	1	0	1	1	0	1	0	0	0	1	1	0
1	0	1	0	1	1	0	1	0	1	0	1	1	0
1	0	1	0	1	1	0	1	0	1	0	0	0	0
1	0	1	0	1	1	0	1	1	0	0	0	0	1

LIN-3	MPK-1	LIN-39	LS	LIN-12m	LIN-12i	CKI-1	EFL-1	LIN-35	SCF	APC	CDK-4/CYD-1	CDK-2/CYE-1	CDK-1/CYB-3
1	0	1	1	1	0	0	0	1	0	1	0	0	1
1	1	1	1	1	0	0	0	1	0	1	0	0	0
1	1	1	1	1	1	0	0	1	0	0	1	0	0
1	0	1	1	1	1	0	0	0	0	0	1	0	0
1	0	1	1	1	1	0	1	0	0	0	1	0	0
1	0	1	1	1	1	0	1	0	0	0	1	1	0
1	0	1	1	1	1	0	1	0	1	0	1	1	0
1	0	1	1	1	1	0	1	0	1	0	0	0	0
1	0	1	1	1	1	0	1	1	0	0	0	0	1

LIN-3	MPK-1	LIN-39	LS	LIN-12m	LIN-12i	CKI-1	EFL-1	LIN-35	SCF	APC	CDK-4/CYD-1	CDK-2/CYE-1	CDK-1/CYB-3
2	1	1	1	1	0	0	0	1	0	1	0	0	1
2	2	1	1	1	0	0	0	1	0	1	0	0	0
2	2	2	1	0	1	0	0	1	0	0	1	0	0
2	1	2	1	1	1	0	0	0	0	0	1	0	0
2	1	1	1	1	1	0	1	0	0	0	1	0	0
2	1	1	1	1	1	0	1	0	0	0	1	1	0
2	1	1	1	1	1	0	1	0	1	0	1	1	0
2	1	1	1	1	1	0	1	0	1	0	0	0	0
2	1	1	1	1	1	0	1	1	0	0	0	0	1

LIN-3	MPK-1	LIN-39	LS	LIN-12m	LIN-12i	CKI-1	EFL-1	LIN-35	SCF	APC	CDK-4/CYD-1	CDK-2/CYE-1	CDK-1/CYB-3
2	2	2	0	0	0	0	0	1	0	0	1	0	0
2	2	2	0	1	0	0	0	0	0	0	1	0	0
2	2	2	0	1	0	0	1	0	0	0	1	0	0
2	2	2	0	1	0	0	1	0	0	0	1	1	0
2	2	2	0	1	0	0	1	0	1	0	1	1	0
2	2	2	0	1	0	0	1	0	1	0	0	0	0
2	2	2	0	0	0	0	1	1	0	0	0	0	1
2	2	2	0	0	0	0	0	1	0	1	0	0	1
2	2	2	0	0	0	0	0	1	0	1	0	0	0

LIN-3	MPK-1	LIN-39	LS	LIN-12m	LIN-12i	CKI-1	EFL-1	LIN-35	SCF	APC	CDK-4/CYD-1	CDK-2/CYE-1	CDK-1/CYB-3
3	2	2	0	0	0	0	0	1	0	0	1	0	0
3	2	2	0	1	0	0	0	0	0	0	1	0	0
3	2	2	0	1	0	0	1	0	0	0	1	0	0
3	2	2	0	1	0	0	1	0	0	0	1	1	0
3	2	2	0	1	0	0	1	0	1	0	1	1	0
3	2	2	0	1	0	0	1	0	1	0	0	0	0
3	2	2	0	0	0	0	1	1	0	0	0	0	1
3	2	2	0	0	0	0	0	1	0	1	0	0	1
3	2	2	0	0	0	0	0	1	0	1	0	0	0

LIN-3	MPK-1	LIN-39	LS	LIN-12m	LIN-12i	CKI-1	EFL-1	LIN-35	SCF	APC	CDK-4/CYD-1	CDK-2/CYE-1	CDK-1/CYB-3
3	2	2	1	0	0	0	0	1	0	0	1	0	0
3	2	2	1	1	0	0	0	0	0	0	1	0	0
3	2	2	1	1	1	0	1	0	0	0	1	0	0
3	2	2	1	1	1	0	1	0	0	0	1	1	0
3	2	2	1	1	1	0	1	0	1	0	1	1	0
3	2	2	1	1	1	0	1	0	1	0	0	0	0
3	2	2	1	0	1	0	1	1	0	0	0	0	1
3	2	2	1	0	0	0	0	1	0	1	0	0	1
3	2	2	1	0	0	0	0	1	0	1	0	0	0

CKI-1 1

LIN-3	MPK-1	LIN-39	LS	LIN-12m	LIN-12i	CKI-1	EFL-1	LIN-35	SCF	APC	CDK-4/CYD-1	CDK-2/CYE-1	CDK-1/CYB-3
0	0	1	0	1	0	1	0	1	0	0	0	0	0

LIN-3	MPK-1	LIN-39	LS	LIN-12m	LIN-12i	CKI-1	EFL-1	LIN-35	SCF	APC	CDK-4/CYD-1	CDK-2/CYE-1	CDK-1/CYB-3
0	0	1	0	1	1	1	0	1	0	0	0	0	0

LIN-3	MPK-1	LIN-39	LS	LIN-12m	LIN-12i	CKI-1	EFL-1	LIN-35	SCF	APC	CDK-4/CYD-1	CDK-2/CYE-1	CDK-1/CYB-3
0	0	1	1	1	1	1	0	1	0	0	0	0	0

LIN-3	MPK-1	LIN-39	LS	LIN-12m	LIN-12i	CKI-1	EFL-1	LIN-35	SCF	APC	CDK-4/CYD-1	CDK-2/CYE-1	CDK-1/CYB-3
1	0	1	0	1	1	1	0	1	0	0	0	0	0

LIN-3	MPK-1	LIN-39	LS	LIN-12m	LIN-12i	CKI-1	EFL-1	LIN-35	SCF	APC	CDK-4/CYD-1	CDK-2/CYE-1	CDK-1/CYB-3
1	0	1	1	1	1	1	0	1	0	0	0	0	0

LIN-3	MPK-1	LIN-39	LS	LIN-12m	LIN-12i	CKI-1	EFL-1	LIN-35	SCF	APC	CDK-4/CYD-1	CDK-2/CYE-1	CDK-1/CYB-3
2	1	1	0	1	1	1	0	1	0	0	0	0	0

LIN-3	MPK-1	LIN-39	LS	LIN-12m	LIN-12i	CKI-1	EFL-1	LIN-35	SCF	APC	CDK-4/CYD-1	CDK-2/CYE-1	CDK-1/CYB-3
2	1	1	1	1	1	1	0	1	0	0	0	0	0

LIN-3	MPK-1	LIN-39	LS	LIN-12m	LIN-12i	CKI-1	EFL-1	LIN-35	SCF	APC	CDK-4/CYD-1	CDK-2/CYE-1	CDK-1/CYB-3
2	2	2	0	0	0	1	0	1	0	0	0	0	0

LIN-3	MPK-1	LIN-39	LS	LIN-12m	LIN-12i	CKI-1	EFL-1	LIN-35	SCF	APC	CDK-4/CYD-1	CDK-2/CYE-1	CDK-1/CYB-3
2	2	2	1	0	0	1	0	1	0	0	0	0	0

LIN-3	MPK-1	LIN-39	LS	LIN-12m	LIN-12i	CKI-1	EFL-1	LIN-35	SCF	APC	CDK-4/CYD-1	CDK-2/CYE-1	CDK-1/CYB-3
3	2	2	0	0	0	1	0	1	0	0	0	0	0

LIN-3	MPK-1	LIN-39	LS	LIN-12m	LIN-12i	CKI-1	EFL-1	LIN-35	SCF	APC	CDK-4/CYD-1	CDK-2/CYE-1	CDK-1/CYB-3
3	2	2	0	0	1	1	0	1	0	0	0	0	0

LIN-3	MPK-1	LIN-39	LS	LIN-12m	LIN-12i	CKI-1	EFL-1	LIN-35	SCF	APC	CDK-4/CYD-1	CDK-2/CYE-1	CDK-1/CYB-3
3	2	2	1	0	0	1	0	1	0	0	0	0	0

LIN-3	MPK-1	LIN-39	LS	LIN-12m	LIN-12i	CKI-1	EFL-1	LIN-35	SCF	APC	CDK-4/CYD-1	CDK-2/CYE-1	CDK-1/CYB-3
3	2	2	1	0	1	1	0	1	0	0	0	0	0

EFL-1 0

LIN-3	MPK-1	LIN-39	LS	LIN-12m	LIN-12i	CKI-1	EFL-1	LIN-35	SCF	APC	CDK-4/CYD-1	CDK-2/CYE-1	CDK-1/CYB-3
0	0	1	0	1	0	0	0	0	0	0	1	0	0

LIN-3	MPK-1	LIN-39	LS	LIN-12m	LIN-12i	CKI-1	EFL-1	LIN-35	SCF	APC	CDK-4/CYD-1	CDK-2/CYE-1	CDK-1/CYB-3
0	0	1	0	1	1	0	0	0	0	0	1	0	0

LIN-3	MPK-1	LIN-39	LS	LIN-12m	LIN-12i	CKI-1	EFL-1	LIN-35	SCF	APC	CDK-4/CYD-1	CDK-2/CYE-1	CDK-1/CYB-3
0	0	1	1	1	1	0	0	0	0	0	1	0	0

LIN-3	MPK-1	LIN-39	LS	LIN-12m	LIN-12i	CKI-1	EFL-1	LIN-35	SCF	APC	CDK-4/CYD-1	CDK-2/CYE-1	CDK-1/CYB-3
1	0	1	0	1	1	0	0	0	0	0	1	0	0

LIN-3	MPK-1	LIN-39	LS	LIN-12m	LIN-12i	CKI-1	EFL-1	LIN-35	SCF	APC	CDK-4/CYD-1	CDK-2/CYE-1	CDK-1/CYB-3
1	0	1	1	1	1	0	0	0	0	0	1	0	0

LIN-3	MPK-1	LIN-39	LS	LIN-12m	LIN-12i	CKI-1	EFL-1	LIN-35	SCF	APC	CDK-4/CYD-1	CDK-2/CYE-1	CDK-1/CYB-3
2	1	1	0	1	1	0	0	0	0	0	1	0	0

LIN-3	MPK-1	LIN-39	LS	LIN-12m	LIN-12i	CKI-1	EFL-1	LIN-35	SCF	APC	CDK-4/CYD-1	CDK-2/CYE-1	CDK-1/CYB-3
2	1	1	1	1	1	0	0	0	0	0	1	0	0

LIN-3	MPK-1	LIN-39	LS	LIN-12m	LIN-12i	CKI-1	EFL-1	LIN-35	SCF	APC	CDK-4/CYD-1	CDK-2/CYE-1	CDK-1/CYB-3
2	2	2	0	1	0	0	0	0	0	0	1	0	0

LIN-3	MPK-1	LIN-39	LS	LIN-12m	LIN-12i	CKI-1	EFL-1	LIN-35	SCF	APC	CDK-4/CYD-1	CDK-2/CYE-1	CDK-1/CYB-3
3	2	2	0	1	0	0	0	0	0	0	1	0	0

LIN-3	MPK-1	LIN-39	LS	LIN-12m	LIN-12i	CKI-1	EFL-1	LIN-35	SCF	APC	CDK-4/CYD-1	CDK-2/CYE-1	CDK-1/CYB-3
3	2	2	0	1	1	0	0	0	0	0	1	0	0

LIN-3	MPK-1	LIN-39	LS	LIN-12m	LIN-12i	CKI-1	EFL-1	LIN-35	SCF	APC	CDK-4/CYD-1	CDK-2/CYE-1	CDK-1/CYB-3
3	2	2	1	1	1	0	0	0	0	0	1	0	0

EFL-1 1

LIN-3	MPK-1	LIN-39	LS	LIN-12m	LIN-12i	CKI-1	EFL-1	LIN-35	SCF	APC	CDK-4/CYD-1	CDK-2/CYE-1	CDK-1/CYB-3
0	0	1	0	1	0	0	1	0	0	1	0	0	1
0	0	1	0	1	0	1	1	1	0	1	0	1	0
0	0	1	0	1	0	1	1	0	0	0	0	0	0
0	0	1	0	1	0	0	1	1	0	0	0	0	0
0	0	1	0	1	0	0	1	1	0	0	1	0	1

LIN-3	MPK-1	LIN-39	LS	LIN-12m	LIN-12i	CKI-1	EFL-1	LIN-35	SCF	APC	CDK-4/CYD-1	CDK-2/CYE-1	CDK-1/CYB-3
0	0	1	1	1	0	0	1	0	0	1	0	0	1
0	0	1	1	1	0	1	1	1	0	1	0	1	0
0	0	1	1	1	1	1	1	0	0	0	0	0	0
0	0	1	1	1	1	0	1	1	0	0	0	0	0
0	0	1	1	1	1	0	1	1	0	0	1	0	1

LIN-3	MPK-1	LIN-39	LS	LIN-12m	LIN-12i	CKI-1	EFL-1	LIN-35	SCF	APC	CDK-4/CYD-1	CDK-2/CYE-1	CDK-1/CYB-3
1	0	1	0	1	0	0	1	0	0	1	0	0	1
1	1	1	0	1	0	1	1	1	0	1	0	1	0
1	1	1	0	1	1	0	1	0	0	0	0	0	0
1	0	1	0	1	1	0	1	1	0	0	1	1	1
1	0	1	0	1	1	0	1	0	1	1	0	0	1
1	0	1	0	1	0	1	1	1	0	0	0	0	0
1	1	1	0	1	1	0	1	1	0	0	0	0	0
1	0	1	0	1	1	0	1	1	0	0	1	0	1

LIN-3	MPK-1	LIN-39	LS	LIN-12m	LIN-12i	CKI-1	EFL-1	LIN-35	SCF	APC	CDK-4/CYD-1	CDK-2/CYE-1	CDK-1/CYB-3
1	0	1	1	1	0	0	1	0	0	1	0	0	1
1	1	1	1	1	0	1	1	1	0	1	0	1	0
1	1	1	1	1	1	0	1	0	0	0	0	0	0
1	0	1	1	1	1	0	1	1	0	0	1	1	1
1	0	1	1	1	1	0	1	0	1	1	0	0	1
1	0	1	1	1	0	1	1	1	0	0	0	0	0
1	1	1	1	1	1	0	1	1	0	0	0	0	0

Mutations

1	0	1	1	1	1	0	1	1	0	0	1	0	1

LIN-3	MPK-1	LIN-39	LS	LIN-12m	LIN-12i	CKI-1	EFL-1	LIN-35	SCF	APC	CDK-4/CYD-1	CDK-2/CYE-1	CDK-1/CYB-3
2	1	1	1	1	0	0	1	1	0	1	0	0	1
2	2	1	1	1	0	0	1	1	0	1	0	0	0
2	2	2	1	0	1	0	1	1	0	0	1	0	0
2	1	2	1	1	1	0	1	0	0	0	1	0	0
2	1	1	1	1	1	0	1	0	0	0	1	1	0
2	1	1	1	1	1	0	1	0	1	0	1	1	0
2	1	1	1	1	1	0	1	0	1	0	0	0	0
2	1	1	1	1	1	0	1	1	0	0	0	0	1

LIN-3	MPK-1	LIN-39	LS	LIN-12m	LIN-12i	CKI-1	EFL-1	LIN-35	SCF	APC	CDK-4/CYD-1	CDK-2/CYE-1	CDK-1/CYB-3
2	2	2	0	0	0	0	1	1	0	0	0	0	1
2	2	2	0	0	0	0	1	1	0	1	0	0	1
2	2	2	0	0	0	0	1	1	0	1	0	0	0
2	2	2	0	0	0	0	1	1	0	0	1	0	0
2	2	2	0	1	0	0	1	0	0	0	1	0	0
2	2	2	0	1	0	0	1	0	0	0	1	1	0
2	2	2	0	1	0	0	1	0	1	0	1	1	0
2	2	2	0	1	0	0	1	0	1	0	0	0	0

LIN-3	MPK-1	LIN-39	LS	LIN-12m	LIN-12i	CKI-1	EFL-1	LIN-35	SCF	APC	CDK-4/CYD-1	CDK-2/CYE-1	CDK-1/CYB-3
3	2	2	0	0	0	0	1	1	0	0	0	0	1
3	2	2	0	0	0	0	1	1	0	1	0	0	1
3	2	2	0	0	0	0	1	1	0	1	0	0	0
3	2	2	0	0	0	0	1	1	0	0	1	0	0
3	2	2	0	1	0	0	1	0	0	0	1	0	0
3	2	2	0	1	0	0	1	0	0	0	1	1	0
3	2	2	0	1	0	0	1	0	1	0	1	1	0
3	2	2	0	1	0	0	1	0	1	0	0	0	0

LIN-3	MPK-1	LIN-39	LS	LIN-12m	LIN-12i	CKI-1	EFL-1	LIN-35	SCF	APC	CDK-4/CYD-1	CDK-2/CYE-1	CDK-1/CYB-3
3	2	2	1	0	0	0	1	1	0	0	1	0	0
3	2	2	1	1	0	0	1	0	0	0	1	0	0
3	2	2	1	1	1	0	1	0	0	0	1	1	0
3	2	2	1	1	1	0	1	0	1	0	1	1	0
3	2	2	1	1	1	0	1	0	1	0	0	0	0
3	2	2	1	0	1	0	1	1	0	0	0	0	1
3	2	2	1	0	0	0	1	1	0	1	0	0	1
3	2	2	1	0	0	0	1	1	0	1	0	0	0

LIN-35 0

LIN-3	MPK-1	LIN-39	LS	LIN-12m	LIN-12i	CKI-1	EFL-1	LIN-35	SCF	APC	CDK-4/CYD-1	CDK-2/CYE-1	CDK-1/CYB-3
2	1	1	0	1	1	0	1	0	0	0	0	0	0
2	1	1	0	1	1	0	1	0	0	0	1	1	1
2	1	1	0	1	1	0	1	0	1	1	0	1	1

LIN-3	MPK-1	LIN-39	LS	LIN-12m	LIN-12i	CKI-1	EFL-1	LIN-35	SCF	APC	CDK-4/CYD-1	CDK-2/CYE-1	CDK-1/CYB-3
2	1	1	1	1	1	0	1	0	0	0	0	0	0
2	1	1	1	1	1	0	1	0	0	0	1	1	1
2	1	1	1	1	1	0	1	0	1	1	0	1	1

LIN-3	MPK-1	LIN-39	LS	LIN-12m	LIN-12i	CKI-1	EFL-1	LIN-35	SCF	APC	CDK-4/CYD-1	CDK-2/CYE-1	CDK-1/CYB-3
2	2	2	0	0	0	0	1	0	0	0	0	0	0
2	2	2	0	0	0	0	1	0	0	0	1	1	1
2	2	2	0	1	0	0	1	0	1	1	0	1	1

LIN-3	MPK-1	LIN-39	LS	LIN-12m	LIN-12i	CKI-1	EFL-1	LIN-35	SCF	APC	CDK-4/CYD-1	CDK-2/CYE-1	CDK-1/CYB-3
3	2	2	0	0	0	0	1	0	0	0	0	0	0
3	2	2	0	0	0	0	1	0	0	0	1	1	1
3	2	2	0	1	0	0	1	0	1	1	0	1	1

LIN-3	MPK-1	LIN-39	LS	LIN-12m	LIN-12i	CKI-1	EFL-1	LIN-35	SCF	APC	CDK-4/CYD-1	CDK-2/CYE-1	CDK-1/CYB-3
3	2	2	0	0	1	0	1	0	0	0	0	0	0
3	2	2	0	0	1	0	1	0	0	0	1	1	1
3	2	2	0	1	1	0	1	0	1	1	0	1	1

LIN-3	MPK-1	LIN-39	LS	LIN-12m	LIN-12i	CKI-1	EFL-1	LIN-35	SCF	APC	CDK-4/CYD-1	CDK-2/CYE-1	CDK-1/CYB-3
3	2	2	1	0	1	0	1	0	0	0	0	0	0
3	2	2	1	0	1	0	1	0	0	0	1	1	1
3	2	2	1	1	1	0	1	0	1	1	0	1	1

LIN-3	MPK-1	LIN-39	LS	LIN-12m	LIN-12i	CKI-1	EFL-1	LIN-35	SCF	APC	CDK-4/CYD-1	CDK-2/CYE-1	CDK-1/CYB-3
0	0	1	0	1	0	0	1	0	0	0	0	0	0
0	0	1	0	1	0	0	1	0	0	0	1	1	1
0	0	1	0	1	0	0	1	0	1	1	0	1	1
0	0	1	0	1	0	1	1	0	0	0	0	0	0

LIN-3	MPK-1	LIN-39	LS	LIN-12m	LIN-12i	CKI-1	EFL-1	LIN-35	SCF	APC	CDK-4/CYD-1	CDK-2/CYE-1	CDK-1/CYB-3
0	0	1	0	1	1	0	1	0	0	0	0	0	0
0	0	1	0	1	1	0	1	0	0	0	1	1	1
0	0	1	0	1	1	0	1	0	1	1	0	1	1
0	0	1	0	1	1	1	1	0	0	0	0	0	0

LIN-3	MPK-1	LIN-39	LS	LIN-12m	LIN-12i	CKI-1	EFL-1	LIN-35	SCF	APC	CDK-4/CYD-1	CDK-2/CYE-1	CDK-1/CYB-3

0	0	1	1	1	1	0	1	0	0	0	0	0	0
0	0	1	1	1	1	0	1	0	0	0	1	1	1
0	0	1	1	1	1	0	1	0	1	1	0	1	1
0	0	1	1	1	1	1	1	0	0	0	0	0	0

LIN-3	MPK-1	LIN-39	LS	LIN-12m	LIN-12i	CKI-1	EFL-1	LIN-35	SCF	APC	CDK-4/CYD-1	CDK-2/CYE-1	CDK-1/CYB-3
1	0	1	0	1	1	0	1	0	0	0	0	0	0
1	0	1	0	1	1	0	1	0	0	0	1	1	1
1	0	1	0	1	1	0	1	0	1	1	0	1	1
1	0	1	0	1	1	1	1	0	0	0	0	0	0

LIN-3	MPK-1	LIN-39	LS	LIN-12m	LIN-12i	CKI-1	EFL-1	LIN-35	SCF	APC	CDK-4/CYD-1	CDK-2/CYE-1	CDK-1/CYB-3
1	0	1	1	1	1	0	1	0	0	0	0	0	0
1	0	1	1	1	1	0	1	0	0	0	1	1	1
1	0	1	1	1	1	0	1	0	1	1	0	1	1
1	0	1	1	1	1	1	1	0	0	0	0	0	0

LIN-3	MPK-1	LIN-39	LS	LIN-12m	LIN-12i	CKI-1	EFL-1	LIN-35	SCF	APC	CDK-4/CYD-1	CDK-2/CYE-1	CDK-1/CYB-3
2	1	1	1	1	0	0	1	0	0	1	0	1	1
2	2	1	1	1	1	0	1	0	0	1	0	1	0
2	1	2	1	0	1	0	1	0	0	0	1	1	0
2	1	1	1	1	1	0	1	0	1	0	1	1	0
2	1	1	1	1	1	0	1	0	1	0	0	0	0
2	1	1	1	1	1	0	1	0	0	0	0	0	1

LIN-3	MPK-1	LIN-39	LS	LIN-12m	LIN-12i	CKI-1	EFL-1	LIN-35	SCF	APC	CDK-4/CYD-1	CDK-2/CYE-1	CDK-1/CYB-3
2	2	2	0	0	0	0	1	0	0	0	0	0	1
2	2	2	0	0	0	0	1	0	0	1	0	1	1
2	2	2	0	0	0	0	1	0	0	1	0	1	0
2	2	2	0	0	0	0	1	0	0	0	1	1	0
2	2	2	0	1	0	0	1	0	1	0	1	1	0
2	2	2	0	1	0	0	1	0	1	0	0	0	0

LIN-3	MPK-1	LIN-39	LS	LIN-12m	LIN-12i	CKI-1	EFL-1	LIN-35	SCF	APC	CDK-4/CYD-1	CDK-2/CYE-1	CDK-1/CYB-3
3	2	2	0	0	0	0	1	0	0	0	0	0	1
3	2	2	0	0	0	0	1	0	0	1	0	1	1
3	2	2	0	0	0	0	1	0	0	1	0	1	0
3	2	2	0	0	0	0	1	0	0	0	1	1	0
3	2	2	0	1	0	0	1	0	1	0	1	1	0
3	2	2	0	1	0	0	1	0	1	0	0	0	0

LIN-3	MPK-1	LIN-39	LS	LIN-12m	LIN-12i	CKI-1	EFL-1	LIN-35	SCF	APC	CDK-4/CYD-1	CDK-2/CYE-1	CDK-1/CYB-3
3	2	2	1	0	0	0	1	0	0	0	1	1	0
3	2	2	1	1	0	0	1	0	1	0	1	1	0
3	2	2	1	1	1	0	1	0	1	0	0	0	0
3	2	2	1	0	1	0	1	0	0	0	0	0	1
3	2	2	1	0	0	0	1	0	0	1	0	1	1
3	2	2	1	0	0	0	1	0	0	1	0	1	0

LIN-35 1

LIN-3	MPK-1	LIN-39	LS	LIN-12m	LIN-12i	CKI-1	EFL-1	LIN-35	SCF	APC	CDK-4/CYD-1	CDK-2/CYE-1	CDK-1/CYB-3
0	0	1	0	1	0	0	0	1	0	0	1	0	0

LIN-3	MPK-1	LIN-39	LS	LIN-12m	LIN-12i	CKI-1	EFL-1	LIN-35	SCF	APC	CDK-4/CYD-1	CDK-2/CYE-1	CDK-1/CYB-3
0	0	1	0	1	1	0	0	1	0	0	1	0	0

LIN-3	MPK-1	LIN-39	LS	LIN-12m	LIN-12i	CKI-1	EFL-1	LIN-35	SCF	APC	CDK-4/CYD-1	CDK-2/CYE-1	CDK-1/CYB-3
0	0	1	1	1	1	0	0	1	0	0	1	0	0

LIN-3	MPK-1	LIN-39	LS	LIN-12m	LIN-12i	CKI-1	EFL-1	LIN-35	SCF	APC	CDK-4/CYD-1	CDK-2/CYE-1	CDK-1/CYB-3
1	0	1	0	1	1	0	0	1	0	0	1	0	0

LIN-3	MPK-1	LIN-39	LS	LIN-12m	LIN-12i	CKI-1	EFL-1	LIN-35	SCF	APC	CDK-4/CYD-1	CDK-2/CYE-1	CDK-1/CYB-3
1	0	1	1	1	1	0	0	1	0	0	1	0	0

LIN-3	MPK-1	LIN-39	LS	LIN-12m	LIN-12i	CKI-1	EFL-1	LIN-35	SCF	APC	CDK-4/CYD-1	CDK-2/CYE-1	CDK-1/CYB-3
2	1	1	0	1	1	0	0	1	0	0	1	0	0

LIN-3	MPK-1	LIN-39	LS	LIN-12m	LIN-12i	CKI-1	EFL-1	LIN-35	SCF	APC	CDK-4/CYD-1	CDK-2/CYE-1	CDK-1/CYB-3
2	1	1	1	1	1	0	0	1	0	0	1	0	0

LIN-3	MPK-1	LIN-39	LS	LIN-12m	LIN-12i	CKI-1	EFL-1	LIN-35	SCF	APC	CDK-4/CYD-1	CDK-2/CYE-1	CDK-1/CYB-3
2	2	2	0	1	0	0	0	1	0	0	1	0	0

LIN-3	MPK-1	LIN-39	LS	LIN-12m	LIN-12i	CKI-1	EFL-1	LIN-35	SCF	APC	CDK-4/CYD-1	CDK-2/CYE-1	CDK-1/CYB-3
3	2	2	0	1	0	0	0	1	0	0	1	0	0

LIN-3	MPK-1	LIN-39	LS	LIN-12m	LIN-12i	CKI-1	EFL-1	LIN-35	SCF	APC	CDK-4/CYD-1	CDK-2/CYE-1	CDK-1/CYB-3
3	2	2	0	1	1	0	0	1	0	0	1	0	0

LIN-3	MPK-1	LIN-39	LS	LIN-12m	LIN-12i	CKI-1	EFL-1	LIN-35	SCF	APC	CDK-4/CYD-1	CDK-2/CYE-1	CDK-1/CYB-3
3	2	2	1	1	1	0	0	1	0	0	1	0	0

SCF 0

LIN-3	MPK-1	LIN-39	LS	LIN-12m	LIN-12i	CKI-1	EFL-1	LIN-35	SCF	APC	CDK-4/CYD-1	CDK-2/CYE-1	CDK-1/CYB-3

0	0	1	0	1	0	0	1	0	0	0	1	1	0

LIN-3	MPK-1	LIN-39	LS	LIN-12m	LIN-12i	CKI-1	EFL-1	LIN-35	SCF	APC	CDK-4/CYD-1	CDK-2/CYE-1	CDK-1/CYB-3
0	0	1	0	1	1	0	1	0	0	0	1	1	0

LIN-3	MPK-1	LIN-39	LS	LIN-12m	LIN-12i	CKI-1	EFL-1	LIN-35	SCF	APC	CDK-4/CYD-1	CDK-2/CYE-1	CDK-1/CYB-3
0	0	1	1	1	1	0	1	0	0	0	1	1	0

LIN-3	MPK-1	LIN-39	LS	LIN-12m	LIN-12i	CKI-1	EFL-1	LIN-35	SCF	APC	CDK-4/CYD-1	CDK-2/CYE-1	CDK-1/CYB-3
1	0	1	0	1	1	0	1	0	0	0	1	1	0

LIN-3	MPK-1	LIN-39	LS	LIN-12m	LIN-12i	CKI-1	EFL-1	LIN-35	SCF	APC	CDK-4/CYD-1	CDK-2/CYE-1	CDK-1/CYB-3
1	0	1	1	1	1	0	1	0	0	0	1	1	0

LIN-3	MPK-1	LIN-39	LS	LIN-12m	LIN-12i	CKI-1	EFL-1	LIN-35	SCF	APC	CDK-4/CYD-1	CDK-2/CYE-1	CDK-1/CYB-3
2	1	1	0	1	1	0	1	0	0	0	1	1	0

LIN-3	MPK-1	LIN-39	LS	LIN-12m	LIN-12i	CKI-1	EFL-1	LIN-35	SCF	APC	CDK-4/CYD-1	CDK-2/CYE-1	CDK-1/CYB-3
2	1	1	1	1	1	0	1	0	0	0	1	1	0

LIN-3	MPK-1	LIN-39	LS	LIN-12m	LIN-12i	CKI-1	EFL-1	LIN-35	SCF	APC	CDK-4/CYD-1	CDK-2/CYE-1	CDK-1/CYB-3
2	2	2	0	1	0	0	1	0	0	0	1	1	0

LIN-3	MPK-1	LIN-39	LS	LIN-12m	LIN-12i	CKI-1	EFL-1	LIN-35	SCF	APC	CDK-4/CYD-1	CDK-2/CYE-1	CDK-1/CYB-3
3	2	2	0	1	0	0	1	0	0	0	1	1	0

LIN-3	MPK-1	LIN-39	LS	LIN-12m	LIN-12i	CKI-1	EFL-1	LIN-35	SCF	APC	CDK-4/CYD-1	CDK-2/CYE-1	CDK-1/CYB-3
3	2	2	0	1	1	0	1	0	0	0	1	1	0

LIN-3	MPK-1	LIN-39	LS	LIN-12m	LIN-12i	CKI-1	EFL-1	LIN-35	SCF	APC	CDK-4/CYD-1	CDK-2/CYE-1	CDK-1/CYB-3
3	2	2	1	1	1	0	1	0	0	0	1	1	0

SCF 1

LIN-3	MPK-1	LIN-39	LS	LIN-12m	LIN-12i	CKI-1	EFL-1	LIN-35	SCF	APC	CDK-4/CYD-1	CDK-2/CYE-1	CDK-1/CYB-3
0	0	1	0	1	0	0	0	1	1	0	0	0	0

LIN-3	MPK-1	LIN-39	LS	LIN-12m	LIN-12i	CKI-1	EFL-1	LIN-35	SCF	APC	CDK-4/CYD-1	CDK-2/CYE-1	CDK-1/CYB-3
0	0	1	0	1	1	0	0	1	1	0	0	0	0

LIN-3	MPK-1	LIN-39	LS	LIN-12m	LIN-12i	CKI-1	EFL-1	LIN-35	SCF	APC	CDK-4/CYD-1	CDK-2/CYE-1	CDK-1/CYB-3
0	0	1	1	1	1	0	0	1	1	0	0	0	0

LIN-3	MPK-1	LIN-39	LS	LIN-12m	LIN-12i	CKI-1	EFL-1	LIN-35	SCF	APC	CDK-4/CYD-1	CDK-2/CYE-1	CDK-1/CYB-3
1	0	1	0	1	1	0	0	1	1	0	0	0	0

LIN-3	MPK-1	LIN-39	LS	LIN-12m	LIN-12i	CKI-1	EFL-1	LIN-35	SCF	APC	CDK-4/CYD-1	CDK-2/CYE-1	CDK-1/CYB-3
1	0	1	1	1	1	0	0	1	1	0	0	0	0

LIN-3	MPK-1	LIN-39	LS	LIN-12m	LIN-12i	CKI-1	EFL-1	LIN-35	SCF	APC	CDK-4/CYD-1	CDK-2/CYE-1	CDK-1/CYB-3
2	1	1	0	1	1	0	0	1	1	0	0	0	0

LIN-3	MPK-1	LIN-39	LS	LIN-12m	LIN-12i	CKI-1	EFL-1	LIN-35	SCF	APC	CDK-4/CYD-1	CDK-2/CYE-1	CDK-1/CYB-3
2	1	1	1	1	1	0	0	1	1	0	0	0	0

LIN-3	MPK-1	LIN-39	LS	LIN-12m	LIN-12i	CKI-1	EFL-1	LIN-35	SCF	APC	CDK-4/CYD-1	CDK-2/CYE-1	CDK-1/CYB-3
2	2	2	0	0	0	0	0	1	1	0	0	0	0

LIN-3	MPK-1	LIN-39	LS	LIN-12m	LIN-12i	CKI-1	EFL-1	LIN-35	SCF	APC	CDK-4/CYD-1	CDK-2/CYE-1	CDK-1/CYB-3
2	2	2	1	0	0	0	0	1	1	0	0	0	0

LIN-3	MPK-1	LIN-39	LS	LIN-12m	LIN-12i	CKI-1	EFL-1	LIN-35	SCF	APC	CDK-4/CYD-1	CDK-2/CYE-1	CDK-1/CYB-3
3	2	2	0	0	0	0	0	1	1	0	0	0	0

LIN-3	MPK-1	LIN-39	LS	LIN-12m	LIN-12i	CKI-1	EFL-1	LIN-35	SCF	APC	CDK-4/CYD-1	CDK-2/CYE-1	CDK-1/CYB-3
3	2	2	0	0	1	0	0	1	1	0	0	0	0

LIN-3	MPK-1	LIN-39	LS	LIN-12m	LIN-12i	CKI-1	EFL-1	LIN-35	SCF	APC	CDK-4/CYD-1	CDK-2/CYE-1	CDK-1/CYB-3
3	2	2	1	0	0	0	0	1	1	0	0	0	0

LIN-3	MPK-1	LIN-39	LS	LIN-12m	LIN-12i	CKI-1	EFL-1	LIN-35	SCF	APC	CDK-4/CYD-1	CDK-2/CYE-1	CDK-1/CYB-3
3	2	2	1	0	1	0	0	1	1	0	0	0	0

APC 0

LIN-3	MPK-1	LIN-39	LS	LIN-12m	LIN-12i	CKI-1	EFL-1	LIN-35	SCF	APC	CDK-4/CYD-1	CDK-2/CYE-1	CDK-1/CYB-3
2	1	1	1	1	0	0	0	1	0	0	0	0	1
2	2	1	1	1	0	0	0	1	0	0	0	0	0
2	2	2	1	0	1	0	0	1	0	0	1	0	0
2	1	2	1	1	1	0	0	0	0	0	1	0	0
2	1	1	1	1	1	0	1	0	0	0	1	0	0
2	1	1	1	1	1	0	1	0	0	0	1	1	0
2	1	1	1	1	1	0	1	0	1	0	1	1	0
2	1	1	1	1	1	0	1	0	1	0	0	0	0
2	1	1	1	1	1	0	1	1	0	0	0	0	1

LIN-3	MPK-1	LIN-39	LS	LIN-12m	LIN-12i	CKI-1	EFL-1	LIN-35	SCF	APC	CDK-4/CYD-1	CDK-2/CYE-1	CDK-1/CYB-3

2	2	2	0	0	0	0	0	1	0	0	0	0	0
2	2	2	0	0	0	0	0	1	0	0	1	0	0
2	2	2	0	1	0	0	0	0	0	0	1	0	0
2	2	2	0	1	0	0	1	0	0	0	1	0	0
2	2	2	0	1	0	0	1	0	0	0	1	1	0
2	2	2	0	1	0	0	1	0	1	0	1	1	0
2	2	2	0	1	0	0	1	0	1	0	0	0	0
2	2	2	0	0	0	0	1	1	0	0	0	0	1
2	2	2	0	0	0	0	0	1	0	0	0	0	1

LIN-3	MPK-1	LIN-39	LS	LIN-12m	LIN-12i	CKI-1	EFL-1	LIN-35	SCF	APC	CDK-4/CYD-1	CDK-2/CYE-1	CDK-1/CYB-3
3	2	2	0	0	0	0	0	1	0	0	0	0	0
3	2	2	0	0	0	0	0	1	0	0	1	0	0
3	2	2	0	1	0	0	0	0	0	0	1	0	0
3	2	2	0	1	0	0	1	0	0	0	1	0	0
3	2	2	0	1	0	0	1	0	0	0	1	1	0
3	2	2	0	1	0	0	1	0	1	0	1	1	0
3	2	2	0	1	0	0	1	0	1	0	0	0	0
3	2	2	0	0	0	0	1	1	0	0	0	0	1
3	2	2	0	0	0	0	0	1	0	0	0	0	1

LIN-3	MPK-1	LIN-39	LS	LIN-12m	LIN-12i	CKI-1	EFL-1	LIN-35	SCF	APC	CDK-4/CYD-1	CDK-2/CYE-1	CDK-1/CYB-3
3	2	2	1	0	0	0	0	1	0	0	0	0	0
3	2	2	1	0	0	0	0	1	0	0	1	0	0
3	2	2	1	1	0	0	0	0	0	0	1	0	0
3	2	2	1	1	1	0	1	0	0	0	1	0	0
3	2	2	1	1	1	0	1	0	0	0	1	1	0
3	2	2	1	1	1	0	1	0	1	0	1	1	0
3	2	2	1	1	1	0	1	0	1	0	0	0	0
3	2	2	1	0	1	0	1	1	0	0	0	0	1
3	2	2	1	0	0	0	0	1	0	0	0	0	1

LIN-3	MPK-1	LIN-39	LS	LIN-12m	LIN-12i	CKI-1	EFL-1	LIN-35	SCF	APC	CDK-4/CYD-1	CDK-2/CYE-1	CDK-1/CYB-3
0	0	1	0	1	0	0	0	0	0	0	1	0	0
0	0	1	0	1	0	0	1	0	0	0	1	0	0
0	0	1	0	1	0	0	1	0	0	0	1	1	0
0	0	1	0	1	0	0	1	0	1	0	1	1	0
0	0	1	0	1	0	0	1	0	1	0	0	0	0
0	0	1	0	1	0	0	1	1	0	0	0	0	1
0	0	1	0	1	0	1	0	1	0	0	0	0	1
0	0	1	0	1	0	1	0	1	0	0	0	0	0
0	0	1	0	1	0	0	0	1	0	0	0	0	0
0	0	1	0	1	0	0	0	1	0	0	1	0	0

LIN-3	MPK-1	LIN-39	LS	LIN-12m	LIN-12i	CKI-1	EFL-1	LIN-35	SCF	APC	CDK-4/CYD-1	CDK-2/CYE-1	CDK-1/CYB-3
0	0	1	1	1	0	1	0	1	0	0	0	0	0
0	0	1	1	1	1	0	0	1	0	0	0	0	0
0	0	1	1	1	1	0	0	1	0	0	1	0	0
0	0	1	1	1	1	0	0	0	0	0	1	0	0
0	0	1	1	1	1	0	1	0	0	0	1	0	0
0	0	1	1	1	1	0	1	0	0	0	1	1	0
0	0	1	1	1	1	0	1	0	1	0	1	1	0
0	0	1	1	1	1	0	1	0	1	0	0	0	0
0	0	1	1	1	1	0	1	1	0	0	0	0	1
0	0	1	1	1	0	1	0	1	0	0	0	0	1

LIN-3	MPK-1	LIN-39	LS	LIN-12m	LIN-12i	CKI-1	EFL-1	LIN-35	SCF	APC	CDK-4/CYD-1	CDK-2/CYE-1	CDK-1/CYB-3
1	0	1	0	1	0	1	0	1	0	0	0	0	1
1	1	1	0	1	0	1	0	1	0	0	0	0	0
1	1	1	0	1	1	0	0	1	0	0	0	0	0
1	0	1	0	1	1	0	0	1	0	0	1	0	0
1	0	1	0	1	1	0	0	0	0	0	1	0	0
1	0	1	0	1	1	0	1	0	0	0	1	0	0
1	0	1	0	1	1	0	1	0	0	0	1	1	0
1	0	1	0	1	1	0	1	0	1	0	1	1	0
1	0	1	0	1	1	0	1	0	1	0	0	0	0
1	0	1	0	1	1	0	1	1	0	0	0	0	1

LIN-3	MPK-1	LIN-39	LS	LIN-12m	LIN-12i	CKI-1	EFL-1	LIN-35	SCF	APC	CDK-4/CYD-1	CDK-2/CYE-1	CDK-1/CYB-3
1	0	1	1	1	0	1	0	1	0	0	0	0	1
1	1	1	1	1	0	1	0	1	0	0	0	0	0
1	1	1	1	1	1	0	0	1	0	0	0	0	0
1	0	1	1	1	1	0	0	1	0	0	1	0	0
1	0	1	1	1	1	0	0	0	0	0	1	0	0
1	0	1	1	1	1	0	1	0	0	0	1	0	0
1	0	1	1	1	1	0	1	0	0	0	1	1	0
1	0	1	1	1	1	0	1	0	1	0	1	1	0
1	0	1	1	1	1	0	1	0	1	0	0	0	0
1	0	1	1	1	1	0	1	1	0	0	0	0	1

APC 1

LIN-3	MPK-1	LIN-39	LS	LIN-12m	LIN-12i	CKI-1	EFL-1	LIN-35	SCF	APC	CDK-4/CYD-1	CDK-2/CYE-1	CDK-1/CYB-3
0	0	1	0	1	0	0	1	0	0	1	1	1	0

LIN-3	MPK-1	LIN-39	LS	LIN-12m	LIN-12i	CKI-1	EFL-1	LIN-35	SCF	APC	CDK-4/CYD-1	CDK-2/CYE-1	CDK-1/CYB-3
0	0	1	0	1	0	1	0	1	0	1	0	0	0

LIN-3	MPK-1	LIN-39	LS	LIN-12m	LIN-12i	CKI-1	EFL-1	LIN-35	SCF	APC	CDK-4/CYD-1	CDK-2/CYE-1	CDK-1/CYB-3
0	0	1	0	1	1	0	1	0	0	1	1	1	0

LIN-3	MPK-1	LIN-39	LS	LIN-12m	LIN-12i	CKI-1	EFL-1	LIN-35	SCF	APC	CDK-4/CYD-1	CDK-2/CYE-1	CDK-1/CYB-3
0	0	1	0	1	1	1	0	1	0	1	0	0	0

LIN-3	MPK-1	LIN-39	LS	LIN-12m	LIN-12i	CKI-1	EFL-1	LIN-35	SCF	APC	CDK-4/CYD-1	CDK-2/CYE-1	CDK-1/CYB-3
0	0	1	1	1	1	0	1	0	0	1	1	1	0

LIN-3	MPK-1	LIN-39	LS	LIN-12m	LIN-12i	CKI-1	EFL-1	LIN-35	SCF	APC	CDK-4/CYD-1	CDK-2/CYE-1	CDK-1/CYB-3
0	0	1	1	1	1	1	0	1	0	1	0	0	0

LIN-3	MPK-1	LIN-39	LS	LIN-12m	LIN-12i	CKI-1	EFL-1	LIN-35	SCF	APC	CDK-4/CYD-1	CDK-2/CYE-1	CDK-1/CYB-3
1	0	1	0	1	1	0	1	0	0	1	1	1	0

LIN-3	MPK-1	LIN-39	LS	LIN-12m	LIN-12i	CKI-1	EFL-1	LIN-35	SCF	APC	CDK-4/CYD-1	CDK-2/CYE-1	CDK-1/CYB-3
1	0	1	0	1	1	1	0	1	0	1	0	0	0

LIN-3	MPK-1	LIN-39	LS	LIN-12m	LIN-12i	CKI-1	EFL-1	LIN-35	SCF	APC	CDK-4/CYD-1	CDK-2/CYE-1	CDK-1/CYB-3
1	0	1	1	1	1	0	1	0	0	1	1	1	0

LIN-3	MPK-1	LIN-39	LS	LIN-12m	LIN-12i	CKI-1	EFL-1	LIN-35	SCF	APC	CDK-4/CYD-1	CDK-2/CYE-1	CDK-1/CYB-3
1	0	1	1	1	1	1	0	1	0	1	0	0	0

LIN-3	MPK-1	LIN-39	LS	LIN-12m	LIN-12i	CKI-1	EFL-1	LIN-35	SCF	APC	CDK-4/CYD-1	CDK-2/CYE-1	CDK-1/CYB-3
2	1	1	0	1	1	0	1	0	0	1	1	1	0

LIN-3	MPK-1	LIN-39	LS	LIN-12m	LIN-12i	CKI-1	EFL-1	LIN-35	SCF	APC	CDK-4/CYD-1	CDK-2/CYE-1	CDK-1/CYB-3
2	1	1	1	1	1	0	1	0	0	1	1	1	0

LIN-3	MPK-1	LIN-39	LS	LIN-12m	LIN-12i	CKI-1	EFL-1	LIN-35	SCF	APC	CDK-4/CYD-1	CDK-2/CYE-1	CDK-1/CYB-3
2	2	2	0	1	0	0	1	0	0	1	1	1	0

LIN-3	MPK-1	LIN-39	LS	LIN-12m	LIN-12i	CKI-1	EFL-1	LIN-35	SCF	APC	CDK-4/CYD-1	CDK-2/CYE-1	CDK-1/CYB-3
3	2	2	0	1	0	0	1	0	0	1	1	1	0

LIN-3	MPK-1	LIN-39	LS	LIN-12m	LIN-12i	CKI-1	EFL-1	LIN-35	SCF	APC	CDK-4/CYD-1	CDK-2/CYE-1	CDK-1/CYB-3
3	2	2	0	1	1	0	1	0	0	1	1	1	0

LIN-3	MPK-1	LIN-39	LS	LIN-12m	LIN-12i	CKI-1	EFL-1	LIN-35	SCF	APC	CDK-4/CYD-1	CDK-2/CYE-1	CDK-1/CYB-3
3	2	2	1	1	1	0	1	0	0	1	1	1	0

LIN-3	MPK-1	LIN-39	LS	LIN-12m	LIN-12i	CKI-1	EFL-1	LIN-35	SCF	APC	CDK-4/CYD-1	CDK-2/CYE-1	CDK-1/CYB-3
0	0	1	0	1	0	0	0	0	0	1	0	0	0
0	0	1	0	1	0	1	1	1	0	1	1	0	0

LIN-3	MPK-1	LIN-39	LS	LIN-12m	LIN-12i	CKI-1	EFL-1	LIN-35	SCF	APC	CDK-4/CYD-1	CDK-2/CYE-1	CDK-1/CYB-3
0	0	1	0	1	1	0	0	0	0	1	0	0	0
0	0	1	0	1	1	1	1	1	0	1	1	0	0

LIN-3	MPK-1	LIN-39	LS	LIN-12m	LIN-12i	CKI-1	EFL-1	LIN-35	SCF	APC	CDK-4/CYD-1	CDK-2/CYE-1	CDK-1/CYB-3
0	0	1	1	1	1	0	0	0	0	1	0	0	0
0	0	1	1	1	1	1	1	1	0	1	1	0	0

LIN-3	MPK-1	LIN-39	LS	LIN-12m	LIN-12i	CKI-1	EFL-1	LIN-35	SCF	APC	CDK-4/CYD-1	CDK-2/CYE-1	CDK-1/CYB-3
1	0	1	0	1	1	0	0	0	0	1	0	0	0
1	0	1	0	1	1	1	1	1	0	1	1	0	0

LIN-3	MPK-1	LIN-39	LS	LIN-12m	LIN-12i	CKI-1	EFL-1	LIN-35	SCF	APC	CDK-4/CYD-1	CDK-2/CYE-1	CDK-1/CYB-3
1	0	1	1	1	1	0	0	0	0	1	0	0	0
1	0	1	1	1	1	1	1	1	0	1	1	0	0

CDK-4/CYD-1 0

LIN-3	MPK-1	LIN-39	LS	LIN-12m	LIN-12i	CKI-1	EFL-1	LIN-35	SCF	APC	CDK-4/CYD-1	CDK-2/CYE-1	CDK-1/CYB-3
0	0	1	0	1	0	0	0	1	0	0	0	0	0

LIN-3	MPK-1	LIN-39	LS	LIN-12m	LIN-12i	CKI-1	EFL-1	LIN-35	SCF	APC	CDK-4/CYD-1	CDK-2/CYE-1	CDK-1/CYB-3
0	0	1	0	1	1	0	0	1	0	0	0	0	0

LIN-3	MPK-1	LIN-39	LS	LIN-12m	LIN-12i	CKI-1	EFL-1	LIN-35	SCF	APC	CDK-4/CYD-1	CDK-2/CYE-1	CDK-1/CYB-3
0	0	1	1	1	1	0	0	1	0	0	0	0	0

LIN-3	MPK-1	LIN-39	LS	LIN-12m	LIN-12i	CKI-1	EFL-1	LIN-35	SCF	APC	CDK-4/CYD-1	CDK-2/CYE-1	CDK-1/CYB-3
1	0	1	0	1	1	0	0	1	0	0	0	0	0

LIN-3	MPK-1	LIN-39	LS	LIN-12m	LIN-12i	CKI-1	EFL-1	LIN-35	SCF	APC	CDK-4/CYD-1	CDK-2/CYE-1	CDK-1/CYB-3
1	0	1	1	1	1	0	0	1	0	0	0	0	0

LIN-3	MPK-1	LIN-39	LS	LIN-12m	LIN-12i	CKI-1	EFL-1	LIN-35	SCF	APC	CDK-4/CYD-1	CDK-2/CYE-1	CDK-1/CYB-3
2	1	1	0	1	1	0	0	1	0	0	0	0	0

LIN-3	MPK-1	LIN-39	LS	LIN-12m	LIN-12i	CKI-1	EFL-1	LIN-35	SCF	APC	CDK-4/CYD-1	CDK-2/CYE-1	CDK-1/CYB-3
2	1	1	1	1	1	0	0	1	0	0	0	0	0

LIN-3	MPK-1	LIN-39	LS	LIN-12m	LIN-12i	CKI-1	EFL-1	LIN-35	SCF	APC	CDK-4/CYD-1	CDK-2/CYE-1	CDK-1/CYB-3

2	2	2	0	0	0	0	0	1	0	0	0	0	0

LIN-3	MPK-1	LIN-39	LS	LIN-12m	LIN-12i	CKI-1	EFL-1	LIN-35	SCF	APC	CDK-4/CYD-1	CDK-2/CYE-1	CDK-1/CYB-3
2	2	2	1	0	0	0	0	1	0	0	0	0	0

LIN-3	MPK-1	LIN-39	LS	LIN-12m	LIN-12i	CKI-1	EFL-1	LIN-35	SCF	APC	CDK-4/CYD-1	CDK-2/CYE-1	CDK-1/CYB-3
3	2	2	0	0	0	0	0	1	0	0	0	0	0

LIN-3	MPK-1	LIN-39	LS	LIN-12m	LIN-12i	CKI-1	EFL-1	LIN-35	SCF	APC	CDK-4/CYD-1	CDK-2/CYE-1	CDK-1/CYB-3
3	2	2	0	0	1	0	0	1	0	0	0	0	0

LIN-3	MPK-1	LIN-39	LS	LIN-12m	LIN-12i	CKI-1	EFL-1	LIN-35	SCF	APC	CDK-4/CYD-1	CDK-2/CYE-1	CDK-1/CYB-3
3	2	2	1	0	0	0	0	1	0	0	0	0	0

LIN-3	MPK-1	LIN-39	LS	LIN-12m	LIN-12i	CKI-1	EFL-1	LIN-35	SCF	APC	CDK-4/CYD-1	CDK-2/CYE-1	CDK-1/CYB-3
3	2	2	1	0	1	0	0	1	0	0	0	0	0

CDK-4/CYD-1 1

LIN-3	MPK-1	LIN-39	LS	LIN-12m	LIN-12i	CKI-1	EFL-1	LIN-35	SCF	APC	CDK-4/CYD-1	CDK-2/CYE-1	CDK-1/CYB-3
0	0	1	0	1	0	0	1	0	0	0	1	0	0
0	0	1	0	1	0	0	1	0	0	0	1	1	0
0	0	1	0	1	0	0	1	0	1	0	1	1	0
0	0	1	0	1	0	0	1	0	1	0	1	0	0

LIN-3	MPK-1	LIN-39	LS	LIN-12m	LIN-12i	CKI-1	EFL-1	LIN-35	SCF	APC	CDK-4/CYD-1	CDK-2/CYE-1	CDK-1/CYB-3
0	0	1	0	1	1	0	1	0	0	0	1	0	0
0	0	1	0	1	1	0	1	0	0	0	1	1	0
0	0	1	0	1	1	0	1	0	1	0	1	1	0
0	0	1	0	1	1	0	1	0	1	0	1	0	0

LIN-3	MPK-1	LIN-39	LS	LIN-12m	LIN-12i	CKI-1	EFL-1	LIN-35	SCF	APC	CDK-4/CYD-1	CDK-2/CYE-1	CDK-1/CYB-3
0	0	1	1	1	1	0	1	0	0	0	1	0	0
0	0	1	1	1	1	0	1	0	0	0	1	1	0
0	0	1	1	1	1	0	1	0	1	0	1	1	0
0	0	1	1	1	1	0	1	0	1	0	1	0	0

LIN-3	MPK-1	LIN-39	LS	LIN-12m	LIN-12i	CKI-1	EFL-1	LIN-35	SCF	APC	CDK-4/CYD-1	CDK-2/CYE-1	CDK-1/CYB-3
1	0	1	0	1	1	0	1	0	0	0	1	0	0
1	0	1	0	1	1	0	1	0	0	0	1	1	0
1	0	1	0	1	1	0	1	0	1	0	1	1	0
1	0	1	0	1	1	0	1	0	1	0	1	0	0

LIN-3	MPK-1	LIN-39	LS	LIN-12m	LIN-12i	CKI-1	EFL-1	LIN-35	SCF	APC	CDK-4/CYD-1	CDK-2/CYE-1	CDK-1/CYB-3
1	0	1	1	1	1	0	1	0	0	0	1	0	0
1	0	1	1	1	1	0	1	0	0	0	1	1	0
1	0	1	1	1	1	0	1	0	1	0	1	1	0
1	0	1	1	1	1	0	1	0	1	0	1	0	0

LIN-3	MPK-1	LIN-39	LS	LIN-12m	LIN-12i	CKI-1	EFL-1	LIN-35	SCF	APC	CDK-4/CYD-1	CDK-2/CYE-1	CDK-1/CYB-3
2	1	1	0	1	1	0	1	0	0	0	1	0	0
2	1	1	0	1	1	0	1	0	0	0	1	1	0
2	1	1	0	1	1	0	1	0	1	0	1	1	0
2	1	1	0	1	1	0	1	0	1	0	1	0	0

LIN-3	MPK-1	LIN-39	LS	LIN-12m	LIN-12i	CKI-1	EFL-1	LIN-35	SCF	APC	CDK-4/CYD-1	CDK-2/CYE-1	CDK-1/CYB-3
2	1	1	1	1	1	0	1	0	0	0	1	0	0
2	1	1	1	1	1	0	1	0	0	0	1	1	0
2	1	1	1	1	1	0	1	0	1	0	1	1	0
2	1	1	1	1	1	0	1	0	1	0	1	0	0

LIN-3	MPK-1	LIN-39	LS	LIN-12m	LIN-12i	CKI-1	EFL-1	LIN-35	SCF	APC	CDK-4/CYD-1	CDK-2/CYE-1	CDK-1/CYB-3
2	2	2	0	1	0	0	1	0	0	0	1	0	0
2	2	2	0	1	0	0	1	0	0	0	1	1	0
2	2	2	0	1	0	0	1	0	1	0	1	1	0
2	2	2	0	1	0	0	1	0	1	0	1	0	0

LIN-3	MPK-1	LIN-39	LS	LIN-12m	LIN-12i	CKI-1	EFL-1	LIN-35	SCF	APC	CDK-4/CYD-1	CDK-2/CYE-1	CDK-1/CYB-3
3	2	2	0	1	0	0	1	0	0	0	1	0	0
3	2	2	0	1	0	0	1	0	0	0	1	1	0
3	2	2	0	1	0	0	1	0	1	0	1	1	0
3	2	2	0	1	0	0	1	0	1	0	1	0	0

LIN-3	MPK-1	LIN-39	LS	LIN-12m	LIN-12i	CKI-1	EFL-1	LIN-35	SCF	APC	CDK-4/CYD-1	CDK-2/CYE-1	CDK-1/CYB-3
3	2	2	0	1	1	0	1	0	0	0	1	0	0
3	2	2	0	1	1	0	1	0	0	0	1	1	0
3	2	2	0	1	1	0	1	0	1	0	1	1	0
3	2	2	0	1	1	0	1	0	1	0	1	0	0

LIN-3	MPK-1	LIN-39	LS	LIN-12m	LIN-12i	CKI-1	EFL-1	LIN-35	SCF	APC	CDK-4/CYD-1	CDK-2/CYE-1	CDK-1/CYB-3
3	2	2	1	1	1	0	1	0	0	0	1	0	0
3	2	2	1	1	1	0	1	0	0	0	1	1	0
3	2	2	1	1	1	0	1	0	1	0	1	1	0
3	2	2	1	1	1	0	1	0	1	0	1	0	0

CDK-2/CYE-1 0

Mutations

LIN-3	MPK-1	LIN-39	LS	LIN-12m	LIN-12i	CKI-1	EFL-1	LIN-35	SCF	APC	CDK-4/CYD-1	CDK-2/CYE-1	CDK-1/CYB-3
0	0	1	0	1	0	0	1	0	0	0	1	0	0

LIN-3	MPK-1	LIN-39	LS	LIN-12m	LIN-12i	CKI-1	EFL-1	LIN-35	SCF	APC	CDK-4/CYD-1	CDK-2/CYE-1	CDK-1/CYB-3
0	0	1	0	1	1	0	1	0	0	0	1	0	0

LIN-3	MPK-1	LIN-39	LS	LIN-12m	LIN-12i	CKI-1	EFL-1	LIN-35	SCF	APC	CDK-4/CYD-1	CDK-2/CYE-1	CDK-1/CYB-3
0	0	1	1	1	1	0	1	0	0	0	1	0	0

LIN-3	MPK-1	LIN-39	LS	LIN-12m	LIN-12i	CKI-1	EFL-1	LIN-35	SCF	APC	CDK-4/CYD-1	CDK-2/CYE-1	CDK-1/CYB-3
1	0	1	0	1	1	0	1	0	0	0	1	0	0

LIN-3	MPK-1	LIN-39	LS	LIN-12m	LIN-12i	CKI-1	EFL-1	LIN-35	SCF	APC	CDK-4/CYD-1	CDK-2/CYE-1	CDK-1/CYB-3
1	0	1	1	1	1	0	1	0	0	0	1	0	0

LIN-3	MPK-1	LIN-39	LS	LIN-12m	LIN-12i	CKI-1	EFL-1	LIN-35	SCF	APC	CDK-4/CYD-1	CDK-2/CYE-1	CDK-1/CYB-3
2	1	1	0	1	1	0	1	0	0	0	1	0	0

LIN-3	MPK-1	LIN-39	LS	LIN-12m	LIN-12i	CKI-1	EFL-1	LIN-35	SCF	APC	CDK-4/CYD-1	CDK-2/CYE-1	CDK-1/CYB-3
2	1	1	1	1	1	0	1	0	0	0	1	0	0

LIN-3	MPK-1	LIN-39	LS	LIN-12m	LIN-12i	CKI-1	EFL-1	LIN-35	SCF	APC	CDK-4/CYD-1	CDK-2/CYE-1	CDK-1/CYB-3
2	2	2	0	1	0	0	1	0	0	0	1	0	0

LIN-3	MPK-1	LIN-39	LS	LIN-12m	LIN-12i	CKI-1	EFL-1	LIN-35	SCF	APC	CDK-4/CYD-1	CDK-2/CYE-1	CDK-1/CYB-3
3	2	2	0	1	0	0	1	0	0	0	1	0	0

LIN-3	MPK-1	LIN-39	LS	LIN-12m	LIN-12i	CKI-1	EFL-1	LIN-35	SCF	APC	CDK-4/CYD-1	CDK-2/CYE-1	CDK-1/CYB-3
3	2	2	0	1	1	0	1	0	0	0	1	0	0

LIN-3	MPK-1	LIN-39	LS	LIN-12m	LIN-12i	CKI-1	EFL-1	LIN-35	SCF	APC	CDK-4/CYD-1	CDK-2/CYE-1	CDK-1/CYB-3
3	2	2	1	1	1	0	1	0	0	0	1	0	0

CDK-2/CYE-1 1

LIN-3	MPK-1	LIN-39	LS	LIN-12m	LIN-12i	CKI-1	EFL-1	LIN-35	SCF	APC	CDK-4/CYD-1	CDK-2/CYE-1	CDK-1/CYB-3
2	1	1	0	1	1	0	1	0	1	0	0	1	1

LIN-3	MPK-1	LIN-39	LS	LIN-12m	LIN-12i	CKI-1	EFL-1	LIN-35	SCF	APC	CDK-4/CYD-1	CDK-2/CYE-1	CDK-1/CYB-3
2	1	1	1	1	1	0	1	0	1	0	0	1	1

LIN-3	MPK-1	LIN-39	LS	LIN-12m	LIN-12i	CKI-1	EFL-1	LIN-35	SCF	APC	CDK-4/CYD-1	CDK-2/CYE-1	CDK-1/CYB-3
2	2	2	0	0	0	0	1	0	1	0	0	1	1

LIN-3	MPK-1	LIN-39	LS	LIN-12m	LIN-12i	CKI-1	EFL-1	LIN-35	SCF	APC	CDK-4/CYD-1	CDK-2/CYE-1	CDK-1/CYB-3
2	2	2	1	0	0	0	1	0	1	0	0	1	1

LIN-3	MPK-1	LIN-39	LS	LIN-12m	LIN-12i	CKI-1	EFL-1	LIN-35	SCF	APC	CDK-4/CYD-1	CDK-2/CYE-1	CDK-1/CYB-3
3	2	2	0	0	0	0	1	0	1	0	0	1	1

LIN-3	MPK-1	LIN-39	LS	LIN-12m	LIN-12i	CKI-1	EFL-1	LIN-35	SCF	APC	CDK-4/CYD-1	CDK-2/CYE-1	CDK-1/CYB-3
3	2	2	0	0	1	0	1	0	1	0	0	1	1

LIN-3	MPK-1	LIN-39	LS	LIN-12m	LIN-12i	CKI-1	EFL-1	LIN-35	SCF	APC	CDK-4/CYD-1	CDK-2/CYE-1	CDK-1/CYB-3
3	2	2	1	0	0	0	1	0	1	0	0	1	1

LIN-3	MPK-1	LIN-39	LS	LIN-12m	LIN-12i	CKI-1	EFL-1	LIN-35	SCF	APC	CDK-4/CYD-1	CDK-2/CYE-1	CDK-1/CYB-3
3	2	2	1	0	1	0	1	0	1	0	0	1	1

LIN-3	MPK-1	LIN-39	LS	LIN-12m	LIN-12i	CKI-1	EFL-1	LIN-35	SCF	APC	CDK-4/CYD-1	CDK-2/CYE-1	CDK-1/CYB-3
0	0	1	0	1	0	0	1	0	1	0	0	1	0
0	0	1	0	1	0	0	1	0	1	0	0	1	1
0	0	1	0	1	0	1	1	0	1	0	0	1	1
0	0	1	0	1	0	1	1	0	1	0	0	1	0

LIN-3	MPK-1	LIN-39	LS	LIN-12m	LIN-12i	CKI-1	EFL-1	LIN-35	SCF	APC	CDK-4/CYD-1	CDK-2/CYE-1	CDK-1/CYB-3
0	0	1	0	1	1	0	1	0	1	0	0	1	0
0	0	1	0	1	1	0	1	0	1	0	0	1	1
0	0	1	0	1	1	1	1	0	1	0	0	1	1
0	0	1	0	1	1	1	1	0	1	0	0	1	0

LIN-3	MPK-1	LIN-39	LS	LIN-12m	LIN-12i	CKI-1	EFL-1	LIN-35	SCF	APC	CDK-4/CYD-1	CDK-2/CYE-1	CDK-1/CYB-3
0	0	1	1	1	1	0	1	0	1	0	0	1	0
0	0	1	1	1	1	0	1	0	1	0	0	1	1
0	0	1	1	1	1	1	1	0	1	0	0	1	1
0	0	1	1	1	1	1	1	0	1	0	0	1	0

LIN-3	MPK-1	LIN-39	LS	LIN-12m	LIN-12i	CKI-1	EFL-1	LIN-35	SCF	APC	CDK-4/CYD-1	CDK-2/CYE-1	CDK-1/CYB-3
1	0	1	0	1	1	0	1	0	1	0	0	1	0
1	0	1	0	1	1	0	1	0	1	0	0	1	1
1	0	1	0	1	1	1	1	0	1	0	0	1	1
1	0	1	0	1	1	1	1	0	1	0	0	1	0

LIN-3	MPK-1	LIN-39	LS	LIN-12m	LIN-12i	CKI-1	EFL-1	LIN-35	SCF	APC	CDK-4/CYD-1	CDK-2/CYE-1	CDK-1/CYB-3
1	0	1	1	1	1	0	1	0	1	0	0	1	0
1	0	1	1	1	1	0	1	0	1	0	0	1	1
1	0	1	1	1	1	1	1	0	1	0	0	1	1

1	0	1	1	1	1	1	1	0	1	0	0	1	0

CDK-1/CYB-3 0

LIN-3	MPK-1	LIN-39	LS	LIN-12m	LIN-12i	CKI-1	EFL-1	LIN-35	SCF	APC	CDK-4/CYD-1	CDK-2/CYE-1	CDK-1/CYB-3
0	0	1	0	1	0	0	0	0	1	0	1	0	0
0	0	1	0	1	0	0	1	0	0	0	0	0	0
0	0	1	0	1	0	0	1	1	0	0	1	1	0

LIN-3	MPK-1	LIN-39	LS	LIN-12m	LIN-12i	CKI-1	EFL-1	LIN-35	SCF	APC	CDK-4/CYD-1	CDK-2/CYE-1	CDK-1/CYB-3
0	0	1	0	1	1	0	0	0	1	0	1	0	0
0	0	1	0	1	1	0	1	0	0	0	0	0	0
0	0	1	0	1	1	0	1	1	0	0	1	1	0

LIN-3	MPK-1	LIN-39	LS	LIN-12m	LIN-12i	CKI-1	EFL-1	LIN-35	SCF	APC	CDK-4/CYD-1	CDK-2/CYE-1	CDK-1/CYB-3
0	0	1	1	1	1	0	0	0	1	0	1	0	0
0	0	1	1	1	1	0	1	0	0	0	0	0	0
0	0	1	1	1	1	0	1	1	0	0	1	1	0

LIN-3	MPK-1	LIN-39	LS	LIN-12m	LIN-12i	CKI-1	EFL-1	LIN-35	SCF	APC	CDK-4/CYD-1	CDK-2/CYE-1	CDK-1/CYB-3
1	0	1	0	1	1	0	0	0	1	0	1	0	0
1	0	1	0	1	1	0	1	0	0	0	0	0	0
1	0	1	0	1	1	0	1	1	0	0	1	1	0

LIN-3	MPK-1	LIN-39	LS	LIN-12m	LIN-12i	CKI-1	EFL-1	LIN-35	SCF	APC	CDK-4/CYD-1	CDK-2/CYE-1	CDK-1/CYB-3
1	0	1	1	1	1	0	0	0	1	0	1	0	0
1	0	1	1	1	1	0	1	0	0	0	0	0	0
1	0	1	1	1	1	0	1	1	0	0	1	1	0

LIN-3	MPK-1	LIN-39	LS	LIN-12m	LIN-12i	CKI-1	EFL-1	LIN-35	SCF	APC	CDK-4/CYD-1	CDK-2/CYE-1	CDK-1/CYB-3
2	1	1	0	1	1	0	0	0	1	0	1	0	0
2	1	1	0	1	1	0	1	0	0	0	0	0	0
2	1	1	0	1	1	0	1	1	0	0	1	1	0

LIN-3	MPK-1	LIN-39	LS	LIN-12m	LIN-12i	CKI-1	EFL-1	LIN-35	SCF	APC	CDK-4/CYD-1	CDK-2/CYE-1	CDK-1/CYB-3
2	1	1	1	1	1	0	0	0	1	0	1	0	0
2	1	1	1	1	1	0	1	0	0	0	0	0	0
2	1	1	1	1	1	0	1	1	0	0	1	1	0

LIN-3	MPK-1	LIN-39	LS	LIN-12m	LIN-12i	CKI-1	EFL-1	LIN-35	SCF	APC	CDK-4/CYD-1	CDK-2/CYE-1	CDK-1/CYB-3
2	2	2	0	0	0	0	1	1	0	0	1	1	0
2	2	2	0	1	0	0	0	0	1	0	1	0	0
2	2	2	0	1	0	0	1	0	0	0	0	0	0

LIN-3	MPK-1	LIN-39	LS	LIN-12m	LIN-12i	CKI-1	EFL-1	LIN-35	SCF	APC	CDK-4/CYD-1	CDK-2/CYE-1	CDK-1/CYB-3
3	2	2	0	0	0	0	1	1	0	0	1	1	0
3	2	2	0	1	0	0	0	0	1	0	1	0	0
3	2	2	0	1	0	0	1	0	0	0	0	0	0

LIN-3	MPK-1	LIN-39	LS	LIN-12m	LIN-12i	CKI-1	EFL-1	LIN-35	SCF	APC	CDK-4/CYD-1	CDK-2/CYE-1	CDK-1/CYB-3
3	2	2	0	0	1	0	1	1	0	0	1	1	0
3	2	2	0	1	1	0	0	0	1	0	1	0	0
3	2	2	0	1	1	0	1	0	0	0	0	0	0

LIN-3	MPK-1	LIN-39	LS	LIN-12m	LIN-12i	CKI-1	EFL-1	LIN-35	SCF	APC	CDK-4/CYD-1	CDK-2/CYE-1	CDK-1/CYB-3
3	2	2	1	0	1	0	1	1	0	0	1	1	0
3	2	2	1	1	1	0	0	0	1	0	1	0	0
3	2	2	1	1	1	0	1	0	0	0	0	0	0

LIN-3	MPK-1	LIN-39	LS	LIN-12m	LIN-12i	CKI-1	EFL-1	LIN-35	SCF	APC	CDK-4/CYD-1	CDK-2/CYE-1	CDK-1/CYB-3
0	0	1	0	1	0	0	0	0	0	0	1	0	0
0	0	1	0	1	0	0	1	0	0	0	1	0	0
0	0	1	0	1	0	0	1	0	0	0	1	1	0
0	0	1	0	1	0	0	1	0	1	0	1	1	0
0	0	1	0	1	0	0	1	0	1	0	0	0	0
0	0	1	0	1	0	0	1	1	0	0	0	0	0
0	0	1	0	1	0	0	0	1	0	0	1	0	0

LIN-3	MPK-1	LIN-39	LS	LIN-12m	LIN-12i	CKI-1	EFL-1	LIN-35	SCF	APC	CDK-4/CYD-1	CDK-2/CYE-1	CDK-1/CYB-3
0	0	1	0	1	1	0	0	0	0	0	1	0	0
0	0	1	0	1	1	0	1	0	0	0	1	0	0
0	0	1	0	1	1	0	1	0	0	0	1	1	0
0	0	1	0	1	1	0	1	0	1	0	1	1	0
0	0	1	0	1	1	0	1	0	1	0	0	0	0
0	0	1	0	1	1	0	1	1	0	0	0	0	0
0	0	1	0	1	1	0	0	1	0	0	1	0	0

LIN-3	MPK-1	LIN-39	LS	LIN-12m	LIN-12i	CKI-1	EFL-1	LIN-35	SCF	APC	CDK-4/CYD-1	CDK-2/CYE-1	CDK-1/CYB-3
0	0	1	1	1	1	0	0	0	0	0	1	0	0
0	0	1	1	1	1	0	1	0	0	0	1	0	0
0	0	1	1	1	1	0	1	0	0	0	1	1	0
0	0	1	1	1	1	0	1	0	1	0	1	1	0
0	0	1	1	1	1	0	1	0	1	0	0	0	0
0	0	1	1	1	1	0	1	1	0	0	0	0	0
0	0	1	1	1	1	0	0	1	0	0	1	0	0

LIN-3	MPK-1	LIN-39	LS	LIN-12m	LIN-12i	CKI-1	EFL-1	LIN-35	SCF	APC	CDK-4/CYD-1	CDK-2/CYE-1	CDK-1/CYB-3

1	0	1	0	1	1	0	0	0	0	0	1	0	0
1	0	1	0	1	1	0	1	0	0	0	1	0	0
1	0	1	0	1	1	0	1	0	0	0	1	1	0
1	0	1	0	1	1	0	1	0	1	0	1	1	0
1	0	1	0	1	1	0	1	0	1	0	0	0	0
1	0	1	0	1	1	0	1	1	0	0	0	0	0
1	0	1	0	1	1	0	0	1	0	0	1	0	0

LIN-3	MPK-1	LIN-39	LS	LIN-12m	LIN-12i	CKI-1	EFL-1	LIN-35	SCF	APC	CDK-4/CYD-1	CDK-2/CYE-1	CDK-1/CYB-3
1	0	1	1	1	1	0	0	0	0	0	1	0	0
1	0	1	1	1	1	0	1	0	0	0	1	0	0
1	0	1	1	1	1	0	1	0	0	0	1	1	0
1	0	1	1	1	1	0	1	0	1	0	1	1	0
1	0	1	1	1	1	0	1	0	1	0	0	0	0
1	0	1	1	1	1	0	1	1	0	0	0	0	0
1	0	1	1	1	1	0	0	1	0	0	1	0	0

LIN-3	MPK-1	LIN-39	LS	LIN-12m	LIN-12i	CKI-1	EFL-1	LIN-35	SCF	APC	CDK-4/CYD-1	CDK-2/CYE-1	CDK-1/CYB-3
2	1	1	0	1	1	0	0	0	0	0	1	0	0
2	1	1	0	1	1	0	1	0	0	0	1	0	0
2	1	1	0	1	1	0	1	0	0	0	1	1	0
2	1	1	0	1	1	0	1	0	1	0	1	1	0
2	1	1	0	1	1	0	1	0	1	0	0	0	0
2	1	1	0	1	1	0	1	1	0	0	0	0	0
2	1	1	0	1	1	0	0	1	0	0	1	0	0

LIN-3	MPK-1	LIN-39	LS	LIN-12m	LIN-12i	CKI-1	EFL-1	LIN-35	SCF	APC	CDK-4/CYD-1	CDK-2/CYE-1	CDK-1/CYB-3
2	1	1	1	1	1	0	0	0	0	0	1	0	0
2	1	1	1	1	1	0	1	0	0	0	1	0	0
2	1	1	1	1	1	0	1	0	0	0	1	1	0
2	1	1	1	1	1	0	1	0	1	0	1	1	0
2	1	1	1	1	1	0	1	0	1	0	0	0	0
2	1	1	1	1	1	0	1	1	0	0	0	0	0
2	1	1	1	1	1	0	0	1	0	0	1	0	0

LIN-3	MPK-1	LIN-39	LS	LIN-12m	LIN-12i	CKI-1	EFL-1	LIN-35	SCF	APC	CDK-4/CYD-1	CDK-2/CYE-1	CDK-1/CYB-3
2	2	2	0	0	0	0	0	1	0	0	1	0	0
2	2	2	0	1	0	0	0	0	0	0	1	0	0
2	2	2	0	1	0	0	1	0	0	0	1	0	0
2	2	2	0	1	0	0	1	0	0	0	1	1	0
2	2	2	0	1	0	0	1	0	1	0	1	1	0
2	2	2	0	1	0	0	1	0	1	0	0	0	0
2	2	2	0	0	0	0	1	1	0	0	0	0	0

LIN-3	MPK-1	LIN-39	LS	LIN-12m	LIN-12i	CKI-1	EFL-1	LIN-35	SCF	APC	CDK-4/CYD-1	CDK-2/CYE-1	CDK-1/CYB-3
3	2	2	0	0	0	0	0	1	0	0	1	0	0
3	2	2	0	1	0	0	0	0	0	0	1	0	0
3	2	2	0	1	0	0	1	0	0	0	1	0	0
3	2	2	0	1	0	0	1	0	0	0	1	1	0
3	2	2	0	1	0	0	1	0	1	0	1	1	0
3	2	2	0	1	0	0	1	0	1	0	0	0	0
3	2	2	0	0	0	0	1	1	0	0	0	0	0

LIN-3	MPK-1	LIN-39	LS	LIN-12m	LIN-12i	CKI-1	EFL-1	LIN-35	SCF	APC	CDK-4/CYD-1	CDK-2/CYE-1	CDK-1/CYB-3
3	2	2	0	0	1	0	0	1	0	0	1	0	0
3	2	2	0	1	1	0	0	0	0	0	1	0	0
3	2	2	0	1	1	0	1	0	0	0	1	0	0
3	2	2	0	1	1	0	1	0	0	0	1	1	0
3	2	2	0	1	1	0	1	0	1	0	1	1	0
3	2	2	0	1	1	0	1	0	1	0	0	0	0
3	2	2	0	0	1	0	1	1	0	0	0	0	0

LIN-3	MPK-1	LIN-39	LS	LIN-12m	LIN-12i	CKI-1	EFL-1	LIN-35	SCF	APC	CDK-4/CYD-1	CDK-2/CYE-1	CDK-1/CYB-3
3	2	2	1	0	1	0	0	1	0	0	1	0	0
3	2	2	1	1	1	0	0	0	0	0	1	0	0
3	2	2	1	1	1	0	1	0	0	0	1	0	0
3	2	2	1	1	1	0	1	0	0	0	1	1	0
3	2	2	1	1	1	0	1	0	1	0	1	1	0
3	2	2	1	1	1	0	1	0	1	0	0	0	0
3	2	2	1	0	1	0	1	1	0	0	0	0	0

CDK-1/CYB-3 1

LIN-3	MPK-1	LIN-39	LS	LIN-12m	LIN-12i	CKI-1	EFL-1	LIN-35	SCF	APC	CDK-4/CYD-1	CDK-2/CYE-1	CDK-1/CYB-3
0	0	1	0	1	0	1	0	1	0	1	0	0	1

LIN-3	MPK-1	LIN-39	LS	LIN-12m	LIN-12i	CKI-1	EFL-1	LIN-35	SCF	APC	CDK-4/CYD-1	CDK-2/CYE-1	CDK-1/CYB-3
0	0	1	1	1	0	1	0	1	0	1	0	0	1

LIN-3	MPK-1	LIN-39	LS	LIN-12m	LIN-12i	CKI-1	EFL-1	LIN-35	SCF	APC	CDK-4/CYD-1	CDK-2/CYE-1	CDK-1/CYB-3
1	1	1	0	1	0	0	0	1	0	1	0	0	1

LIN-3	MPK-1	LIN-39	LS	LIN-12m	LIN-12i	CKI-1	EFL-1	LIN-35	SCF	APC	CDK-4/CYD-1	CDK-2/CYE-1	CDK-1/CYB-3
1	1	1	1	1	0	0	0	1	0	1	0	0	1

LIN-3	MPK-1	LIN-39	LS	LIN-12m	LIN-12i	CKI-1	EFL-1	LIN-35	SCF	APC	CDK-4/CYD-1	CDK-2/CYE-1	CDK-1/CYB-3
2	1	1	0	1	1	0	1	0	0	1	0	1	1

Mutations

LIN-3	MPK-1	LIN-39	LS	LIN-12m	LIN-12i	CKI-1	EFL-1	LIN-35	SCF	APC	CDK-4/CYD-1	CDK-2/CYE-1	CDK-1/CYB-3
2	1	1	1	1	1	0	1	0	0	1	0	1	1

LIN-3	MPK-1	LIN-39	LS	LIN-12m	LIN-12i	CKI-1	EFL-1	LIN-35	SCF	APC	CDK-4/CYD-1	CDK-2/CYE-1	CDK-1/CYB-3
2	2	2	0	0	0	0	0	1	0	1	0	0	1

LIN-3	MPK-1	LIN-39	LS	LIN-12m	LIN-12i	CKI-1	EFL-1	LIN-35	SCF	APC	CDK-4/CYD-1	CDK-2/CYE-1	CDK-1/CYB-3
2	2	2	0	0	0	0	1	0	0	1	0	1	1

LIN-3	MPK-1	LIN-39	LS	LIN-12m	LIN-12i	CKI-1	EFL-1	LIN-35	SCF	APC	CDK-4/CYD-1	CDK-2/CYE-1	CDK-1/CYB-3
2	2	2	1	0	0	0	0	1	0	1	0	0	1

LIN-3	MPK-1	LIN-39	LS	LIN-12m	LIN-12i	CKI-1	EFL-1	LIN-35	SCF	APC	CDK-4/CYD-1	CDK-2/CYE-1	CDK-1/CYB-3
2	2	2	1	0	0	0	1	0	0	1	0	1	1

LIN-3	MPK-1	LIN-39	LS	LIN-12m	LIN-12i	CKI-1	EFL-1	LIN-35	SCF	APC	CDK-4/CYD-1	CDK-2/CYE-1	CDK-1/CYB-3
3	2	2	0	0	0	0	0	1	0	1	0	0	1

LIN-3	MPK-1	LIN-39	LS	LIN-12m	LIN-12i	CKI-1	EFL-1	LIN-35	SCF	APC	CDK-4/CYD-1	CDK-2/CYE-1	CDK-1/CYB-3
3	2	2	0	0	0	0	1	0	0	1	0	1	1

LIN-3	MPK-1	LIN-39	LS	LIN-12m	LIN-12i	CKI-1	EFL-1	LIN-35	SCF	APC	CDK-4/CYD-1	CDK-2/CYE-1	CDK-1/CYB-3
3	2	2	0	0	1	0	1	0	0	1	0	1	1

LIN-3	MPK-1	LIN-39	LS	LIN-12m	LIN-12i	CKI-1	EFL-1	LIN-35	SCF	APC	CDK-4/CYD-1	CDK-2/CYE-1	CDK-1/CYB-3
3	2	2	1	0	0	0	0	1	0	1	0	0	1

LIN-3	MPK-1	LIN-39	LS	LIN-12m	LIN-12i	CKI-1	EFL-1	LIN-35	SCF	APC	CDK-4/CYD-1	CDK-2/CYE-1	CDK-1/CYB-3
3	2	2	1	0	0	0	1	0	0	1	0	1	1

LIN-3	MPK-1	LIN-39	LS	LIN-12m	LIN-12i	CKI-1	EFL-1	LIN-35	SCF	APC	CDK-4/CYD-1	CDK-2/CYE-1	CDK-1/CYB-3
3	2	2	1	0	1	0	1	0	0	1	0	1	1
