# Supplementary material for: A model of the regulatory network involved in the control of the cell cycle and cell differentiation in the Caenorhabditis elegans vulva
Source: BMC Bioinformatics. 2015 Mar 13;16:81. doi: 10.1186/s12859-015-0498-z (PMC4367908; doi:10.1186/s12859-015-0498-z)
Supplement: Additional file 3 — Table S2. The simulated phenotypic effect caused by removing each interaction [25,27,38,42,68,85,86,88,91-94,117,119,122,123]. [file 12859_2015_498_MOESM3_ESM.pdf]

Additional file 3: Table S2 The simulated phenotypic effect caused by removing each interaction

Phenotypes	Interactions removed
Wild type	*MPK-1 to LIN-12m [82,83], LIN-12i to LIN-12m [84], LIN-12i to LIN-12i [85], CDK-2/CYE-1 to LIN12i [42], CDK-1/CYB-3 to CKI-1, CDK-4/CYD-1 to CKI-1, CDK-2/CYE-1 to LIN-35, APC to SCF, SCF to APC, CKI-1 to CDK-2/CYE-1, CKI-1 to CDK-1/CYB-3, CDK-1/CYB-3 to CDK-1/CYB-3*
No primary fate	*LIN-3 to MPK-1 [27,86], MPK-1 to LIN-39 [68], LIN-39 to LIN-39 [72,87]*
No divisions	*CDK-4/CYD-1 to LIN-35, LIN-39 to SCF, CDK-2/CYE-1 to SCF, EFL-1 to CDK-2/CYE-1*
Endo-replication	*SCF to CDK-4/CYD-1, EFL-1 to CDK-1/CYB-3*
Longer cell cycle	*LIN-3 to MPK-1, MPK-1 to CKI-1, LIN-35 to CDK-2/CYE-1, SCF to CDK-2/CYE-1*
Shorter cell cycle	*APC to CKI-1, LIN-35 to EFL-1, CDK-1/CYB-3 to APC, CKI-1 to CDK-4/CYD-1, SCF to CDK-4/CYD-1, CDK-1/CYB-3 to CDK-4/CYD-1, LIN-35 to CDK-2/CYE-1, EFL-1 to CDK-1/CYB-3*
Modified cell cycle	*CDK-4/CYD-1 to CDK-1/CYB-3*
Secondary with LIN-3(0) and LS(0)	*CDK-1/CYB-3 to LIN12i [42], CDK-4/CYD-1 to LIN-35, LIN-39 to SCF, CDK-2/CYE-1 to SCF, SCF to CDK-4/CYD-1, EFL-1 to CDK-2/CYE-1, EFL-1 to CDK-1/CYB-3*
Secondary with LIN-3(2) and LS(0)	*CDK-1/CYB-3 to LIN12i [42], CDK-4/CYD-1 to LIN-35, LIN-39 to SCF, CDK-2/CYE-1 to SCF, SCF to CDK-4/CYD-1, EFL-1 to CDK-2/CYE-1, LIN-35 to CDK-2/CYE-1, SCF to CDK-2/CYE-1, EFL-1 to CDK-1/CYB-3, APC to CDK-1/CYB-3*
Primary with LIN-3(2) and LS(1)	*LIN-12i to MPK-1 [25,88], LIN-39 to LIN-12m [89], CDK-4/CYD-1 to LIN-12m [42], LS to LIN-12i [69,70], LIN-12m to LIN-12i [69,70], APC to CDK-1/CYB-3*
Tertiary with LIN-3(1) and LS(0)	*LIN-3 to LIN-12i [27,38]*
Tertiary with LIN-3(0) and LS(1)	*LIN-39 to LIN-12m [89], LS to LIN-12i [69,70], LIN-12m to LIN-12i [69,70]*

Interactions in black have a phenotype that reproduce what is reported in the literature; interactions in red have a simulated effect that differs from what is reported in the literature; and interactions in blue are predictions of our model.
