# Supplementary material for: A model of the regulatory network involved in the control of the cell cycle and cell differentiation in the Caenorhabditis elegans vulva
Source: BMC Bioinformatics. 2015 Mar 13;16:81. doi: 10.1186/s12859-015-0498-z (PMC4367908; doi:10.1186/s12859-015-0498-z)
Supplement: Additional file 4 — Interactions. This file contains the attractors produced by the simulation of each interaction removal. [file 12859_2015_498_MOESM4_ESM.pdf]

Interactions

LIN-3 **to** **MPK-1**

LIN-3	MPK-1	LIN-39	LS	LIN-12m	LIN-12i	CKI-1	EFL-1	LIN-35	SCF	APC	CDK-4/CYD-1	CDK-2/CYE-1	CDK-1/CYB-3
0	0	1	0	1	0	0	0	0	0	0	1	0	0
0	0	1	0	1	0	0	1	0	0	0	1	0	0
0	0	1	0	1	0	0	1	0	0	0	1	1	0
0	0	1	0	1	0	0	1	0	1	0	1	1	0
0	0	1	0	1	0	0	1	0	1	0	0	0	0
0	0	1	0	1	0	0	1	1	0	0	0	0	1
0	0	1	0	1	0	1	0	1	0	1	0	0	1
0	0	1	0	1	0	1	0	1	0	1	0	0	0
0	0	1	0	1	0	1	0	1	0	0	0	0	0
0	0	1	0	1	0	0	0	1	0	0	0	0	0
0	0	1	0	1	0	0	0	1	0	0	1	0	0

LIN-3	MPK-1	LIN-39	LS	LIN-12m	LIN-12i	CKI-1	EFL-1	LIN-35	SCF	APC	CDK-4/CYD-1	CDK-2/CYE-1	CDK-1/CYB-3
0	0	1	1	1	0	1	0	1	0	1	0	0	0
0	0	1	1	1	1	1	0	1	0	0	0	0	0
0	0	1	1	1	1	0	0	1	0	0	0	0	0
0	0	1	1	1	1	0	0	1	0	0	1	0	0
0	0	1	1	1	1	0	0	0	0	0	1	0	0
0	0	1	1	1	1	0	1	0	0	0	1	0	0
0	0	1	1	1	1	0	1	0	0	0	1	1	0
0	0	1	1	1	1	0	1	0	1	0	1	1	0
0	0	1	1	1	1	0	1	0	1	0	0	0	0
0	0	1	1	1	1	0	1	1	0	0	0	0	1
0	0	1	1	1	0	1	0	1	0	1	0	0	1

LIN-3	MPK-1	LIN-39	LS	LIN-12m	LIN-12i	CKI-1	EFL-1	LIN-35	SCF	APC	CDK-4/CYD-1	CDK-2/CYE-1	CDK-1/CYB-3
1	0	1	0	1	0	1	0	1	0	1	0	0	0
1	0	1	0	1	1	1	0	1	0	0	0	0	0
1	0	1	0	1	1	0	0	1	0	0	0	0	0
1	0	1	0	1	1	0	0	1	0	0	1	0	0
1	0	1	0	1	1	0	0	0	0	0	1	0	0
1	0	1	0	1	1	0	1	0	0	0	1	0	0
1	0	1	0	1	1	0	1	0	0	0	1	1	0
1	0	1	0	1	1	0	1	0	1	0	1	1	0
1	0	1	0	1	1	0	1	0	1	0	0	0	0
1	0	1	0	1	1	0	1	1	0	0	0	0	1
1	0	1	0	1	0	1	0	1	0	1	0	0	1

LIN-3	MPK-1	LIN-39	LS	LIN-12m	LIN-12i	CKI-1	EFL-1	LIN-35	SCF	APC	CDK-4/CYD-1	CDK-2/CYE-1	CDK-1/CYB-3
1	0	1	1	1	0	1	0	1	0	1	0	0	0
1	0	1	1	1	1	1	0	1	0	0	0	0	0
1	0	1	1	1	1	0	0	1	0	0	0	0	0
1	0	1	1	1	1	0	0	1	0	0	1	0	0
1	0	1	1	1	1	0	0	0	0	0	1	0	0
1	0	1	1	1	1	0	1	0	0	0	1	0	0
1	0	1	1	1	1	0	1	0	0	0	1	1	0
1	0	1	1	1	1	0	1	0	1	0	1	1	0
1	0	1	1	1	1	0	1	0	1	0	0	0	0
1	0	1	1	1	1	0	1	1	0	0	0	0	1
1	0	1	1	1	0	1	0	1	0	1	0	0	1

LIN-3	MPK-1	LIN-39	LS	LIN-12m	LIN-12i	CKI-1	EFL-1	LIN-35	SCF	APC	CDK-4/CYD-1	CDK-2/CYE-1	CDK-1/CYB-3
2	0	1	0	1	0	0	0	0	0	0	1	0	0
2	0	1	0	1	0	0	1	0	0	0	1	0	0
2	0	1	0	1	0	0	1	0	0	0	1	1	0
2	0	1	0	1	0	0	1	0	1	0	1	1	0
2	0	1	0	1	0	0	1	0	1	0	0	0	0
2	0	1	0	1	0	0	1	1	0	0	0	0	1

2	0	1	0	1	0	1	0	1	0	1	0	0	1
2	0	1	0	1	0	1	0	1	0	1	0	0	0
2	0	1	0	1	0	1	0	1	0	0	0	0	0
2	0	1	0	1	0	0	0	1	0	0	0	0	0
2	0	1	0	1	0	0	0	1	0	0	1	0	0

LIN-3	MPK-1	LIN-39	LS	LIN-12m	LIN-12i	CKI-1	EFL-1	LIN-35	SCF	APC	CDK-4/CYD-1	CDK-2/CYE-1	CDK-1/CYB-3
2	0	1	1	1	0	1	0	1	0	1	0	0	0
2	0	1	1	1	1	1	0	1	0	0	0	0	0
2	0	1	1	1	1	0	0	1	0	0	0	0	0
2	0	1	1	1	1	0	0	1	0	0	1	0	0
2	0	1	1	1	1	0	0	0	0	0	1	0	0
2	0	1	1	1	1	0	1	0	0	0	1	0	0
2	0	1	1	1	1	0	1	0	0	0	1	1	0
2	0	1	1	1	1	0	1	0	1	0	1	1	0
2	0	1	1	1	1	0	1	0	1	0	0	0	0
2	0	1	1	1	1	0	1	1	0	0	0	0	1
2	0	1	1	1	0	1	0	1	0	1	0	0	1

LIN-3	MPK-1	LIN-39	LS	LIN-12m	LIN-12i	CKI-1	EFL-1	LIN-35	SCF	APC	CDK-4/CYD-1	CDK-2/CYE-1	CDK-1/CYB-3
3	0	1	0	1	0	0	0	0	0	0	1	0	0
3	0	1	0	1	0	0	1	0	0	0	1	0	0
3	0	1	0	1	0	0	1	0	0	0	1	1	0
3	0	1	0	1	0	0	1	0	1	0	1	1	0
3	0	1	0	1	0	0	1	0	1	0	0	0	0
3	0	1	0	1	0	0	1	1	0	0	0	0	1
3	0	1	0	1	0	1	0	1	0	1	0	0	1
3	0	1	0	1	0	1	0	1	0	1	0	0	0
3	0	1	0	1	0	1	0	1	0	0	0	0	0
3	0	1	0	1	0	0	0	1	0	0	0	0	0
3	0	1	0	1	0	0	0	1	0	0	1	0	0

LIN-3	MPK-1	LIN-39	LS	LIN-12m	LIN-12i	CKI-1	EFL-1	LIN-35	SCF	APC	CDK-4/CYD-1	CDK-2/CYE-1	CDK-1/CYB-3
3	0	1	1	1	0	1	0	1	0	1	0	0	0
3	0	1	1	1	1	1	0	1	0	0	0	0	0
3	0	1	1	1	1	0	0	1	0	0	0	0	0
3	0	1	1	1	1	0	0	1	0	0	1	0	0
3	0	1	1	1	1	0	0	0	0	0	1	0	0
3	0	1	1	1	1	0	1	0	0	0	1	0	0
3	0	1	1	1	1	0	1	0	0	0	1	1	0
3	0	1	1	1	1	0	1	0	1	0	1	1	0
3	0	1	1	1	1	0	1	0	1	0	0	0	0
3	0	1	1	1	1	0	1	1	0	0	0	0	1
3	0	1	1	1	0	1	0	1	0	1	0	0	1

LIN-12i to MPK-1

LIN-3	MPK-1	LIN-39	LS	LIN-12m	LIN-12i	CKI-1	EFL-1	LIN-35	SCF	APC	CDK-4/CYD-1	CDK-2/CYE-1	CDK-1/CYB-3
1	1	1	0	1	0	0	0	1	0	1	0	0	0
1	1	1	0	1	1	0	0	1	0	0	1	0	0
1	1	1	0	1	1	0	0	0	0	0	1	0	0
1	1	1	0	1	1	0	1	0	0	0	1	0	0
1	1	1	0	1	1	0	1	0	0	0	1	1	0
1	1	1	0	1	1	0	1	0	1	0	1	1	0
1	1	1	0	1	1	0	1	0	1	0	0	0	0
1	1	1	0	1	1	0	1	1	0	0	0	0	1
1	1	1	0	1	0	0	0	1	0	1	0	0	1

LIN-3	MPK-1	LIN-39	LS	LIN-12m	LIN-12i	CKI-1	EFL-1	LIN-35	SCF	APC	CDK-4/CYD-1	CDK-2/CYE-1	CDK-1/CYB-3
1	1	1	1	1	0	0	0	1	0	1	0	0	0
1	1	1	1	1	1	0	0	1	0	0	1	0	0

1	1	1	1	1	1	0	0	0	0	0	1	0	0
1	1	1	1	1	1	0	1	0	0	0	1	0	0
1	1	1	1	1	1	0	1	0	0	0	1	1	0
1	1	1	1	1	1	0	1	0	1	0	1	1	0
1	1	1	1	1	1	0	1	0	1	0	0	0	0
1	1	1	1	1	1	0	1	1	0	0	0	0	1
1	1	1	1	1	0	0	0	1	0	1	0	0	1

LIN-3	MPK-1	LIN-39	LS	LIN-12m	LIN-12i	CKI-1	EFL-1	LIN-35	SCF	APC	CDK-4/CYD-1	CDK-2/CYE-1	CDK-1/CYB-3
2	2	2	0	0	0	0	0	1	0	0	1	0	0
2	2	2	0	1	0	0	0	0	0	0	1	0	0
2	2	2	0	1	0	0	1	0	0	0	1	0	0
2	2	2	0	1	0	0	1	0	0	0	1	1	0
2	2	2	0	1	0	0	1	0	1	0	1	1	0
2	2	2	0	1	0	0	1	0	1	0	0	0	0
2	2	2	0	0	0	0	1	1	0	0	0	0	1
2	2	2	0	0	0	0	0	1	0	1	0	0	1
2	2	2	0	0	0	0	0	1	0	1	0	0	0

LIN-3	MPK-1	LIN-39	LS	LIN-12m	LIN-12i	CKI-1	EFL-1	LIN-35	SCF	APC	CDK-4/CYD-1	CDK-2/CYE-1	CDK-1/CYB-3
2	2	2	1	0	0	0	0	1	0	0	1	0	0
2	2	2	1	1	0	0	0	0	0	0	1	0	0
2	2	2	1	1	1	0	1	0	0	0	1	0	0
2	2	2	1	1	1	0	1	0	0	0	1	1	0
2	2	2	1	1	1	0	1	0	1	0	1	1	0
2	2	2	1	1	1	0	1	0	1	0	0	0	0
2	2	2	1	0	1	0	1	1	0	0	0	0	1
2	2	2	1	0	0	0	0	1	0	1	0	0	1
2	2	2	1	0	0	0	0	1	0	1	0	0	0

LIN-3	MPK-1	LIN-39	LS	LIN-12m	LIN-12i	CKI-1	EFL-1	LIN-35	SCF	APC	CDK-4/CYD-1	CDK-2/CYE-1	CDK-1/CYB-3
3	2	2	0	0	0	0	0	1	0	0	1	0	0
3	2	2	0	1	0	0	0	0	0	0	1	0	0
3	2	2	0	1	0	0	1	0	0	0	1	0	0
3	2	2	0	1	0	0	1	0	0	0	1	1	0
3	2	2	0	1	0	0	1	0	1	0	1	1	0
3	2	2	0	1	0	0	1	0	1	0	0	0	0
3	2	2	0	0	0	0	1	1	0	0	0	0	1
3	2	2	0	0	0	0	0	1	0	1	0	0	1
3	2	2	0	0	0	0	0	1	0	1	0	0	0

LIN-3	MPK-1	LIN-39	LS	LIN-12m	LIN-12i	CKI-1	EFL-1	LIN-35	SCF	APC	CDK-4/CYD-1	CDK-2/CYE-1	CDK-1/CYB-3
3	2	2	1	0	0	0	0	1	0	0	1	0	0
3	2	2	1	1	0	0	0	0	0	0	1	0	0
3	2	2	1	1	1	0	1	0	0	0	1	0	0
3	2	2	1	1	1	0	1	0	0	0	1	1	0
3	2	2	1	1	1	0	1	0	1	0	1	1	0
3	2	2	1	1	1	0	1	0	1	0	0	0	0
3	2	2	1	0	1	0	1	1	0	0	0	0	1
3	2	2	1	0	0	0	0	1	0	1	0	0	1
3	2	2	1	0	0	0	0	1	0	1	0	0	0

LIN-3	MPK-1	LIN-39	LS	LIN-12m	LIN-12i	CKI-1	EFL-1	LIN-35	SCF	APC	CDK-4/CYD-1	CDK-2/CYE-1	CDK-1/CYB-3
0	0	1	0	1	0	0	0	0	0	0	1	0	0
0	0	1	0	1	0	0	1	0	0	0	1	0	0
0	0	1	0	1	0	0	1	0	0	0	1	1	0
0	0	1	0	1	0	0	1	0	1	0	1	1	0
0	0	1	0	1	0	0	1	0	1	0	0	0	0
0	0	1	0	1	0	0	1	1	0	0	0	0	1
0	0	1	0	1	0	1	0	1	0	1	0	0	1
0	0	1	0	1	0	1	0	1	0	1	0	0	0

0	0	1	0	1	0	1	0	1	0	0	0	0	0
0	0	1	0	1	0	0	0	1	0	0	0	0	0
0	0	1	0	1	0	0	0	1	0	0	1	0	0

LIN-3	MPK-1	LIN-39	LS	LIN-12m	LIN-12i	CKI-1	EFL-1	LIN-35	SCF	APC	CDK-4/CYD-1	CDK-2/CYE-1	CDK-1/CYB-3
0	0	1	1	1	0	1	0	1	0	1	0	0	0
0	0	1	1	1	1	1	0	1	0	0	0	0	0
0	0	1	1	1	1	0	0	1	0	0	0	0	0
0	0	1	1	1	1	0	0	1	0	0	1	0	0
0	0	1	1	1	1	0	0	0	0	0	1	0	0
0	0	1	1	1	1	0	1	0	0	0	1	0	0
0	0	1	1	1	1	0	1	0	0	0	1	1	0
0	0	1	1	1	1	0	1	0	1	0	1	1	0
0	0	1	1	1	1	0	1	0	1	0	0	0	0
0	0	1	1	1	1	0	1	1	0	0	0	0	1
0	0	1	1	1	0	1	0	1	0	1	0	0	1

MPK-1 to LIN-39

LIN-3	MPK-1	LIN-39	LS	LIN-12m	LIN-12i	CKI-1	EFL-1	LIN-35	SCF	APC	CDK-4/CYD-1	CDK-2/CYE-1	CDK-1/CYB-3
2	1	1	1	1	0	0	0	1	0	1	0	0	1
2	2	1	1	1	0	0	0	1	0	1	0	0	0
2	2	1	1	0	1	0	0	1	0	0	1	0	0
2	1	1	1	1	1	0	0	0	0	0	1	0	0
2	1	1	1	1	1	0	1	0	0	0	1	0	0
2	1	1	1	1	1	0	1	0	0	0	1	1	0
2	1	1	1	1	1	0	1	0	1	0	1	1	0
2	1	1	1	1	1	0	1	0	1	0	0	0	0
2	1	1	1	1	1	0	1	1	0	0	0	0	1

LIN-3	MPK-1	LIN-39	LS	LIN-12m	LIN-12i	CKI-1	EFL-1	LIN-35	SCF	APC	CDK-4/CYD-1	CDK-2/CYE-1	CDK-1/CYB-3
2	2	1	0	0	0	0	0	1	0	0	1	0	0
2	2	1	0	1	0	0	0	0	0	0	1	0	0
2	2	1	0	1	0	0	1	0	0	0	1	0	0
2	2	1	0	1	0	0	1	0	0	0	1	1	0
2	2	1	0	1	0	0	1	0	1	0	1	1	0
2	2	1	0	1	0	0	1	0	1	0	0	0	0
2	2	1	0	0	0	0	1	1	0	0	0	0	1
2	2	1	0	0	0	0	0	1	0	1	0	0	1
2	2	1	0	0	0	0	0	1	0	1	0	0	0

LIN-3	MPK-1	LIN-39	LS	LIN-12m	LIN-12i	CKI-1	EFL-1	LIN-35	SCF	APC	CDK-4/CYD-1	CDK-2/CYE-1	CDK-1/CYB-3
3	2	1	0	0	0	0	0	1	0	0	1	0	0
3	2	1	0	1	0	0	0	0	0	0	1	0	0
3	2	1	0	1	0	0	1	0	0	0	1	0	0
3	2	1	0	1	0	0	1	0	0	0	1	1	0
3	2	1	0	1	0	0	1	0	1	0	1	1	0
3	2	1	0	1	0	0	1	0	1	0	0	0	0
3	2	1	0	0	0	0	1	1	0	0	0	0	1
3	2	1	0	0	0	0	0	1	0	1	0	0	1
3	2	1	0	0	0	0	0	1	0	1	0	0	0

LIN-3	MPK-1	LIN-39	LS	LIN-12m	LIN-12i	CKI-1	EFL-1	LIN-35	SCF	APC	CDK-4/CYD-1	CDK-2/CYE-1	CDK-1/CYB-3
3	2	1	1	0	0	0	0	1	0	0	1	0	0
3	2	1	1	1	0	0	0	0	0	0	1	0	0
3	2	1	1	1	1	0	1	0	0	0	1	0	0
3	2	1	1	1	1	0	1	0	0	0	1	1	0
3	2	1	1	1	1	0	1	0	1	0	1	1	0
3	2	1	1	1	1	0	1	0	1	0	0	0	0
3	2	1	1	0	1	0	1	1	0	0	0	0	1
3	2	1	1	0	0	0	0	1	0	1	0	0	1

3	2	1	1	0	0	0	0	1	0	1	0	0	0

LIN-3	MPK-1	LIN-39	LS	LIN-12m	LIN-12i	CKI-1	EFL-1	LIN-35	SCF	APC	CDK-4/CYD-1	CDK-2/CYE-1	CDK-1/CYB-3
1	0	1	0	1	0	1	0	1	0	1	0	0	1
1	1	1	0	1	0	1	0	1	0	1	0	0	0
1	1	1	0	1	1	0	0	1	0	0	0	0	0
1	0	1	0	1	1	0	0	1	0	0	1	0	0
1	0	1	0	1	1	0	0	0	0	0	1	0	0
1	0	1	0	1	1	0	1	0	0	0	1	0	0
1	0	1	0	1	1	0	1	0	0	0	1	1	0
1	0	1	0	1	1	0	1	0	1	0	1	1	0
1	0	1	0	1	1	0	1	0	1	0	0	0	0
1	0	1	0	1	1	0	1	1	0	0	0	0	1

LIN-3	MPK-1	LIN-39	LS	LIN-12m	LIN-12i	CKI-1	EFL-1	LIN-35	SCF	APC	CDK-4/CYD-1	CDK-2/CYE-1	CDK-1/CYB-3
1	0	1	1	1	0	1	0	1	0	1	0	0	1
1	1	1	1	1	0	1	0	1	0	1	0	0	0
1	1	1	1	1	1	0	0	1	0	0	0	0	0
1	0	1	1	1	1	0	0	1	0	0	1	0	0
1	0	1	1	1	1	0	0	0	0	0	1	0	0
1	0	1	1	1	1	0	1	0	0	0	1	0	0
1	0	1	1	1	1	0	1	0	0	0	1	1	0
1	0	1	1	1	1	0	1	0	1	0	1	1	0
1	0	1	1	1	1	0	1	0	1	0	0	0	0
1	0	1	1	1	1	0	1	1	0	0	0	0	1

LIN-3	MPK-1	LIN-39	LS	LIN-12m	LIN-12i	CKI-1	EFL-1	LIN-35	SCF	APC	CDK-4/CYD-1	CDK-2/CYE-1	CDK-1/CYB-3
0	0	1	0	1	0	0	0	0	0	0	1	0	0
0	0	1	0	1	0	0	1	0	0	0	1	0	0
0	0	1	0	1	0	0	1	0	0	0	1	1	0
0	0	1	0	1	0	0	1	0	1	0	1	1	0
0	0	1	0	1	0	0	1	0	1	0	0	0	0
0	0	1	0	1	0	0	1	1	0	0	0	0	1
0	0	1	0	1	0	1	0	1	0	1	0	0	1
0	0	1	0	1	0	1	0	1	0	1	0	0	0
0	0	1	0	1	0	1	0	1	0	0	0	0	0
0	0	1	0	1	0	0	0	1	0	0	0	0	0
0	0	1	0	1	0	0	0	1	0	0	1	0	0

LIN-3	MPK-1	LIN-39	LS	LIN-12m	LIN-12i	CKI-1	EFL-1	LIN-35	SCF	APC	CDK-4/CYD-1	CDK-2/CYE-1	CDK-1/CYB-3
0	0	1	1	1	0	1	0	1	0	1	0	0	0
0	0	1	1	1	1	1	0	1	0	0	0	0	0
0	0	1	1	1	1	0	0	1	0	0	0	0	0
0	0	1	1	1	1	0	0	1	0	0	1	0	0
0	0	1	1	1	1	0	0	0	0	0	1	0	0
0	0	1	1	1	1	0	1	0	0	0	1	0	0
0	0	1	1	1	1	0	1	0	0	0	1	1	0
0	0	1	1	1	1	0	1	0	1	0	1	1	0
0	0	1	1	1	1	0	1	0	1	0	0	0	0
0	0	1	1	1	1	0	1	1	0	0	0	0	1
0	0	1	1	1	0	1	0	1	0	1	0	0	1

LIN-39 to LIN-39

LIN-3	MPK-1	LIN-39	LS	LIN-12m	LIN-12i	CKI-1	EFL-1	LIN-35	SCF	APC	CDK-4/CYD-1	CDK-2/CYE-1	CDK-1/CYB-3
2	1	1	1	1	0	0	0	1	0	1	0	0	1
2	2	1	1	1	0	0	0	1	0	1	0	0	0
2	2	1	1	0	1	0	0	1	0	0	1	0	0
2	1	1	1	1	1	0	0	0	0	0	1	0	0
2	1	1	1	1	1	0	1	0	0	0	1	0	0
2	1	1	1	1	1	0	1	0	0	0	1	1	0

2	1	1	1	1	1	0	1	0	1	0	1	1	0
2	1	1	1	1	1	0	1	0	1	0	0	0	0
2	1	1	1	1	1	0	1	1	0	0	0	0	1

LIN-3	MPK-1	LIN-39	LS	LIN-12m	LIN-12i	CKI-1	EFL-1	LIN-35	SCF	APC	CDK-4/CYD-1	CDK-2/CYE-1	CDK-1/CYB-3
2	2	1	0	0	0	0	0	1	0	0	1	0	0
2	2	1	0	1	0	0	0	0	0	0	1	0	0
2	2	1	0	1	0	0	1	0	0	0	1	0	0
2	2	1	0	1	0	0	1	0	0	0	1	1	0
2	2	1	0	1	0	0	1	0	1	0	1	1	0
2	2	1	0	1	0	0	1	0	1	0	0	0	0
2	2	1	0	0	0	0	1	1	0	0	0	0	1
2	2	1	0	0	0	0	0	1	0	1	0	0	1
2	2	1	0	0	0	0	0	1	0	1	0	0	0

LIN-3	MPK-1	LIN-39	LS	LIN-12m	LIN-12i	CKI-1	EFL-1	LIN-35	SCF	APC	CDK-4/CYD-1	CDK-2/CYE-1	CDK-1/CYB-3
3	2	1	0	0	0	0	0	1	0	0	1	0	0
3	2	1	0	1	0	0	0	0	0	0	1	0	0
3	2	1	0	1	0	0	1	0	0	0	1	0	0
3	2	1	0	1	0	0	1	0	0	0	1	1	0
3	2	1	0	1	0	0	1	0	1	0	1	1	0
3	2	1	0	1	0	0	1	0	1	0	0	0	0
3	2	1	0	0	0	0	1	1	0	0	0	0	1
3	2	1	0	0	0	0	0	1	0	1	0	0	1
3	2	1	0	0	0	0	0	1	0	1	0	0	0

LIN-3	MPK-1	LIN-39	LS	LIN-12m	LIN-12i	CKI-1	EFL-1	LIN-35	SCF	APC	CDK-4/CYD-1	CDK-2/CYE-1	CDK-1/CYB-3
3	2	1	1	0	0	0	0	1	0	0	1	0	0
3	2	1	1	1	0	0	0	0	0	0	1	0	0
3	2	1	1	1	1	0	1	0	0	0	1	0	0
3	2	1	1	1	1	0	1	0	0	0	1	1	0
3	2	1	1	1	1	0	1	0	1	0	1	1	0
3	2	1	1	1	1	0	1	0	1	0	0	0	0
3	2	1	1	0	1	0	1	1	0	0	0	0	1
3	2	1	1	0	0	0	0	1	0	1	0	0	1
3	2	1	1	0	0	0	0	1	0	1	0	0	0

LIN-3	MPK-1	LIN-39	LS	LIN-12m	LIN-12i	CKI-1	EFL-1	LIN-35	SCF	APC	CDK-4/CYD-1	CDK-2/CYE-1	CDK-1/CYB-3
1	0	1	0	1	0	1	0	1	0	1	0	0	1
1	1	1	0	1	0	1	0	1	0	1	0	0	0
1	1	1	0	1	1	0	0	1	0	0	0	0	0
1	0	1	0	1	1	0	0	1	0	0	1	0	0
1	0	1	0	1	1	0	0	0	0	0	1	0	0
1	0	1	0	1	1	0	1	0	0	0	1	0	0
1	0	1	0	1	1	0	1	0	0	0	1	1	0
1	0	1	0	1	1	0	1	0	1	0	1	1	0
1	0	1	0	1	1	0	1	0	1	0	0	0	0
1	0	1	0	1	1	0	1	1	0	0	0	0	1

LIN-3	MPK-1	LIN-39	LS	LIN-12m	LIN-12i	CKI-1	EFL-1	LIN-35	SCF	APC	CDK-4/CYD-1	CDK-2/CYE-1	CDK-1/CYB-3
1	0	1	1	1	0	1	0	1	0	1	0	0	1
1	1	1	1	1	0	1	0	1	0	1	0	0	0
1	1	1	1	1	1	0	0	1	0	0	0	0	0
1	0	1	1	1	1	0	0	1	0	0	1	0	0
1	0	1	1	1	1	0	0	0	0	0	1	0	0
1	0	1	1	1	1	0	1	0	0	0	1	0	0
1	0	1	1	1	1	0	1	0	0	0	1	1	0
1	0	1	1	1	1	0	1	0	1	0	1	1	0
1	0	1	1	1	1	0	1	0	1	0	0	0	0
1	0	1	1	1	1	0	1	1	0	0	0	0	1

LIN-3	MPK-1	LIN-39	LS	LIN-12m	LIN-12i	CKI-1	EFL-1	LIN-35	SCF	APC	CDK-4/CYD-1	CDK-2/CYE-1	CDK-1/CYB-3
0	0	1	0	1	0	0	0	0	0	0	1	0	0
0	0	1	0	1	0	0	1	0	0	0	1	0	0
0	0	1	0	1	0	0	1	0	0	0	1	1	0
0	0	1	0	1	0	0	1	0	1	0	1	1	0
0	0	1	0	1	0	0	1	0	1	0	0	0	0
0	0	1	0	1	0	0	1	1	0	0	0	0	1
0	0	1	0	1	0	1	0	1	0	1	0	0	1
0	0	1	0	1	0	1	0	1	0	1	0	0	0
0	0	1	0	1	0	1	0	1	0	0	0	0	0
0	0	1	0	1	0	0	0	1	0	0	0	0	0
0	0	1	0	1	0	0	0	1	0	0	1	0	0

LIN-3	MPK-1	LIN-39	LS	LIN-12m	LIN-12i	CKI-1	EFL-1	LIN-35	SCF	APC	CDK-4/CYD-1	CDK-2/CYE-1	CDK-1/CYB-3
0	0	1	1	1	0	1	0	1	0	1	0	0	0
0	0	1	1	1	1	1	0	1	0	0	0	0	0
0	0	1	1	1	1	0	0	1	0	0	0	0	0
0	0	1	1	1	1	0	0	1	0	0	1	0	0
0	0	1	1	1	1	0	0	0	0	0	1	0	0
0	0	1	1	1	1	0	1	0	0	0	1	0	0
0	0	1	1	1	1	0	1	0	0	0	1	1	0
0	0	1	1	1	1	0	1	0	1	0	1	1	0
0	0	1	1	1	1	0	1	0	1	0	0	0	0
0	0	1	1	1	1	0	1	1	0	0	0	0	1
0	0	1	1	1	0	1	0	1	0	1	0	0	1

MPK-1 to LIN-12m

LIN-3	MPK-1	LIN-39	LS	LIN-12m	LIN-12i	CKI-1	EFL-1	LIN-35	SCF	APC	CDK-4/CYD-1	CDK-2/CYE-1	CDK-1/CYB-3
2	1	1	1	1	0	0	0	1	0	1	0	0	1
2	2	1	1	1	0	0	0	1	0	1	0	0	0
2	2	2	1	1	1	0	0	1	0	0	1	0	0
2	1	2	1	1	1	0	0	0	0	0	1	0	0
2	1	1	1	1	1	0	1	0	0	0	1	0	0
2	1	1	1	1	1	0	1	0	0	0	1	1	0
2	1	1	1	1	1	0	1	0	1	0	1	1	0
2	1	1	1	1	1	0	1	0	1	0	0	0	0
2	1	1	1	1	1	0	1	1	0	0	0	0	1

LIN-3	MPK-1	LIN-39	LS	LIN-12m	LIN-12i	CKI-1	EFL-1	LIN-35	SCF	APC	CDK-4/CYD-1	CDK-2/CYE-1	CDK-1/CYB-3
2	2	2	0	1	0	0	0	0	0	0	1	0	0
2	2	2	0	1	0	0	1	0	0	0	1	0	0
2	2	2	0	1	0	0	1	0	0	0	1	1	0
2	2	2	0	1	0	0	1	0	1	0	1	1	0
2	2	2	0	1	0	0	1	0	1	0	0	0	0
2	2	2	0	1	0	0	1	1	0	0	0	0	1
2	2	2	0	1	0	0	0	1	0	1	0	0	1
2	2	2	0	1	0	0	0	1	0	1	0	0	0
2	2	2	0	1	0	0	0	1	0	0	1	0	0

LIN-3	MPK-1	LIN-39	LS	LIN-12m	LIN-12i	CKI-1	EFL-1	LIN-35	SCF	APC	CDK-4/CYD-1	CDK-2/CYE-1	CDK-1/CYB-3
3	2	2	0	1	0	0	0	0	0	0	1	0	0
3	2	2	0	1	0	0	1	0	0	0	1	0	0
3	2	2	0	1	0	0	1	0	0	0	1	1	0
3	2	2	0	1	0	0	1	0	1	0	1	1	0
3	2	2	0	1	0	0	1	0	1	0	0	0	0
3	2	2	0	1	0	0	1	1	0	0	0	0	1
3	2	2	0	1	0	0	0	1	0	1	0	0	1
3	2	2	0	1	0	0	0	1	0	1	0	0	0
3	2	2	0	1	0	0	0	1	0	0	1	0	0

LIN-3	MPK-1	LIN-39	LS	LIN-12m	LIN-12i	CKI-1	EFL-1	LIN-35	SCF	APC	CDK-4/CYD-1	CDK-2/CYE-1	CDK-1/CYB-3
3	2	2	1	1	0	0	0	1	0	1	0	0	0
3	2	2	1	1	1	0	0	1	0	0	1	0	0
3	2	2	1	1	1	0	0	0	0	0	1	0	0
3	2	2	1	1	1	0	1	0	0	0	1	0	0
3	2	2	1	1	1	0	1	0	0	0	1	1	0
3	2	2	1	1	1	0	1	0	1	0	1	1	0
3	2	2	1	1	1	0	1	0	1	0	0	0	0
3	2	2	1	1	1	0	1	1	0	0	0	0	1
3	2	2	1	1	0	0	0	1	0	1	0	0	1

LIN-3	MPK-1	LIN-39	LS	LIN-12m	LIN-12i	CKI-1	EFL-1	LIN-35	SCF	APC	CDK-4/CYD-1	CDK-2/CYE-1	CDK-1/CYB-3
1	0	1	0	1	0	1	0	1	0	1	0	0	1
1	1	1	0	1	0	1	0	1	0	1	0	0	0
1	1	1	0	1	1	0	0	1	0	0	0	0	0
1	0	1	0	1	1	0	0	1	0	0	1	0	0
1	0	1	0	1	1	0	0	0	0	0	1	0	0
1	0	1	0	1	1	0	1	0	0	0	1	0	0
1	0	1	0	1	1	0	1	0	0	0	1	1	0
1	0	1	0	1	1	0	1	0	1	0	1	1	0
1	0	1	0	1	1	0	1	0	1	0	0	0	0
1	0	1	0	1	1	0	1	1	0	0	0	0	1

LIN-3	MPK-1	LIN-39	LS	LIN-12m	LIN-12i	CKI-1	EFL-1	LIN-35	SCF	APC	CDK-4/CYD-1	CDK-2/CYE-1	CDK-1/CYB-3
1	0	1	1	1	0	1	0	1	0	1	0	0	1
1	1	1	1	1	0	1	0	1	0	1	0	0	0
1	1	1	1	1	1	0	0	1	0	0	0	0	0
1	0	1	1	1	1	0	0	1	0	0	1	0	0
1	0	1	1	1	1	0	0	0	0	0	1	0	0
1	0	1	1	1	1	0	1	0	0	0	1	0	0
1	0	1	1	1	1	0	1	0	0	0	1	1	0
1	0	1	1	1	1	0	1	0	1	0	1	1	0
1	0	1	1	1	1	0	1	0	1	0	0	0	0
1	0	1	1	1	1	0	1	1	0	0	0	0	1

LIN-3	MPK-1	LIN-39	LS	LIN-12m	LIN-12i	CKI-1	EFL-1	LIN-35	SCF	APC	CDK-4/CYD-1	CDK-2/CYE-1	CDK-1/CYB-3
0	0	1	0	1	0	0	0	0	0	0	1	0	0
0	0	1	0	1	0	0	1	0	0	0	1	0	0
0	0	1	0	1	0	0	1	0	0	0	1	1	0
0	0	1	0	1	0	0	1	0	1	0	1	1	0
0	0	1	0	1	0	0	1	0	1	0	0	0	0
0	0	1	0	1	0	0	1	1	0	0	0	0	1
0	0	1	0	1	0	1	0	1	0	1	0	0	1
0	0	1	0	1	0	1	0	1	0	1	0	0	0
0	0	1	0	1	0	1	0	1	0	0	0	0	0
0	0	1	0	1	0	0	0	1	0	0	0	0	0
0	0	1	0	1	0	0	0	1	0	0	1	0	0

LIN-3	MPK-1	LIN-39	LS	LIN-12m	LIN-12i	CKI-1	EFL-1	LIN-35	SCF	APC	CDK-4/CYD-1	CDK-2/CYE-1	CDK-1/CYB-3
0	0	1	1	1	0	1	0	1	0	1	0	0	0
0	0	1	1	1	1	1	0	1	0	0	0	0	0
0	0	1	1	1	1	0	0	1	0	0	0	0	0
0	0	1	1	1	1	0	0	1	0	0	1	0	0
0	0	1	1	1	1	0	0	0	0	0	1	0	0
0	0	1	1	1	1	0	1	0	0	0	1	0	0
0	0	1	1	1	1	0	1	0	0	0	1	1	0
0	0	1	1	1	1	0	1	0	1	0	1	1	0
0	0	1	1	1	1	0	1	0	1	0	0	0	0
0	0	1	1	1	1	0	1	1	0	0	0	0	1
0	0	1	1	1	0	1	0	1	0	1	0	0	1

LIN-39 to LIN-12m

LIN-3	MPK-1	LIN-39	LS	LIN-12m	LIN-12i	CKI-1	EFL-1	LIN-35	SCF	APC	CDK-4/CYD-1	CDK-2/CYE-1	CDK-1/CYB-3
2	2	2	0	0	0	0	0	0	0	0	1	0	0
2	2	2	0	0	0	0	1	0	0	0	1	0	0
2	2	2	0	0	0	0	1	0	0	0	1	1	0
2	2	2	0	0	0	0	1	0	1	0	1	1	0
2	2	2	0	0	0	0	1	0	1	0	0	0	0
2	2	2	0	0	0	0	1	1	0	0	0	0	1
2	2	2	0	0	0	0	0	1	0	1	0	0	1
2	2	2	0	0	0	0	0	1	0	1	0	0	0
2	2	2	0	0	0	0	0	1	0	0	1	0	0

LIN-3	MPK-1	LIN-39	LS	LIN-12m	LIN-12i	CKI-1	EFL-1	LIN-35	SCF	APC	CDK-4/CYD-1	CDK-2/CYE-1	CDK-1/CYB-3
2	2	2	1	0	0	0	0	0	0	0	1	0	0
2	2	2	1	0	0	0	1	0	0	0	1	0	0
2	2	2	1	0	0	0	1	0	0	0	1	1	0
2	2	2	1	0	0	0	1	0	1	0	1	1	0
2	2	2	1	0	0	0	1	0	1	0	0	0	0
2	2	2	1	0	0	0	1	1	0	0	0	0	1
2	2	2	1	0	0	0	0	1	0	1	0	0	1
2	2	2	1	0	0	0	0	1	0	1	0	0	0
2	2	2	1	0	0	0	0	1	0	0	1	0	0

LIN-3	MPK-1	LIN-39	LS	LIN-12m	LIN-12i	CKI-1	EFL-1	LIN-35	SCF	APC	CDK-4/CYD-1	CDK-2/CYE-1	CDK-1/CYB-3
3	2	2	0	0	0	0	0	0	0	0	1	0	0
3	2	2	0	0	0	0	1	0	0	0	1	0	0
3	2	2	0	0	0	0	1	0	0	0	1	1	0
3	2	2	0	0	0	0	1	0	1	0	1	1	0
3	2	2	0	0	0	0	1	0	1	0	0	0	0
3	2	2	0	0	0	0	1	1	0	0	0	0	1
3	2	2	0	0	0	0	0	1	0	1	0	0	1
3	2	2	0	0	0	0	0	1	0	1	0	0	0
3	2	2	0	0	0	0	0	1	0	0	1	0	0

LIN-3	MPK-1	LIN-39	LS	LIN-12m	LIN-12i	CKI-1	EFL-1	LIN-35	SCF	APC	CDK-4/CYD-1	CDK-2/CYE-1	CDK-1/CYB-3
3	2	2	1	0	0	0	0	0	0	0	1	0	0
3	2	2	1	0	0	0	1	0	0	0	1	0	0
3	2	2	1	0	0	0	1	0	0	0	1	1	0
3	2	2	1	0	0	0	1	0	1	0	1	1	0
3	2	2	1	0	0	0	1	0	1	0	0	0	0
3	2	2	1	0	0	0	1	1	0	0	0	0	1
3	2	2	1	0	0	0	0	1	0	1	0	0	1
3	2	2	1	0	0	0	0	1	0	1	0	0	0
3	2	2	1	0	0	0	0	1	0	0	1	0	0

LIN-3	MPK-1	LIN-39	LS	LIN-12m	LIN-12i	CKI-1	EFL-1	LIN-35	SCF	APC	CDK-4/CYD-1	CDK-2/CYE-1	CDK-1/CYB-3
1	0	1	0	1	0	1	0	1	0	1	0	0	1
1	1	1	0	0	0	1	0	1	0	1	0	0	0
1	1	1	0	0	1	0	0	1	0	0	0	0	0
1	0	1	0	1	1	0	0	1	0	0	1	0	0
1	0	1	0	1	1	0	0	0	0	0	1	0	0
1	0	1	0	1	1	0	1	0	0	0	1	0	0
1	0	1	0	1	1	0	1	0	0	0	1	1	0
1	0	1	0	1	1	0	1	0	1	0	1	1	0
1	0	1	0	1	1	0	1	0	1	0	0	0	0
1	0	1	0	1	1	0	1	1	0	0	0	0	1

LIN-3	MPK-1	LIN-39	LS	LIN-12m	LIN-12i	CKI-1	EFL-1	LIN-35	SCF	APC	CDK-4/CYD-1	CDK-2/CYE-1	CDK-1/CYB-3
1	0	1	1	1	0	1	0	1	0	1	0	0	1
1	1	1	1	0	0	1	0	1	0	1	0	0	0

1	1	1	1	0	1	0	0	1	0	0	0	0	0
1	0	1	1	1	1	0	0	1	0	0	1	0	0
1	0	1	1	1	1	0	0	0	0	0	1	0	0
1	0	1	1	1	1	0	1	0	0	0	1	0	0
1	0	1	1	1	1	0	1	0	0	0	1	1	0
1	0	1	1	1	1	0	1	0	1	0	1	1	0
1	0	1	1	1	1	0	1	0	1	0	0	0	0
1	0	1	1	1	1	0	1	1	0	0	0	0	1

LIN-3	MPK-1	LIN-39	LS	LIN-12m	LIN-12i	CKI-1	EFL-1	LIN-35	SCF	APC	CDK-4/CYD-1	CDK-2/CYE-1	CDK-1/CYB-3
0	0	1	0	0	0	0	0	0	0	0	1	0	0
0	0	1	0	0	0	0	1	0	0	0	1	0	0
0	0	1	0	0	0	0	1	0	0	0	1	1	0
0	0	1	0	0	0	0	1	0	1	0	1	1	0
0	0	1	0	0	0	0	1	0	1	0	0	0	0
0	0	1	0	0	0	0	1	1	0	0	0	0	1
0	0	1	0	0	0	1	0	1	0	1	0	0	1
0	0	1	0	0	0	1	0	1	0	1	0	0	0
0	0	1	0	0	0	1	0	1	0	0	0	0	0
0	0	1	0	0	0	0	0	1	0	0	0	0	0
0	0	1	0	0	0	0	0	1	0	0	1	0	0

LIN-3	MPK-1	LIN-39	LS	LIN-12m	LIN-12i	CKI-1	EFL-1	LIN-35	SCF	APC	CDK-4/CYD-1	CDK-2/CYE-1	CDK-1/CYB-3
0	0	1	1	0	0	0	0	0	0	0	1	0	0
0	0	1	1	0	0	0	1	0	0	0	1	0	0
0	0	1	1	0	0	0	1	0	0	0	1	1	0
0	0	1	1	0	0	0	1	0	1	0	1	1	0
0	0	1	1	0	0	0	1	0	1	0	0	0	0
0	0	1	1	0	0	0	1	1	0	0	0	0	1
0	0	1	1	0	0	1	0	1	0	1	0	0	1
0	0	1	1	0	0	1	0	1	0	1	0	0	0
0	0	1	1	0	0	1	0	1	0	0	0	0	0
0	0	1	1	0	0	0	0	1	0	0	0	0	0
0	0	1	1	0	0	0	0	1	0	0	1	0	0

LIN-12i to LIN-12m

LIN-3	MPK-1	LIN-39	LS	LIN-12m	LIN-12i	CKI-1	EFL-1	LIN-35	SCF	APC	CDK-4/CYD-1	CDK-2/CYE-1	CDK-1/CYB-3
2	1	1	1	1	0	0	0	1	0	1	0	0	1
2	2	1	1	1	0	0	0	1	0	1	0	0	0
2	2	2	1	0	1	0	0	1	0	0	1	0	0
2	1	2	1	1	1	0	0	0	0	0	1	0	0
2	1	1	1	1	1	0	1	0	0	0	1	0	0
2	1	1	1	1	1	0	1	0	0	0	1	1	0
2	1	1	1	1	1	0	1	0	1	0	1	1	0
2	1	1	1	1	1	0	1	0	1	0	0	0	0
2	1	1	1	1	1	0	1	1	0	0	0	0	1

LIN-3	MPK-1	LIN-39	LS	LIN-12m	LIN-12i	CKI-1	EFL-1	LIN-35	SCF	APC	CDK-4/CYD-1	CDK-2/CYE-1	CDK-1/CYB-3
2	2	2	0	0	0	0	0	1	0	0	1	0	0
2	2	2	0	1	0	0	0	0	0	0	1	0	0
2	2	2	0	1	0	0	1	0	0	0	1	0	0
2	2	2	0	1	0	0	1	0	0	0	1	1	0
2	2	2	0	1	0	0	1	0	1	0	1	1	0
2	2	2	0	1	0	0	1	0	1	0	0	0	0
2	2	2	0	0	0	0	1	1	0	0	0	0	1
2	2	2	0	0	0	0	0	1	0	1	0	0	1
2	2	2	0	0	0	0	0	1	0	1	0	0	0

LIN-3	MPK-1	LIN-39	LS	LIN-12m	LIN-12i	CKI-1	EFL-1	LIN-35	SCF	APC	CDK-4/CYD-1	CDK-2/CYE-1	CDK-1/CYB-3
3	2	2	0	0	0	0	0	1	0	0	1	0	0

3	2	2	0	1	0	0	0	0	0	0	1	0	0
3	2	2	0	1	0	0	1	0	0	0	1	0	0
3	2	2	0	1	0	0	1	0	0	0	1	1	0
3	2	2	0	1	0	0	1	0	1	0	1	1	0
3	2	2	0	1	0	0	1	0	1	0	0	0	0
3	2	2	0	0	0	0	1	1	0	0	0	0	1
3	2	2	0	0	0	0	0	1	0	1	0	0	1
3	2	2	0	0	0	0	0	1	0	1	0	0	0

LIN-3	MPK-1	LIN-39	LS	LIN-12m	LIN-12i	CKI-1	EFL-1	LIN-35	SCF	APC	CDK-4/CYD-1	CDK-2/CYE-1	CDK-1/CYB-3
3	2	2	1	0	0	0	0	1	0	0	1	0	0
3	2	2	1	1	0	0	0	0	0	0	1	0	0
3	2	2	1	1	1	0	1	0	0	0	1	0	0
3	2	2	1	1	1	0	1	0	0	0	1	1	0
3	2	2	1	1	1	0	1	0	1	0	1	1	0
3	2	2	1	1	1	0	1	0	1	0	0	0	0
3	2	2	1	0	1	0	1	1	0	0	0	0	1
3	2	2	1	0	0	0	0	1	0	1	0	0	1
3	2	2	1	0	0	0	0	1	0	1	0	0	0

LIN-3	MPK-1	LIN-39	LS	LIN-12m	LIN-12i	CKI-1	EFL-1	LIN-35	SCF	APC	CDK-4/CYD-1	CDK-2/CYE-1	CDK-1/CYB-3
1	0	1	0	1	0	1	0	1	0	1	0	0	1
1	1	1	0	1	0	1	0	1	0	1	0	0	0
1	1	1	0	1	1	0	0	1	0	0	0	0	0
1	0	1	0	1	1	0	0	1	0	0	1	0	0
1	0	1	0	1	1	0	0	0	0	0	1	0	0
1	0	1	0	1	1	0	1	0	0	0	1	0	0
1	0	1	0	1	1	0	1	0	0	0	1	1	0
1	0	1	0	1	1	0	1	0	1	0	1	1	0
1	0	1	0	1	1	0	1	0	1	0	0	0	0
1	0	1	0	1	1	0	1	1	0	0	0	0	1

LIN-3	MPK-1	LIN-39	LS	LIN-12m	LIN-12i	CKI-1	EFL-1	LIN-35	SCF	APC	CDK-4/CYD-1	CDK-2/CYE-1	CDK-1/CYB-3
1	0	1	1	1	0	1	0	1	0	1	0	0	1
1	1	1	1	1	0	1	0	1	0	1	0	0	0
1	1	1	1	1	1	0	0	1	0	0	0	0	0
1	0	1	1	1	1	0	0	1	0	0	1	0	0
1	0	1	1	1	1	0	0	0	0	0	1	0	0
1	0	1	1	1	1	0	1	0	0	0	1	0	0
1	0	1	1	1	1	0	1	0	0	0	1	1	0
1	0	1	1	1	1	0	1	0	1	0	1	1	0
1	0	1	1	1	1	0	1	0	1	0	0	0	0
1	0	1	1	1	1	0	1	1	0	0	0	0	1

LIN-3	MPK-1	LIN-39	LS	LIN-12m	LIN-12i	CKI-1	EFL-1	LIN-35	SCF	APC	CDK-4/CYD-1	CDK-2/CYE-1	CDK-1/CYB-3
0	0	1	0	1	0	0	0	0	0	0	1	0	0
0	0	1	0	1	0	0	1	0	0	0	1	0	0
0	0	1	0	1	0	0	1	0	0	0	1	1	0
0	0	1	0	1	0	0	1	0	1	0	1	1	0
0	0	1	0	1	0	0	1	0	1	0	0	0	0
0	0	1	0	1	0	0	1	1	0	0	0	0	1
0	0	1	0	1	0	1	0	1	0	1	0	0	1
0	0	1	0	1	0	1	0	1	0	1	0	0	0
0	0	1	0	1	0	1	0	1	0	0	0	0	0
0	0	1	0	1	0	0	0	1	0	0	0	0	0
0	0	1	0	1	0	0	0	1	0	0	1	0	0

LIN-3	MPK-1	LIN-39	LS	LIN-12m	LIN-12i	CKI-1	EFL-1	LIN-35	SCF	APC	CDK-4/CYD-1	CDK-2/CYE-1	CDK-1/CYB-3
0	0	1	1	1	0	1	0	1	0	1	0	0	0
0	0	1	1	1	1	1	0	1	0	0	0	0	0
0	0	1	1	1	1	0	0	1	0	0	0	0	0

0	0	1	1	1	1	0	0	1	0	0	1	0	0
0	0	1	1	1	1	0	0	0	0	0	1	0	0
0	0	1	1	1	1	0	1	0	0	0	1	0	0
0	0	1	1	1	1	0	1	0	0	0	1	1	0
0	0	1	1	1	1	0	1	0	1	0	1	1	0
0	0	1	1	1	1	0	1	0	1	0	0	0	0
0	0	1	1	1	1	0	1	1	0	0	0	0	1
0	0	1	1	1	0	1	0	1	0	1	0	0	1

CDK-4/CYD-1 to LIN-12m

LIN-3	MPK-1	LIN-39	LS	LIN-12m	LIN-12i	CKI-1	EFL-1	LIN-35	SCF	APC	CDK-4/CYD-1	CDK-2/CYE-1	CDK-1/CYB-3
2	1	1	1	1	0	0	0	1	0	1	0	0	1
2	2	1	1	1	0	0	0	1	0	1	0	0	0
2	2	2	1	0	1	0	0	1	0	0	1	0	0
2	1	2	1	0	1	0	0	0	0	0	1	0	0
2	1	1	1	1	1	0	1	0	0	0	1	0	0
2	1	1	1	1	1	0	1	0	0	0	1	1	0
2	1	1	1	1	1	0	1	0	1	0	1	1	0
2	1	1	1	1	1	0	1	0	1	0	0	0	0
2	1	1	1	1	1	0	1	1	0	0	0	0	1

LIN-3	MPK-1	LIN-39	LS	LIN-12m	LIN-12i	CKI-1	EFL-1	LIN-35	SCF	APC	CDK-4/CYD-1	CDK-2/CYE-1	CDK-1/CYB-3
2	2	2	0	0	0	0	0	0	0	0	1	0	0
2	2	2	0	0	0	0	1	0	0	0	1	0	0
2	2	2	0	0	0	0	1	0	0	0	1	1	0
2	2	2	0	0	0	0	1	0	1	0	1	1	0
2	2	2	0	0	0	0	1	0	1	0	0	0	0
2	2	2	0	0	0	0	1	1	0	0	0	0	1
2	2	2	0	0	0	0	0	1	0	1	0	0	1
2	2	2	0	0	0	0	0	1	0	1	0	0	0
2	2	2	0	0	0	0	0	1	0	0	1	0	0

LIN-3	MPK-1	LIN-39	LS	LIN-12m	LIN-12i	CKI-1	EFL-1	LIN-35	SCF	APC	CDK-4/CYD-1	CDK-2/CYE-1	CDK-1/CYB-3
2	2	2	1	0	0	0	0	0	0	0	1	0	0
2	2	2	1	0	0	0	1	0	0	0	1	0	0
2	2	2	1	0	0	0	1	0	0	0	1	1	0
2	2	2	1	0	0	0	1	0	1	0	1	1	0
2	2	2	1	0	0	0	1	0	1	0	0	0	0
2	2	2	1	0	0	0	1	1	0	0	0	0	1
2	2	2	1	0	0	0	0	1	0	1	0	0	1
2	2	2	1	0	0	0	0	1	0	1	0	0	0
2	2	2	1	0	0	0	0	1	0	0	1	0	0

LIN-3	MPK-1	LIN-39	LS	LIN-12m	LIN-12i	CKI-1	EFL-1	LIN-35	SCF	APC	CDK-4/CYD-1	CDK-2/CYE-1	CDK-1/CYB-3
3	2	2	0	0	0	0	0	0	0	0	1	0	0
3	2	2	0	0	0	0	1	0	0	0	1	0	0
3	2	2	0	0	0	0	1	0	0	0	1	1	0
3	2	2	0	0	0	0	1	0	1	0	1	1	0
3	2	2	0	0	0	0	1	0	1	0	0	0	0
3	2	2	0	0	0	0	1	1	0	0	0	0	1
3	2	2	0	0	0	0	0	1	0	1	0	0	1
3	2	2	0	0	0	0	0	1	0	1	0	0	0
3	2	2	0	0	0	0	0	1	0	0	1	0	0

LIN-3	MPK-1	LIN-39	LS	LIN-12m	LIN-12i	CKI-1	EFL-1	LIN-35	SCF	APC	CDK-4/CYD-1	CDK-2/CYE-1	CDK-1/CYB-3
3	2	2	1	0	0	0	0	0	0	0	1	0	0
3	2	2	1	0	0	0	1	0	0	0	1	0	0
3	2	2	1	0	0	0	1	0	0	0	1	1	0
3	2	2	1	0	0	0	1	0	1	0	1	1	0
3	2	2	1	0	0	0	1	0	1	0	0	0	0

3	2	2	1	0	0	0	1	1	0	0	0	0	1
3	2	2	1	0	0	0	0	1	0	1	0	0	1
3	2	2	1	0	0	0	0	1	0	1	0	0	0
3	2	2	1	0	0	0	0	1	0	0	1	0	0

LIN-3	MPK-1	LIN-39	LS	LIN-12m	LIN-12i	CKI-1	EFL-1	LIN-35	SCF	APC	CDK-4/CYD-1	CDK-2/CYE-1	CDK-1/CYB-3
1	0	1	0	1	0	1	0	1	0	1	0	0	1
1	1	1	0	1	0	1	0	1	0	1	0	0	0
1	1	1	0	1	1	0	0	1	0	0	0	0	0
1	0	1	0	1	1	0	0	1	0	0	1	0	0
1	0	1	0	1	1	0	0	0	0	0	1	0	0
1	0	1	0	1	1	0	1	0	0	0	1	0	0
1	0	1	0	1	1	0	1	0	0	0	1	1	0
1	0	1	0	1	1	0	1	0	1	0	1	1	0
1	0	1	0	1	1	0	1	0	1	0	0	0	0
1	0	1	0	1	1	0	1	1	0	0	0	0	1

LIN-3	MPK-1	LIN-39	LS	LIN-12m	LIN-12i	CKI-1	EFL-1	LIN-35	SCF	APC	CDK-4/CYD-1	CDK-2/CYE-1	CDK-1/CYB-3
1	0	1	1	1	0	1	0	1	0	1	0	0	1
1	1	1	1	1	0	1	0	1	0	1	0	0	0
1	1	1	1	1	1	0	0	1	0	0	0	0	0
1	0	1	1	1	1	0	0	1	0	0	1	0	0
1	0	1	1	1	1	0	0	0	0	0	1	0	0
1	0	1	1	1	1	0	1	0	0	0	1	0	0
1	0	1	1	1	1	0	1	0	0	0	1	1	0
1	0	1	1	1	1	0	1	0	1	0	1	1	0
1	0	1	1	1	1	0	1	0	1	0	0	0	0
1	0	1	1	1	1	0	1	1	0	0	0	0	1

LIN-3	MPK-1	LIN-39	LS	LIN-12m	LIN-12i	CKI-1	EFL-1	LIN-35	SCF	APC	CDK-4/CYD-1	CDK-2/CYE-1	CDK-1/CYB-3
0	0	1	0	1	0	0	0	0	0	0	1	0	0
0	0	1	0	1	0	0	1	0	0	0	1	0	0
0	0	1	0	1	0	0	1	0	0	0	1	1	0
0	0	1	0	1	0	0	1	0	1	0	1	1	0
0	0	1	0	1	0	0	1	0	1	0	0	0	0
0	0	1	0	1	0	0	1	1	0	0	0	0	1
0	0	1	0	1	0	1	0	1	0	1	0	0	1
0	0	1	0	1	0	1	0	1	0	1	0	0	0
0	0	1	0	1	0	1	0	1	0	0	0	0	0
0	0	1	0	1	0	0	0	1	0	0	0	0	0
0	0	1	0	1	0	0	0	1	0	0	1	0	0

LIN-3	MPK-1	LIN-39	LS	LIN-12m	LIN-12i	CKI-1	EFL-1	LIN-35	SCF	APC	CDK-4/CYD-1	CDK-2/CYE-1	CDK-1/CYB-3
0	0	1	1	1	0	1	0	1	0	1	0	0	0
0	0	1	1	1	1	1	0	1	0	0	0	0	0
0	0	1	1	1	1	0	0	1	0	0	0	0	0
0	0	1	1	1	1	0	0	1	0	0	1	0	0
0	0	1	1	1	1	0	0	0	0	0	1	0	0
0	0	1	1	1	1	0	1	0	0	0	1	0	0
0	0	1	1	1	1	0	1	0	0	0	1	1	0
0	0	1	1	1	1	0	1	0	1	0	1	1	0
0	0	1	1	1	1	0	1	0	1	0	0	0	0
0	0	1	1	1	1	0	1	1	0	0	0	0	1
0	0	1	1	1	0	1	0	1	0	1	0	0	1

LIN-3 to LIN-12i

LIN-3	MPK-1	LIN-39	LS	LIN-12m	LIN-12i	CKI-1	EFL-1	LIN-35	SCF	APC	CDK-4/CYD-1	CDK-2/CYE-1	CDK-1/CYB-3
1	1	1	0	1	0	0	0	0	0	0	1	0	0
1	1	1	0	1	0	0	1	0	0	0	1	0	0
1	1	1	0	1	0	0	1	0	0	0	1	1	0

1	1	1	0	1	0	0	1	0	1	0	1	1	0
1	1	1	0	1	0	0	1	0	1	0	0	0	0
1	1	1	0	1	0	0	1	1	0	0	0	0	1
1	1	1	0	1	0	0	0	1	0	1	0	0	1
1	1	1	0	1	0	0	0	1	0	1	0	0	0
1	1	1	0	1	0	0	0	1	0	0	1	0	0

LIN-3	MPK-1	LIN-39	LS	LIN-12m	LIN-12i	CKI-1	EFL-1	LIN-35	SCF	APC	CDK-4/CYD-1	CDK-2/CYE-1	CDK-1/CYB-3
2	1	1	1	1	0	0	0	1	0	1	0	0	1
2	2	1	1	1	0	0	0	1	0	1	0	0	0
2	2	2	1	0	1	0	0	1	0	0	1	0	0
2	1	2	1	1	1	0	0	0	0	0	1	0	0
2	1	1	1	1	1	0	1	0	0	0	1	0	0
2	1	1	1	1	1	0	1	0	0	0	1	1	0
2	1	1	1	1	1	0	1	0	1	0	1	1	0
2	1	1	1	1	1	0	1	0	1	0	0	0	0
2	1	1	1	1	1	0	1	1	0	0	0	0	1

LIN-3	MPK-1	LIN-39	LS	LIN-12m	LIN-12i	CKI-1	EFL-1	LIN-35	SCF	APC	CDK-4/CYD-1	CDK-2/CYE-1	CDK-1/CYB-3
2	2	2	0	0	0	0	0	1	0	0	1	0	0
2	2	2	0	1	0	0	0	0	0	0	1	0	0
2	2	2	0	1	0	0	1	0	0	0	1	0	0
2	2	2	0	1	0	0	1	0	0	0	1	1	0
2	2	2	0	1	0	0	1	0	1	0	1	1	0
2	2	2	0	1	0	0	1	0	1	0	0	0	0
2	2	2	0	0	0	0	1	1	0	0	0	0	1
2	2	2	0	0	0	0	0	1	0	1	0	0	1
2	2	2	0	0	0	0	0	1	0	1	0	0	0

LIN-3	MPK-1	LIN-39	LS	LIN-12m	LIN-12i	CKI-1	EFL-1	LIN-35	SCF	APC	CDK-4/CYD-1	CDK-2/CYE-1	CDK-1/CYB-3
3	2	2	0	0	0	0	0	1	0	0	1	0	0
3	2	2	0	1	0	0	0	0	0	0	1	0	0
3	2	2	0	1	0	0	1	0	0	0	1	0	0
3	2	2	0	1	0	0	1	0	0	0	1	1	0
3	2	2	0	1	0	0	1	0	1	0	1	1	0
3	2	2	0	1	0	0	1	0	1	0	0	0	0
3	2	2	0	0	0	0	1	1	0	0	0	0	1
3	2	2	0	0	0	0	0	1	0	1	0	0	1
3	2	2	0	0	0	0	0	1	0	1	0	0	0

LIN-3	MPK-1	LIN-39	LS	LIN-12m	LIN-12i	CKI-1	EFL-1	LIN-35	SCF	APC	CDK-4/CYD-1	CDK-2/CYE-1	CDK-1/CYB-3
3	2	2	1	0	0	0	0	1	0	0	1	0	0
3	2	2	1	1	0	0	0	0	0	0	1	0	0
3	2	2	1	1	1	0	1	0	0	0	1	0	0
3	2	2	1	1	1	0	1	0	0	0	1	1	0
3	2	2	1	1	1	0	1	0	1	0	1	1	0
3	2	2	1	1	1	0	1	0	1	0	0	0	0
3	2	2	1	0	1	0	1	1	0	0	0	0	1
3	2	2	1	0	0	0	0	1	0	1	0	0	1
3	2	2	1	0	0	0	0	1	0	1	0	0	0

LIN-3	MPK-1	LIN-39	LS	LIN-12m	LIN-12i	CKI-1	EFL-1	LIN-35	SCF	APC	CDK-4/CYD-1	CDK-2/CYE-1	CDK-1/CYB-3
1	0	1	1	1	0	1	0	1	0	1	0	0	1
1	1	1	1	1	0	1	0	1	0	1	0	0	0
1	1	1	1	1	1	0	0	1	0	0	0	0	0
1	0	1	1	1	1	0	0	1	0	0	1	0	0
1	0	1	1	1	1	0	0	0	0	0	1	0	0
1	0	1	1	1	1	0	1	0	0	0	1	0	0
1	0	1	1	1	1	0	1	0	0	0	1	1	0
1	0	1	1	1	1	0	1	0	1	0	1	1	0
1	0	1	1	1	1	0	1	0	1	0	0	0	0

1	0	1	1	1	1	0	1	1	0	0	0	0	1

LIN-3	MPK-1	LIN-39	LS	LIN-12m	LIN-12i	CKI-1	EFL-1	LIN-35	SCF	APC	CDK-4/CYD-1	CDK-2/CYE-1	CDK-1/CYB-3
0	0	1	0	1	0	0	0	0	0	0	1	0	0
0	0	1	0	1	0	0	1	0	0	0	1	0	0
0	0	1	0	1	0	0	1	0	0	0	1	1	0
0	0	1	0	1	0	0	1	0	1	0	1	1	0
0	0	1	0	1	0	0	1	0	1	0	0	0	0
0	0	1	0	1	0	0	1	1	0	0	0	0	1
0	0	1	0	1	0	1	0	1	0	1	0	0	1
0	0	1	0	1	0	1	0	1	0	1	0	0	0
0	0	1	0	1	0	1	0	1	0	0	0	0	0
0	0	1	0	1	0	0	0	1	0	0	0	0	0
0	0	1	0	1	0	0	0	1	0	0	1	0	0

LIN-3	MPK-1	LIN-39	LS	LIN-12m	LIN-12i	CKI-1	EFL-1	LIN-35	SCF	APC	CDK-4/CYD-1	CDK-2/CYE-1	CDK-1/CYB-3
0	0	1	1	1	0	1	0	1	0	1	0	0	0
0	0	1	1	1	1	1	0	1	0	0	0	0	0
0	0	1	1	1	1	0	0	1	0	0	0	0	0
0	0	1	1	1	1	0	0	1	0	0	1	0	0
0	0	1	1	1	1	0	0	0	0	0	1	0	0
0	0	1	1	1	1	0	1	0	0	0	1	0	0
0	0	1	1	1	1	0	1	0	0	0	1	1	0
0	0	1	1	1	1	0	1	0	1	0	1	1	0
0	0	1	1	1	1	0	1	0	1	0	0	0	0
0	0	1	1	1	1	0	1	1	0	0	0	0	1
0	0	1	1	1	0	1	0	1	0	1	0	0	1

LS to LIN-12i

LIN-3	MPK-1	LIN-39	LS	LIN-12m	LIN-12i	CKI-1	EFL-1	LIN-35	SCF	APC	CDK-4/CYD-1	CDK-2/CYE-1	CDK-1/CYB-3
2	2	2	0	0	0	0	0	1	0	0	1	0	0
2	2	2	0	1	0	0	0	0	0	0	1	0	0
2	2	2	0	1	0	0	1	0	0	0	1	0	0
2	2	2	0	1	0	0	1	0	0	0	1	1	0
2	2	2	0	1	0	0	1	0	1	0	1	1	0
2	2	2	0	1	0	0	1	0	1	0	0	0	0
2	2	2	0	0	0	0	1	1	0	0	0	0	1
2	2	2	0	0	0	0	0	1	0	1	0	0	1
2	2	2	0	0	0	0	0	1	0	1	0	0	0

LIN-3	MPK-1	LIN-39	LS	LIN-12m	LIN-12i	CKI-1	EFL-1	LIN-35	SCF	APC	CDK-4/CYD-1	CDK-2/CYE-1	CDK-1/CYB-3
2	2	2	1	0	0	0	0	1	0	0	1	0	0
2	2	2	1	1	0	0	0	0	0	0	1	0	0
2	2	2	1	1	0	0	1	0	0	0	1	0	0
2	2	2	1	1	0	0	1	0	0	0	1	1	0
2	2	2	1	1	0	0	1	0	1	0	1	1	0
2	2	2	1	1	0	0	1	0	1	0	0	0	0
2	2	2	1	0	0	0	1	1	0	0	0	0	1
2	2	2	1	0	0	0	0	1	0	1	0	0	1
2	2	2	1	0	0	0	0	1	0	1	0	0	0

LIN-3	MPK-1	LIN-39	LS	LIN-12m	LIN-12i	CKI-1	EFL-1	LIN-35	SCF	APC	CDK-4/CYD-1	CDK-2/CYE-1	CDK-1/CYB-3
3	2	2	0	0	0	0	0	1	0	0	1	0	0
3	2	2	0	1	0	0	0	0	0	0	1	0	0
3	2	2	0	1	0	0	1	0	0	0	1	0	0
3	2	2	0	1	0	0	1	0	0	0	1	1	0
3	2	2	0	1	0	0	1	0	1	0	1	1	0
3	2	2	0	1	0	0	1	0	1	0	0	0	0
3	2	2	0	0	0	0	1	1	0	0	0	0	1
3	2	2	0	0	0	0	0	1	0	1	0	0	1

3	2	2	0	0	0	0	0	1	0	1	0	0	0

LIN-3	MPK-1	LIN-39	LS	LIN-12m	LIN-12i	CKI-1	EFL-1	LIN-35	SCF	APC	CDK-4/CYD-1	CDK-2/CYE-1	CDK-1/CYB-3
3	2	2	1	0	0	0	0	1	0	0	1	0	0
3	2	2	1	1	0	0	0	0	0	0	1	0	0
3	2	2	1	1	0	0	1	0	0	0	1	0	0
3	2	2	1	1	0	0	1	0	0	0	1	1	0
3	2	2	1	1	0	0	1	0	1	0	1	1	0
3	2	2	1	1	0	0	1	0	1	0	0	0	0
3	2	2	1	0	0	0	1	1	0	0	0	0	1
3	2	2	1	0	0	0	0	1	0	1	0	0	1
3	2	2	1	0	0	0	0	1	0	1	0	0	0

LIN-3	MPK-1	LIN-39	LS	LIN-12m	LIN-12i	CKI-1	EFL-1	LIN-35	SCF	APC	CDK-4/CYD-1	CDK-2/CYE-1	CDK-1/CYB-3
1	0	1	0	1	0	1	0	1	0	1	0	0	1
1	1	1	0	1	0	1	0	1	0	1	0	0	0
1	1	1	0	1	1	0	0	1	0	0	0	0	0
1	0	1	0	1	1	0	0	1	0	0	1	0	0
1	0	1	0	1	1	0	0	0	0	0	1	0	0
1	0	1	0	1	1	0	1	0	0	0	1	0	0
1	0	1	0	1	1	0	1	0	0	0	1	1	0
1	0	1	0	1	1	0	1	0	1	0	1	1	0
1	0	1	0	1	1	0	1	0	1	0	0	0	0
1	0	1	0	1	1	0	1	1	0	0	0	0	1

LIN-3	MPK-1	LIN-39	LS	LIN-12m	LIN-12i	CKI-1	EFL-1	LIN-35	SCF	APC	CDK-4/CYD-1	CDK-2/CYE-1	CDK-1/CYB-3
1	0	1	1	1	0	1	0	1	0	1	0	0	1
1	1	1	1	1	0	1	0	1	0	1	0	0	0
1	1	1	1	1	1	0	0	1	0	0	0	0	0
1	0	1	1	1	1	0	0	1	0	0	1	0	0
1	0	1	1	1	1	0	0	0	0	0	1	0	0
1	0	1	1	1	1	0	1	0	0	0	1	0	0
1	0	1	1	1	1	0	1	0	0	0	1	1	0
1	0	1	1	1	1	0	1	0	1	0	1	1	0
1	0	1	1	1	1	0	1	0	1	0	0	0	0
1	0	1	1	1	1	0	1	1	0	0	0	0	1

LIN-3	MPK-1	LIN-39	LS	LIN-12m	LIN-12i	CKI-1	EFL-1	LIN-35	SCF	APC	CDK-4/CYD-1	CDK-2/CYE-1	CDK-1/CYB-3
0	0	1	0	1	0	0	0	0	0	0	1	0	0
0	0	1	0	1	0	0	1	0	0	0	1	0	0
0	0	1	0	1	0	0	1	0	0	0	1	1	0
0	0	1	0	1	0	0	1	0	1	0	1	1	0
0	0	1	0	1	0	0	1	0	1	0	0	0	0
0	0	1	0	1	0	0	1	1	0	0	0	0	1
0	0	1	0	1	0	1	0	1	0	1	0	0	1
0	0	1	0	1	0	1	0	1	0	1	0	0	0
0	0	1	0	1	0	1	0	1	0	0	0	0	0
0	0	1	0	1	0	0	0	1	0	0	0	0	0
0	0	1	0	1	0	0	0	1	0	0	1	0	0

LIN-3	MPK-1	LIN-39	LS	LIN-12m	LIN-12i	CKI-1	EFL-1	LIN-35	SCF	APC	CDK-4/CYD-1	CDK-2/CYE-1	CDK-1/CYB-3
0	0	1	1	1	0	0	0	0	0	0	1	0	0
0	0	1	1	1	0	0	1	0	0	0	1	0	0
0	0	1	1	1	0	0	1	0	0	0	1	1	0
0	0	1	1	1	0	0	1	0	1	0	1	1	0
0	0	1	1	1	0	0	1	0	1	0	0	0	0
0	0	1	1	1	0	0	1	1	0	0	0	0	1
0	0	1	1	1	0	1	0	1	0	1	0	0	1
0	0	1	1	1	0	1	0	1	0	1	0	0	0
0	0	1	1	1	0	1	0	1	0	0	0	0	0
0	0	1	1	1	0	0	0	1	0	0	0	0	0

0	0	1	1	1	0	0	0	1	0	0	1	0	0

LIN-12m to LIN-12i

LIN-3	MPK-1	LIN-39	LS	LIN-12m	LIN-12i	CKI-1	EFL-1	LIN-35	SCF	APC	CDK-4/CYD-1	CDK-2/CYE-1	CDK-1/CYB-3
2	2	2	0	0	0	0	0	1	0	0	1	0	0
2	2	2	0	1	0	0	0	0	0	0	1	0	0
2	2	2	0	1	0	0	1	0	0	0	1	0	0
2	2	2	0	1	0	0	1	0	0	0	1	1	0
2	2	2	0	1	0	0	1	0	1	0	1	1	0
2	2	2	0	1	0	0	1	0	1	0	0	0	0
2	2	2	0	0	0	0	1	1	0	0	0	0	1
2	2	2	0	0	0	0	0	1	0	1	0	0	1
2	2	2	0	0	0	0	0	1	0	1	0	0	0

LIN-3	MPK-1	LIN-39	LS	LIN-12m	LIN-12i	CKI-1	EFL-1	LIN-35	SCF	APC	CDK-4/CYD-1	CDK-2/CYE-1	CDK-1/CYB-3
2	2	2	1	0	0	0	0	1	0	0	1	0	0
2	2	2	1	1	0	0	0	0	0	0	1	0	0
2	2	2	1	1	0	0	1	0	0	0	1	0	0
2	2	2	1	1	0	0	1	0	0	0	1	1	0
2	2	2	1	1	0	0	1	0	1	0	1	1	0
2	2	2	1	1	0	0	1	0	1	0	0	0	0
2	2	2	1	0	0	0	1	1	0	0	0	0	1
2	2	2	1	0	0	0	0	1	0	1	0	0	1
2	2	2	1	0	0	0	0	1	0	1	0	0	0

LIN-3	MPK-1	LIN-39	LS	LIN-12m	LIN-12i	CKI-1	EFL-1	LIN-35	SCF	APC	CDK-4/CYD-1	CDK-2/CYE-1	CDK-1/CYB-3
3	2	2	0	0	0	0	0	1	0	0	1	0	0
3	2	2	0	1	0	0	0	0	0	0	1	0	0
3	2	2	0	1	0	0	1	0	0	0	1	0	0
3	2	2	0	1	0	0	1	0	0	0	1	1	0
3	2	2	0	1	0	0	1	0	1	0	1	1	0
3	2	2	0	1	0	0	1	0	1	0	0	0	0
3	2	2	0	0	0	0	1	1	0	0	0	0	1
3	2	2	0	0	0	0	0	1	0	1	0	0	1
3	2	2	0	0	0	0	0	1	0	1	0	0	0

LIN-3	MPK-1	LIN-39	LS	LIN-12m	LIN-12i	CKI-1	EFL-1	LIN-35	SCF	APC	CDK-4/CYD-1	CDK-2/CYE-1	CDK-1/CYB-3
3	2	2	1	0	0	0	0	1	0	0	1	0	0
3	2	2	1	1	0	0	0	0	0	0	1	0	0
3	2	2	1	1	0	0	1	0	0	0	1	0	0
3	2	2	1	1	0	0	1	0	0	0	1	1	0
3	2	2	1	1	0	0	1	0	1	0	1	1	0
3	2	2	1	1	0	0	1	0	1	0	0	0	0
3	2	2	1	0	0	0	1	1	0	0	0	0	1
3	2	2	1	0	0	0	0	1	0	1	0	0	1
3	2	2	1	0	0	0	0	1	0	1	0	0	0

LIN-3	MPK-1	LIN-39	LS	LIN-12m	LIN-12i	CKI-1	EFL-1	LIN-35	SCF	APC	CDK-4/CYD-1	CDK-2/CYE-1	CDK-1/CYB-3
1	0	1	0	1	0	1	0	1	0	1	0	0	1
1	1	1	0	1	0	1	0	1	0	1	0	0	0
1	1	1	0	1	1	0	0	1	0	0	0	0	0
1	0	1	0	1	1	0	0	1	0	0	1	0	0
1	0	1	0	1	1	0	0	0	0	0	1	0	0
1	0	1	0	1	1	0	1	0	0	0	1	0	0
1	0	1	0	1	1	0	1	0	0	0	1	1	0
1	0	1	0	1	1	0	1	0	1	0	1	1	0
1	0	1	0	1	1	0	1	0	1	0	0	0	0
1	0	1	0	1	1	0	1	1	0	0	0	0	1

LIN-3	MPK-1	LIN-39	LS	LIN-12m	LIN-12i	CKI-1	EFL-1	LIN-35	SCF	APC	CDK-4/CYD-1	CDK-2/CYE-1	CDK-1/CYB-3

1	0	1	1	1	0	1	0	1	0	1	0	0	1
1	1	1	1	1	0	1	0	1	0	1	0	0	0
1	1	1	1	1	1	0	0	1	0	0	0	0	0
1	0	1	1	1	1	0	0	1	0	0	1	0	0
1	0	1	1	1	1	0	0	0	0	0	1	0	0
1	0	1	1	1	1	0	1	0	0	0	1	0	0
1	0	1	1	1	1	0	1	0	0	0	1	1	0
1	0	1	1	1	1	0	1	0	1	0	1	1	0
1	0	1	1	1	1	0	1	0	1	0	0	0	0
1	0	1	1	1	1	0	1	1	0	0	0	0	1

LIN-3	MPK-1	LIN-39	LS	LIN-12m	LIN-12i	CKI-1	EFL-1	LIN-35	SCF	APC	CDK-4/CYD-1	CDK-2/CYE-1	CDK-1/CYB-3
0	0	1	0	1	0	0	0	0	0	0	1	0	0
0	0	1	0	1	0	0	1	0	0	0	1	0	0
0	0	1	0	1	0	0	1	0	0	0	1	1	0
0	0	1	0	1	0	0	1	0	1	0	1	1	0
0	0	1	0	1	0	0	1	0	1	0	0	0	0
0	0	1	0	1	0	0	1	1	0	0	0	0	1
0	0	1	0	1	0	1	0	1	0	1	0	0	1
0	0	1	0	1	0	1	0	1	0	1	0	0	0
0	0	1	0	1	0	1	0	1	0	0	0	0	0
0	0	1	0	1	0	0	0	1	0	0	0	0	0
0	0	1	0	1	0	0	0	1	0	0	1	0	0

LIN-3	MPK-1	LIN-39	LS	LIN-12m	LIN-12i	CKI-1	EFL-1	LIN-35	SCF	APC	CDK-4/CYD-1	CDK-2/CYE-1	CDK-1/CYB-3
0	0	1	1	1	0	0	0	0	0	0	1	0	0
0	0	1	1	1	0	0	1	0	0	0	1	0	0
0	0	1	1	1	0	0	1	0	0	0	1	1	0
0	0	1	1	1	0	0	1	0	1	0	1	1	0
0	0	1	1	1	0	0	1	0	1	0	0	0	0
0	0	1	1	1	0	0	1	1	0	0	0	0	1
0	0	1	1	1	0	1	0	1	0	1	0	0	1
0	0	1	1	1	0	1	0	1	0	1	0	0	0
0	0	1	1	1	0	1	0	1	0	0	0	0	0
0	0	1	1	1	0	0	0	1	0	0	0	0	0
0	0	1	1	1	0	0	0	1	0	0	1	0	0

LIN-12i to LIN-12i

LIN-3	MPK-1	LIN-39	LS	LIN-12m	LIN-12i	CKI-1	EFL-1	LIN-35	SCF	APC	CDK-4/CYD-1	CDK-2/CYE-1	CDK-1/CYB-3
2	1	1	1	1	0	0	0	1	0	1	0	0	1
2	2	1	1	1	0	0	0	1	0	1	0	0	0
2	2	2	1	0	1	0	0	1	0	0	1	0	0
2	1	2	1	1	0	0	0	0	0	0	1	0	0
2	2	1	1	1	1	0	1	0	0	0	1	0	0
2	1	2	1	1	1	0	1	0	0	0	1	1	0
2	1	1	1	1	1	0	1	0	1	0	1	1	0
2	1	1	1	1	1	0	1	0	1	0	0	0	0
2	1	1	1	1	1	0	1	1	0	0	0	0	1

LIN-3	MPK-1	LIN-39	LS	LIN-12m	LIN-12i	CKI-1	EFL-1	LIN-35	SCF	APC	CDK-4/CYD-1	CDK-2/CYE-1	CDK-1/CYB-3
2	2	2	0	0	0	0	0	1	0	0	1	0	0
2	2	2	0	1	0	0	0	0	0	0	1	0	0
2	2	2	0	1	0	0	1	0	0	0	1	0	0
2	2	2	0	1	0	0	1	0	0	0	1	1	0
2	2	2	0	1	0	0	1	0	1	0	1	1	0
2	2	2	0	1	0	0	1	0	1	0	0	0	0
2	2	2	0	0	0	0	1	1	0	0	0	0	1
2	2	2	0	0	0	0	0	1	0	1	0	0	1
2	2	2	0	0	0	0	0	1	0	1	0	0	0

LIN-3	MPK-1	LIN-39	LS	LIN-12m	LIN-12i	CKI-1	EFL-1	LIN-35	SCF	APC	CDK-4/CYD-1	CDK-2/CYE-1	CDK-1/CYB-3
3	2	2	0	0	0	0	0	1	0	0	1	0	0
3	2	2	0	1	0	0	0	0	0	0	1	0	0
3	2	2	0	1	0	0	1	0	0	0	1	0	0
3	2	2	0	1	0	0	1	0	0	0	1	1	0
3	2	2	0	1	0	0	1	0	1	0	1	1	0
3	2	2	0	1	0	0	1	0	1	0	0	0	0
3	2	2	0	0	0	0	1	1	0	0	0	0	1
3	2	2	0	0	0	0	0	1	0	1	0	0	1
3	2	2	0	0	0	0	0	1	0	1	0	0	0

LIN-3	MPK-1	LIN-39	LS	LIN-12m	LIN-12i	CKI-1	EFL-1	LIN-35	SCF	APC	CDK-4/CYD-1	CDK-2/CYE-1	CDK-1/CYB-3
3	2	2	1	0	0	0	0	1	0	0	1	0	0
3	2	2	1	1	0	0	0	0	0	0	1	0	0
3	2	2	1	1	1	0	1	0	0	0	1	0	0
3	2	2	1	1	1	0	1	0	0	0	1	1	0
3	2	2	1	1	1	0	1	0	1	0	1	1	0
3	2	2	1	1	1	0	1	0	1	0	0	0	0
3	2	2	1	0	1	0	1	1	0	0	0	0	1
3	2	2	1	0	0	0	0	1	0	1	0	0	1
3	2	2	1	0	0	0	0	1	0	1	0	0	0

LIN-3	MPK-1	LIN-39	LS	LIN-12m	LIN-12i	CKI-1	EFL-1	LIN-35	SCF	APC	CDK-4/CYD-1	CDK-2/CYE-1	CDK-1/CYB-3
1	0	1	0	1	0	1	0	1	0	1	0	0	1
1	1	1	0	1	0	1	0	1	0	1	0	0	0
1	1	1	0	1	1	0	0	1	0	0	0	0	0
1	0	1	0	1	1	0	0	1	0	0	1	0	0
1	0	1	0	1	1	0	0	0	0	0	1	0	0
1	0	1	0	1	1	0	1	0	0	0	1	0	0
1	0	1	0	1	1	0	1	0	0	0	1	1	0
1	0	1	0	1	1	0	1	0	1	0	1	1	0
1	0	1	0	1	1	0	1	0	1	0	0	0	0
1	0	1	0	1	1	0	1	1	0	0	0	0	1

LIN-3	MPK-1	LIN-39	LS	LIN-12m	LIN-12i	CKI-1	EFL-1	LIN-35	SCF	APC	CDK-4/CYD-1	CDK-2/CYE-1	CDK-1/CYB-3
1	0	1	1	1	0	1	0	1	0	1	0	0	1
1	1	1	1	1	0	1	0	1	0	1	0	0	0
1	1	1	1	1	1	0	0	1	0	0	0	0	0
1	0	1	1	1	1	0	0	1	0	0	1	0	0
1	0	1	1	1	1	0	0	0	0	0	1	0	0
1	0	1	1	1	1	0	1	0	0	0	1	0	0
1	0	1	1	1	1	0	1	0	0	0	1	1	0
1	0	1	1	1	1	0	1	0	1	0	1	1	0
1	0	1	1	1	1	0	1	0	1	0	0	0	0
1	0	1	1	1	1	0	1	1	0	0	0	0	1

LIN-3	MPK-1	LIN-39	LS	LIN-12m	LIN-12i	CKI-1	EFL-1	LIN-35	SCF	APC	CDK-4/CYD-1	CDK-2/CYE-1	CDK-1/CYB-3
0	0	1	0	1	0	0	0	0	0	0	1	0	0
0	0	1	0	1	0	0	1	0	0	0	1	0	0
0	0	1	0	1	0	0	1	0	0	0	1	1	0
0	0	1	0	1	0	0	1	0	1	0	1	1	0
0	0	1	0	1	0	0	1	0	1	0	0	0	0
0	0	1	0	1	0	0	1	1	0	0	0	0	1
0	0	1	0	1	0	1	0	1	0	1	0	0	1
0	0	1	0	1	0	1	0	1	0	1	0	0	0
0	0	1	0	1	0	1	0	1	0	0	0	0	0
0	0	1	0	1	0	0	0	1	0	0	0	0	0
0	0	1	0	1	0	0	0	1	0	0	1	0	0

LIN-3	MPK-1	LIN-39	LS	LIN-12m	LIN-12i	CKI-1	EFL-1	LIN-35	SCF	APC	CDK-4/CYD-1	CDK-2/CYE-1	CDK-1/CYB-3
0	0	1	1	1	0	1	0	1	0	1	0	0	0

0	0	1	1	1	1	1	0	1	0	0	0	0	0
0	0	1	1	1	1	0	0	1	0	0	0	0	0
0	0	1	1	1	1	0	0	1	0	0	1	0	0
0	0	1	1	1	1	0	0	0	0	0	1	0	0
0	0	1	1	1	1	0	1	0	0	0	1	0	0
0	0	1	1	1	1	0	1	0	0	0	1	1	0
0	0	1	1	1	1	0	1	0	1	0	1	1	0
0	0	1	1	1	1	0	1	0	1	0	0	0	0
0	0	1	1	1	1	0	1	1	0	0	0	0	1
0	0	1	1	1	0	1	0	1	0	1	0	0	1

CDK-2/CYE-1 to LIN-12i

LIN-3	MPK-1	LIN-39	LS	LIN-12m	LIN-12i	CKI-1	EFL-1	LIN-35	SCF	APC	CDK-4/CYD-1	CDK-2/CYE-1	CDK-1/CYB-3
2	1	1	1	1	0	0	0	1	0	1	0	0	1
2	2	1	1	1	0	0	0	1	0	1	0	0	0
2	2	2	1	0	1	0	0	1	0	0	1	0	0
2	1	2	1	1	1	0	0	0	0	0	1	0	0
2	1	1	1	1	1	0	1	0	0	0	1	0	0
2	1	1	1	1	1	0	1	0	0	0	1	1	0
2	1	1	1	1	1	0	1	0	1	0	1	1	0
2	1	1	1	1	1	0	1	0	1	0	0	0	0
2	1	1	1	1	1	0	1	1	0	0	0	0	1

LIN-3	MPK-1	LIN-39	LS	LIN-12m	LIN-12i	CKI-1	EFL-1	LIN-35	SCF	APC	CDK-4/CYD-1	CDK-2/CYE-1	CDK-1/CYB-3
2	2	2	0	0	0	0	0	1	0	0	1	0	0
2	2	2	0	1	0	0	0	0	0	0	1	0	0
2	2	2	0	1	0	0	1	0	0	0	1	0	0
2	2	2	0	1	0	0	1	0	0	0	1	1	0
2	2	2	0	1	0	0	1	0	1	0	1	1	0
2	2	2	0	1	0	0	1	0	1	0	0	0	0
2	2	2	0	0	0	0	1	1	0	0	0	0	1
2	2	2	0	0	0	0	0	1	0	1	0	0	1
2	2	2	0	0	0	0	0	1	0	1	0	0	0

LIN-3	MPK-1	LIN-39	LS	LIN-12m	LIN-12i	CKI-1	EFL-1	LIN-35	SCF	APC	CDK-4/CYD-1	CDK-2/CYE-1	CDK-1/CYB-3
3	2	2	0	0	0	0	0	1	0	0	1	0	0
3	2	2	0	1	0	0	0	0	0	0	1	0	0
3	2	2	0	1	0	0	1	0	0	0	1	0	0
3	2	2	0	1	0	0	1	0	0	0	1	1	0
3	2	2	0	1	0	0	1	0	1	0	1	1	0
3	2	2	0	1	0	0	1	0	1	0	0	0	0
3	2	2	0	0	0	0	1	1	0	0	0	0	1
3	2	2	0	0	0	0	0	1	0	1	0	0	1
3	2	2	0	0	0	0	0	1	0	1	0	0	0

LIN-3	MPK-1	LIN-39	LS	LIN-12m	LIN-12i	CKI-1	EFL-1	LIN-35	SCF	APC	CDK-4/CYD-1	CDK-2/CYE-1	CDK-1/CYB-3
3	2	2	1	0	0	0	0	1	0	0	1	0	0
3	2	2	1	1	0	0	0	0	0	0	1	0	0
3	2	2	1	1	1	0	1	0	0	0	1	0	0
3	2	2	1	1	1	0	1	0	0	0	1	1	0
3	2	2	1	1	1	0	1	0	1	0	1	1	0
3	2	2	1	1	1	0	1	0	1	0	0	0	0
3	2	2	1	0	1	0	1	1	0	0	0	0	1
3	2	2	1	0	0	0	0	1	0	1	0	0	1
3	2	2	1	0	0	0	0	1	0	1	0	0	0

LIN-3	MPK-1	LIN-39	LS	LIN-12m	LIN-12i	CKI-1	EFL-1	LIN-35	SCF	APC	CDK-4/CYD-1	CDK-2/CYE-1	CDK-1/CYB-3
1	0	1	0	1	0	1	0	1	0	1	0	0	1
1	1	1	0	1	0	1	0	1	0	1	0	0	0
1	1	1	0	1	1	0	0	1	0	0	0	0	0

1	0	1	0	1	1	0	0	1	0	0	1	0	0
1	0	1	0	1	1	0	0	0	0	0	1	0	0
1	0	1	0	1	1	0	1	0	0	0	1	0	0
1	0	1	0	1	1	0	1	0	0	0	1	1	0
1	0	1	0	1	1	0	1	0	1	0	1	1	0
1	0	1	0	1	1	0	1	0	1	0	0	0	0
1	0	1	0	1	1	0	1	1	0	0	0	0	1

LIN-3	MPK-1	LIN-39	LS	LIN-12m	LIN-12i	CKI-1	EFL-1	LIN-35	SCF	APC	CDK-4/CYD-1	CDK-2/CYE-1	CDK-1/CYB-3
1	0	1	1	1	0	1	0	1	0	1	0	0	1
1	1	1	1	1	0	1	0	1	0	1	0	0	0
1	1	1	1	1	1	0	0	1	0	0	0	0	0
1	0	1	1	1	1	0	0	1	0	0	1	0	0
1	0	1	1	1	1	0	0	0	0	0	1	0	0
1	0	1	1	1	1	0	1	0	0	0	1	0	0
1	0	1	1	1	1	0	1	0	0	0	1	1	0
1	0	1	1	1	1	0	1	0	1	0	1	1	0
1	0	1	1	1	1	0	1	0	1	0	0	0	0
1	0	1	1	1	1	0	1	1	0	0	0	0	1

LIN-3	MPK-1	LIN-39	LS	LIN-12m	LIN-12i	CKI-1	EFL-1	LIN-35	SCF	APC	CDK-4/CYD-1	CDK-2/CYE-1	CDK-1/CYB-3
0	0	1	0	1	0	0	0	0	0	0	1	0	0
0	0	1	0	1	0	0	1	0	0	0	1	0	0
0	0	1	0	1	0	0	1	0	0	0	1	1	0
0	0	1	0	1	0	0	1	0	1	0	1	1	0
0	0	1	0	1	0	0	1	0	1	0	0	0	0
0	0	1	0	1	0	0	1	1	0	0	0	0	1
0	0	1	0	1	0	1	0	1	0	1	0	0	1
0	0	1	0	1	0	1	0	1	0	1	0	0	0
0	0	1	0	1	0	1	0	1	0	0	0	0	0
0	0	1	0	1	0	0	0	1	0	0	0	0	0
0	0	1	0	1	0	0	0	1	0	0	1	0	0

LIN-3	MPK-1	LIN-39	LS	LIN-12m	LIN-12i	CKI-1	EFL-1	LIN-35	SCF	APC	CDK-4/CYD-1	CDK-2/CYE-1	CDK-1/CYB-3
0	0	1	1	1	0	1	0	1	0	1	0	0	0
0	0	1	1	1	1	1	0	1	0	0	0	0	0
0	0	1	1	1	1	0	0	1	0	0	0	0	0
0	0	1	1	1	1	0	0	1	0	0	1	0	0
0	0	1	1	1	1	0	0	0	0	0	1	0	0
0	0	1	1	1	1	0	1	0	0	0	1	0	0
0	0	1	1	1	1	0	1	0	0	0	1	1	0
0	0	1	1	1	1	0	1	0	1	0	1	1	0
0	0	1	1	1	1	0	1	0	1	0	0	0	0
0	0	1	1	1	1	0	1	1	0	0	0	0	1
0	0	1	1	1	0	1	0	1	0	1	0	0	1

CDK-1/CYB-3 to LIN-12i

LIN-3	MPK-1	LIN-39	LS	LIN-12m	LIN-12i	CKI-1	EFL-1	LIN-35	SCF	APC	CDK-4/CYD-1	CDK-2/CYE-1	CDK-1/CYB-3
2	1	1	0	1	1	0	0	0	0	0	1	0	0
2	1	1	0	1	1	0	1	0	0	0	1	0	0
2	1	1	0	1	1	0	1	0	0	0	1	1	0
2	1	1	0	1	1	0	1	0	1	0	1	1	0
2	1	1	0	1	1	0	1	0	1	0	0	0	0
2	1	1	0	1	1	0	1	1	0	0	0	0	1
2	1	1	0	1	1	0	0	1	0	1	0	0	1
2	1	1	0	1	1	0	0	1	0	1	0	0	0
2	1	1	0	1	1	0	0	1	0	0	1	0	0

LIN-3	MPK-1	LIN-39	LS	LIN-12m	LIN-12i	CKI-1	EFL-1	LIN-35	SCF	APC	CDK-4/CYD-1	CDK-2/CYE-1	CDK-1/CYB-3
2	1	1	1	1	1	0	0	0	0	0	1	0	0

2	1	1	1	1	1	0	1	0	0	0	1	0	0
2	1	1	1	1	1	0	1	0	0	0	1	1	0
2	1	1	1	1	1	0	1	0	1	0	1	1	0
2	1	1	1	1	1	0	1	0	1	0	0	0	0
2	1	1	1	1	1	0	1	1	0	0	0	0	1
2	1	1	1	1	1	0	0	1	0	1	0	0	1
2	1	1	1	1	1	0	0	1	0	1	0	0	0
2	1	1	1	1	1	0	0	1	0	0	1	0	0

LIN-3	MPK-1	LIN-39	LS	LIN-12m	LIN-12i	CKI-1	EFL-1	LIN-35	SCF	APC	CDK-4/CYD-1	CDK-2/CYE-1	CDK-1/CYB-3
2	2	2	0	0	0	0	0	1	0	0	1	0	0
2	2	2	0	1	0	0	0	0	0	0	1	0	0
2	2	2	0	1	0	0	1	0	0	0	1	0	0
2	2	2	0	1	0	0	1	0	0	0	1	1	0
2	2	2	0	1	0	0	1	0	1	0	1	1	0
2	2	2	0	1	0	0	1	0	1	0	0	0	0
2	2	2	0	0	0	0	1	1	0	0	0	0	1
2	2	2	0	0	0	0	0	1	0	1	0	0	1
2	2	2	0	0	0	0	0	1	0	1	0	0	0

LIN-3	MPK-1	LIN-39	LS	LIN-12m	LIN-12i	CKI-1	EFL-1	LIN-35	SCF	APC	CDK-4/CYD-1	CDK-2/CYE-1	CDK-1/CYB-3
3	2	2	0	0	0	0	0	1	0	0	1	0	0
3	2	2	0	1	0	0	0	0	0	0	1	0	0
3	2	2	0	1	0	0	1	0	0	0	1	0	0
3	2	2	0	1	0	0	1	0	0	0	1	1	0
3	2	2	0	1	0	0	1	0	1	0	1	1	0
3	2	2	0	1	0	0	1	0	1	0	0	0	0
3	2	2	0	0	0	0	1	1	0	0	0	0	1
3	2	2	0	0	0	0	0	1	0	1	0	0	1
3	2	2	0	0	0	0	0	1	0	1	0	0	0

LIN-3	MPK-1	LIN-39	LS	LIN-12m	LIN-12i	CKI-1	EFL-1	LIN-35	SCF	APC	CDK-4/CYD-1	CDK-2/CYE-1	CDK-1/CYB-3
3	2	2	0	0	1	0	0	1	0	0	1	0	0
3	2	2	0	1	1	0	0	0	0	0	1	0	0
3	2	2	0	1	1	0	1	0	0	0	1	0	0
3	2	2	0	1	1	0	1	0	0	0	1	1	0
3	2	2	0	1	1	0	1	0	1	0	1	1	0
3	2	2	0	1	1	0	1	0	1	0	0	0	0
3	2	2	0	0	1	0	1	1	0	0	0	0	1
3	2	2	0	0	1	0	0	1	0	1	0	0	1
3	2	2	0	0	1	0	0	1	0	1	0	0	0

LIN-3	MPK-1	LIN-39	LS	LIN-12m	LIN-12i	CKI-1	EFL-1	LIN-35	SCF	APC	CDK-4/CYD-1	CDK-2/CYE-1	CDK-1/CYB-3
3	2	2	1	0	1	0	0	1	0	0	1	0	0
3	2	2	1	1	1	0	0	0	0	0	1	0	0
3	2	2	1	1	1	0	1	0	0	0	1	0	0
3	2	2	1	1	1	0	1	0	0	0	1	1	0
3	2	2	1	1	1	0	1	0	1	0	1	1	0
3	2	2	1	1	1	0	1	0	1	0	0	0	0
3	2	2	1	0	1	0	1	1	0	0	0	0	1
3	2	2	1	0	1	0	0	1	0	1	0	0	1
3	2	2	1	0	1	0	0	1	0	1	0	0	0

LIN-3	MPK-1	LIN-39	LS	LIN-12m	LIN-12i	CKI-1	EFL-1	LIN-35	SCF	APC	CDK-4/CYD-1	CDK-2/CYE-1	CDK-1/CYB-3
0	0	1	0	1	0	0	0	0	0	0	1	0	0
0	0	1	0	1	0	0	1	0	0	0	1	0	0
0	0	1	0	1	0	0	1	0	0	0	1	1	0
0	0	1	0	1	0	0	1	0	1	0	1	1	0
0	0	1	0	1	0	0	1	0	1	0	0	0	0
0	0	1	0	1	0	0	1	1	0	0	0	0	1
0	0	1	0	1	0	1	0	1	0	1	0	0	1

0	0	1	0	1	0	1	0	1	0	1	0	0	0
0	0	1	0	1	0	1	0	1	0	0	0	0	0
0	0	1	0	1	0	0	0	1	0	0	0	0	0
0	0	1	0	1	0	0	0	1	0	0	1	0	0

LIN-3	MPK-1	LIN-39	LS	LIN-12m	LIN-12i	CKI-1	EFL-1	LIN-35	SCF	APC	CDK-4/CYD-1	CDK-2/CYE-1	CDK-1/CYB-3
0	0	1	0	1	1	0	0	0	0	0	1	0	0
0	0	1	0	1	1	0	1	0	0	0	1	0	0
0	0	1	0	1	1	0	1	0	0	0	1	1	0
0	0	1	0	1	1	0	1	0	1	0	1	1	0
0	0	1	0	1	1	0	1	0	1	0	0	0	0
0	0	1	0	1	1	0	1	1	0	0	0	0	1
0	0	1	0	1	1	1	0	1	0	1	0	0	1
0	0	1	0	1	1	1	0	1	0	1	0	0	0
0	0	1	0	1	1	1	0	1	0	0	0	0	0
0	0	1	0	1	1	0	0	1	0	0	0	0	0
0	0	1	0	1	1	0	0	1	0	0	1	0	0

LIN-3	MPK-1	LIN-39	LS	LIN-12m	LIN-12i	CKI-1	EFL-1	LIN-35	SCF	APC	CDK-4/CYD-1	CDK-2/CYE-1	CDK-1/CYB-3
0	0	1	1	1	1	0	0	0	0	0	1	0	0
0	0	1	1	1	1	0	1	0	0	0	1	0	0
0	0	1	1	1	1	0	1	0	0	0	1	1	0
0	0	1	1	1	1	0	1	0	1	0	1	1	0
0	0	1	1	1	1	0	1	0	1	0	0	0	0
0	0	1	1	1	1	0	1	1	0	0	0	0	1
0	0	1	1	1	1	1	0	1	0	1	0	0	1
0	0	1	1	1	1	1	0	1	0	1	0	0	0
0	0	1	1	1	1	1	0	1	0	0	0	0	0
0	0	1	1	1	1	0	0	1	0	0	0	0	0
0	0	1	1	1	1	0	0	1	0	0	1	0	0

LIN-3	MPK-1	LIN-39	LS	LIN-12m	LIN-12i	CKI-1	EFL-1	LIN-35	SCF	APC	CDK-4/CYD-1	CDK-2/CYE-1	CDK-1/CYB-3
1	0	1	0	1	1	0	0	0	0	0	1	0	0
1	0	1	0	1	1	0	1	0	0	0	1	0	0
1	0	1	0	1	1	0	1	0	0	0	1	1	0
1	0	1	0	1	1	0	1	0	1	0	1	1	0
1	0	1	0	1	1	0	1	0	1	0	0	0	0
1	0	1	0	1	1	0	1	1	0	0	0	0	1
1	0	1	0	1	1	1	0	1	0	1	0	0	1
1	0	1	0	1	1	1	0	1	0	1	0	0	0
1	0	1	0	1	1	1	0	1	0	0	0	0	0
1	0	1	0	1	1	0	0	1	0	0	0	0	0
1	0	1	0	1	1	0	0	1	0	0	1	0	0

LIN-3	MPK-1	LIN-39	LS	LIN-12m	LIN-12i	CKI-1	EFL-1	LIN-35	SCF	APC	CDK-4/CYD-1	CDK-2/CYE-1	CDK-1/CYB-3
1	0	1	1	1	1	0	0	0	0	0	1	0	0
1	0	1	1	1	1	0	1	0	0	0	1	0	0
1	0	1	1	1	1	0	1	0	0	0	1	1	0
1	0	1	1	1	1	0	1	0	1	0	1	1	0
1	0	1	1	1	1	0	1	0	1	0	0	0	0
1	0	1	1	1	1	0	1	1	0	0	0	0	1
1	0	1	1	1	1	1	0	1	0	1	0	0	1
1	0	1	1	1	1	1	0	1	0	1	0	0	0
1	0	1	1	1	1	1	0	1	0	0	0	0	0
1	0	1	1	1	1	0	0	1	0	0	0	0	0
1	0	1	1	1	1	0	0	1	0	0	1	0	0

MPK-1 to CKI-1

LIN-3	MPK-1	LIN-39	LS	LIN-12m	LIN-12i	CKI-1	EFL-1	LIN-35	SCF	APC	CDK-4/CYD-1	CDK-2/CYE-1	CDK-1/CYB-3
0	0	1	0	1	0	0	0	0	0	0	1	0	0

Interactions

0	0	1	0	1	0	0	1	0	0	0	1	0	0
0	0	1	0	1	0	0	1	0	0	0	1	1	0
0	0	1	0	1	0	0	1	0	1	0	1	1	0
0	0	1	0	1	0	0	1	0	1	0	0	0	0
0	0	1	0	1	0	0	1	1	0	0	0	0	1
0	0	1	0	1	0	1	0	1	0	1	0	0	1
0	0	1	0	1	0	1	0	1	0	1	0	0	0
0	0	1	0	1	0	1	0	1	0	0	0	0	0
0	0	1	0	1	0	0	0	1	0	0	0	0	0
0	0	1	0	1	0	0	0	1	0	0	1	0	0

LIN-3	MPK-1	LIN-39	LS	LIN-12m	LIN-12i	CKI-1	EFL-1	LIN-35	SCF	APC	CDK-4/CYD-1	CDK-2/CYE-1	CDK-1/CYB-3
0	0	1	1	1	0	1	0	1	0	1	0	0	0
0	0	1	1	1	1	1	0	1	0	0	0	0	0
0	0	1	1	1	1	0	0	1	0	0	0	0	0
0	0	1	1	1	1	0	0	1	0	0	1	0	0
0	0	1	1	1	1	0	0	0	0	0	1	0	0
0	0	1	1	1	1	0	1	0	0	0	1	0	0
0	0	1	1	1	1	0	1	0	0	0	1	1	0
0	0	1	1	1	1	0	1	0	1	0	1	1	0
0	0	1	1	1	1	0	1	0	1	0	0	0	0
0	0	1	1	1	1	0	1	1	0	0	0	0	1
0	0	1	1	1	0	1	0	1	0	1	0	0	1

LIN-3	MPK-1	LIN-39	LS	LIN-12m	LIN-12i	CKI-1	EFL-1	LIN-35	SCF	APC	CDK-4/CYD-1	CDK-2/CYE-1	CDK-1/CYB-3
1	0	1	0	1	0	1	0	1	0	1	0	0	1
1	1	1	0	1	0	1	0	1	0	1	0	0	0
1	1	1	0	1	1	1	0	1	0	0	0	0	0
1	0	1	0	1	1	0	0	1	0	0	0	0	0
1	0	1	0	1	1	0	0	1	0	0	1	0	0
1	0	1	0	1	1	0	0	0	0	0	1	0	0
1	0	1	0	1	1	0	1	0	0	0	1	0	0
1	0	1	0	1	1	0	1	0	0	0	1	1	0
1	0	1	0	1	1	0	1	0	1	0	1	1	0
1	0	1	0	1	1	0	1	0	1	0	0	0	0
1	0	1	0	1	1	0	1	1	0	0	0	0	1

LIN-3	MPK-1	LIN-39	LS	LIN-12m	LIN-12i	CKI-1	EFL-1	LIN-35	SCF	APC	CDK-4/CYD-1	CDK-2/CYE-1	CDK-1/CYB-3
1	0	1	1	1	0	1	0	1	0	1	0	0	1
1	1	1	1	1	0	1	0	1	0	1	0	0	0
1	1	1	1	1	1	1	0	1	0	0	0	0	0
1	0	1	1	1	1	0	0	1	0	0	0	0	0
1	0	1	1	1	1	0	0	1	0	0	1	0	0
1	0	1	1	1	1	0	0	0	0	0	1	0	0
1	0	1	1	1	1	0	1	0	0	0	1	0	0
1	0	1	1	1	1	0	1	0	0	0	1	1	0
1	0	1	1	1	1	0	1	0	1	0	1	1	0
1	0	1	1	1	1	0	1	0	1	0	0	0	0
1	0	1	1	1	1	0	1	1	0	0	0	0	1

LIN-3	MPK-1	LIN-39	LS	LIN-12m	LIN-12i	CKI-1	EFL-1	LIN-35	SCF	APC	CDK-4/CYD-1	CDK-2/CYE-1	CDK-1/CYB-3
2	1	1	1	1	0	1	0	1	0	1	0	0	1
2	2	1	1	1	0	1	0	1	0	1	0	0	0
2	2	2	1	0	1	1	0	1	0	0	0	0	0
2	1	2	1	0	1	0	0	1	0	0	0	0	0
2	1	1	1	1	1	0	0	1	0	0	1	0	0
2	1	1	1	1	1	0	0	0	0	0	1	0	0
2	1	1	1	1	1	0	1	0	0	0	1	0	0
2	1	1	1	1	1	0	1	0	0	0	1	1	0
2	1	1	1	1	1	0	1	0	1	0	1	1	0
2	1	1	1	1	1	0	1	0	1	0	0	0	0

2	1	1	1	1	1	0	1	1	0	0	0	0	1

LIN-3	MPK-1	LIN-39	LS	LIN-12m	LIN-12i	CKI-1	EFL-1	LIN-35	SCF	APC	CDK-4/CYD-1	CDK-2/CYE-1	CDK-1/CYB-3
2	2	2	0	0	0	0	0	1	0	0	0	0	0
2	2	2	0	0	0	0	0	1	0	0	1	0	0
2	2	2	0	1	0	0	0	0	0	0	1	0	0
2	2	2	0	1	0	0	1	0	0	0	1	0	0
2	2	2	0	1	0	0	1	0	0	0	1	1	0
2	2	2	0	1	0	0	1	0	1	0	1	1	0
2	2	2	0	1	0	0	1	0	1	0	0	0	0
2	2	2	0	0	0	0	1	1	0	0	0	0	1
2	2	2	0	0	0	1	0	1	0	1	0	0	1
2	2	2	0	0	0	1	0	1	0	1	0	0	0
2	2	2	0	0	0	1	0	1	0	0	0	0	0

LIN-3	MPK-1	LIN-39	LS	LIN-12m	LIN-12i	CKI-1	EFL-1	LIN-35	SCF	APC	CDK-4/CYD-1	CDK-2/CYE-1	CDK-1/CYB-3
3	2	2	0	0	0	0	0	1	0	0	0	0	0
3	2	2	0	0	0	0	0	1	0	0	1	0	0
3	2	2	0	1	0	0	0	0	0	0	1	0	0
3	2	2	0	1	0	0	1	0	0	0	1	0	0
3	2	2	0	1	0	0	1	0	0	0	1	1	0
3	2	2	0	1	0	0	1	0	1	0	1	1	0
3	2	2	0	1	0	0	1	0	1	0	0	0	0
3	2	2	0	0	0	0	1	1	0	0	0	0	1
3	2	2	0	0	0	1	0	1	0	1	0	0	1
3	2	2	0	0	0	1	0	1	0	1	0	0	0
3	2	2	0	0	0	1	0	1	0	0	0	0	0

LIN-3	MPK-1	LIN-39	LS	LIN-12m	LIN-12i	CKI-1	EFL-1	LIN-35	SCF	APC	CDK-4/CYD-1	CDK-2/CYE-1	CDK-1/CYB-3
3	2	2	1	0	0	0	0	1	0	0	0	0	0
3	2	2	1	0	0	0	0	1	0	0	1	0	0
3	2	2	1	1	0	0	0	0	0	0	1	0	0
3	2	2	1	1	1	0	1	0	0	0	1	0	0
3	2	2	1	1	1	0	1	0	0	0	1	1	0
3	2	2	1	1	1	0	1	0	1	0	1	1	0
3	2	2	1	1	1	0	1	0	1	0	0	0	0
3	2	2	1	0	1	0	1	1	0	0	0	0	1
3	2	2	1	0	0	1	0	1	0	1	0	0	1
3	2	2	1	0	0	1	0	1	0	1	0	0	0
3	2	2	1	0	0	1	0	1	0	0	0	0	0

APC to CKI-1

LIN-3	MPK-1	LIN-39	LS	LIN-12m	LIN-12i	CKI-1	EFL-1	LIN-35	SCF	APC	CDK-4/CYD-1	CDK-2/CYE-1	CDK-1/CYB-3
2	1	1	1	1	0	0	0	1	0	1	0	0	1
2	2	1	1	1	0	0	0	1	0	1	0	0	0
2	2	2	1	0	1	0	0	1	0	0	1	0	0
2	1	2	1	1	1	0	0	0	0	0	1	0	0
2	1	1	1	1	1	0	1	0	0	0	1	0	0
2	1	1	1	1	1	0	1	0	0	0	1	1	0
2	1	1	1	1	1	0	1	0	1	0	1	1	0
2	1	1	1	1	1	0	1	0	1	0	0	0	0
2	1	1	1	1	1	0	1	1	0	0	0	0	1

LIN-3	MPK-1	LIN-39	LS	LIN-12m	LIN-12i	CKI-1	EFL-1	LIN-35	SCF	APC	CDK-4/CYD-1	CDK-2/CYE-1	CDK-1/CYB-3
2	2	2	0	0	0	0	0	1	0	0	1	0	0
2	2	2	0	1	0	0	0	0	0	0	1	0	0
2	2	2	0	1	0	0	1	0	0	0	1	0	0
2	2	2	0	1	0	0	1	0	0	0	1	1	0
2	2	2	0	1	0	0	1	0	1	0	1	1	0
2	2	2	0	1	0	0	1	0	1	0	0	0	0

2	2	2	0	0	0	0	1	1	0	0	0	0	1
2	2	2	0	0	0	0	0	1	0	1	0	0	1
2	2	2	0	0	0	0	0	1	0	1	0	0	0

LIN-3	MPK-1	LIN-39	LS	LIN-12m	LIN-12i	CKI-1	EFL-1	LIN-35	SCF	APC	CDK-4/CYD-1	CDK-2/CYE-1	CDK-1/CYB-3
3	2	2	0	0	0	0	0	1	0	0	1	0	0
3	2	2	0	1	0	0	0	0	0	0	1	0	0
3	2	2	0	1	0	0	1	0	0	0	1	0	0
3	2	2	0	1	0	0	1	0	0	0	1	1	0
3	2	2	0	1	0	0	1	0	1	0	1	1	0
3	2	2	0	1	0	0	1	0	1	0	0	0	0
3	2	2	0	0	0	0	1	1	0	0	0	0	1
3	2	2	0	0	0	0	0	1	0	1	0	0	1
3	2	2	0	0	0	0	0	1	0	1	0	0	0

LIN-3	MPK-1	LIN-39	LS	LIN-12m	LIN-12i	CKI-1	EFL-1	LIN-35	SCF	APC	CDK-4/CYD-1	CDK-2/CYE-1	CDK-1/CYB-3
3	2	2	1	0	0	0	0	1	0	0	1	0	0
3	2	2	1	1	0	0	0	0	0	0	1	0	0
3	2	2	1	1	1	0	1	0	0	0	1	0	0
3	2	2	1	1	1	0	1	0	0	0	1	1	0
3	2	2	1	1	1	0	1	0	1	0	1	1	0
3	2	2	1	1	1	0	1	0	1	0	0	0	0
3	2	2	1	0	1	0	1	1	0	0	0	0	1
3	2	2	1	0	0	0	0	1	0	1	0	0	1
3	2	2	1	0	0	0	0	1	0	1	0	0	0

LIN-3	MPK-1	LIN-39	LS	LIN-12m	LIN-12i	CKI-1	EFL-1	LIN-35	SCF	APC	CDK-4/CYD-1	CDK-2/CYE-1	CDK-1/CYB-3
0	0	1	0	1	0	0	0	0	0	0	1	0	0
0	0	1	0	1	0	0	1	0	0	0	1	0	0
0	0	1	0	1	0	0	1	0	0	0	1	1	0
0	0	1	0	1	0	0	1	0	1	0	1	1	0
0	0	1	0	1	0	0	1	0	1	0	0	0	0
0	0	1	0	1	0	0	1	1	0	0	0	0	1
0	0	1	0	1	0	1	0	1	0	1	0	0	1
0	0	1	0	1	0	1	0	1	0	1	0	0	0
0	0	1	0	1	0	0	0	1	0	0	0	0	0
0	0	1	0	1	0	0	0	1	0	0	1	0	0

LIN-3	MPK-1	LIN-39	LS	LIN-12m	LIN-12i	CKI-1	EFL-1	LIN-35	SCF	APC	CDK-4/CYD-1	CDK-2/CYE-1	CDK-1/CYB-3
0	0	1	1	1	0	1	0	1	0	1	0	0	0
0	0	1	1	1	1	0	0	1	0	0	0	0	0
0	0	1	1	1	1	0	0	1	0	0	1	0	0
0	0	1	1	1	1	0	0	0	0	0	1	0	0
0	0	1	1	1	1	0	1	0	0	0	1	0	0
0	0	1	1	1	1	0	1	0	0	0	1	1	0
0	0	1	1	1	1	0	1	0	1	0	1	1	0
0	0	1	1	1	1	0	1	0	1	0	0	0	0
0	0	1	1	1	1	0	1	1	0	0	0	0	1
0	0	1	1	1	0	1	0	1	0	1	0	0	1

LIN-3	MPK-1	LIN-39	LS	LIN-12m	LIN-12i	CKI-1	EFL-1	LIN-35	SCF	APC	CDK-4/CYD-1	CDK-2/CYE-1	CDK-1/CYB-3
1	0	1	0	1	0	1	0	1	0	1	0	0	1
1	1	1	0	1	0	1	0	1	0	1	0	0	0
1	1	1	0	1	1	0	0	1	0	0	0	0	0
1	0	1	0	1	1	0	0	1	0	0	1	0	0
1	0	1	0	1	1	0	0	0	0	0	1	0	0
1	0	1	0	1	1	0	1	0	0	0	1	0	0
1	0	1	0	1	1	0	1	0	0	0	1	1	0
1	0	1	0	1	1	0	1	0	1	0	1	1	0
1	0	1	0	1	1	0	1	0	1	0	0	0	0
1	0	1	0	1	1	0	1	1	0	0	0	0	1

LIN-3	MPK-1	LIN-39	LS	LIN-12m	LIN-12i	CKI-1	EFL-1	LIN-35	SCF	APC	CDK-4/CYD-1	CDK-2/CYE-1	CDK-1/CYB-3
1	0	1	1	1	0	1	0	1	0	1	0	0	1
1	1	1	1	1	0	1	0	1	0	1	0	0	0
1	1	1	1	1	1	0	0	1	0	0	0	0	0
1	0	1	1	1	1	0	0	1	0	0	1	0	0
1	0	1	1	1	1	0	0	0	0	0	1	0	0
1	0	1	1	1	1	0	1	0	0	0	1	0	0
1	0	1	1	1	1	0	1	0	0	0	1	1	0
1	0	1	1	1	1	0	1	0	1	0	1	1	0
1	0	1	1	1	1	0	1	0	1	0	0	0	0
1	0	1	1	1	1	0	1	1	0	0	0	0	1

CDK-4/CYD-1 to CKI-1

LIN-3	MPK-1	LIN-39	LS	LIN-12m	LIN-12i	CKI-1	EFL-1	LIN-35	SCF	APC	CDK-4/CYD-1	CDK-2/CYE-1	CDK-1/CYB-3
2	1	1	1	1	0	0	0	1	0	1	0	0	1
2	2	1	1	1	0	0	0	1	0	1	0	0	0
2	2	2	1	0	1	0	0	1	0	0	1	0	0
2	1	2	1	1	1	0	0	0	0	0	1	0	0
2	1	1	1	1	1	0	1	0	0	0	1	0	0
2	1	1	1	1	1	0	1	0	0	0	1	1	0
2	1	1	1	1	1	0	1	0	1	0	1	1	0
2	1	1	1	1	1	0	1	0	1	0	0	0	0
2	1	1	1	1	1	0	1	1	0	0	0	0	1

LIN-3	MPK-1	LIN-39	LS	LIN-12m	LIN-12i	CKI-1	EFL-1	LIN-35	SCF	APC	CDK-4/CYD-1	CDK-2/CYE-1	CDK-1/CYB-3
2	2	2	0	0	0	0	0	1	0	0	1	0	0
2	2	2	0	1	0	0	0	0	0	0	1	0	0
2	2	2	0	1	0	0	1	0	0	0	1	0	0
2	2	2	0	1	0	0	1	0	0	0	1	1	0
2	2	2	0	1	0	0	1	0	1	0	1	1	0
2	2	2	0	1	0	0	1	0	1	0	0	0	0
2	2	2	0	0	0	0	1	1	0	0	0	0	1
2	2	2	0	0	0	0	0	1	0	1	0	0	1
2	2	2	0	0	0	0	0	1	0	1	0	0	0

LIN-3	MPK-1	LIN-39	LS	LIN-12m	LIN-12i	CKI-1	EFL-1	LIN-35	SCF	APC	CDK-4/CYD-1	CDK-2/CYE-1	CDK-1/CYB-3
3	2	2	0	0	0	0	0	1	0	0	1	0	0
3	2	2	0	1	0	0	0	0	0	0	1	0	0
3	2	2	0	1	0	0	1	0	0	0	1	0	0
3	2	2	0	1	0	0	1	0	0	0	1	1	0
3	2	2	0	1	0	0	1	0	1	0	1	1	0
3	2	2	0	1	0	0	1	0	1	0	0	0	0
3	2	2	0	0	0	0	1	1	0	0	0	0	1
3	2	2	0	0	0	0	0	1	0	1	0	0	1
3	2	2	0	0	0	0	0	1	0	1	0	0	0

LIN-3	MPK-1	LIN-39	LS	LIN-12m	LIN-12i	CKI-1	EFL-1	LIN-35	SCF	APC	CDK-4/CYD-1	CDK-2/CYE-1	CDK-1/CYB-3
3	2	2	1	0	0	0	0	1	0	0	1	0	0
3	2	2	1	1	0	0	0	0	0	0	1	0	0
3	2	2	1	1	1	0	1	0	0	0	1	0	0
3	2	2	1	1	1	0	1	0	0	0	1	1	0
3	2	2	1	1	1	0	1	0	1	0	1	1	0
3	2	2	1	1	1	0	1	0	1	0	0	0	0
3	2	2	1	0	1	0	1	1	0	0	0	0	1
3	2	2	1	0	0	0	0	1	0	1	0	0	1
3	2	2	1	0	0	0	0	1	0	1	0	0	0

LIN-3	MPK-1	LIN-39	LS	LIN-12m	LIN-12i	CKI-1	EFL-1	LIN-35	SCF	APC	CDK-4/CYD-1	CDK-2/CYE-1	CDK-1/CYB-3
1	0	1	0	1	0	1	0	1	0	1	0	0	1

1	1	1	0	1	0	1	0	1	0	1	0	0	0
1	1	1	0	1	1	0	0	1	0	0	0	0	0
1	0	1	0	1	1	0	0	1	0	0	1	0	0
1	0	1	0	1	1	0	0	0	0	0	1	0	0
1	0	1	0	1	1	0	1	0	0	0	1	0	0
1	0	1	0	1	1	0	1	0	0	0	1	1	0
1	0	1	0	1	1	0	1	0	1	0	1	1	0
1	0	1	0	1	1	0	1	0	1	0	0	0	0
1	0	1	0	1	1	0	1	1	0	0	0	0	1

LIN-3	MPK-1	LIN-39	LS	LIN-12m	LIN-12i	CKI-1	EFL-1	LIN-35	SCF	APC	CDK-4/CYD-1	CDK-2/CYE-1	CDK-1/CYB-3
1	0	1	1	1	0	1	0	1	0	1	0	0	1
1	1	1	1	1	0	1	0	1	0	1	0	0	0
1	1	1	1	1	1	0	0	1	0	0	0	0	0
1	0	1	1	1	1	0	0	1	0	0	1	0	0
1	0	1	1	1	1	0	0	0	0	0	1	0	0
1	0	1	1	1	1	0	1	0	0	0	1	0	0
1	0	1	1	1	1	0	1	0	0	0	1	1	0
1	0	1	1	1	1	0	1	0	1	0	1	1	0
1	0	1	1	1	1	0	1	0	1	0	0	0	0
1	0	1	1	1	1	0	1	1	0	0	0	0	1

LIN-3	MPK-1	LIN-39	LS	LIN-12m	LIN-12i	CKI-1	EFL-1	LIN-35	SCF	APC	CDK-4/CYD-1	CDK-2/CYE-1	CDK-1/CYB-3
0	0	1	0	1	0	0	0	0	0	0	1	0	0
0	0	1	0	1	0	0	1	0	0	0	1	0	0
0	0	1	0	1	0	0	1	0	0	0	1	1	0
0	0	1	0	1	0	0	1	0	1	0	1	1	0
0	0	1	0	1	0	0	1	0	1	0	0	0	0
0	0	1	0	1	0	0	1	1	0	0	0	0	1
0	0	1	0	1	0	1	0	1	0	1	0	0	1
0	0	1	0	1	0	1	0	1	0	1	0	0	0
0	0	1	0	1	0	1	0	1	0	0	0	0	0
0	0	1	0	1	0	0	0	1	0	0	0	0	0
0	0	1	0	1	0	0	0	1	0	0	1	0	0

LIN-3	MPK-1	LIN-39	LS	LIN-12m	LIN-12i	CKI-1	EFL-1	LIN-35	SCF	APC	CDK-4/CYD-1	CDK-2/CYE-1	CDK-1/CYB-3
0	0	1	1	1	0	1	0	1	0	1	0	0	0
0	0	1	1	1	1	1	0	1	0	0	0	0	0
0	0	1	1	1	1	0	0	1	0	0	0	0	0
0	0	1	1	1	1	0	0	1	0	0	1	0	0
0	0	1	1	1	1	0	0	0	0	0	1	0	0
0	0	1	1	1	1	0	1	0	0	0	1	0	0
0	0	1	1	1	1	0	1	0	0	0	1	1	0
0	0	1	1	1	1	0	1	0	1	0	1	1	0
0	0	1	1	1	1	0	1	0	1	0	0	0	0
0	0	1	1	1	1	0	1	1	0	0	0	0	1
0	0	1	1	1	0	1	0	1	0	1	0	0	1

CDK-1/CYB-3 to CKI-1

LIN-3	MPK-1	LIN-39	LS	LIN-12m	LIN-12i	CKI-1	EFL-1	LIN-35	SCF	APC	CDK-4/CYD-1	CDK-2/CYE-1	CDK-1/CYB-3
2	1	1	1	1	0	0	0	1	0	1	0	0	1
2	2	1	1	1	0	0	0	1	0	1	0	0	0
2	2	2	1	0	1	0	0	1	0	0	1	0	0
2	1	2	1	1	1	0	0	0	0	0	1	0	0
2	1	1	1	1	1	0	1	0	0	0	1	0	0
2	1	1	1	1	1	0	1	0	0	0	1	1	0
2	1	1	1	1	1	0	1	0	1	0	1	1	0
2	1	1	1	1	1	0	1	0	1	0	0	0	0
2	1	1	1	1	1	0	1	1	0	0	0	0	1

LIN-3	MPK-1	LIN-39	LS	LIN-12m	LIN-12i	CKI-1	EFL-1	LIN-35	SCF	APC	CDK-4/CYD-1	CDK-2/CYE-1	CDK-1/CYB-3
2	2	2	0	0	0	0	0	1	0	0	1	0	0
2	2	2	0	1	0	0	0	0	0	0	1	0	0
2	2	2	0	1	0	0	1	0	0	0	1	0	0
2	2	2	0	1	0	0	1	0	0	0	1	1	0
2	2	2	0	1	0	0	1	0	1	0	1	1	0
2	2	2	0	1	0	0	1	0	1	0	0	0	0
2	2	2	0	0	0	0	1	1	0	0	0	0	1
2	2	2	0	0	0	0	0	1	0	1	0	0	1
2	2	2	0	0	0	0	0	1	0	1	0	0	0

LIN-3	MPK-1	LIN-39	LS	LIN-12m	LIN-12i	CKI-1	EFL-1	LIN-35	SCF	APC	CDK-4/CYD-1	CDK-2/CYE-1	CDK-1/CYB-3
3	2	2	0	0	0	0	0	1	0	0	1	0	0
3	2	2	0	1	0	0	0	0	0	0	1	0	0
3	2	2	0	1	0	0	1	0	0	0	1	0	0
3	2	2	0	1	0	0	1	0	0	0	1	1	0
3	2	2	0	1	0	0	1	0	1	0	1	1	0
3	2	2	0	1	0	0	1	0	1	0	0	0	0
3	2	2	0	0	0	0	1	1	0	0	0	0	1
3	2	2	0	0	0	0	0	1	0	1	0	0	1
3	2	2	0	0	0	0	0	1	0	1	0	0	0

LIN-3	MPK-1	LIN-39	LS	LIN-12m	LIN-12i	CKI-1	EFL-1	LIN-35	SCF	APC	CDK-4/CYD-1	CDK-2/CYE-1	CDK-1/CYB-3
3	2	2	1	0	0	0	0	1	0	0	1	0	0
3	2	2	1	1	0	0	0	0	0	0	1	0	0
3	2	2	1	1	1	0	1	0	0	0	1	0	0
3	2	2	1	1	1	0	1	0	0	0	1	1	0
3	2	2	1	1	1	0	1	0	1	0	1	1	0
3	2	2	1	1	1	0	1	0	1	0	0	0	0
3	2	2	1	0	1	0	1	1	0	0	0	0	1
3	2	2	1	0	0	0	0	1	0	1	0	0	1
3	2	2	1	0	0	0	0	1	0	1	0	0	0

LIN-3	MPK-1	LIN-39	LS	LIN-12m	LIN-12i	CKI-1	EFL-1	LIN-35	SCF	APC	CDK-4/CYD-1	CDK-2/CYE-1	CDK-1/CYB-3
1	0	1	0	1	0	0	0	1	0	1	0	0	1
1	1	1	0	1	0	1	0	1	0	1	0	0	0
1	1	1	0	1	1	0	0	1	0	0	0	0	0
1	0	1	0	1	1	0	0	1	0	0	1	0	0
1	0	1	0	1	1	0	0	0	0	0	1	0	0
1	0	1	0	1	1	0	1	0	0	0	1	0	0
1	0	1	0	1	1	0	1	0	0	0	1	1	0
1	0	1	0	1	1	0	1	0	1	0	1	1	0
1	0	1	0	1	1	0	1	0	1	0	0	0	0
1	0	1	0	1	1	0	1	1	0	0	0	0	1

LIN-3	MPK-1	LIN-39	LS	LIN-12m	LIN-12i	CKI-1	EFL-1	LIN-35	SCF	APC	CDK-4/CYD-1	CDK-2/CYE-1	CDK-1/CYB-3
1	0	1	1	1	0	0	0	1	0	1	0	0	1
1	1	1	1	1	0	1	0	1	0	1	0	0	0
1	1	1	1	1	1	0	0	1	0	0	0	0	0
1	0	1	1	1	1	0	0	1	0	0	1	0	0
1	0	1	1	1	1	0	0	0	0	0	1	0	0
1	0	1	1	1	1	0	1	0	0	0	1	0	0
1	0	1	1	1	1	0	1	0	0	0	1	1	0
1	0	1	1	1	1	0	1	0	1	0	1	1	0
1	0	1	1	1	1	0	1	0	1	0	0	0	0
1	0	1	1	1	1	0	1	1	0	0	0	0	1

LIN-3	MPK-1	LIN-39	LS	LIN-12m	LIN-12i	CKI-1	EFL-1	LIN-35	SCF	APC	CDK-4/CYD-1	CDK-2/CYE-1	CDK-1/CYB-3
0	0	1	0	1	0	0	0	0	0	0	1	0	0
0	0	1	0	1	0	0	1	0	0	0	1	0	0
0	0	1	0	1	0	0	1	0	0	0	1	1	0

0	0	1	0	1	0	0	1	0	1	0	1	1	0
0	0	1	0	1	0	0	1	0	1	0	0	0	0
0	0	1	0	1	0	0	1	1	0	0	0	0	1
0	0	1	0	1	0	0	0	1	0	1	0	0	1
0	0	1	0	1	0	1	0	1	0	1	0	0	0
0	0	1	0	1	0	1	0	1	0	0	0	0	0
0	0	1	0	1	0	0	0	1	0	0	0	0	0
0	0	1	0	1	0	0	0	1	0	0	1	0	0

LIN-3	MPK-1	LIN-39	LS	LIN-12m	LIN-12i	CKI-1	EFL-1	LIN-35	SCF	APC	CDK-4/CYD-1	CDK-2/CYE-1	CDK-1/CYB-3
0	0	1	1	1	0	0	0	1	0	1	0	0	1
0	0	1	1	1	0	1	0	1	0	1	0	0	0
0	0	1	1	1	1	1	0	1	0	0	0	0	0
0	0	1	1	1	1	0	0	1	0	0	0	0	0
0	0	1	1	1	1	0	0	1	0	0	1	0	0
0	0	1	1	1	1	0	0	0	0	0	1	0	0
0	0	1	1	1	1	0	1	0	0	0	1	0	0
0	0	1	1	1	1	0	1	0	0	0	1	1	0
0	0	1	1	1	1	0	1	0	1	0	1	1	0
0	0	1	1	1	1	0	1	0	1	0	0	0	0
0	0	1	1	1	1	0	1	1	0	0	0	0	1

LIN-35 to EFL-1

LIN-3	MPK-1	LIN-39	LS	LIN-12m	LIN-12i	CKI-1	EFL-1	LIN-35	SCF	APC	CDK-4/CYD-1	CDK-2/CYE-1	CDK-1/CYB-3
0	0	1	0	1	0	0	1	0	0	1	0	0	1
0	0	1	0	1	0	1	1	1	0	1	0	1	0
0	0	1	0	1	0	1	1	0	0	0	0	0	0
0	0	1	0	1	0	0	1	1	0	0	0	0	0
0	0	1	0	1	0	0	1	1	0	0	1	0	1

LIN-3	MPK-1	LIN-39	LS	LIN-12m	LIN-12i	CKI-1	EFL-1	LIN-35	SCF	APC	CDK-4/CYD-1	CDK-2/CYE-1	CDK-1/CYB-3
0	0	1	1	1	0	0	1	0	0	1	0	0	1
0	0	1	1	1	0	1	1	1	0	1	0	1	0
0	0	1	1	1	1	1	1	0	0	0	0	0	0
0	0	1	1	1	1	0	1	1	0	0	0	0	0
0	0	1	1	1	1	0	1	1	0	0	1	0	1

LIN-3	MPK-1	LIN-39	LS	LIN-12m	LIN-12i	CKI-1	EFL-1	LIN-35	SCF	APC	CDK-4/CYD-1	CDK-2/CYE-1	CDK-1/CYB-3
1	0	1	0	1	0	0	1	0	0	1	0	0	1
1	1	1	0	1	0	1	1	1	0	1	0	1	0
1	1	1	0	1	1	0	1	0	0	0	0	0	0
1	0	1	0	1	1	0	1	1	0	0	1	1	1
1	0	1	0	1	1	0	1	0	1	1	0	0	1
1	0	1	0	1	0	1	1	1	0	0	0	0	0
1	1	1	0	1	1	0	1	1	0	0	0	0	0
1	0	1	0	1	1	0	1	1	0	0	1	0	1

LIN-3	MPK-1	LIN-39	LS	LIN-12m	LIN-12i	CKI-1	EFL-1	LIN-35	SCF	APC	CDK-4/CYD-1	CDK-2/CYE-1	CDK-1/CYB-3
1	0	1	1	1	0	0	1	0	0	1	0	0	1
1	1	1	1	1	0	1	1	1	0	1	0	1	0
1	1	1	1	1	1	0	1	0	0	0	0	0	0
1	0	1	1	1	1	0	1	1	0	0	1	1	1
1	0	1	1	1	1	0	1	0	1	1	0	0	1
1	0	1	1	1	0	1	1	1	0	0	0	0	0
1	1	1	1	1	1	0	1	1	0	0	0	0	0
1	0	1	1	1	1	0	1	1	0	0	1	0	1

LIN-3	MPK-1	LIN-39	LS	LIN-12m	LIN-12i	CKI-1	EFL-1	LIN-35	SCF	APC	CDK-4/CYD-1	CDK-2/CYE-1	CDK-1/CYB-3
2	1	1	1	1	0	0	1	1	0	1	0	0	1
2	2	1	1	1	0	0	1	1	0	1	0	0	0

2	2	2	1	0	1	0	1	1	0	0	1	0	0
2	1	2	1	1	1	0	1	0	0	0	1	0	0
2	1	1	1	1	1	0	1	0	0	0	1	1	0
2	1	1	1	1	1	0	1	0	1	0	1	1	0
2	1	1	1	1	1	0	1	0	1	0	0	0	0
2	1	1	1	1	1	0	1	1	0	0	0	0	1

LIN-3	MPK-1	LIN-39	LS	LIN-12m	LIN-12i	CKI-1	EFL-1	LIN-35	SCF	APC	CDK-4/CYD-1	CDK-2/CYE-1	CDK-1/CYB-3
2	2	2	0	0	0	0	1	1	0	0	0	0	1
2	2	2	0	0	0	0	1	1	0	1	0	0	1
2	2	2	0	0	0	0	1	1	0	1	0	0	0
2	2	2	0	0	0	0	1	1	0	0	1	0	0
2	2	2	0	1	0	0	1	0	0	0	1	0	0
2	2	2	0	1	0	0	1	0	0	0	1	1	0
2	2	2	0	1	0	0	1	0	1	0	1	1	0
2	2	2	0	1	0	0	1	0	1	0	0	0	0

LIN-3	MPK-1	LIN-39	LS	LIN-12m	LIN-12i	CKI-1	EFL-1	LIN-35	SCF	APC	CDK-4/CYD-1	CDK-2/CYE-1	CDK-1/CYB-3
3	2	2	0	0	0	0	1	1	0	0	0	0	1
3	2	2	0	0	0	0	1	1	0	1	0	0	1
3	2	2	0	0	0	0	1	1	0	1	0	0	0
3	2	2	0	0	0	0	1	1	0	0	1	0	0
3	2	2	0	1	0	0	1	0	0	0	1	0	0
3	2	2	0	1	0	0	1	0	0	0	1	1	0
3	2	2	0	1	0	0	1	0	1	0	1	1	0
3	2	2	0	1	0	0	1	0	1	0	0	0	0

LIN-3	MPK-1	LIN-39	LS	LIN-12m	LIN-12i	CKI-1	EFL-1	LIN-35	SCF	APC	CDK-4/CYD-1	CDK-2/CYE-1	CDK-1/CYB-3
3	2	2	1	0	0	0	1	1	0	0	1	0	0
3	2	2	1	1	0	0	1	0	0	0	1	0	0
3	2	2	1	1	1	0	1	0	0	0	1	1	0
3	2	2	1	1	1	0	1	0	1	0	1	1	0
3	2	2	1	1	1	0	1	0	1	0	0	0	0
3	2	2	1	0	1	0	1	1	0	0	0	0	1
3	2	2	1	0	0	0	1	1	0	1	0	0	1
3	2	2	1	0	0	0	1	1	0	1	0	0	0

CDK-4/CYD-1 to LIN-35

LIN-3	MPK-1	LIN-39	LS	LIN-12m	LIN-12i	CKI-1	EFL-1	LIN-35	SCF	APC	CDK-4/CYD-1	CDK-2/CYE-1	CDK-1/CYB-3
0	0	1	0	1	0	0	0	1	0	0	1	0	0

LIN-3	MPK-1	LIN-39	LS	LIN-12m	LIN-12i	CKI-1	EFL-1	LIN-35	SCF	APC	CDK-4/CYD-1	CDK-2/CYE-1	CDK-1/CYB-3
0	0	1	0	1	1	0	0	1	0	0	1	0	0

LIN-3	MPK-1	LIN-39	LS	LIN-12m	LIN-12i	CKI-1	EFL-1	LIN-35	SCF	APC	CDK-4/CYD-1	CDK-2/CYE-1	CDK-1/CYB-3
0	0	1	1	1	1	0	0	1	0	0	1	0	0

LIN-3	MPK-1	LIN-39	LS	LIN-12m	LIN-12i	CKI-1	EFL-1	LIN-35	SCF	APC	CDK-4/CYD-1	CDK-2/CYE-1	CDK-1/CYB-3
1	0	1	0	1	1	0	0	1	0	0	1	0	0

LIN-3	MPK-1	LIN-39	LS	LIN-12m	LIN-12i	CKI-1	EFL-1	LIN-35	SCF	APC	CDK-4/CYD-1	CDK-2/CYE-1	CDK-1/CYB-3
1	0	1	1	1	1	0	0	1	0	0	1	0	0

LIN-3	MPK-1	LIN-39	LS	LIN-12m	LIN-12i	CKI-1	EFL-1	LIN-35	SCF	APC	CDK-4/CYD-1	CDK-2/CYE-1	CDK-1/CYB-3
2	1	1	0	1	1	0	0	1	0	0	1	0	0

LIN-3	MPK-1	LIN-39	LS	LIN-12m	LIN-12i	CKI-1	EFL-1	LIN-35	SCF	APC	CDK-4/CYD-1	CDK-2/CYE-1	CDK-1/CYB-3
2	1	1	1	1	1	0	0	1	0	0	1	0	0

LIN-3	MPK-1	LIN-39	LS	LIN-12m	LIN-12i	CKI-1	EFL-1	LIN-35	SCF	APC	CDK-4/CYD-1	CDK-2/CYE-1	CDK-1/CYB-3

2	2	2	0	1	0	0	0	1	0	0	1	0	0

LIN-3	MPK-1	LIN-39	LS	LIN-12m	LIN-12i	CKI-1	EFL-1	LIN-35	SCF	APC	CDK-4/CYD-1	CDK-2/CYE-1	CDK-1/CYB-3
3	2	2	0	1	0	0	0	1	0	0	1	0	0

LIN-3	MPK-1	LIN-39	LS	LIN-12m	LIN-12i	CKI-1	EFL-1	LIN-35	SCF	APC	CDK-4/CYD-1	CDK-2/CYE-1	CDK-1/CYB-3
3	2	2	0	1	1	0	0	1	0	0	1	0	0

LIN-3	MPK-1	LIN-39	LS	LIN-12m	LIN-12i	CKI-1	EFL-1	LIN-35	SCF	APC	CDK-4/CYD-1	CDK-2/CYE-1	CDK-1/CYB-3
3	2	2	1	1	1	0	0	1	0	0	1	0	0

CDK-2/CYE-1 to LIN-35

LIN-3	MPK-1	LIN-39	LS	LIN-12m	LIN-12i	CKI-1	EFL-1	LIN-35	SCF	APC	CDK-4/CYD-1	CDK-2/CYE-1	CDK-1/CYB-3
2	1	1	1	1	0	0	0	1	0	1	0	0	1
2	2	1	1	1	0	0	0	1	0	1	0	0	0
2	2	2	1	0	1	0	0	1	0	0	1	0	0
2	1	2	1	1	1	0	0	0	0	0	1	0	0
2	1	1	1	1	1	0	1	0	0	0	1	0	0
2	1	1	1	1	1	0	1	0	0	0	1	1	0
2	1	1	1	1	1	0	1	0	1	0	1	1	0
2	1	1	1	1	1	0	1	0	1	0	0	0	0
2	1	1	1	1	1	0	1	1	0	0	0	0	1

LIN-3	MPK-1	LIN-39	LS	LIN-12m	LIN-12i	CKI-1	EFL-1	LIN-35	SCF	APC	CDK-4/CYD-1	CDK-2/CYE-1	CDK-1/CYB-3
2	2	2	0	0	0	0	0	1	0	0	1	0	0
2	2	2	0	1	0	0	0	0	0	0	1	0	0
2	2	2	0	1	0	0	1	0	0	0	1	0	0
2	2	2	0	1	0	0	1	0	0	0	1	1	0
2	2	2	0	1	0	0	1	0	1	0	1	1	0
2	2	2	0	1	0	0	1	0	1	0	0	0	0
2	2	2	0	0	0	0	1	1	0	0	0	0	1
2	2	2	0	0	0	0	0	1	0	1	0	0	1
2	2	2	0	0	0	0	0	1	0	1	0	0	0

LIN-3	MPK-1	LIN-39	LS	LIN-12m	LIN-12i	CKI-1	EFL-1	LIN-35	SCF	APC	CDK-4/CYD-1	CDK-2/CYE-1	CDK-1/CYB-3
3	2	2	0	0	0	0	0	1	0	0	1	0	0
3	2	2	0	1	0	0	0	0	0	0	1	0	0
3	2	2	0	1	0	0	1	0	0	0	1	0	0
3	2	2	0	1	0	0	1	0	0	0	1	1	0
3	2	2	0	1	0	0	1	0	1	0	1	1	0
3	2	2	0	1	0	0	1	0	1	0	0	0	0
3	2	2	0	0	0	0	1	1	0	0	0	0	1
3	2	2	0	0	0	0	0	1	0	1	0	0	1
3	2	2	0	0	0	0	0	1	0	1	0	0	0

LIN-3	MPK-1	LIN-39	LS	LIN-12m	LIN-12i	CKI-1	EFL-1	LIN-35	SCF	APC	CDK-4/CYD-1	CDK-2/CYE-1	CDK-1/CYB-3
3	2	2	1	0	0	0	0	1	0	0	1	0	0
3	2	2	1	1	0	0	0	0	0	0	1	0	0
3	2	2	1	1	1	0	1	0	0	0	1	0	0
3	2	2	1	1	1	0	1	0	0	0	1	1	0
3	2	2	1	1	1	0	1	0	1	0	1	1	0
3	2	2	1	1	1	0	1	0	1	0	0	0	0
3	2	2	1	0	1	0	1	1	0	0	0	0	1
3	2	2	1	0	0	0	0	1	0	1	0	0	1
3	2	2	1	0	0	0	0	1	0	1	0	0	0

LIN-3	MPK-1	LIN-39	LS	LIN-12m	LIN-12i	CKI-1	EFL-1	LIN-35	SCF	APC	CDK-4/CYD-1	CDK-2/CYE-1	CDK-1/CYB-3
1	0	1	0	1	0	1	0	1	0	1	0	0	1
1	1	1	0	1	0	1	0	1	0	1	0	0	0
1	1	1	0	1	1	0	0	1	0	0	0	0	0

1	0	1	0	1	1	0	0	1	0	0	1	0	0
1	0	1	0	1	1	0	0	0	0	0	1	0	0
1	0	1	0	1	1	0	1	0	0	0	1	0	0
1	0	1	0	1	1	0	1	0	0	0	1	1	0
1	0	1	0	1	1	0	1	0	1	0	1	1	0
1	0	1	0	1	1	0	1	0	1	0	0	0	0
1	0	1	0	1	1	0	1	1	0	0	0	0	1

LIN-3	MPK-1	LIN-39	LS	LIN-12m	LIN-12i	CKI-1	EFL-1	LIN-35	SCF	APC	CDK-4/CYD-1	CDK-2/CYE-1	CDK-1/CYB-3
1	0	1	1	1	0	1	0	1	0	1	0	0	1
1	1	1	1	1	0	1	0	1	0	1	0	0	0
1	1	1	1	1	1	0	0	1	0	0	0	0	0
1	0	1	1	1	1	0	0	1	0	0	1	0	0
1	0	1	1	1	1	0	0	0	0	0	1	0	0
1	0	1	1	1	1	0	1	0	0	0	1	0	0
1	0	1	1	1	1	0	1	0	0	0	1	1	0
1	0	1	1	1	1	0	1	0	1	0	1	1	0
1	0	1	1	1	1	0	1	0	1	0	0	0	0
1	0	1	1	1	1	0	1	1	0	0	0	0	1

LIN-3	MPK-1	LIN-39	LS	LIN-12m	LIN-12i	CKI-1	EFL-1	LIN-35	SCF	APC	CDK-4/CYD-1	CDK-2/CYE-1	CDK-1/CYB-3
0	0	1	0	1	0	0	0	0	0	0	1	0	0
0	0	1	0	1	0	0	1	0	0	0	1	0	0
0	0	1	0	1	0	0	1	0	0	0	1	1	0
0	0	1	0	1	0	0	1	0	1	0	1	1	0
0	0	1	0	1	0	0	1	0	1	0	0	0	0
0	0	1	0	1	0	0	1	1	0	0	0	0	1
0	0	1	0	1	0	1	0	1	0	1	0	0	1
0	0	1	0	1	0	1	0	1	0	1	0	0	0
0	0	1	0	1	0	1	0	1	0	0	0	0	0
0	0	1	0	1	0	0	0	1	0	0	0	0	0
0	0	1	0	1	0	0	0	1	0	0	1	0	0

LIN-3	MPK-1	LIN-39	LS	LIN-12m	LIN-12i	CKI-1	EFL-1	LIN-35	SCF	APC	CDK-4/CYD-1	CDK-2/CYE-1	CDK-1/CYB-3
0	0	1	1	1	0	1	0	1	0	1	0	0	0
0	0	1	1	1	1	1	0	1	0	0	0	0	0
0	0	1	1	1	1	0	0	1	0	0	0	0	0
0	0	1	1	1	1	0	0	1	0	0	1	0	0
0	0	1	1	1	1	0	0	0	0	0	1	0	0
0	0	1	1	1	1	0	1	0	0	0	1	0	0
0	0	1	1	1	1	0	1	0	0	0	1	1	0
0	0	1	1	1	1	0	1	0	1	0	1	1	0
0	0	1	1	1	1	0	1	0	1	0	0	0	0
0	0	1	1	1	1	0	1	1	0	0	0	0	1
0	0	1	1	1	0	1	0	1	0	1	0	0	1

LIN-39 to SCF

LIN-3	MPK-1	LIN-39	LS	LIN-12m	LIN-12i	CKI-1	EFL-1	LIN-35	SCF	APC	CDK-4/CYD-1	CDK-2/CYE-1	CDK-1/CYB-3
0	0	1	0	1	0	0	1	0	0	0	1	1	0

LIN-3	MPK-1	LIN-39	LS	LIN-12m	LIN-12i	CKI-1	EFL-1	LIN-35	SCF	APC	CDK-4/CYD-1	CDK-2/CYE-1	CDK-1/CYB-3
0	0	1	0	1	1	0	1	0	0	0	1	1	0

LIN-3	MPK-1	LIN-39	LS	LIN-12m	LIN-12i	CKI-1	EFL-1	LIN-35	SCF	APC	CDK-4/CYD-1	CDK-2/CYE-1	CDK-1/CYB-3
0	0	1	1	1	1	0	1	0	0	0	1	1	0

LIN-3	MPK-1	LIN-39	LS	LIN-12m	LIN-12i	CKI-1	EFL-1	LIN-35	SCF	APC	CDK-4/CYD-1	CDK-2/CYE-1	CDK-1/CYB-3
1	0	1	0	1	1	0	1	0	0	0	1	1	0

LIN-3	MPK-1	LIN-39	LS	LIN-12m	LIN-12i	CKI-1	EFL-1	LIN-35	SCF	APC	CDK-4/CYD-1	CDK-2/CYE-1	CDK-1/CYB-3

1	0	1	1	1	1	0	1	0	0	0	1	1	0

LIN-3	MPK-1	LIN-39	LS	LIN-12m	LIN-12i	CKI-1	EFL-1	LIN-35	SCF	APC	CDK-4/CYD-1	CDK-2/CYE-1	CDK-1/CYB-3
2	1	1	0	1	1	0	1	0	0	0	1	1	0

LIN-3	MPK-1	LIN-39	LS	LIN-12m	LIN-12i	CKI-1	EFL-1	LIN-35	SCF	APC	CDK-4/CYD-1	CDK-2/CYE-1	CDK-1/CYB-3
2	1	1	1	1	1	0	1	0	0	0	1	1	0

LIN-3	MPK-1	LIN-39	LS	LIN-12m	LIN-12i	CKI-1	EFL-1	LIN-35	SCF	APC	CDK-4/CYD-1	CDK-2/CYE-1	CDK-1/CYB-3
2	2	2	0	1	0	0	1	0	0	0	1	1	0

LIN-3	MPK-1	LIN-39	LS	LIN-12m	LIN-12i	CKI-1	EFL-1	LIN-35	SCF	APC	CDK-4/CYD-1	CDK-2/CYE-1	CDK-1/CYB-3
3	2	2	0	1	0	0	1	0	0	0	1	1	0

LIN-3	MPK-1	LIN-39	LS	LIN-12m	LIN-12i	CKI-1	EFL-1	LIN-35	SCF	APC	CDK-4/CYD-1	CDK-2/CYE-1	CDK-1/CYB-3
3	2	2	0	1	1	0	1	0	0	0	1	1	0

LIN-3	MPK-1	LIN-39	LS	LIN-12m	LIN-12i	CKI-1	EFL-1	LIN-35	SCF	APC	CDK-4/CYD-1	CDK-2/CYE-1	CDK-1/CYB-3
3	2	2	1	1	1	0	1	0	0	0	1	1	0

APC to SCF

LIN-3	MPK-1	LIN-39	LS	LIN-12m	LIN-12i	CKI-1	EFL-1	LIN-35	SCF	APC	CDK-4/CYD-1	CDK-2/CYE-1	CDK-1/CYB-3
2	1	1	1	1	0	0	0	1	0	1	0	0	1
2	2	1	1	1	0	0	0	1	0	1	0	0	0
2	2	2	1	0	1	0	0	1	0	0	1	0	0
2	1	2	1	1	1	0	0	0	0	0	1	0	0
2	1	1	1	1	1	0	1	0	0	0	1	0	0
2	1	1	1	1	1	0	1	0	0	0	1	1	0
2	1	1	1	1	1	0	1	0	1	0	1	1	0
2	1	1	1	1	1	0	1	0	1	0	0	0	0
2	1	1	1	1	1	0	1	1	0	0	0	0	1

LIN-3	MPK-1	LIN-39	LS	LIN-12m	LIN-12i	CKI-1	EFL-1	LIN-35	SCF	APC	CDK-4/CYD-1	CDK-2/CYE-1	CDK-1/CYB-3
2	2	2	0	0	0	0	0	1	0	0	1	0	0
2	2	2	0	1	0	0	0	0	0	0	1	0	0
2	2	2	0	1	0	0	1	0	0	0	1	0	0
2	2	2	0	1	0	0	1	0	0	0	1	1	0
2	2	2	0	1	0	0	1	0	1	0	1	1	0
2	2	2	0	1	0	0	1	0	1	0	0	0	0
2	2	2	0	0	0	0	1	1	0	0	0	0	1
2	2	2	0	0	0	0	0	1	0	1	0	0	1
2	2	2	0	0	0	0	0	1	0	1	0	0	0

LIN-3	MPK-1	LIN-39	LS	LIN-12m	LIN-12i	CKI-1	EFL-1	LIN-35	SCF	APC	CDK-4/CYD-1	CDK-2/CYE-1	CDK-1/CYB-3
3	2	2	0	0	0	0	0	1	0	0	1	0	0
3	2	2	0	1	0	0	0	0	0	0	1	0	0
3	2	2	0	1	0	0	1	0	0	0	1	0	0
3	2	2	0	1	0	0	1	0	0	0	1	1	0
3	2	2	0	1	0	0	1	0	1	0	1	1	0
3	2	2	0	1	0	0	1	0	1	0	0	0	0
3	2	2	0	0	0	0	1	1	0	0	0	0	1
3	2	2	0	0	0	0	0	1	0	1	0	0	1
3	2	2	0	0	0	0	0	1	0	1	0	0	0

LIN-3	MPK-1	LIN-39	LS	LIN-12m	LIN-12i	CKI-1	EFL-1	LIN-35	SCF	APC	CDK-4/CYD-1	CDK-2/CYE-1	CDK-1/CYB-3
3	2	2	1	0	0	0	0	1	0	0	1	0	0
3	2	2	1	1	0	0	0	0	0	0	1	0	0
3	2	2	1	1	1	0	1	0	0	0	1	0	0
3	2	2	1	1	1	0	1	0	0	0	1	1	0
3	2	2	1	1	1	0	1	0	1	0	1	1	0

3	2	2	1	1	1	0	1	0	1	0	0	0	0
3	2	2	1	0	1	0	1	1	0	0	0	0	1
3	2	2	1	0	0	0	0	1	0	1	0	0	1
3	2	2	1	0	0	0	0	1	0	1	0	0	0

LIN-3	MPK-1	LIN-39	LS	LIN-12m	LIN-12i	CKI-1	EFL-1	LIN-35	SCF	APC	CDK-4/CYD-1	CDK-2/CYE-1	CDK-1/CYB-3
1	0	1	0	1	0	1	0	1	0	1	0	0	1
1	1	1	0	1	0	1	0	1	0	1	0	0	0
1	1	1	0	1	1	0	0	1	0	0	0	0	0
1	0	1	0	1	1	0	0	1	0	0	1	0	0
1	0	1	0	1	1	0	0	0	0	0	1	0	0
1	0	1	0	1	1	0	1	0	0	0	1	0	0
1	0	1	0	1	1	0	1	0	0	0	1	1	0
1	0	1	0	1	1	0	1	0	1	0	1	1	0
1	0	1	0	1	1	0	1	0	1	0	0	0	0
1	0	1	0	1	1	0	1	1	0	0	0	0	1

LIN-3	MPK-1	LIN-39	LS	LIN-12m	LIN-12i	CKI-1	EFL-1	LIN-35	SCF	APC	CDK-4/CYD-1	CDK-2/CYE-1	CDK-1/CYB-3
1	0	1	1	1	0	1	0	1	0	1	0	0	1
1	1	1	1	1	0	1	0	1	0	1	0	0	0
1	1	1	1	1	1	0	0	1	0	0	0	0	0
1	0	1	1	1	1	0	0	1	0	0	1	0	0
1	0	1	1	1	1	0	0	0	0	0	1	0	0
1	0	1	1	1	1	0	1	0	0	0	1	0	0
1	0	1	1	1	1	0	1	0	0	0	1	1	0
1	0	1	1	1	1	0	1	0	1	0	1	1	0
1	0	1	1	1	1	0	1	0	1	0	0	0	0
1	0	1	1	1	1	0	1	1	0	0	0	0	1

LIN-3	MPK-1	LIN-39	LS	LIN-12m	LIN-12i	CKI-1	EFL-1	LIN-35	SCF	APC	CDK-4/CYD-1	CDK-2/CYE-1	CDK-1/CYB-3
0	0	1	0	1	0	0	0	0	0	0	1	0	0
0	0	1	0	1	0	0	1	0	0	0	1	0	0
0	0	1	0	1	0	0	1	0	0	0	1	1	0
0	0	1	0	1	0	0	1	0	1	0	1	1	0
0	0	1	0	1	0	0	1	0	1	0	0	0	0
0	0	1	0	1	0	0	1	1	0	0	0	0	1
0	0	1	0	1	0	1	0	1	0	1	0	0	1
0	0	1	0	1	0	1	0	1	0	1	0	0	0
0	0	1	0	1	0	1	0	1	0	0	0	0	0
0	0	1	0	1	0	0	0	1	0	0	0	0	0
0	0	1	0	1	0	0	0	1	0	0	1	0	0

LIN-3	MPK-1	LIN-39	LS	LIN-12m	LIN-12i	CKI-1	EFL-1	LIN-35	SCF	APC	CDK-4/CYD-1	CDK-2/CYE-1	CDK-1/CYB-3
0	0	1	1	1	0	1	0	1	0	1	0	0	0
0	0	1	1	1	1	1	0	1	0	0	0	0	0
0	0	1	1	1	1	0	0	1	0	0	0	0	0
0	0	1	1	1	1	0	0	1	0	0	1	0	0
0	0	1	1	1	1	0	0	0	0	0	1	0	0
0	0	1	1	1	1	0	1	0	0	0	1	0	0
0	0	1	1	1	1	0	1	0	0	0	1	1	0
0	0	1	1	1	1	0	1	0	1	0	1	1	0
0	0	1	1	1	1	0	1	0	1	0	0	0	0
0	0	1	1	1	1	0	1	1	0	0	0	0	1
0	0	1	1	1	0	1	0	1	0	1	0	0	1

CDK-2/CYE-1 to SCF

LIN-3	MPK-1	LIN-39	LS	LIN-12m	LIN-12i	CKI-1	EFL-1	LIN-35	SCF	APC	CDK-4/CYD-1	CDK-2/CYE-1	CDK-1/CYB-3
0	0	1	0	1	0	0	1	0	0	0	1	1	0

LIN-3	MPK-1	LIN-39	LS	LIN-12m	LIN-12i	CKI-1	EFL-1	LIN-35	SCF	APC	CDK-4/CYD-1	CDK-2/CYE-1	CDK-1/CYB-3

0	0	1	0	1	1	0	1	0	0	0	1	1	0

LIN-3	MPK-1	LIN-39	LS	LIN-12m	LIN-12i	CKI-1	EFL-1	LIN-35	SCF	APC	CDK-4/CYD-1	CDK-2/CYE-1	CDK-1/CYB-3
0	0	1	1	1	1	0	1	0	0	0	1	1	0

LIN-3	MPK-1	LIN-39	LS	LIN-12m	LIN-12i	CKI-1	EFL-1	LIN-35	SCF	APC	CDK-4/CYD-1	CDK-2/CYE-1	CDK-1/CYB-3
1	0	1	0	1	1	0	1	0	0	0	1	1	0

LIN-3	MPK-1	LIN-39	LS	LIN-12m	LIN-12i	CKI-1	EFL-1	LIN-35	SCF	APC	CDK-4/CYD-1	CDK-2/CYE-1	CDK-1/CYB-3
1	0	1	1	1	1	0	1	0	0	0	1	1	0

LIN-3	MPK-1	LIN-39	LS	LIN-12m	LIN-12i	CKI-1	EFL-1	LIN-35	SCF	APC	CDK-4/CYD-1	CDK-2/CYE-1	CDK-1/CYB-3
2	1	1	0	1	1	0	1	0	0	0	1	1	0

LIN-3	MPK-1	LIN-39	LS	LIN-12m	LIN-12i	CKI-1	EFL-1	LIN-35	SCF	APC	CDK-4/CYD-1	CDK-2/CYE-1	CDK-1/CYB-3
2	1	1	1	1	1	0	1	0	0	0	1	1	0

LIN-3	MPK-1	LIN-39	LS	LIN-12m	LIN-12i	CKI-1	EFL-1	LIN-35	SCF	APC	CDK-4/CYD-1	CDK-2/CYE-1	CDK-1/CYB-3
2	2	2	0	1	0	0	1	0	0	0	1	1	0

LIN-3	MPK-1	LIN-39	LS	LIN-12m	LIN-12i	CKI-1	EFL-1	LIN-35	SCF	APC	CDK-4/CYD-1	CDK-2/CYE-1	CDK-1/CYB-3
3	2	2	0	1	0	0	1	0	0	0	1	1	0

LIN-3	MPK-1	LIN-39	LS	LIN-12m	LIN-12i	CKI-1	EFL-1	LIN-35	SCF	APC	CDK-4/CYD-1	CDK-2/CYE-1	CDK-1/CYB-3
3	2	2	0	1	1	0	1	0	0	0	1	1	0

LIN-3	MPK-1	LIN-39	LS	LIN-12m	LIN-12i	CKI-1	EFL-1	LIN-35	SCF	APC	CDK-4/CYD-1	CDK-2/CYE-1	CDK-1/CYB-3
3	2	2	1	1	1	0	1	0	0	0	1	1	0

SCF to APC

LIN-3	MPK-1	LIN-39	LS	LIN-12m	LIN-12i	CKI-1	EFL-1	LIN-35	SCF	APC	CDK-4/CYD-1	CDK-2/CYE-1	CDK-1/CYB-3
2	1	1	1	1	0	0	0	1	0	1	0	0	1
2	2	1	1	1	0	0	0	1	0	1	0	0	0
2	2	2	1	0	1	0	0	1	0	0	1	0	0
2	1	2	1	1	1	0	0	0	0	0	1	0	0
2	1	1	1	1	1	0	1	0	0	0	1	0	0
2	1	1	1	1	1	0	1	0	0	0	1	1	0
2	1	1	1	1	1	0	1	0	1	0	1	1	0
2	1	1	1	1	1	0	1	0	1	0	0	0	0
2	1	1	1	1	1	0	1	1	0	0	0	0	1

LIN-3	MPK-1	LIN-39	LS	LIN-12m	LIN-12i	CKI-1	EFL-1	LIN-35	SCF	APC	CDK-4/CYD-1	CDK-2/CYE-1	CDK-1/CYB-3
2	2	2	0	0	0	0	0	1	0	0	1	0	0
2	2	2	0	1	0	0	0	0	0	0	1	0	0
2	2	2	0	1	0	0	1	0	0	0	1	0	0
2	2	2	0	1	0	0	1	0	0	0	1	1	0
2	2	2	0	1	0	0	1	0	1	0	1	1	0
2	2	2	0	1	0	0	1	0	1	0	0	0	0
2	2	2	0	0	0	0	1	1	0	0	0	0	1
2	2	2	0	0	0	0	0	1	0	1	0	0	1
2	2	2	0	0	0	0	0	1	0	1	0	0	0

LIN-3	MPK-1	LIN-39	LS	LIN-12m	LIN-12i	CKI-1	EFL-1	LIN-35	SCF	APC	CDK-4/CYD-1	CDK-2/CYE-1	CDK-1/CYB-3
3	2	2	0	0	0	0	0	1	0	0	1	0	0
3	2	2	0	1	0	0	0	0	0	0	1	0	0
3	2	2	0	1	0	0	1	0	0	0	1	0	0
3	2	2	0	1	0	0	1	0	0	0	1	1	0
3	2	2	0	1	0	0	1	0	1	0	1	1	0
3	2	2	0	1	0	0	1	0	1	0	0	0	0
3	2	2	0	0	0	0	1	1	0	0	0	0	1

3	2	2	0	0	0	0	0	1	0	1	0	0	1
3	2	2	0	0	0	0	0	1	0	1	0	0	0

LIN-3	MPK-1	LIN-39	LS	LIN-12m	LIN-12i	CKI-1	EFL-1	LIN-35	SCF	APC	CDK-4/CYD-1	CDK-2/CYE-1	CDK-1/CYB-3
3	2	2	1	0	0	0	0	1	0	0	1	0	0
3	2	2	1	1	0	0	0	0	0	0	1	0	0
3	2	2	1	1	1	0	1	0	0	0	1	0	0
3	2	2	1	1	1	0	1	0	0	0	1	1	0
3	2	2	1	1	1	0	1	0	1	0	1	1	0
3	2	2	1	1	1	0	1	0	1	0	0	0	0
3	2	2	1	0	1	0	1	1	0	0	0	0	1
3	2	2	1	0	0	0	0	1	0	1	0	0	1
3	2	2	1	0	0	0	0	1	0	1	0	0	0

LIN-3	MPK-1	LIN-39	LS	LIN-12m	LIN-12i	CKI-1	EFL-1	LIN-35	SCF	APC	CDK-4/CYD-1	CDK-2/CYE-1	CDK-1/CYB-3
1	0	1	0	1	0	1	0	1	0	1	0	0	1
1	1	1	0	1	0	1	0	1	0	1	0	0	0
1	1	1	0	1	1	0	0	1	0	0	0	0	0
1	0	1	0	1	1	0	0	1	0	0	1	0	0
1	0	1	0	1	1	0	0	0	0	0	1	0	0
1	0	1	0	1	1	0	1	0	0	0	1	0	0
1	0	1	0	1	1	0	1	0	0	0	1	1	0
1	0	1	0	1	1	0	1	0	1	0	1	1	0
1	0	1	0	1	1	0	1	0	1	0	0	0	0
1	0	1	0	1	1	0	1	1	0	0	0	0	1

LIN-3	MPK-1	LIN-39	LS	LIN-12m	LIN-12i	CKI-1	EFL-1	LIN-35	SCF	APC	CDK-4/CYD-1	CDK-2/CYE-1	CDK-1/CYB-3
1	0	1	1	1	0	1	0	1	0	1	0	0	1
1	1	1	1	1	0	1	0	1	0	1	0	0	0
1	1	1	1	1	1	0	0	1	0	0	0	0	0
1	0	1	1	1	1	0	0	1	0	0	1	0	0
1	0	1	1	1	1	0	0	0	0	0	1	0	0
1	0	1	1	1	1	0	1	0	0	0	1	0	0
1	0	1	1	1	1	0	1	0	0	0	1	1	0
1	0	1	1	1	1	0	1	0	1	0	1	1	0
1	0	1	1	1	1	0	1	0	1	0	0	0	0
1	0	1	1	1	1	0	1	1	0	0	0	0	1

LIN-3	MPK-1	LIN-39	LS	LIN-12m	LIN-12i	CKI-1	EFL-1	LIN-35	SCF	APC	CDK-4/CYD-1	CDK-2/CYE-1	CDK-1/CYB-3
0	0	1	0	1	0	0	0	0	0	0	1	0	0
0	0	1	0	1	0	0	1	0	0	0	1	0	0
0	0	1	0	1	0	0	1	0	0	0	1	1	0
0	0	1	0	1	0	0	1	0	1	0	1	1	0
0	0	1	0	1	0	0	1	0	1	0	0	0	0
0	0	1	0	1	0	0	1	1	0	0	0	0	1
0	0	1	0	1	0	1	0	1	0	1	0	0	1
0	0	1	0	1	0	1	0	1	0	1	0	0	0
0	0	1	0	1	0	1	0	1	0	0	0	0	0
0	0	1	0	1	0	0	0	1	0	0	0	0	0
0	0	1	0	1	0	0	0	1	0	0	1	0	0

LIN-3	MPK-1	LIN-39	LS	LIN-12m	LIN-12i	CKI-1	EFL-1	LIN-35	SCF	APC	CDK-4/CYD-1	CDK-2/CYE-1	CDK-1/CYB-3
0	0	1	1	1	0	1	0	1	0	1	0	0	0
0	0	1	1	1	1	1	0	1	0	0	0	0	0
0	0	1	1	1	1	0	0	1	0	0	0	0	0
0	0	1	1	1	1	0	0	1	0	0	1	0	0
0	0	1	1	1	1	0	0	0	0	0	1	0	0
0	0	1	1	1	1	0	1	0	0	0	1	0	0
0	0	1	1	1	1	0	1	0	0	0	1	1	0
0	0	1	1	1	1	0	1	0	1	0	1	1	0
0	0	1	1	1	1	0	1	0	1	0	0	0	0

0	0	1	1	1	1	0	1	1	0	0	0	0	1
0	0	1	1	1	0	1	0	1	0	1	0	0	1

CDK-1/CYB-3 to APC

LIN-3	MPK-1	LIN-39	LS	LIN-12m	LIN-12i	CKI-1	EFL-1	LIN-35	SCF	APC	CDK-4/CYD-1	CDK-2/CYE-1	CDK-1/CYB-3
2	1	1	1	1	0	0	0	1	0	0	0	0	1
2	2	1	1	1	0	0	0	1	0	0	0	0	0
2	2	2	1	0	1	0	0	1	0	0	1	0	0
2	1	2	1	1	1	0	0	0	0	0	1	0	0
2	1	1	1	1	1	0	1	0	0	0	1	0	0
2	1	1	1	1	1	0	1	0	0	0	1	1	0
2	1	1	1	1	1	0	1	0	1	0	1	1	0
2	1	1	1	1	1	0	1	0	1	0	0	0	0
2	1	1	1	1	1	0	1	1	0	0	0	0	1

LIN-3	MPK-1	LIN-39	LS	LIN-12m	LIN-12i	CKI-1	EFL-1	LIN-35	SCF	APC	CDK-4/CYD-1	CDK-2/CYE-1	CDK-1/CYB-3
2	2	2	0	0	0	0	0	1	0	0	0	0	0
2	2	2	0	0	0	0	0	1	0	0	1	0	0
2	2	2	0	1	0	0	0	0	0	0	1	0	0
2	2	2	0	1	0	0	1	0	0	0	1	0	0
2	2	2	0	1	0	0	1	0	0	0	1	1	0
2	2	2	0	1	0	0	1	0	1	0	1	1	0
2	2	2	0	1	0	0	1	0	1	0	0	0	0
2	2	2	0	0	0	0	1	1	0	0	0	0	1
2	2	2	0	0	0	0	0	1	0	0	0	0	1

LIN-3	MPK-1	LIN-39	LS	LIN-12m	LIN-12i	CKI-1	EFL-1	LIN-35	SCF	APC	CDK-4/CYD-1	CDK-2/CYE-1	CDK-1/CYB-3
3	2	2	0	0	0	0	0	1	0	0	0	0	0
3	2	2	0	0	0	0	0	1	0	0	1	0	0
3	2	2	0	1	0	0	0	0	0	0	1	0	0
3	2	2	0	1	0	0	1	0	0	0	1	0	0
3	2	2	0	1	0	0	1	0	0	0	1	1	0
3	2	2	0	1	0	0	1	0	1	0	1	1	0
3	2	2	0	1	0	0	1	0	1	0	0	0	0
3	2	2	0	0	0	0	1	1	0	0	0	0	1
3	2	2	0	0	0	0	0	1	0	0	0	0	1

LIN-3	MPK-1	LIN-39	LS	LIN-12m	LIN-12i	CKI-1	EFL-1	LIN-35	SCF	APC	CDK-4/CYD-1	CDK-2/CYE-1	CDK-1/CYB-3
3	2	2	1	0	0	0	0	1	0	0	0	0	0
3	2	2	1	0	0	0	0	1	0	0	1	0	0
3	2	2	1	1	0	0	0	0	0	0	1	0	0
3	2	2	1	1	1	0	1	0	0	0	1	0	0
3	2	2	1	1	1	0	1	0	0	0	1	1	0
3	2	2	1	1	1	0	1	0	1	0	1	1	0
3	2	2	1	1	1	0	1	0	1	0	0	0	0
3	2	2	1	0	1	0	1	1	0	0	0	0	1
3	2	2	1	0	0	0	0	1	0	0	0	0	1

LIN-3	MPK-1	LIN-39	LS	LIN-12m	LIN-12i	CKI-1	EFL-1	LIN-35	SCF	APC	CDK-4/CYD-1	CDK-2/CYE-1	CDK-1/CYB-3
0	0	1	0	1	0	0	0	0	0	0	1	0	0
0	0	1	0	1	0	0	1	0	0	0	1	0	0
0	0	1	0	1	0	0	1	0	0	0	1	1	0
0	0	1	0	1	0	0	1	0	1	0	1	1	0
0	0	1	0	1	0	0	1	0	1	0	0	0	0
0	0	1	0	1	0	0	1	1	0	0	0	0	1
0	0	1	0	1	0	1	0	1	0	0	0	0	1
0	0	1	0	1	0	1	0	1	0	0	0	0	0
0	0	1	0	1	0	0	0	1	0	0	0	0	0
0	0	1	0	1	0	0	0	1	0	0	1	0	0

LIN-3	MPK-1	LIN-39	LS	LIN-12m	LIN-12i	CKI-1	EFL-1	LIN-35	SCF	APC	CDK-4/CYD-1	CDK-2/CYE-1	CDK-1/CYB-3
0	0	1	1	1	0	1	0	1	0	0	0	0	0
0	0	1	1	1	1	0	0	1	0	0	0	0	0
0	0	1	1	1	1	0	0	1	0	0	1	0	0
0	0	1	1	1	1	0	0	0	0	0	1	0	0
0	0	1	1	1	1	0	1	0	0	0	1	0	0
0	0	1	1	1	1	0	1	0	0	0	1	1	0
0	0	1	1	1	1	0	1	0	1	0	1	1	0
0	0	1	1	1	1	0	1	0	1	0	0	0	0
0	0	1	1	1	1	0	1	1	0	0	0	0	1
0	0	1	1	1	0	1	0	1	0	0	0	0	1

LIN-3	MPK-1	LIN-39	LS	LIN-12m	LIN-12i	CKI-1	EFL-1	LIN-35	SCF	APC	CDK-4/CYD-1	CDK-2/CYE-1	CDK-1/CYB-3
1	0	1	0	1	0	1	0	1	0	0	0	0	1
1	1	1	0	1	0	1	0	1	0	0	0	0	0
1	1	1	0	1	1	0	0	1	0	0	0	0	0
1	0	1	0	1	1	0	0	1	0	0	1	0	0
1	0	1	0	1	1	0	0	0	0	0	1	0	0
1	0	1	0	1	1	0	1	0	0	0	1	0	0
1	0	1	0	1	1	0	1	0	0	0	1	1	0
1	0	1	0	1	1	0	1	0	1	0	1	1	0
1	0	1	0	1	1	0	1	0	1	0	0	0	0
1	0	1	0	1	1	0	1	1	0	0	0	0	1

LIN-3	MPK-1	LIN-39	LS	LIN-12m	LIN-12i	CKI-1	EFL-1	LIN-35	SCF	APC	CDK-4/CYD-1	CDK-2/CYE-1	CDK-1/CYB-3
1	0	1	1	1	0	1	0	1	0	0	0	0	1
1	1	1	1	1	0	1	0	1	0	0	0	0	0
1	1	1	1	1	1	0	0	1	0	0	0	0	0
1	0	1	1	1	1	0	0	1	0	0	1	0	0
1	0	1	1	1	1	0	0	0	0	0	1	0	0
1	0	1	1	1	1	0	1	0	0	0	1	0	0
1	0	1	1	1	1	0	1	0	0	0	1	1	0
1	0	1	1	1	1	0	1	0	1	0	1	1	0
1	0	1	1	1	1	0	1	0	1	0	0	0	0
1	0	1	1	1	1	0	1	1	0	0	0	0	1

CKI-1 to CDK-4/CYD-1

LIN-3	MPK-1	LIN-39	LS	LIN-12m	LIN-12i	CKI-1	EFL-1	LIN-35	SCF	APC	CDK-4/CYD-1	CDK-2/CYE-1	CDK-1/CYB-3
0	0	1	0	1	0	0	0	0	0	0	1	0	0
0	0	1	0	1	0	0	1	0	0	0	1	0	0
0	0	1	0	1	0	0	1	0	0	0	1	1	0
0	0	1	0	1	0	0	1	0	1	0	1	1	0
0	0	1	0	1	0	0	1	0	1	0	0	0	0
0	0	1	0	1	0	0	1	1	0	0	0	0	1
0	0	1	0	1	0	1	0	1	0	1	0	0	1
0	0	1	0	1	0	1	0	1	0	1	0	0	0
0	0	1	0	1	0	1	0	1	0	0	1	0	0

LIN-3	MPK-1	LIN-39	LS	LIN-12m	LIN-12i	CKI-1	EFL-1	LIN-35	SCF	APC	CDK-4/CYD-1	CDK-2/CYE-1	CDK-1/CYB-3
0	0	1	1	1	0	1	0	1	0	1	0	0	0
0	0	1	1	1	1	1	0	1	0	0	1	0	0
0	0	1	1	1	1	0	0	0	0	0	1	0	0
0	0	1	1	1	1	0	1	0	0	0	1	0	0
0	0	1	1	1	1	0	1	0	0	0	1	1	0
0	0	1	1	1	1	0	1	0	1	0	1	1	0
0	0	1	1	1	1	0	1	0	1	0	0	0	0
0	0	1	1	1	1	0	1	1	0	0	0	0	1
0	0	1	1	1	0	1	0	1	0	1	0	0	1

LIN-3	MPK-1	LIN-39	LS	LIN-12m	LIN-12i	CKI-1	EFL-1	LIN-35	SCF	APC	CDK-4/CYD-1	CDK-2/CYE-1	CDK-1/CYB-3

1	0	1	0	1	0	1	0	1	0	1	0	0	1
1	1	1	0	1	0	1	0	1	0	1	0	0	0
1	1	1	0	1	1	0	0	1	0	0	1	0	0
1	0	1	0	1	1	0	0	0	0	0	1	0	0
1	0	1	0	1	1	0	1	0	0	0	1	0	0
1	0	1	0	1	1	0	1	0	0	0	1	1	0
1	0	1	0	1	1	0	1	0	1	0	1	1	0
1	0	1	0	1	1	0	1	0	1	0	0	0	0
1	0	1	0	1	1	0	1	1	0	0	0	0	1

LIN-3	MPK-1	LIN-39	LS	LIN-12m	LIN-12i	CKI-1	EFL-1	LIN-35	SCF	APC	CDK-4/CYD-1	CDK-2/CYE-1	CDK-1/CYB-3
1	0	1	1	1	0	1	0	1	0	1	0	0	1
1	1	1	1	1	0	1	0	1	0	1	0	0	0
1	1	1	1	1	1	0	0	1	0	0	1	0	0
1	0	1	1	1	1	0	0	0	0	0	1	0	0
1	0	1	1	1	1	0	1	0	0	0	1	0	0
1	0	1	1	1	1	0	1	0	0	0	1	1	0
1	0	1	1	1	1	0	1	0	1	0	1	1	0
1	0	1	1	1	1	0	1	0	1	0	0	0	0
1	0	1	1	1	1	0	1	1	0	0	0	0	1

LIN-3	MPK-1	LIN-39	LS	LIN-12m	LIN-12i	CKI-1	EFL-1	LIN-35	SCF	APC	CDK-4/CYD-1	CDK-2/CYE-1	CDK-1/CYB-3
2	1	1	1	1	0	0	0	1	0	1	0	0	1
2	2	1	1	1	0	0	0	1	0	1	0	0	0
2	2	2	1	0	1	0	0	1	0	0	1	0	0
2	1	2	1	1	1	0	0	0	0	0	1	0	0
2	1	1	1	1	1	0	1	0	0	0	1	0	0
2	1	1	1	1	1	0	1	0	0	0	1	1	0
2	1	1	1	1	1	0	1	0	1	0	1	1	0
2	1	1	1	1	1	0	1	0	1	0	0	0	0
2	1	1	1	1	1	0	1	1	0	0	0	0	1

LIN-3	MPK-1	LIN-39	LS	LIN-12m	LIN-12i	CKI-1	EFL-1	LIN-35	SCF	APC	CDK-4/CYD-1	CDK-2/CYE-1	CDK-1/CYB-3
2	2	2	0	0	0	0	0	1	0	0	1	0	0
2	2	2	0	1	0	0	0	0	0	0	1	0	0
2	2	2	0	1	0	0	1	0	0	0	1	0	0
2	2	2	0	1	0	0	1	0	0	0	1	1	0
2	2	2	0	1	0	0	1	0	1	0	1	1	0
2	2	2	0	1	0	0	1	0	1	0	0	0	0
2	2	2	0	0	0	0	1	1	0	0	0	0	1
2	2	2	0	0	0	0	0	1	0	1	0	0	1
2	2	2	0	0	0	0	0	1	0	1	0	0	0

LIN-3	MPK-1	LIN-39	LS	LIN-12m	LIN-12i	CKI-1	EFL-1	LIN-35	SCF	APC	CDK-4/CYD-1	CDK-2/CYE-1	CDK-1/CYB-3
3	2	2	0	0	0	0	0	1	0	0	1	0	0
3	2	2	0	1	0	0	0	0	0	0	1	0	0
3	2	2	0	1	0	0	1	0	0	0	1	0	0
3	2	2	0	1	0	0	1	0	0	0	1	1	0
3	2	2	0	1	0	0	1	0	1	0	1	1	0
3	2	2	0	1	0	0	1	0	1	0	0	0	0
3	2	2	0	0	0	0	1	1	0	0	0	0	1
3	2	2	0	0	0	0	0	1	0	1	0	0	1
3	2	2	0	0	0	0	0	1	0	1	0	0	0

LIN-3	MPK-1	LIN-39	LS	LIN-12m	LIN-12i	CKI-1	EFL-1	LIN-35	SCF	APC	CDK-4/CYD-1	CDK-2/CYE-1	CDK-1/CYB-3
3	2	2	1	0	0	0	0	1	0	0	1	0	0
3	2	2	1	1	0	0	0	0	0	0	1	0	0
3	2	2	1	1	1	0	1	0	0	0	1	0	0
3	2	2	1	1	1	0	1	0	0	0	1	1	0
3	2	2	1	1	1	0	1	0	1	0	1	1	0
3	2	2	1	1	1	0	1	0	1	0	0	0	0

3	2	2	1	0	1	0	1	1	0	0	0	0	1
3	2	2	1	0	0	0	0	1	0	1	0	0	1
3	2	2	1	0	0	0	0	1	0	1	0	0	0

SCF to CDK-4/CYD-1

LIN-3	MPK-1	LIN-39	LS	LIN-12m	LIN-12i	CKI-1	EFL-1	LIN-35	SCF	APC	CDK-4/CYD-1	CDK-2/CYE-1	CDK-1/CYB-3
0	0	1	0	1	0	0	1	0	0	0	1	0	0
0	0	1	0	1	0	0	1	0	0	0	1	1	0
0	0	1	0	1	0	0	1	0	1	0	1	1	0
0	0	1	0	1	0	0	1	0	1	0	1	0	0

LIN-3	MPK-1	LIN-39	LS	LIN-12m	LIN-12i	CKI-1	EFL-1	LIN-35	SCF	APC	CDK-4/CYD-1	CDK-2/CYE-1	CDK-1/CYB-3
0	0	1	0	1	1	0	1	0	0	0	1	0	0
0	0	1	0	1	1	0	1	0	0	0	1	1	0
0	0	1	0	1	1	0	1	0	1	0	1	1	0
0	0	1	0	1	1	0	1	0	1	0	1	0	0

LIN-3	MPK-1	LIN-39	LS	LIN-12m	LIN-12i	CKI-1	EFL-1	LIN-35	SCF	APC	CDK-4/CYD-1	CDK-2/CYE-1	CDK-1/CYB-3
0	0	1	1	1	1	0	1	0	0	0	1	0	0
0	0	1	1	1	1	0	1	0	0	0	1	1	0
0	0	1	1	1	1	0	1	0	1	0	1	1	0
0	0	1	1	1	1	0	1	0	1	0	1	0	0

LIN-3	MPK-1	LIN-39	LS	LIN-12m	LIN-12i	CKI-1	EFL-1	LIN-35	SCF	APC	CDK-4/CYD-1	CDK-2/CYE-1	CDK-1/CYB-3
1	0	1	0	1	1	0	1	0	0	0	1	0	0
1	0	1	0	1	1	0	1	0	0	0	1	1	0
1	0	1	0	1	1	0	1	0	1	0	1	1	0
1	0	1	0	1	1	0	1	0	1	0	1	0	0

LIN-3	MPK-1	LIN-39	LS	LIN-12m	LIN-12i	CKI-1	EFL-1	LIN-35	SCF	APC	CDK-4/CYD-1	CDK-2/CYE-1	CDK-1/CYB-3
1	0	1	1	1	1	0	1	0	0	0	1	0	0
1	0	1	1	1	1	0	1	0	0	0	1	1	0
1	0	1	1	1	1	0	1	0	1	0	1	1	0
1	0	1	1	1	1	0	1	0	1	0	1	0	0

LIN-3	MPK-1	LIN-39	LS	LIN-12m	LIN-12i	CKI-1	EFL-1	LIN-35	SCF	APC	CDK-4/CYD-1	CDK-2/CYE-1	CDK-1/CYB-3
2	1	1	0	1	1	0	1	0	0	0	1	0	0
2	1	1	0	1	1	0	1	0	0	0	1	1	0
2	1	1	0	1	1	0	1	0	1	0	1	1	0
2	1	1	0	1	1	0	1	0	1	0	1	0	0

LIN-3	MPK-1	LIN-39	LS	LIN-12m	LIN-12i	CKI-1	EFL-1	LIN-35	SCF	APC	CDK-4/CYD-1	CDK-2/CYE-1	CDK-1/CYB-3
2	1	1	1	1	1	0	1	0	0	0	1	0	0
2	1	1	1	1	1	0	1	0	0	0	1	1	0
2	1	1	1	1	1	0	1	0	1	0	1	1	0
2	1	1	1	1	1	0	1	0	1	0	1	0	0

LIN-3	MPK-1	LIN-39	LS	LIN-12m	LIN-12i	CKI-1	EFL-1	LIN-35	SCF	APC	CDK-4/CYD-1	CDK-2/CYE-1	CDK-1/CYB-3
2	2	2	0	1	0	0	1	0	0	0	1	0	0
2	2	2	0	1	0	0	1	0	0	0	1	1	0
2	2	2	0	1	0	0	1	0	1	0	1	1	0
2	2	2	0	1	0	0	1	0	1	0	1	0	0

LIN-3	MPK-1	LIN-39	LS	LIN-12m	LIN-12i	CKI-1	EFL-1	LIN-35	SCF	APC	CDK-4/CYD-1	CDK-2/CYE-1	CDK-1/CYB-3
3	2	2	0	1	0	0	1	0	0	0	1	0	0
3	2	2	0	1	0	0	1	0	0	0	1	1	0
3	2	2	0	1	0	0	1	0	1	0	1	1	0
3	2	2	0	1	0	0	1	0	1	0	1	0	0

LIN-3	MPK-1	LIN-39	LS	LIN-12m	LIN-12i	CKI-1	EFL-1	LIN-35	SCF	APC	CDK-4/CYD-1	CDK-2/CYE-1	CDK-1/CYB-3

3	2	2	0	1	1	0	1	0	0	0	1	0	0
3	2	2	0	1	1	0	1	0	0	0	1	1	0
3	2	2	0	1	1	0	1	0	1	0	1	1	0
3	2	2	0	1	1	0	1	0	1	0	1	0	0

LIN-3	MPK-1	LIN-39	LS	LIN-12m	LIN-12i	CKI-1	EFL-1	LIN-35	SCF	APC	CDK-4/CYD-1	CDK-2/CYE-1	CDK-1/CYB-3
3	2	2	1	1	1	0	1	0	0	0	1	0	0
3	2	2	1	1	1	0	1	0	0	0	1	1	0
3	2	2	1	1	1	0	1	0	1	0	1	1	0
3	2	2	1	1	1	0	1	0	1	0	1	0	0

CDK-1/CYB-3 to CDK-4/CYD-1

LIN-3	MPK-1	LIN-39	LS	LIN-12m	LIN-12i	CKI-1	EFL-1	LIN-35	SCF	APC	CDK-4/CYD-1	CDK-2/CYE-1	CDK-1/CYB-3
0	0	1	0	1	0	0	0	0	1	1	1	0	1
0	0	1	0	1	0	0	1	0	0	0	0	0	0
0	0	1	0	1	0	0	1	1	0	0	1	1	1

LIN-3	MPK-1	LIN-39	LS	LIN-12m	LIN-12i	CKI-1	EFL-1	LIN-35	SCF	APC	CDK-4/CYD-1	CDK-2/CYE-1	CDK-1/CYB-3
0	0	1	1	1	0	0	1	0	0	0	0	0	0
0	0	1	1	1	1	0	1	1	0	0	1	1	1
0	0	1	1	1	1	0	0	0	1	1	1	0	1

LIN-3	MPK-1	LIN-39	LS	LIN-12m	LIN-12i	CKI-1	EFL-1	LIN-35	SCF	APC	CDK-4/CYD-1	CDK-2/CYE-1	CDK-1/CYB-3
1	0	1	0	1	0	0	1	0	0	0	0	0	0
1	1	1	0	1	1	0	1	1	0	0	1	1	1
1	0	1	0	1	1	0	0	0	1	1	1	0	1

LIN-3	MPK-1	LIN-39	LS	LIN-12m	LIN-12i	CKI-1	EFL-1	LIN-35	SCF	APC	CDK-4/CYD-1	CDK-2/CYE-1	CDK-1/CYB-3
1	0	1	1	1	0	0	1	0	0	0	0	0	0
1	1	1	1	1	1	0	1	1	0	0	1	1	1
1	0	1	1	1	1	0	0	0	1	1	1	0	1

LIN-3	MPK-1	LIN-39	LS	LIN-12m	LIN-12i	CKI-1	EFL-1	LIN-35	SCF	APC	CDK-4/CYD-1	CDK-2/CYE-1	CDK-1/CYB-3
2	1	1	1	1	0	0	1	0	0	0	0	0	0
2	2	1	1	1	1	0	1	1	0	0	1	1	1
2	1	2	1	1	1	0	0	0	1	1	1	0	1

LIN-3	MPK-1	LIN-39	LS	LIN-12m	LIN-12i	CKI-1	EFL-1	LIN-35	SCF	APC	CDK-4/CYD-1	CDK-2/CYE-1	CDK-1/CYB-3
2	2	2	0	0	0	0	1	1	0	0	1	1	1
2	2	2	0	1	0	0	0	0	1	1	1	0	1
2	2	2	0	1	0	0	1	0	0	0	0	0	0

LIN-3	MPK-1	LIN-39	LS	LIN-12m	LIN-12i	CKI-1	EFL-1	LIN-35	SCF	APC	CDK-4/CYD-1	CDK-2/CYE-1	CDK-1/CYB-3
3	2	2	0	0	0	0	1	1	0	0	1	1	1
3	2	2	0	1	0	0	0	0	1	1	1	0	1
3	2	2	0	1	0	0	1	0	0	0	0	0	0

LIN-3	MPK-1	LIN-39	LS	LIN-12m	LIN-12i	CKI-1	EFL-1	LIN-35	SCF	APC	CDK-4/CYD-1	CDK-2/CYE-1	CDK-1/CYB-3
3	2	2	1	0	1	0	1	1	0	0	1	1	1
3	2	2	1	1	1	0	0	0	1	1	1	0	1
3	2	2	1	1	0	0	1	0	0	0	0	0	0

LIN-3	MPK-1	LIN-39	LS	LIN-12m	LIN-12i	CKI-1	EFL-1	LIN-35	SCF	APC	CDK-4/CYD-1	CDK-2/CYE-1	CDK-1/CYB-3
2	1	1	1	1	0	0	0	1	0	1	1	0	1
2	2	1	1	1	0	0	0	0	0	1	1	0	0
2	2	2	1	1	1	0	1	0	0	0	1	0	0
2	1	2	1	1	1	0	1	0	0	0	1	1	0
2	1	1	1	1	1	0	1	0	1	0	1	1	0
2	1	1	1	1	1	0	1	0	1	0	0	0	0
2	1	1	1	1	1	0	1	1	0	0	0	0	1

LIN-3	MPK-1	LIN-39	LS	LIN-12m	LIN-12i	CKI-1	EFL-1	LIN-35	SCF	APC	CDK-4/CYD-1	CDK-2/CYE-1	CDK-1/CYB-3
2	2	2	0	0	0	0	0	1	0	1	1	0	1
2	2	2	0	1	0	0	0	0	0	1	1	0	0
2	2	2	0	1	0	0	1	0	0	0	1	0	0
2	2	2	0	1	0	0	1	0	0	0	1	1	0
2	2	2	0	1	0	0	1	0	1	0	1	1	0
2	2	2	0	1	0	0	1	0	1	0	0	0	0
2	2	2	0	0	0	0	1	1	0	0	0	0	1

LIN-3	MPK-1	LIN-39	LS	LIN-12m	LIN-12i	CKI-1	EFL-1	LIN-35	SCF	APC	CDK-4/CYD-1	CDK-2/CYE-1	CDK-1/CYB-3
3	2	2	0	0	0	0	0	1	0	1	1	0	1
3	2	2	0	1	0	0	0	0	0	1	1	0	0
3	2	2	0	1	0	0	1	0	0	0	1	0	0
3	2	2	0	1	0	0	1	0	0	0	1	1	0
3	2	2	0	1	0	0	1	0	1	0	1	1	0
3	2	2	0	1	0	0	1	0	1	0	0	0	0
3	2	2	0	0	0	0	1	1	0	0	0	0	1

LIN-3	MPK-1	LIN-39	LS	LIN-12m	LIN-12i	CKI-1	EFL-1	LIN-35	SCF	APC	CDK-4/CYD-1	CDK-2/CYE-1	CDK-1/CYB-3
3	2	2	1	0	0	0	0	1	0	1	1	0	1
3	2	2	1	1	0	0	0	0	0	1	1	0	0
3	2	2	1	1	1	0	1	0	0	0	1	0	0
3	2	2	1	1	1	0	1	0	0	0	1	1	0
3	2	2	1	1	1	0	1	0	1	0	1	1	0
3	2	2	1	1	1	0	1	0	1	0	0	0	0
3	2	2	1	0	1	0	1	1	0	0	0	0	1

LIN-3	MPK-1	LIN-39	LS	LIN-12m	LIN-12i	CKI-1	EFL-1	LIN-35	SCF	APC	CDK-4/CYD-1	CDK-2/CYE-1	CDK-1/CYB-3
1	0	1	0	1	0	1	0	1	0	1	1	0	1
1	1	1	0	1	0	0	0	0	0	1	0	0	0
1	1	1	0	1	1	0	1	1	0	0	1	0	0
1	0	1	0	1	1	0	0	0	0	0	1	0	0
1	0	1	0	1	1	0	1	0	0	0	1	0	0
1	0	1	0	1	1	0	1	0	0	0	1	1	0
1	0	1	0	1	1	0	1	0	1	0	1	1	0
1	0	1	0	1	1	0	1	0	1	0	0	0	0
1	0	1	0	1	1	0	1	1	0	0	0	0	1

LIN-3	MPK-1	LIN-39	LS	LIN-12m	LIN-12i	CKI-1	EFL-1	LIN-35	SCF	APC	CDK-4/CYD-1	CDK-2/CYE-1	CDK-1/CYB-3
1	0	1	1	1	0	1	0	1	0	1	1	0	1
1	1	1	1	1	0	0	0	0	0	1	0	0	0
1	1	1	1	1	1	0	1	1	0	0	1	0	0
1	0	1	1	1	1	0	0	0	0	0	1	0	0
1	0	1	1	1	1	0	1	0	0	0	1	0	0
1	0	1	1	1	1	0	1	0	0	0	1	1	0
1	0	1	1	1	1	0	1	0	1	0	1	1	0
1	0	1	1	1	1	0	1	0	1	0	0	0	0
1	0	1	1	1	1	0	1	1	0	0	0	0	1

LIN-3	MPK-1	LIN-39	LS	LIN-12m	LIN-12i	CKI-1	EFL-1	LIN-35	SCF	APC	CDK-4/CYD-1	CDK-2/CYE-1	CDK-1/CYB-3
0	0	1	0	1	0	0	0	0	0	0	0	0	0
0	0	1	0	1	0	0	1	1	0	0	1	0	0
0	0	1	0	1	0	0	0	0	0	0	1	0	0
0	0	1	0	1	0	0	1	0	0	0	1	0	0
0	0	1	0	1	0	0	1	0	0	0	1	1	0
0	0	1	0	1	0	0	1	0	1	0	1	1	0
0	0	1	0	1	0	0	1	0	1	0	0	0	0
0	0	1	0	1	0	0	1	1	0	0	0	0	1
0	0	1	0	1	0	1	0	1	0	1	1	0	1
0	0	1	0	1	0	0	0	0	0	1	0	0	0

0	0	1	0	1	0	1	1	1	0	0	1	0	0

LIN-3	MPK-1	LIN-39	LS	LIN-12m	LIN-12i	CKI-1	EFL-1	LIN-35	SCF	APC	CDK-4/CYD-1	CDK-2/CYE-1	CDK-1/CYB-3
0	0	1	1	1	0	0	0	0	0	1	0	0	0
0	0	1	1	1	1	1	1	1	0	0	1	0	0
0	0	1	1	1	1	0	0	0	0	0	0	0	0
0	0	1	1	1	1	0	1	1	0	0	1	0	0
0	0	1	1	1	1	0	0	0	0	0	1	0	0
0	0	1	1	1	1	0	1	0	0	0	1	0	0
0	0	1	1	1	1	0	1	0	0	0	1	1	0
0	0	1	1	1	1	0	1	0	1	0	1	1	0
0	0	1	1	1	1	0	1	0	1	0	0	0	0
0	0	1	1	1	1	0	1	1	0	0	0	0	1
0	0	1	1	1	0	1	0	1	0	1	1	0	1

CKI-1 to CDK-2/CYE-1

LIN-3	MPK-1	LIN-39	LS	LIN-12m	LIN-12i	CKI-1	EFL-1	LIN-35	SCF	APC	CDK-4/CYD-1	CDK-2/CYE-1	CDK-1/CYB-3
2	1	1	1	1	0	0	0	1	0	1	0	0	1
2	2	1	1	1	0	0	0	1	0	1	0	0	0
2	2	2	1	0	1	0	0	1	0	0	1	0	0
2	1	2	1	1	1	0	0	0	0	0	1	0	0
2	1	1	1	1	1	0	1	0	0	0	1	0	0
2	1	1	1	1	1	0	1	0	0	0	1	1	0
2	1	1	1	1	1	0	1	0	1	0	1	1	0
2	1	1	1	1	1	0	1	0	1	0	0	0	0
2	1	1	1	1	1	0	1	1	0	0	0	0	1

LIN-3	MPK-1	LIN-39	LS	LIN-12m	LIN-12i	CKI-1	EFL-1	LIN-35	SCF	APC	CDK-4/CYD-1	CDK-2/CYE-1	CDK-1/CYB-3
2	2	2	0	0	0	0	0	1	0	0	1	0	0
2	2	2	0	1	0	0	0	0	0	0	1	0	0
2	2	2	0	1	0	0	1	0	0	0	1	0	0
2	2	2	0	1	0	0	1	0	0	0	1	1	0
2	2	2	0	1	0	0	1	0	1	0	1	1	0
2	2	2	0	1	0	0	1	0	1	0	0	0	0
2	2	2	0	0	0	0	1	1	0	0	0	0	1
2	2	2	0	0	0	0	0	1	0	1	0	0	1
2	2	2	0	0	0	0	0	1	0	1	0	0	0

LIN-3	MPK-1	LIN-39	LS	LIN-12m	LIN-12i	CKI-1	EFL-1	LIN-35	SCF	APC	CDK-4/CYD-1	CDK-2/CYE-1	CDK-1/CYB-3
3	2	2	0	0	0	0	0	1	0	0	1	0	0
3	2	2	0	1	0	0	0	0	0	0	1	0	0
3	2	2	0	1	0	0	1	0	0	0	1	0	0
3	2	2	0	1	0	0	1	0	0	0	1	1	0
3	2	2	0	1	0	0	1	0	1	0	1	1	0
3	2	2	0	1	0	0	1	0	1	0	0	0	0
3	2	2	0	0	0	0	1	1	0	0	0	0	1
3	2	2	0	0	0	0	0	1	0	1	0	0	1
3	2	2	0	0	0	0	0	1	0	1	0	0	0

LIN-3	MPK-1	LIN-39	LS	LIN-12m	LIN-12i	CKI-1	EFL-1	LIN-35	SCF	APC	CDK-4/CYD-1	CDK-2/CYE-1	CDK-1/CYB-3
3	2	2	1	0	0	0	0	1	0	0	1	0	0
3	2	2	1	1	0	0	0	0	0	0	1	0	0
3	2	2	1	1	1	0	1	0	0	0	1	0	0
3	2	2	1	1	1	0	1	0	0	0	1	1	0
3	2	2	1	1	1	0	1	0	1	0	1	1	0
3	2	2	1	1	1	0	1	0	1	0	0	0	0
3	2	2	1	0	1	0	1	1	0	0	0	0	1
3	2	2	1	0	0	0	0	1	0	1	0	0	1
3	2	2	1	0	0	0	0	1	0	1	0	0	0

LIN-3	MPK-1	LIN-39	LS	LIN-12m	LIN-12i	CKI-1	EFL-1	LIN-35	SCF	APC	CDK-4/CYD-1	CDK-2/CYE-1	CDK-1/CYB-3
1	0	1	0	1	0	1	0	1	0	1	0	0	1
1	1	1	0	1	0	1	0	1	0	1	0	0	0
1	1	1	0	1	1	0	0	1	0	0	0	0	0
1	0	1	0	1	1	0	0	1	0	0	1	0	0
1	0	1	0	1	1	0	0	0	0	0	1	0	0
1	0	1	0	1	1	0	1	0	0	0	1	0	0
1	0	1	0	1	1	0	1	0	0	0	1	1	0
1	0	1	0	1	1	0	1	0	1	0	1	1	0
1	0	1	0	1	1	0	1	0	1	0	0	0	0
1	0	1	0	1	1	0	1	1	0	0	0	0	1

LIN-3	MPK-1	LIN-39	LS	LIN-12m	LIN-12i	CKI-1	EFL-1	LIN-35	SCF	APC	CDK-4/CYD-1	CDK-2/CYE-1	CDK-1/CYB-3
1	0	1	1	1	0	1	0	1	0	1	0	0	1
1	1	1	1	1	0	1	0	1	0	1	0	0	0
1	1	1	1	1	1	0	0	1	0	0	0	0	0
1	0	1	1	1	1	0	0	1	0	0	1	0	0
1	0	1	1	1	1	0	0	0	0	0	1	0	0
1	0	1	1	1	1	0	1	0	0	0	1	0	0
1	0	1	1	1	1	0	1	0	0	0	1	1	0
1	0	1	1	1	1	0	1	0	1	0	1	1	0
1	0	1	1	1	1	0	1	0	1	0	0	0	0
1	0	1	1	1	1	0	1	1	0	0	0	0	1

LIN-3	MPK-1	LIN-39	LS	LIN-12m	LIN-12i	CKI-1	EFL-1	LIN-35	SCF	APC	CDK-4/CYD-1	CDK-2/CYE-1	CDK-1/CYB-3
0	0	1	0	1	0	0	0	0	0	0	1	0	0
0	0	1	0	1	0	0	1	0	0	0	1	0	0
0	0	1	0	1	0	0	1	0	0	0	1	1	0
0	0	1	0	1	0	0	1	0	1	0	1	1	0
0	0	1	0	1	0	0	1	0	1	0	0	0	0
0	0	1	0	1	0	0	1	1	0	0	0	0	1
0	0	1	0	1	0	1	0	1	0	1	0	0	1
0	0	1	0	1	0	1	0	1	0	1	0	0	0
0	0	1	0	1	0	1	0	1	0	0	0	0	0
0	0	1	0	1	0	0	0	1	0	0	0	0	0
0	0	1	0	1	0	0	0	1	0	0	1	0	0

LIN-3	MPK-1	LIN-39	LS	LIN-12m	LIN-12i	CKI-1	EFL-1	LIN-35	SCF	APC	CDK-4/CYD-1	CDK-2/CYE-1	CDK-1/CYB-3
0	0	1	1	1	0	1	0	1	0	1	0	0	0
0	0	1	1	1	1	1	0	1	0	0	0	0	0
0	0	1	1	1	1	0	0	1	0	0	0	0	0
0	0	1	1	1	1	0	0	1	0	0	1	0	0
0	0	1	1	1	1	0	0	0	0	0	1	0	0
0	0	1	1	1	1	0	1	0	0	0	1	0	0
0	0	1	1	1	1	0	1	0	0	0	1	1	0
0	0	1	1	1	1	0	1	0	1	0	1	1	0
0	0	1	1	1	1	0	1	0	1	0	0	0	0
0	0	1	1	1	1	0	1	1	0	0	0	0	1
0	0	1	1	1	0	1	0	1	0	1	0	0	1

EFL-1 to CDK-2/CYE-1

LIN-3	MPK-1	LIN-39	LS	LIN-12m	LIN-12i	CKI-1	EFL-1	LIN-35	SCF	APC	CDK-4/CYD-1	CDK-2/CYE-1	CDK-1/CYB-3
0	0	1	0	1	0	0	1	0	0	0	1	0	0

LIN-3	MPK-1	LIN-39	LS	LIN-12m	LIN-12i	CKI-1	EFL-1	LIN-35	SCF	APC	CDK-4/CYD-1	CDK-2/CYE-1	CDK-1/CYB-3
0	0	1	0	1	1	0	1	0	0	0	1	0	0

LIN-3	MPK-1	LIN-39	LS	LIN-12m	LIN-12i	CKI-1	EFL-1	LIN-35	SCF	APC	CDK-4/CYD-1	CDK-2/CYE-1	CDK-1/CYB-3
0	0	1	1	1	1	0	1	0	0	0	1	0	0

LIN-3	MPK-1	LIN-39	LS	LIN-12m	LIN-12i	CKI-1	EFL-1	LIN-35	SCF	APC	CDK-4/CYD-1	CDK-2/CYE-1	CDK-1/CYB-3
1	0	1	0	1	1	0	1	0	0	0	1	0	0

LIN-3	MPK-1	LIN-39	LS	LIN-12m	LIN-12i	CKI-1	EFL-1	LIN-35	SCF	APC	CDK-4/CYD-1	CDK-2/CYE-1	CDK-1/CYB-3
1	0	1	1	1	1	0	1	0	0	0	1	0	0

LIN-3	MPK-1	LIN-39	LS	LIN-12m	LIN-12i	CKI-1	EFL-1	LIN-35	SCF	APC	CDK-4/CYD-1	CDK-2/CYE-1	CDK-1/CYB-3
2	1	1	0	1	1	0	1	0	0	0	1	0	0

LIN-3	MPK-1	LIN-39	LS	LIN-12m	LIN-12i	CKI-1	EFL-1	LIN-35	SCF	APC	CDK-4/CYD-1	CDK-2/CYE-1	CDK-1/CYB-3
2	1	1	1	1	1	0	1	0	0	0	1	0	0

LIN-3	MPK-1	LIN-39	LS	LIN-12m	LIN-12i	CKI-1	EFL-1	LIN-35	SCF	APC	CDK-4/CYD-1	CDK-2/CYE-1	CDK-1/CYB-3
2	2	2	0	1	0	0	1	0	0	0	1	0	0

LIN-3	MPK-1	LIN-39	LS	LIN-12m	LIN-12i	CKI-1	EFL-1	LIN-35	SCF	APC	CDK-4/CYD-1	CDK-2/CYE-1	CDK-1/CYB-3
3	2	2	0	1	0	0	1	0	0	0	1	0	0

LIN-3	MPK-1	LIN-39	LS	LIN-12m	LIN-12i	CKI-1	EFL-1	LIN-35	SCF	APC	CDK-4/CYD-1	CDK-2/CYE-1	CDK-1/CYB-3
3	2	2	0	1	1	0	1	0	0	0	1	0	0

LIN-3	MPK-1	LIN-39	LS	LIN-12m	LIN-12i	CKI-1	EFL-1	LIN-35	SCF	APC	CDK-4/CYD-1	CDK-2/CYE-1	CDK-1/CYB-3
3	2	2	1	1	1	0	1	0	0	0	1	0	0

LIN-35 to CDK-2/CYE-1

LIN-3	MPK-1	LIN-39	LS	LIN-12m	LIN-12i	CKI-1	EFL-1	LIN-35	SCF	APC	CDK-4/CYD-1	CDK-2/CYE-1	CDK-1/CYB-3
2	1	1	0	1	1	0	0	0	1	1	0	0	0
2	1	1	0	1	1	0	1	1	0	0	0	0	0
2	1	1	0	1	1	0	0	1	0	0	1	1	1

LIN-3	MPK-1	LIN-39	LS	LIN-12m	LIN-12i	CKI-1	EFL-1	LIN-35	SCF	APC	CDK-4/CYD-1	CDK-2/CYE-1	CDK-1/CYB-3
2	1	1	0	1	1	0	0	0	1	1	0	1	1
2	1	1	0	1	1	0	1	0	0	0	0	0	0
2	1	1	0	1	1	0	1	1	0	0	1	1	1

LIN-3	MPK-1	LIN-39	LS	LIN-12m	LIN-12i	CKI-1	EFL-1	LIN-35	SCF	APC	CDK-4/CYD-1	CDK-2/CYE-1	CDK-1/CYB-3
2	1	1	1	1	1	0	0	0	1	1	0	0	0
2	1	1	1	1	1	0	1	1	0	0	0	0	0
2	1	1	1	1	1	0	0	1	0	0	1	1	1

LIN-3	MPK-1	LIN-39	LS	LIN-12m	LIN-12i	CKI-1	EFL-1	LIN-35	SCF	APC	CDK-4/CYD-1	CDK-2/CYE-1	CDK-1/CYB-3
2	1	1	1	1	1	0	0	0	1	1	0	1	1
2	1	1	1	1	1	0	1	0	0	0	0	0	0
2	1	1	1	1	1	0	1	1	0	0	1	1	1

LIN-3	MPK-1	LIN-39	LS	LIN-12m	LIN-12i	CKI-1	EFL-1	LIN-35	SCF	APC	CDK-4/CYD-1	CDK-2/CYE-1	CDK-1/CYB-3
2	2	2	0	0	0	0	0	1	0	0	1	1	1
2	2	2	0	1	0	0	0	0	1	1	0	0	0
2	2	2	0	0	0	0	1	1	0	0	0	0	0

LIN-3	MPK-1	LIN-39	LS	LIN-12m	LIN-12i	CKI-1	EFL-1	LIN-35	SCF	APC	CDK-4/CYD-1	CDK-2/CYE-1	CDK-1/CYB-3
2	2	2	0	0	0	0	1	0	0	0	0	0	0
2	2	2	0	0	0	0	1	1	0	0	1	1	1
2	2	2	0	1	0	0	0	0	1	1	0	1	1

LIN-3	MPK-1	LIN-39	LS	LIN-12m	LIN-12i	CKI-1	EFL-1	LIN-35	SCF	APC	CDK-4/CYD-1	CDK-2/CYE-1	CDK-1/CYB-3
3	2	2	0	0	0	0	0	1	0	0	1	1	1
3	2	2	0	1	0	0	0	0	1	1	0	0	0
3	2	2	0	0	0	0	1	1	0	0	0	0	0

Interactions

LIN-3	MPK-1	LIN-39	LS	LIN-12m	LIN-12i	CKI-1	EFL-1	LIN-35	SCF	APC	CDK-4/CYD-1	CDK-2/CYE-1	CDK-1/CYB-3
3	2	2	0	0	0	0	1	0	0	0	0	0	0
3	2	2	0	0	0	0	1	1	0	0	1	1	1
3	2	2	0	1	0	0	0	0	1	1	0	1	1

LIN-3	MPK-1	LIN-39	LS	LIN-12m	LIN-12i	CKI-1	EFL-1	LIN-35	SCF	APC	CDK-4/CYD-1	CDK-2/CYE-1	CDK-1/CYB-3
3	2	2	0	0	1	0	0	1	0	0	1	1	1
3	2	2	0	1	1	0	0	0	1	1	0	0	0
3	2	2	0	0	1	0	1	1	0	0	0	0	0

LIN-3	MPK-1	LIN-39	LS	LIN-12m	LIN-12i	CKI-1	EFL-1	LIN-35	SCF	APC	CDK-4/CYD-1	CDK-2/CYE-1	CDK-1/CYB-3
3	2	2	0	0	1	0	1	0	0	0	0	0	0
3	2	2	0	0	1	0	1	1	0	0	1	1	1
3	2	2	0	1	1	0	0	0	1	1	0	1	1

LIN-3	MPK-1	LIN-39	LS	LIN-12m	LIN-12i	CKI-1	EFL-1	LIN-35	SCF	APC	CDK-4/CYD-1	CDK-2/CYE-1	CDK-1/CYB-3
3	2	2	1	0	1	0	0	1	0	0	1	1	1
3	2	2	1	1	1	0	0	0	1	1	0	0	0
3	2	2	1	0	1	0	1	1	0	0	0	0	0

LIN-3	MPK-1	LIN-39	LS	LIN-12m	LIN-12i	CKI-1	EFL-1	LIN-35	SCF	APC	CDK-4/CYD-1	CDK-2/CYE-1	CDK-1/CYB-3
3	2	2	1	0	1	0	1	0	0	0	0	0	0
3	2	2	1	0	1	0	1	1	0	0	1	1	1
3	2	2	1	1	1	0	0	0	1	1	0	1	1

LIN-3	MPK-1	LIN-39	LS	LIN-12m	LIN-12i	CKI-1	EFL-1	LIN-35	SCF	APC	CDK-4/CYD-1	CDK-2/CYE-1	CDK-1/CYB-3
0	0	1	0	1	0	0	0	0	0	0	1	0	0
0	0	1	0	1	0	0	1	0	0	0	1	0	0
0	0	1	0	1	0	0	1	0	0	0	1	1	0
0	0	1	0	1	0	0	1	0	1	0	1	1	0
0	0	1	0	1	0	0	1	0	1	0	0	0	0
0	0	1	0	1	0	0	1	1	0	0	0	0	1
0	0	1	0	1	0	1	0	1	0	1	0	1	1
0	0	1	0	1	0	1	0	0	0	1	0	0	0
0	0	1	0	1	0	1	1	1	0	0	0	0	0
0	0	1	0	1	0	0	0	1	0	0	0	0	0
0	0	1	0	1	0	0	0	1	0	0	1	0	0

LIN-3	MPK-1	LIN-39	LS	LIN-12m	LIN-12i	CKI-1	EFL-1	LIN-35	SCF	APC	CDK-4/CYD-1	CDK-2/CYE-1	CDK-1/CYB-3
0	0	1	1	1	0	1	0	1	0	1	0	1	1
0	0	1	1	1	1	1	0	0	0	1	0	0	0
0	0	1	1	1	1	1	1	1	0	0	0	0	0
0	0	1	1	1	1	0	0	1	0	0	0	0	0
0	0	1	1	1	1	0	0	1	0	0	1	0	0
0	0	1	1	1	1	0	0	0	0	0	1	0	0
0	0	1	1	1	1	0	1	0	0	0	1	0	0
0	0	1	1	1	1	0	1	0	0	0	1	1	0
0	0	1	1	1	1	0	1	0	1	0	1	1	0
0	0	1	1	1	1	0	1	0	1	0	0	0	0
0	0	1	1	1	1	0	1	1	0	0	0	0	1

LIN-3	MPK-1	LIN-39	LS	LIN-12m	LIN-12i	CKI-1	EFL-1	LIN-35	SCF	APC	CDK-4/CYD-1	CDK-2/CYE-1	CDK-1/CYB-3
1	0	1	0	1	0	1	0	1	0	1	0	1	1
1	1	1	0	1	1	1	0	0	0	1	0	0	0
1	0	1	0	1	1	0	1	1	0	0	0	0	0
1	0	1	0	1	1	0	0	1	0	0	1	1	1
1	0	1	0	1	1	0	0	0	1	1	0	0	0
1	0	1	0	1	1	1	1	1	0	0	0	0	0
1	0	1	0	1	1	0	0	1	0	0	0	0	0
1	0	1	0	1	1	0	0	1	0	0	1	0	0
1	0	1	0	1	1	0	0	0	0	0	1	0	0

1	0	1	0	1	1	0	1	0	0	0	1	0	0
1	0	1	0	1	1	0	1	0	0	0	1	1	0
1	0	1	0	1	1	0	1	0	1	0	1	1	0
1	0	1	0	1	1	0	1	0	1	0	0	0	0
1	0	1	0	1	1	0	1	1	0	0	0	0	1

LIN-3	MPK-1	LIN-39	LS	LIN-12m	LIN-12i	CKI-1	EFL-1	LIN-35	SCF	APC	CDK-4/CYD-1	CDK-2/CYE-1	CDK-1/CYB-3
1	0	1	1	1	0	1	0	1	0	1	0	1	1
1	1	1	1	1	1	1	0	0	0	1	0	0	0
1	0	1	1	1	1	0	1	1	0	0	0	0	0
1	0	1	1	1	1	0	0	1	0	0	1	1	1
1	0	1	1	1	1	0	0	0	1	1	0	0	0
1	0	1	1	1	1	1	1	1	0	0	0	0	0
1	0	1	1	1	1	0	0	1	0	0	0	0	0
1	0	1	1	1	1	0	0	1	0	0	1	0	0
1	0	1	1	1	1	0	0	0	0	0	1	0	0
1	0	1	1	1	1	0	1	0	0	0	1	0	0
1	0	1	1	1	1	0	1	0	0	0	1	1	0
1	0	1	1	1	1	0	1	0	1	0	1	1	0
1	0	1	1	1	1	0	1	0	1	0	0	0	0
1	0	1	1	1	1	0	1	1	0	0	0	0	1

SCF to CDK-2/CYE-1

LIN-3	MPK-1	LIN-39	LS	LIN-12m	LIN-12i	CKI-1	EFL-1	LIN-35	SCF	APC	CDK-4/CYD-1	CDK-2/CYE-1	CDK-1/CYB-3
2	1	1	0	1	1	0	1	0	1	0	0	1	1

LIN-3	MPK-1	LIN-39	LS	LIN-12m	LIN-12i	CKI-1	EFL-1	LIN-35	SCF	APC	CDK-4/CYD-1	CDK-2/CYE-1	CDK-1/CYB-3
2	1	1	1	1	1	0	1	0	1	0	0	1	1

LIN-3	MPK-1	LIN-39	LS	LIN-12m	LIN-12i	CKI-1	EFL-1	LIN-35	SCF	APC	CDK-4/CYD-1	CDK-2/CYE-1	CDK-1/CYB-3
2	2	2	0	0	0	0	1	0	1	0	0	1	1

LIN-3	MPK-1	LIN-39	LS	LIN-12m	LIN-12i	CKI-1	EFL-1	LIN-35	SCF	APC	CDK-4/CYD-1	CDK-2/CYE-1	CDK-1/CYB-3
2	2	2	1	0	0	0	1	0	1	0	0	1	1

LIN-3	MPK-1	LIN-39	LS	LIN-12m	LIN-12i	CKI-1	EFL-1	LIN-35	SCF	APC	CDK-4/CYD-1	CDK-2/CYE-1	CDK-1/CYB-3
3	2	2	0	0	0	0	1	0	1	0	0	1	1

LIN-3	MPK-1	LIN-39	LS	LIN-12m	LIN-12i	CKI-1	EFL-1	LIN-35	SCF	APC	CDK-4/CYD-1	CDK-2/CYE-1	CDK-1/CYB-3
3	2	2	0	0	1	0	1	0	1	0	0	1	1

LIN-3	MPK-1	LIN-39	LS	LIN-12m	LIN-12i	CKI-1	EFL-1	LIN-35	SCF	APC	CDK-4/CYD-1	CDK-2/CYE-1	CDK-1/CYB-3
3	2	2	1	0	0	0	1	0	1	0	0	1	1

LIN-3	MPK-1	LIN-39	LS	LIN-12m	LIN-12i	CKI-1	EFL-1	LIN-35	SCF	APC	CDK-4/CYD-1	CDK-2/CYE-1	CDK-1/CYB-3
3	2	2	1	0	1	0	1	0	1	0	0	1	1

LIN-3	MPK-1	LIN-39	LS	LIN-12m	LIN-12i	CKI-1	EFL-1	LIN-35	SCF	APC	CDK-4/CYD-1	CDK-2/CYE-1	CDK-1/CYB-3
0	0	1	0	1	0	0	0	0	0	0	0	0	0
0	0	1	0	1	0	0	1	1	0	0	1	0	0
0	0	1	0	1	0	0	0	0	0	0	1	0	0
0	0	1	0	1	0	0	1	0	0	0	1	0	0
0	0	1	0	1	0	0	1	0	0	0	1	1	0
0	0	1	0	1	0	0	1	0	1	0	1	1	0
0	0	1	0	1	0	0	1	0	1	0	0	1	0
0	0	1	0	1	0	0	1	0	1	0	0	1	1
0	0	1	0	1	0	1	1	0	1	0	0	1	1
0	0	1	0	1	0	1	1	0	1	0	0	0	0
0	0	1	0	1	0	0	1	1	0	0	0	0	0
0	0	1	0	1	0	0	0	1	0	0	1	0	1

0	0	1	0	1	0	0	0	0	0	1	0	0	0
0	0	1	0	1	0	1	1	1	0	0	1	0	0

LIN-3	MPK-1	LIN-39	LS	LIN-12m	LIN-12i	CKI-1	EFL-1	LIN-35	SCF	APC	CDK-4/CYD-1	CDK-2/CYE-1	CDK-1/CYB-3
0	0	1	1	1	0	0	0	0	0	1	0	0	0
0	0	1	1	1	1	1	1	1	0	0	1	0	0
0	0	1	1	1	1	0	0	0	0	0	0	0	0
0	0	1	1	1	1	0	1	1	0	0	1	0	0
0	0	1	1	1	1	0	0	0	0	0	1	0	0
0	0	1	1	1	1	0	1	0	0	0	1	0	0
0	0	1	1	1	1	0	1	0	0	0	1	1	0
0	0	1	1	1	1	0	1	0	1	0	1	1	0
0	0	1	1	1	1	0	1	0	1	0	0	1	0
0	0	1	1	1	1	0	1	0	1	0	0	1	1
0	0	1	1	1	1	1	1	0	1	0	0	1	1
0	0	1	1	1	1	1	1	0	1	0	0	0	0
0	0	1	1	1	1	0	1	1	0	0	0	0	0
0	0	1	1	1	1	0	0	1	0	0	1	0	1

LIN-3	MPK-1	LIN-39	LS	LIN-12m	LIN-12i	CKI-1	EFL-1	LIN-35	SCF	APC	CDK-4/CYD-1	CDK-2/CYE-1	CDK-1/CYB-3
1	0	1	0	1	0	0	0	0	0	1	0	0	0
1	1	1	0	1	1	1	1	1	0	0	1	0	0
1	0	1	0	1	1	0	0	0	0	0	0	0	0
1	0	1	0	1	1	0	1	1	0	0	1	0	0
1	0	1	0	1	1	0	0	0	0	0	1	0	0
1	0	1	0	1	1	0	1	0	0	0	1	0	0
1	0	1	0	1	1	0	1	0	0	0	1	1	0
1	0	1	0	1	1	0	1	0	1	0	1	1	0
1	0	1	0	1	1	0	1	0	1	0	0	1	0
1	0	1	0	1	1	0	1	0	1	0	0	1	1
1	0	1	0	1	1	1	1	0	1	0	0	1	1
1	0	1	0	1	1	1	1	0	1	0	0	0	0
1	0	1	0	1	1	0	1	1	0	0	0	0	0
1	0	1	0	1	1	0	0	1	0	0	1	0	1

LIN-3	MPK-1	LIN-39	LS	LIN-12m	LIN-12i	CKI-1	EFL-1	LIN-35	SCF	APC	CDK-4/CYD-1	CDK-2/CYE-1	CDK-1/CYB-3
1	0	1	1	1	0	0	0	0	0	1	0	0	0
1	1	1	1	1	1	1	1	1	0	0	1	0	0
1	0	1	1	1	1	0	0	0	0	0	0	0	0
1	0	1	1	1	1	0	1	1	0	0	1	0	0
1	0	1	1	1	1	0	0	0	0	0	1	0	0
1	0	1	1	1	1	0	1	0	0	0	1	0	0
1	0	1	1	1	1	0	1	0	0	0	1	1	0
1	0	1	1	1	1	0	1	0	1	0	1	1	0
1	0	1	1	1	1	0	1	0	1	0	0	1	0
1	0	1	1	1	1	0	1	0	1	0	0	1	1
1	0	1	1	1	1	1	1	0	1	0	0	1	1
1	0	1	1	1	1	1	1	0	1	0	0	0	0
1	0	1	1	1	1	0	1	1	0	0	0	0	0
1	0	1	1	1	1	0	0	1	0	0	1	0	1

CKI-1 to CDK-1/CYB-3

LIN-3	MPK-1	LIN-39	LS	LIN-12m	LIN-12i	CKI-1	EFL-1	LIN-35	SCF	APC	CDK-4/CYD-1	CDK-2/CYE-1	CDK-1/CYB-3
2	1	1	1	1	0	0	0	1	0	1	0	0	1
2	2	1	1	1	0	0	0	1	0	1	0	0	0
2	2	2	1	0	1	0	0	1	0	0	1	0	0
2	1	2	1	1	1	0	0	0	0	0	1	0	0
2	1	1	1	1	1	0	1	0	0	0	1	0	0
2	1	1	1	1	1	0	1	0	0	0	1	1	0
2	1	1	1	1	1	0	1	0	1	0	1	1	0

2	1	1	1	1	1	0	1	0	1	0	0	0	0
2	1	1	1	1	1	0	1	1	0	0	0	0	1

LIN-3	MPK-1	LIN-39	LS	LIN-12m	LIN-12i	CKI-1	EFL-1	LIN-35	SCF	APC	CDK-4/CYD-1	CDK-2/CYE-1	CDK-1/CYB-3
2	2	2	0	0	0	0	0	1	0	0	1	0	0
2	2	2	0	1	0	0	0	0	0	0	1	0	0
2	2	2	0	1	0	0	1	0	0	0	1	0	0
2	2	2	0	1	0	0	1	0	0	0	1	1	0
2	2	2	0	1	0	0	1	0	1	0	1	1	0
2	2	2	0	1	0	0	1	0	1	0	0	0	0
2	2	2	0	0	0	0	1	1	0	0	0	0	1
2	2	2	0	0	0	0	0	1	0	1	0	0	1
2	2	2	0	0	0	0	0	1	0	1	0	0	0

LIN-3	MPK-1	LIN-39	LS	LIN-12m	LIN-12i	CKI-1	EFL-1	LIN-35	SCF	APC	CDK-4/CYD-1	CDK-2/CYE-1	CDK-1/CYB-3
3	2	2	0	0	0	0	0	1	0	0	1	0	0
3	2	2	0	1	0	0	0	0	0	0	1	0	0
3	2	2	0	1	0	0	1	0	0	0	1	0	0
3	2	2	0	1	0	0	1	0	0	0	1	1	0
3	2	2	0	1	0	0	1	0	1	0	1	1	0
3	2	2	0	1	0	0	1	0	1	0	0	0	0
3	2	2	0	0	0	0	1	1	0	0	0	0	1
3	2	2	0	0	0	0	0	1	0	1	0	0	1
3	2	2	0	0	0	0	0	1	0	1	0	0	0

LIN-3	MPK-1	LIN-39	LS	LIN-12m	LIN-12i	CKI-1	EFL-1	LIN-35	SCF	APC	CDK-4/CYD-1	CDK-2/CYE-1	CDK-1/CYB-3
3	2	2	1	0	0	0	0	1	0	0	1	0	0
3	2	2	1	1	0	0	0	0	0	0	1	0	0
3	2	2	1	1	1	0	1	0	0	0	1	0	0
3	2	2	1	1	1	0	1	0	0	0	1	1	0
3	2	2	1	1	1	0	1	0	1	0	1	1	0
3	2	2	1	1	1	0	1	0	1	0	0	0	0
3	2	2	1	0	1	0	1	1	0	0	0	0	1
3	2	2	1	0	0	0	0	1	0	1	0	0	1
3	2	2	1	0	0	0	0	1	0	1	0	0	0

LIN-3	MPK-1	LIN-39	LS	LIN-12m	LIN-12i	CKI-1	EFL-1	LIN-35	SCF	APC	CDK-4/CYD-1	CDK-2/CYE-1	CDK-1/CYB-3
1	0	1	0	1	0	1	0	1	0	1	0	0	1
1	1	1	0	1	0	1	0	1	0	1	0	0	0
1	1	1	0	1	1	0	0	1	0	0	0	0	0
1	0	1	0	1	1	0	0	1	0	0	1	0	0
1	0	1	0	1	1	0	0	0	0	0	1	0	0
1	0	1	0	1	1	0	1	0	0	0	1	0	0
1	0	1	0	1	1	0	1	0	0	0	1	1	0
1	0	1	0	1	1	0	1	0	1	0	1	1	0
1	0	1	0	1	1	0	1	0	1	0	0	0	0
1	0	1	0	1	1	0	1	1	0	0	0	0	1

LIN-3	MPK-1	LIN-39	LS	LIN-12m	LIN-12i	CKI-1	EFL-1	LIN-35	SCF	APC	CDK-4/CYD-1	CDK-2/CYE-1	CDK-1/CYB-3
1	0	1	1	1	0	1	0	1	0	1	0	0	1
1	1	1	1	1	0	1	0	1	0	1	0	0	0
1	1	1	1	1	1	0	0	1	0	0	0	0	0
1	0	1	1	1	1	0	0	1	0	0	1	0	0
1	0	1	1	1	1	0	0	0	0	0	1	0	0
1	0	1	1	1	1	0	1	0	0	0	1	0	0
1	0	1	1	1	1	0	1	0	0	0	1	1	0
1	0	1	1	1	1	0	1	0	1	0	1	1	0
1	0	1	1	1	1	0	1	0	1	0	0	0	0
1	0	1	1	1	1	0	1	1	0	0	0	0	1

LIN-3	MPK-1	LIN-39	LS	LIN-12m	LIN-12i	CKI-1	EFL-1	LIN-35	SCF	APC	CDK-4/CYD-1	CDK-2/CYE-1	CDK-1/CYB-3

0	0	1	0	1	0	0	0	0	0	0	1	0	0
0	0	1	0	1	0	0	1	0	0	0	1	0	0
0	0	1	0	1	0	0	1	0	0	0	1	1	0
0	0	1	0	1	0	0	1	0	1	0	1	1	0
0	0	1	0	1	0	0	1	0	1	0	0	0	0
0	0	1	0	1	0	0	1	1	0	0	0	0	1
0	0	1	0	1	0	1	0	1	0	1	0	0	1
0	0	1	0	1	0	1	0	1	0	1	0	0	0
0	0	1	0	1	0	1	0	1	0	0	0	0	0
0	0	1	0	1	0	0	0	1	0	0	0	0	0
0	0	1	0	1	0	0	0	1	0	0	1	0	0

LIN-3	MPK-1	LIN-39	LS	LIN-12m	LIN-12i	CKI-1	EFL-1	LIN-35	SCF	APC	CDK-4/CYD-1	CDK-2/CYE-1	CDK-1/CYB-3
0	0	1	1	1	0	1	0	1	0	1	0	0	0
0	0	1	1	1	1	1	0	1	0	0	0	0	0
0	0	1	1	1	1	0	0	1	0	0	0	0	0
0	0	1	1	1	1	0	0	1	0	0	1	0	0
0	0	1	1	1	1	0	0	0	0	0	1	0	0
0	0	1	1	1	1	0	1	0	0	0	1	0	0
0	0	1	1	1	1	0	1	0	0	0	1	1	0
0	0	1	1	1	1	0	1	0	1	0	1	1	0
0	0	1	1	1	1	0	1	0	1	0	0	0	0
0	0	1	1	1	1	0	1	1	0	0	0	0	1
0	0	1	1	1	0	1	0	1	0	1	0	0	1

EFL-1 to CDK-1/CYB-3

LIN-3	MPK-1	LIN-39	LS	LIN-12m	LIN-12i	CKI-1	EFL-1	LIN-35	SCF	APC	CDK-4/CYD-1	CDK-2/CYE-1	CDK-1/CYB-3
0	0	1	0	1	0	0	0	0	1	0	1	0	0
0	0	1	0	1	0	0	1	0	0	0	0	0	0
0	0	1	0	1	0	0	1	1	0	0	1	1	0

LIN-3	MPK-1	LIN-39	LS	LIN-12m	LIN-12i	CKI-1	EFL-1	LIN-35	SCF	APC	CDK-4/CYD-1	CDK-2/CYE-1	CDK-1/CYB-3
0	0	1	0	1	1	0	0	0	1	0	1	0	0
0	0	1	0	1	1	0	1	0	0	0	0	0	0
0	0	1	0	1	1	0	1	1	0	0	1	1	0

LIN-3	MPK-1	LIN-39	LS	LIN-12m	LIN-12i	CKI-1	EFL-1	LIN-35	SCF	APC	CDK-4/CYD-1	CDK-2/CYE-1	CDK-1/CYB-3
0	0	1	1	1	1	0	0	0	1	0	1	0	0
0	0	1	1	1	1	0	1	0	0	0	0	0	0
0	0	1	1	1	1	0	1	1	0	0	1	1	0

LIN-3	MPK-1	LIN-39	LS	LIN-12m	LIN-12i	CKI-1	EFL-1	LIN-35	SCF	APC	CDK-4/CYD-1	CDK-2/CYE-1	CDK-1/CYB-3
1	0	1	0	1	1	0	0	0	1	0	1	0	0
1	0	1	0	1	1	0	1	0	0	0	0	0	0
1	0	1	0	1	1	0	1	1	0	0	1	1	0

LIN-3	MPK-1	LIN-39	LS	LIN-12m	LIN-12i	CKI-1	EFL-1	LIN-35	SCF	APC	CDK-4/CYD-1	CDK-2/CYE-1	CDK-1/CYB-3
1	0	1	1	1	1	0	0	0	1	0	1	0	0
1	0	1	1	1	1	0	1	0	0	0	0	0	0
1	0	1	1	1	1	0	1	1	0	0	1	1	0

LIN-3	MPK-1	LIN-39	LS	LIN-12m	LIN-12i	CKI-1	EFL-1	LIN-35	SCF	APC	CDK-4/CYD-1	CDK-2/CYE-1	CDK-1/CYB-3
2	1	1	0	1	1	0	0	0	1	0	1	0	0
2	1	1	0	1	1	0	1	0	0	0	0	0	0
2	1	1	0	1	1	0	1	1	0	0	1	1	0

LIN-3	MPK-1	LIN-39	LS	LIN-12m	LIN-12i	CKI-1	EFL-1	LIN-35	SCF	APC	CDK-4/CYD-1	CDK-2/CYE-1	CDK-1/CYB-3
2	1	1	1	1	1	0	0	0	1	0	1	0	0
2	1	1	1	1	1	0	1	0	0	0	0	0	0
2	1	1	1	1	1	0	1	1	0	0	1	1	0

LIN-3	MPK-1	LIN-39	LS	LIN-12m	LIN-12i	CKI-1	EFL-1	LIN-35	SCF	APC	CDK-4/CYD-1	CDK-2/CYE-1	CDK-1/CYB-3
2	2	2	0	0	0	0	1	1	0	0	1	1	0
2	2	2	0	1	0	0	0	0	1	0	1	0	0
2	2	2	0	1	0	0	1	0	0	0	0	0	0

LIN-3	MPK-1	LIN-39	LS	LIN-12m	LIN-12i	CKI-1	EFL-1	LIN-35	SCF	APC	CDK-4/CYD-1	CDK-2/CYE-1	CDK-1/CYB-3
3	2	2	0	0	0	0	1	1	0	0	1	1	0
3	2	2	0	1	0	0	0	0	1	0	1	0	0
3	2	2	0	1	0	0	1	0	0	0	0	0	0

LIN-3	MPK-1	LIN-39	LS	LIN-12m	LIN-12i	CKI-1	EFL-1	LIN-35	SCF	APC	CDK-4/CYD-1	CDK-2/CYE-1	CDK-1/CYB-3
3	2	2	0	0	1	0	1	1	0	0	1	1	0
3	2	2	0	1	1	0	0	0	1	0	1	0	0
3	2	2	0	1	1	0	1	0	0	0	0	0	0

LIN-3	MPK-1	LIN-39	LS	LIN-12m	LIN-12i	CKI-1	EFL-1	LIN-35	SCF	APC	CDK-4/CYD-1	CDK-2/CYE-1	CDK-1/CYB-3
3	2	2	1	0	1	0	1	1	0	0	1	1	0
3	2	2	1	1	1	0	0	0	1	0	1	0	0
3	2	2	1	1	1	0	1	0	0	0	0	0	0

LIN-3	MPK-1	LIN-39	LS	LIN-12m	LIN-12i	CKI-1	EFL-1	LIN-35	SCF	APC	CDK-4/CYD-1	CDK-2/CYE-1	CDK-1/CYB-3
0	0	1	0	1	0	0	0	0	0	0	1	0	0
0	0	1	0	1	0	0	1	0	0	0	1	0	0
0	0	1	0	1	0	0	1	0	0	0	1	1	0
0	0	1	0	1	0	0	1	0	1	0	1	1	0
0	0	1	0	1	0	0	1	0	1	0	0	0	0
0	0	1	0	1	0	0	1	1	0	0	0	0	0
0	0	1	0	1	0	0	0	1	0	0	1	0	0

LIN-3	MPK-1	LIN-39	LS	LIN-12m	LIN-12i	CKI-1	EFL-1	LIN-35	SCF	APC	CDK-4/CYD-1	CDK-2/CYE-1	CDK-1/CYB-3
0	0	1	0	1	1	0	0	0	0	0	1	0	0
0	0	1	0	1	1	0	1	0	0	0	1	0	0
0	0	1	0	1	1	0	1	0	0	0	1	1	0
0	0	1	0	1	1	0	1	0	1	0	1	1	0
0	0	1	0	1	1	0	1	0	1	0	0	0	0
0	0	1	0	1	1	0	1	1	0	0	0	0	0
0	0	1	0	1	1	0	0	1	0	0	1	0	0

LIN-3	MPK-1	LIN-39	LS	LIN-12m	LIN-12i	CKI-1	EFL-1	LIN-35	SCF	APC	CDK-4/CYD-1	CDK-2/CYE-1	CDK-1/CYB-3
0	0	1	1	1	1	0	0	0	0	0	1	0	0
0	0	1	1	1	1	0	1	0	0	0	1	0	0
0	0	1	1	1	1	0	1	0	0	0	1	1	0
0	0	1	1	1	1	0	1	0	1	0	1	1	0
0	0	1	1	1	1	0	1	0	1	0	0	0	0
0	0	1	1	1	1	0	1	1	0	0	0	0	0
0	0	1	1	1	1	0	0	1	0	0	1	0	0

LIN-3	MPK-1	LIN-39	LS	LIN-12m	LIN-12i	CKI-1	EFL-1	LIN-35	SCF	APC	CDK-4/CYD-1	CDK-2/CYE-1	CDK-1/CYB-3
1	0	1	0	1	1	0	0	0	0	0	1	0	0
1	0	1	0	1	1	0	1	0	0	0	1	0	0
1	0	1	0	1	1	0	1	0	0	0	1	1	0
1	0	1	0	1	1	0	1	0	1	0	1	1	0
1	0	1	0	1	1	0	1	0	1	0	0	0	0
1	0	1	0	1	1	0	1	1	0	0	0	0	0
1	0	1	0	1	1	0	0	1	0	0	1	0	0

LIN-3	MPK-1	LIN-39	LS	LIN-12m	LIN-12i	CKI-1	EFL-1	LIN-35	SCF	APC	CDK-4/CYD-1	CDK-2/CYE-1	CDK-1/CYB-3
1	0	1	1	1	1	0	0	0	0	0	1	0	0
1	0	1	1	1	1	0	1	0	0	0	1	0	0
1	0	1	1	1	1	0	1	0	0	0	1	1	0

1	0	1	1	1	1	0	1	0	1	0	1	1	0
1	0	1	1	1	1	0	1	0	1	0	0	0	0
1	0	1	1	1	1	0	1	1	0	0	0	0	0
1	0	1	1	1	1	0	0	1	0	0	1	0	0

LIN-3	MPK-1	LIN-39	LS	LIN-12m	LIN-12i	CKI-1	EFL-1	LIN-35	SCF	APC	CDK-4/CYD-1	CDK-2/CYE-1	CDK-1/CYB-3
2	1	1	0	1	1	0	0	0	0	0	1	0	0
2	1	1	0	1	1	0	1	0	0	0	1	0	0
2	1	1	0	1	1	0	1	0	0	0	1	1	0
2	1	1	0	1	1	0	1	0	1	0	1	1	0
2	1	1	0	1	1	0	1	0	1	0	0	0	0
2	1	1	0	1	1	0	1	1	0	0	0	0	0
2	1	1	0	1	1	0	0	1	0	0	1	0	0

LIN-3	MPK-1	LIN-39	LS	LIN-12m	LIN-12i	CKI-1	EFL-1	LIN-35	SCF	APC	CDK-4/CYD-1	CDK-2/CYE-1	CDK-1/CYB-3
2	1	1	1	1	1	0	0	0	0	0	1	0	0
2	1	1	1	1	1	0	1	0	0	0	1	0	0
2	1	1	1	1	1	0	1	0	0	0	1	1	0
2	1	1	1	1	1	0	1	0	1	0	1	1	0
2	1	1	1	1	1	0	1	0	1	0	0	0	0
2	1	1	1	1	1	0	1	1	0	0	0	0	0
2	1	1	1	1	1	0	0	1	0	0	1	0	0

LIN-3	MPK-1	LIN-39	LS	LIN-12m	LIN-12i	CKI-1	EFL-1	LIN-35	SCF	APC	CDK-4/CYD-1	CDK-2/CYE-1	CDK-1/CYB-3
2	2	2	0	0	0	0	0	1	0	0	1	0	0
2	2	2	0	1	0	0	0	0	0	0	1	0	0
2	2	2	0	1	0	0	1	0	0	0	1	0	0
2	2	2	0	1	0	0	1	0	0	0	1	1	0
2	2	2	0	1	0	0	1	0	1	0	1	1	0
2	2	2	0	1	0	0	1	0	1	0	0	0	0
2	2	2	0	0	0	0	1	1	0	0	0	0	0

LIN-3	MPK-1	LIN-39	LS	LIN-12m	LIN-12i	CKI-1	EFL-1	LIN-35	SCF	APC	CDK-4/CYD-1	CDK-2/CYE-1	CDK-1/CYB-3
3	2	2	0	0	0	0	0	1	0	0	1	0	0
3	2	2	0	1	0	0	0	0	0	0	1	0	0
3	2	2	0	1	0	0	1	0	0	0	1	0	0
3	2	2	0	1	0	0	1	0	0	0	1	1	0
3	2	2	0	1	0	0	1	0	1	0	1	1	0
3	2	2	0	1	0	0	1	0	1	0	0	0	0
3	2	2	0	0	0	0	1	1	0	0	0	0	0

LIN-3	MPK-1	LIN-39	LS	LIN-12m	LIN-12i	CKI-1	EFL-1	LIN-35	SCF	APC	CDK-4/CYD-1	CDK-2/CYE-1	CDK-1/CYB-3
3	2	2	0	0	1	0	0	1	0	0	1	0	0
3	2	2	0	1	1	0	0	0	0	0	1	0	0
3	2	2	0	1	1	0	1	0	0	0	1	0	0
3	2	2	0	1	1	0	1	0	0	0	1	1	0
3	2	2	0	1	1	0	1	0	1	0	1	1	0
3	2	2	0	1	1	0	1	0	1	0	0	0	0
3	2	2	0	0	1	0	1	1	0	0	0	0	0

LIN-3	MPK-1	LIN-39	LS	LIN-12m	LIN-12i	CKI-1	EFL-1	LIN-35	SCF	APC	CDK-4/CYD-1	CDK-2/CYE-1	CDK-1/CYB-3
3	2	2	1	0	1	0	0	1	0	0	1	0	0
3	2	2	1	1	1	0	0	0	0	0	1	0	0
3	2	2	1	1	1	0	1	0	0	0	1	0	0
3	2	2	1	1	1	0	1	0	0	0	1	1	0
3	2	2	1	1	1	0	1	0	1	0	1	1	0
3	2	2	1	1	1	0	1	0	1	0	0	0	0
3	2	2	1	0	1	0	1	1	0	0	0	0	0

APC to CDK-1/CYB-3

LIN-3	MPK-1	LIN-39	LS	LIN-12m	LIN-12i	CKI-1	EFL-1	LIN-35	SCF	APC	CDK-4/CYD-1	CDK-2/CYE-1	CDK-1/CYB-3
2	1	1	0	1	1	0	1	0	0	1	0	1	1

LIN-3	MPK-1	LIN-39	LS	LIN-12m	LIN-12i	CKI-1	EFL-1	LIN-35	SCF	APC	CDK-4/CYD-1	CDK-2/CYE-1	CDK-1/CYB-3
2	1	1	1	1	1	0	1	0	0	1	0	1	1

LIN-3	MPK-1	LIN-39	LS	LIN-12m	LIN-12i	CKI-1	EFL-1	LIN-35	SCF	APC	CDK-4/CYD-1	CDK-2/CYE-1	CDK-1/CYB-3
2	2	2	0	0	0	0	1	0	0	1	0	1	1

LIN-3	MPK-1	LIN-39	LS	LIN-12m	LIN-12i	CKI-1	EFL-1	LIN-35	SCF	APC	CDK-4/CYD-1	CDK-2/CYE-1	CDK-1/CYB-3
2	2	2	1	0	0	0	1	0	0	1	0	1	1

LIN-3	MPK-1	LIN-39	LS	LIN-12m	LIN-12i	CKI-1	EFL-1	LIN-35	SCF	APC	CDK-4/CYD-1	CDK-2/CYE-1	CDK-1/CYB-3
3	2	2	0	0	0	0	1	0	0	1	0	1	1

LIN-3	MPK-1	LIN-39	LS	LIN-12m	LIN-12i	CKI-1	EFL-1	LIN-35	SCF	APC	CDK-4/CYD-1	CDK-2/CYE-1	CDK-1/CYB-3
3	2	2	0	0	1	0	1	0	0	1	0	1	1

LIN-3	MPK-1	LIN-39	LS	LIN-12m	LIN-12i	CKI-1	EFL-1	LIN-35	SCF	APC	CDK-4/CYD-1	CDK-2/CYE-1	CDK-1/CYB-3
3	2	2	1	0	0	0	1	0	0	1	0	1	1

LIN-3	MPK-1	LIN-39	LS	LIN-12m	LIN-12i	CKI-1	EFL-1	LIN-35	SCF	APC	CDK-4/CYD-1	CDK-2/CYE-1	CDK-1/CYB-3
3	2	2	1	0	1	0	1	0	0	1	0	1	1

LIN-3	MPK-1	LIN-39	LS	LIN-12m	LIN-12i	CKI-1	EFL-1	LIN-35	SCF	APC	CDK-4/CYD-1	CDK-2/CYE-1	CDK-1/CYB-3
2	1	1	1	1	0	0	0	1	0	1	0	0	1
2	2	1	1	1	0	0	0	1	0	1	0	0	0
2	2	2	1	0	1	0	0	1	0	0	1	0	0
2	1	2	1	1	1	0	0	0	0	0	1	0	0
2	1	1	1	1	1	0	1	0	0	0	1	0	0
2	1	1	1	1	1	0	1	0	0	0	1	1	0
2	1	1	1	1	1	0	1	0	1	0	1	1	0
2	1	1	1	1	1	0	1	0	1	0	0	0	0
2	1	1	1	1	1	0	1	1	0	0	0	0	1

LIN-3	MPK-1	LIN-39	LS	LIN-12m	LIN-12i	CKI-1	EFL-1	LIN-35	SCF	APC	CDK-4/CYD-1	CDK-2/CYE-1	CDK-1/CYB-3
2	2	2	0	0	0	0	0	1	0	0	1	0	0
2	2	2	0	1	0	0	0	0	0	0	1	0	0
2	2	2	0	1	0	0	1	0	0	0	1	0	0
2	2	2	0	1	0	0	1	0	0	0	1	1	0
2	2	2	0	1	0	0	1	0	1	0	1	1	0
2	2	2	0	1	0	0	1	0	1	0	0	0	0
2	2	2	0	0	0	0	1	1	0	0	0	0	1
2	2	2	0	0	0	0	0	1	0	1	0	0	1
2	2	2	0	0	0	0	0	1	0	1	0	0	0

LIN-3	MPK-1	LIN-39	LS	LIN-12m	LIN-12i	CKI-1	EFL-1	LIN-35	SCF	APC	CDK-4/CYD-1	CDK-2/CYE-1	CDK-1/CYB-3
3	2	2	0	0	0	0	0	1	0	0	1	0	0
3	2	2	0	1	0	0	0	0	0	0	1	0	0
3	2	2	0	1	0	0	1	0	0	0	1	0	0
3	2	2	0	1	0	0	1	0	0	0	1	1	0
3	2	2	0	1	0	0	1	0	1	0	1	1	0
3	2	2	0	1	0	0	1	0	1	0	0	0	0
3	2	2	0	0	0	0	1	1	0	0	0	0	1
3	2	2	0	0	0	0	0	1	0	1	0	0	1
3	2	2	0	0	0	0	0	1	0	1	0	0	0

LIN-3	MPK-1	LIN-39	LS	LIN-12m	LIN-12i	CKI-1	EFL-1	LIN-35	SCF	APC	CDK-4/CYD-1	CDK-2/CYE-1	CDK-1/CYB-3
3	2	2	1	0	0	0	0	1	0	0	1	0	0
3	2	2	1	1	0	0	0	0	0	0	1	0	0
3	2	2	1	1	1	0	1	0	0	0	1	0	0

3	2	2	1	1	1	0	1	0	0	0	1	1	0
3	2	2	1	1	1	0	1	0	1	0	1	1	0
3	2	2	1	1	1	0	1	0	1	0	0	0	0
3	2	2	1	0	1	0	1	1	0	0	0	0	1
3	2	2	1	0	0	0	0	1	0	1	0	0	1
3	2	2	1	0	0	0	0	1	0	1	0	0	0

LIN-3	MPK-1	LIN-39	LS	LIN-12m	LIN-12i	CKI-1	EFL-1	LIN-35	SCF	APC	CDK-4/CYD-1	CDK-2/CYE-1	CDK-1/CYB-3
1	0	1	0	1	0	1	0	1	0	1	0	0	1
1	1	1	0	1	0	1	0	1	0	1	0	0	0
1	1	1	0	1	1	0	0	1	0	0	0	0	0
1	0	1	0	1	1	0	0	1	0	0	1	0	0
1	0	1	0	1	1	0	0	0	0	0	1	0	0
1	0	1	0	1	1	0	1	0	0	0	1	0	0
1	0	1	0	1	1	0	1	0	0	0	1	1	0
1	0	1	0	1	1	0	1	0	1	0	1	1	0
1	0	1	0	1	1	0	1	0	1	0	0	0	0
1	0	1	0	1	1	0	1	1	0	0	0	0	1

LIN-3	MPK-1	LIN-39	LS	LIN-12m	LIN-12i	CKI-1	EFL-1	LIN-35	SCF	APC	CDK-4/CYD-1	CDK-2/CYE-1	CDK-1/CYB-3
1	0	1	1	1	0	1	0	1	0	1	0	0	1
1	1	1	1	1	0	1	0	1	0	1	0	0	0
1	1	1	1	1	1	0	0	1	0	0	0	0	0
1	0	1	1	1	1	0	0	1	0	0	1	0	0
1	0	1	1	1	1	0	0	0	0	0	1	0	0
1	0	1	1	1	1	0	1	0	0	0	1	0	0
1	0	1	1	1	1	0	1	0	0	0	1	1	0
1	0	1	1	1	1	0	1	0	1	0	1	1	0
1	0	1	1	1	1	0	1	0	1	0	0	0	0
1	0	1	1	1	1	0	1	1	0	0	0	0	1

LIN-3	MPK-1	LIN-39	LS	LIN-12m	LIN-12i	CKI-1	EFL-1	LIN-35	SCF	APC	CDK-4/CYD-1	CDK-2/CYE-1	CDK-1/CYB-3
0	0	1	0	1	0	0	0	0	0	0	1	0	0
0	0	1	0	1	0	0	1	0	0	0	1	0	0
0	0	1	0	1	0	0	1	0	0	0	1	1	0
0	0	1	0	1	0	0	1	0	1	0	1	1	0
0	0	1	0	1	0	0	1	0	1	0	0	0	0
0	0	1	0	1	0	0	1	1	0	0	0	0	1
0	0	1	0	1	0	1	0	1	0	1	0	0	1
0	0	1	0	1	0	1	0	1	0	1	0	0	0
0	0	1	0	1	0	1	0	1	0	0	0	0	0
0	0	1	0	1	0	0	0	1	0	0	0	0	0
0	0	1	0	1	0	0	0	1	0	0	1	0	0

LIN-3	MPK-1	LIN-39	LS	LIN-12m	LIN-12i	CKI-1	EFL-1	LIN-35	SCF	APC	CDK-4/CYD-1	CDK-2/CYE-1	CDK-1/CYB-3
0	0	1	1	1	0	1	0	1	0	1	0	0	0
0	0	1	1	1	1	1	0	1	0	0	0	0	0
0	0	1	1	1	1	0	0	1	0	0	0	0	0
0	0	1	1	1	1	0	0	1	0	0	1	0	0
0	0	1	1	1	1	0	0	0	0	0	1	0	0
0	0	1	1	1	1	0	1	0	0	0	1	0	0
0	0	1	1	1	1	0	1	0	0	0	1	1	0
0	0	1	1	1	1	0	1	0	1	0	1	1	0
0	0	1	1	1	1	0	1	0	1	0	0	0	0
0	0	1	1	1	1	0	1	1	0	0	0	0	1
0	0	1	1	1	0	1	0	1	0	1	0	0	1

CDK-4/CYD-1 to CDK-1/CYB-3

LIN-3	MPK-1	LIN-39	LS	LIN-12m	LIN-12i	CKI-1	EFL-1	LIN-35	SCF	APC	CDK-4/CYD-1	CDK-2/CYE-1	CDK-1/CYB-3
0	0	1	0	1	0	0	0	0	0	0	0	0	0

0	0	1	0	1	0	0	1	1	0	0	1	0	0
0	0	1	0	1	0	0	0	0	0	0	1	0	1
0	0	1	0	1	0	0	1	0	0	1	0	0	0
0	0	1	0	1	0	1	1	1	0	0	1	1	0
0	0	1	0	1	0	0	0	0	1	0	0	0	0
0	0	1	0	1	0	0	1	1	0	0	0	0	0
0	0	1	0	1	0	0	0	1	0	0	1	0	1
0	0	1	0	1	0	0	0	0	0	1	0	0	0
0	0	1	0	1	0	1	1	1	0	0	1	0	0

LIN-3	MPK-1	LIN-39	LS	LIN-12m	LIN-12i	CKI-1	EFL-1	LIN-35	SCF	APC	CDK-4/CYD-1	CDK-2/CYE-1	CDK-1/CYB-3
0	0	1	1	1	0	0	0	0	0	1	0	0	0
0	0	1	1	1	1	1	1	1	0	0	1	0	0
0	0	1	1	1	1	0	0	0	0	0	0	0	0
0	0	1	1	1	1	0	1	1	0	0	1	0	0
0	0	1	1	1	1	0	0	0	0	0	1	0	1
0	0	1	1	1	0	0	1	0	0	1	0	0	0
0	0	1	1	1	1	1	1	1	0	0	1	1	0
0	0	1	1	1	1	0	0	0	1	0	0	0	0
0	0	1	1	1	1	0	1	1	0	0	0	0	0
0	0	1	1	1	1	0	0	1	0	0	1	0	1

LIN-3	MPK-1	LIN-39	LS	LIN-12m	LIN-12i	CKI-1	EFL-1	LIN-35	SCF	APC	CDK-4/CYD-1	CDK-2/CYE-1	CDK-1/CYB-3
1	0	1	0	1	0	0	0	0	0	1	0	0	0
1	1	1	0	1	1	1	1	1	0	0	1	0	0
1	0	1	0	1	1	0	0	0	0	0	0	0	0
1	0	1	0	1	1	0	1	1	0	0	1	0	0
1	0	1	0	1	1	0	0	0	0	0	1	0	1
1	0	1	0	1	0	0	1	0	0	1	0	0	0
1	1	1	0	1	1	1	1	1	0	0	1	1	0
1	0	1	0	1	1	0	0	0	1	0	0	0	0
1	0	1	0	1	1	0	1	1	0	0	0	0	0
1	0	1	0	1	1	0	0	1	0	0	1	0	1

LIN-3	MPK-1	LIN-39	LS	LIN-12m	LIN-12i	CKI-1	EFL-1	LIN-35	SCF	APC	CDK-4/CYD-1	CDK-2/CYE-1	CDK-1/CYB-3
1	0	1	1	1	0	0	0	0	0	1	0	0	0
1	1	1	1	1	1	1	1	1	0	0	1	0	0
1	0	1	1	1	1	0	0	0	0	0	0	0	0
1	0	1	1	1	1	0	1	1	0	0	1	0	0
1	0	1	1	1	1	0	0	0	0	0	1	0	1
1	0	1	1	1	0	0	1	0	0	1	0	0	0
1	1	1	1	1	1	1	1	1	0	0	1	1	0
1	0	1	1	1	1	0	0	0	1	0	0	0	0
1	0	1	1	1	1	0	1	1	0	0	0	0	0
1	0	1	1	1	1	0	0	1	0	0	1	0	1

LIN-3	MPK-1	LIN-39	LS	LIN-12m	LIN-12i	CKI-1	EFL-1	LIN-35	SCF	APC	CDK-4/CYD-1	CDK-2/CYE-1	CDK-1/CYB-3
2	1	1	1	1	0	0	1	0	0	0	0	0	0
2	2	1	1	1	1	0	1	1	0	0	1	1	1
2	1	2	1	1	1	0	0	0	1	1	0	0	1
2	1	1	1	1	0	0	1	1	0	0	0	0	0
2	2	1	1	1	1	0	0	1	0	0	1	0	1
2	1	2	1	1	0	0	0	0	0	1	0	0	0
2	2	1	1	1	1	0	1	1	0	0	1	0	0
2	1	2	1	1	1	0	0	0	0	0	1	0	1
2	1	1	1	1	0	0	1	0	0	1	0	0	0
2	2	1	1	1	1	0	1	1	0	0	1	1	0
2	1	2	1	1	1	0	0	0	1	0	1	0	1

LIN-3	MPK-1	LIN-39	LS	LIN-12m	LIN-12i	CKI-1	EFL-1	LIN-35	SCF	APC	CDK-4/CYD-1	CDK-2/CYE-1	CDK-1/CYB-3
2	2	2	0	0	0	0	0	1	0	0	1	0	1

2	2	2	0	1	0	0	0	0	0	1	0	0	0
2	2	2	0	0	0	0	1	1	0	0	1	0	0
2	2	2	0	1	0	0	0	0	0	0	1	0	1
2	2	2	0	1	0	0	1	0	0	1	0	0	0
2	2	2	0	0	0	0	1	1	0	0	1	1	0
2	2	2	0	1	0	0	0	0	1	0	1	0	1
2	2	2	0	1	0	0	1	0	0	0	0	0	0
2	2	2	0	0	0	0	1	1	0	0	1	1	1
2	2	2	0	1	0	0	0	0	1	1	0	0	1
2	2	2	0	0	0	0	1	1	0	0	0	0	0

LIN-3	MPK-1	LIN-39	LS	LIN-12m	LIN-12i	CKI-1	EFL-1	LIN-35	SCF	APC	CDK-4/CYD-1	CDK-2/CYE-1	CDK-1/CYB-3
3	2	2	0	0	0	0	0	1	0	0	1	0	1
3	2	2	0	1	0	0	0	0	0	1	0	0	0
3	2	2	0	0	0	0	1	1	0	0	1	0	0
3	2	2	0	1	0	0	0	0	0	0	1	0	1
3	2	2	0	1	0	0	1	0	0	1	0	0	0
3	2	2	0	0	0	0	1	1	0	0	1	1	0
3	2	2	0	1	0	0	0	0	1	0	1	0	1
3	2	2	0	1	0	0	1	0	0	0	0	0	0
3	2	2	0	0	0	0	1	1	0	0	1	1	1
3	2	2	0	1	0	0	0	0	1	1	0	0	1
3	2	2	0	0	0	0	1	1	0	0	0	0	0

LIN-3	MPK-1	LIN-39	LS	LIN-12m	LIN-12i	CKI-1	EFL-1	LIN-35	SCF	APC	CDK-4/CYD-1	CDK-2/CYE-1	CDK-1/CYB-3
3	2	2	1	0	0	0	0	1	0	0	1	0	1
3	2	2	1	1	0	0	0	0	0	1	0	0	0
3	2	2	1	0	1	0	1	1	0	0	1	0	0
3	2	2	1	1	1	0	0	0	0	0	1	0	1
3	2	2	1	1	0	0	1	0	0	1	0	0	0
3	2	2	1	0	1	0	1	1	0	0	1	1	0
3	2	2	1	1	1	0	0	0	1	0	1	0	1
3	2	2	1	1	0	0	1	0	0	0	0	0	0
3	2	2	1	0	1	0	1	1	0	0	1	1	1
3	2	2	1	1	1	0	0	0	1	1	0	0	1
3	2	2	1	0	0	0	1	1	0	0	0	0	0

CDK-1/CYB-3 to CDK-1/CYB-3

LIN-3	MPK-1	LIN-39	LS	LIN-12m	LIN-12i	CKI-1	EFL-1	LIN-35	SCF	APC	CDK-4/CYD-1	CDK-2/CYE-1	CDK-1/CYB-3
2	1	1	1	1	0	0	0	1	0	1	0	0	1
2	2	1	1	1	0	0	0	1	0	1	0	0	0
2	2	2	1	0	1	0	0	1	0	0	1	0	0
2	1	2	1	1	1	0	0	0	0	0	1	0	0
2	1	1	1	1	1	0	1	0	0	0	1	0	0
2	1	1	1	1	1	0	1	0	0	0	1	1	0
2	1	1	1	1	1	0	1	0	1	0	1	1	0
2	1	1	1	1	1	0	1	0	1	0	0	0	0
2	1	1	1	1	1	0	1	1	0	0	0	0	1

LIN-3	MPK-1	LIN-39	LS	LIN-12m	LIN-12i	CKI-1	EFL-1	LIN-35	SCF	APC	CDK-4/CYD-1	CDK-2/CYE-1	CDK-1/CYB-3
2	2	2	0	0	0	0	0	1	0	0	1	0	0
2	2	2	0	1	0	0	0	0	0	0	1	0	0
2	2	2	0	1	0	0	1	0	0	0	1	0	0
2	2	2	0	1	0	0	1	0	0	0	1	1	0
2	2	2	0	1	0	0	1	0	1	0	1	1	0
2	2	2	0	1	0	0	1	0	1	0	0	0	0
2	2	2	0	0	0	0	1	1	0	0	0	0	1
2	2	2	0	0	0	0	0	1	0	1	0	0	1
2	2	2	0	0	0	0	0	1	0	1	0	0	0

LIN-3	MPK-1	LIN-39	LS	LIN-12m	LIN-12i	CKI-1	EFL-1	LIN-35	SCF	APC	CDK-4/CYD-1	CDK-2/CYE-1	CDK-1/CYB-3
3	2	2	0	0	0	0	0	1	0	0	1	0	0
3	2	2	0	1	0	0	0	0	0	0	1	0	0
3	2	2	0	1	0	0	1	0	0	0	1	0	0
3	2	2	0	1	0	0	1	0	0	0	1	1	0
3	2	2	0	1	0	0	1	0	1	0	1	1	0
3	2	2	0	1	0	0	1	0	1	0	0	0	0
3	2	2	0	0	0	0	1	1	0	0	0	0	1
3	2	2	0	0	0	0	0	1	0	1	0	0	1
3	2	2	0	0	0	0	0	1	0	1	0	0	0

LIN-3	MPK-1	LIN-39	LS	LIN-12m	LIN-12i	CKI-1	EFL-1	LIN-35	SCF	APC	CDK-4/CYD-1	CDK-2/CYE-1	CDK-1/CYB-3
3	2	2	1	0	0	0	0	1	0	0	1	0	0
3	2	2	1	1	0	0	0	0	0	0	1	0	0
3	2	2	1	1	1	0	1	0	0	0	1	0	0
3	2	2	1	1	1	0	1	0	0	0	1	1	0
3	2	2	1	1	1	0	1	0	1	0	1	1	0
3	2	2	1	1	1	0	1	0	1	0	0	0	0
3	2	2	1	0	1	0	1	1	0	0	0	0	1
3	2	2	1	0	0	0	0	1	0	1	0	0	1
3	2	2	1	0	0	0	0	1	0	1	0	0	0

LIN-3	MPK-1	LIN-39	LS	LIN-12m	LIN-12i	CKI-1	EFL-1	LIN-35	SCF	APC	CDK-4/CYD-1	CDK-2/CYE-1	CDK-1/CYB-3
1	0	1	0	1	0	1	0	1	0	1	0	0	1
1	1	1	0	1	0	1	0	1	0	1	0	0	0
1	1	1	0	1	1	0	0	1	0	0	0	0	0
1	0	1	0	1	1	0	0	1	0	0	1	0	0
1	0	1	0	1	1	0	0	0	0	0	1	0	0
1	0	1	0	1	1	0	1	0	0	0	1	0	0
1	0	1	0	1	1	0	1	0	0	0	1	1	0
1	0	1	0	1	1	0	1	0	1	0	1	1	0
1	0	1	0	1	1	0	1	0	1	0	0	0	0
1	0	1	0	1	1	0	1	1	0	0	0	0	1

LIN-3	MPK-1	LIN-39	LS	LIN-12m	LIN-12i	CKI-1	EFL-1	LIN-35	SCF	APC	CDK-4/CYD-1	CDK-2/CYE-1	CDK-1/CYB-3
1	0	1	1	1	0	1	0	1	0	1	0	0	1
1	1	1	1	1	0	1	0	1	0	1	0	0	0
1	1	1	1	1	1	0	0	1	0	0	0	0	0
1	0	1	1	1	1	0	0	1	0	0	1	0	0
1	0	1	1	1	1	0	0	0	0	0	1	0	0
1	0	1	1	1	1	0	1	0	0	0	1	0	0
1	0	1	1	1	1	0	1	0	0	0	1	1	0
1	0	1	1	1	1	0	1	0	1	0	1	1	0
1	0	1	1	1	1	0	1	0	1	0	0	0	0
1	0	1	1	1	1	0	1	1	0	0	0	0	1

LIN-3	MPK-1	LIN-39	LS	LIN-12m	LIN-12i	CKI-1	EFL-1	LIN-35	SCF	APC	CDK-4/CYD-1	CDK-2/CYE-1	CDK-1/CYB-3
0	0	1	0	1	0	0	0	0	0	0	1	0	0
0	0	1	0	1	0	0	1	0	0	0	1	0	0
0	0	1	0	1	0	0	1	0	0	0	1	1	0
0	0	1	0	1	0	0	1	0	1	0	1	1	0
0	0	1	0	1	0	0	1	0	1	0	0	0	0
0	0	1	0	1	0	0	1	1	0	0	0	0	1
0	0	1	0	1	0	1	0	1	0	1	0	0	1
0	0	1	0	1	0	1	0	1	0	1	0	0	0
0	0	1	0	1	0	1	0	1	0	0	0	0	0
0	0	1	0	1	0	0	0	1	0	0	0	0	0
0	0	1	0	1	0	0	0	1	0	0	1	0	0

LIN-3	MPK-1	LIN-39	LS	LIN-12m	LIN-12i	CKI-1	EFL-1	LIN-35	SCF	APC	CDK-4/CYD-1	CDK-2/CYE-1	CDK-1/CYB-3
0	0	1	1	1	0	1	0	1	0	1	0	0	0

0	0	1	1	1	1	1	0	1	0	0	0	0	0
0	0	1	1	1	1	0	0	1	0	0	0	0	0
0	0	1	1	1	1	0	0	1	0	0	1	0	0
0	0	1	1	1	1	0	0	0	0	0	1	0	0
0	0	1	1	1	1	0	1	0	0	0	1	0	0
0	0	1	1	1	1	0	1	0	0	0	1	1	0
0	0	1	1	1	1	0	1	0	1	0	1	1	0
0	0	1	1	1	1	0	1	0	1	0	0	0	0
0	0	1	1	1	1	0	1	1	0	0	0	0	1
0	0	1	1	1	0	1	0	1	0	1	0	0	1
